# Taming 2,2′-biimidazole ligands in trivalent chromium complexes†

**DOI:** 10.1039/d4dt01608d

**Published:** 2024-07-06

**Authors:** Julien Chong, Amina Benchohra, Céline Besnard, Laure Guénée, Arnulf Rosspeintner, Carlos M. Cruz, Juan-Ramón Jiménez, Claude Piguet

**Affiliations:** a Department of Inorganic and Analytical Chemistry, University of Geneva 30 quai E. Ansermet CH-1211 Geneva 4 Switzerland Claude.Piguet@unige.ch; b Laboratoire CEMCA UMR, CNRS 6521, UFR Sciences and Techniques 6 avenue Victor Le Gorgeu 29238 Brest Cedex 3 France; c Laboratory of Crystallography, University of Geneva 24 quai E. Ansermet CH-1211 Geneva 4 Switzerland; d Department of Physical Chemistry, University of Geneva 30 quai E. Ansermet CH-1211 Geneva 4 Switzerland; e Department of Organic Chemistry, Unidad de Excelencia de Química (UEQ), University of Granada Avda. Fuente Nueva s/n 18071 Granada Spain; f Departamento de Química Inorgánica, Facultad de Ciencias, Universidad de Granada and Unidad de Excelencia en Quımica (UEQ) Avda. Fuente Nueva s/n 18071 Granada Spain

## Abstract

Complete or partial replacement of well-known five-membered chelating 2,2′-bipyridine (bipy) or 1,10-phenanthroline (phen) ligands with analogous didentate 2,2′-biimidazole (H_2_biim) provides novel perspectives for exploiting the latter pH-tuneable bridging unit for connecting inert trivalent chromium with cationic partners. The most simple homoleptic complex [Cr(H_2_biim)_3_]^3+^ and its stepwise deprotonated analogues are only poorly soluble in most solvents and their characterization is limited to some solid-state structures, in which the pseudo-octahedral [CrN_6_] units are found to be intermolecularly connected *via* peripheral N–H⋯X hydrogen bonds. Moreover, the associated high-energy stretching N–H vibrations drastically quench the targeted near infrared (NIR) Cr^III^-based phosphorescence, which makes these homoleptic building blocks incompatible with the design of molecular-based luminescent assemblies. Restricting the number of bound 2,2′-biimidazole ligands to a single unit in the challenging heteroleptic [Cr(phen)_2_(H_*x*_biim)]^(1+*x*)+^ (*x* = 2–0) complexes overcomes the latter limitations and allows (i) the synthesis and characterization of these [CrN_6_] chromophores in the solid state and in solution, (ii) the stepwise and controlled deprotonation of the bound 2,2′-biimidazole ligand and (iii) the implementation of Cr-centered phosphorescence with energies, lifetimes and quantum yields adapted for using the latter chromophores as sensitizers in promising ‘complex-as-ligand’ strategies.

## Abstract 2

Moving from homoleptic [Cr(biimidazole)_3_]^3+^ toward heteroleptic [Cr(phen)_2_(biimidazole)]^3+^ restores solubility, stability and pH-dependent near infrared phosphorescence at the molecular level.

## Graphical abstract

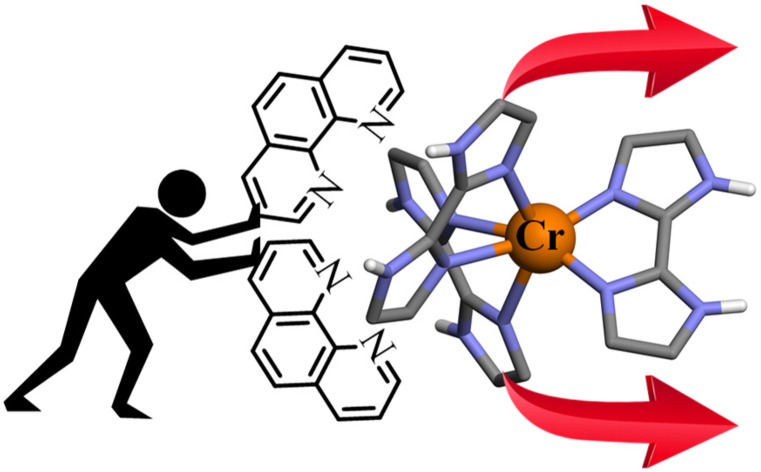

## Introduction

The lack of first-order orbital momentum in pseudo-octahedral trivalent chromium complexes makes isotropic and spin-only [CrX_6_] units ideal partners for programming, tuning and rationalizing magnetic coupling with neighbouring paramagnetic d-block^1–5^ and f-block^6–10^ cations. In this context, many of the chromium-containing heterometallic assemblies exploit the kinetically inert [Cr(CN)_6_]^3−^ synthon, which behaves as a ‘complex-as-ligand’ connecting cationic metals *via* cyanide bridges in solid state materials.^1–10^ The realization that, beyond magnetic properties, both lanthanide-based light downshifting^11,12^ and light upconversion^13,14^ can be boosted by close Cr(iii) sensitizers/emitters resulted in some active search for novel synthetic strategies, leading to Cr^III^–Ln^III^ molecular pairs (Ln(iii) is a trivalent lanthanide cation) beyond serendipitous co-crystallization processes.^15^ One approach involves self-assembly processes with segmental multisite ligands where labile Cr^II^ precursors are selectively recognized by didentate binding units while Ln^III^ is caught by adjacent tridentate sites. The subsequent Cr^II^ to Cr^III^ oxidation provides kinetically inert heterometallic triple-stranded Cr^III^–Ln^III^ and Cr^III^–Ln^III^–Cr^III^ helicates with remarkable photophysical properties,^16,17^ among which is the programming of the first molecular-based energy transfer light-upconversion (ETU) process.^13^

**Scheme 1:**
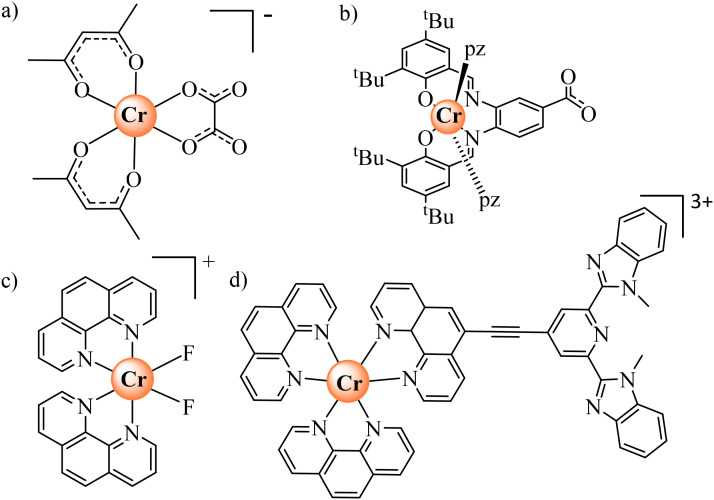
Kinetically-inert heteroleptic six-coordinate Cr^III^ complexes used as complex-as-ligand when preparing multimetallic assemblies.^12,18–21^

A more versatile synthetic strategy for incorporating open-shell [CrX_6_] chromophores as tuneable and operable sensitizers in multimetallic (supra)molecular architectures involves extending the ‘complex-as-ligand’ strategy, originally used for introducing [Cr(CN)_6_]^3−^ into multimetallic coordination polymers.^1–10,18^ Taking advantage of kinetically-controlled ligand exchange processes around inert Cr(iii), a few heteroleptic six-coordinate complexes could be prepared, in which an oxalate (Scheme 1a),^12^ a phenyl-carboxylate (Scheme 1b),^19^ a difluoride (Scheme 1c)^20^ or an extended ethyne-bis(benzimidazole)pyridine (Scheme 1d)^21^ acted as a bridging unit between the Cr(iii)-based complex-as-ligand unit and some adjacent d-block or f-block partners. However, the molecular aspects of their association processes in solution remain elusive and only solid-state crystal data support the physicochemical analyses. Considering the recent recognition that strong-field [CrN_6_] chromophores are ideal for maximizing phosphorescence quantum yields, emission lifetimes and sensitization in ‘molecular rubies’,^22–24^ there is clearly a need for the design of novel [CrN_6_] analogues working as complex-as-ligand, but using more accessible and reliable bridging units.

**Scheme 2:**
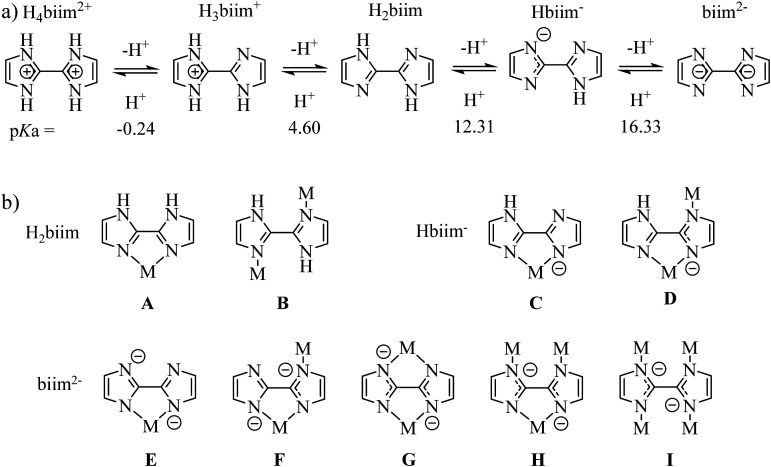
a) Successive acid–base equilibria of fully protonated 2,2′-biimidazole (H_4_biim^2+^) with p*K*_a_ values measured in DMF : H_2_O = 7 : 3 (*I* = 0.1 M).^25^ (b) Different coordination modes encountered in the literature for H_2_biim (A^26,27^ and B^28^), Hbiim^−^ (C and D)^30^ and biim^2−^ (E–I).^31–34^

**Fig. 1:**
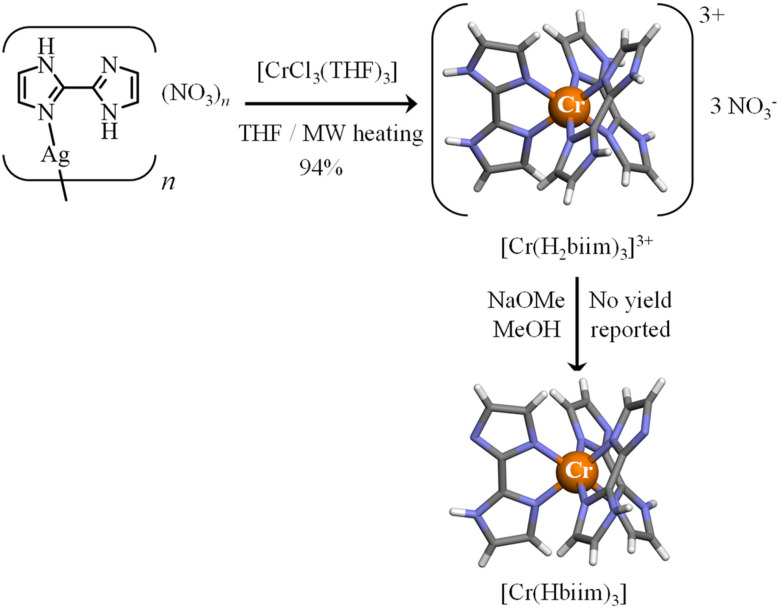
Synthesis of [Cr(H_2_biim)_3_]^3+^ (ref. 35) and [Cr(Hbiim)_3_].^36^ The molecular structures of the complexes are those found in the crystal structures of [Cr(H_2_biim)_3_](NO_3_)_3_ (CCDC-603707) and [Cr(Hbiim)_3_]·C_6_H_6_·2H_2_O (CCDC-603730).^36^ Color code: C = grey, N = blue, H = white, and Cr = orange.

With this in mind, the 2,2′-biimidazole ligand (H_2_biim, Scheme 2a)^25^ is famous for working as a versatile bridging ligand after its binding to a metallic cation (Scheme 2b). In its protonated form, it can either form hydrogen bonds for sensing anions (mode A in Scheme 2b)^26,27^ or connect other cations in a linear way (mode B in Scheme 2b).^28^ More often,^29^ multimetallic assemblies are obtained after stepwise deprotonation of the bound 2,2′-biimidazole ligand to give bridging Hbiim^−^ (modes C and D in Scheme 2b)^30^ and biim^2−^ scaffolds (modes E–I in Scheme 2b).^31–34^ Surprisingly, the studies dealing with the complexation of inert Cr^III^ centers by potentially bridging 2,2′-biimidazole are undervalued, probably due to the synthetic difficulties associated with the synthesis of these poorly soluble and pH-sensitive complexes.^35–38^ To the best of our knowledge, only the molecular structures of [Cr(H_2_biim)_3_]^3+^,^36^ [Cr(H_2_biim)_2_(Hbiim)]^2+^ (ref. 37) and [Cr(Hbiim)_3_]^36^ have been characterized in the solid state following poorly reproducible and serendipitous crystallization from intricate mixtures of ligands and metals in the presence of various amounts of counter-anions and/or solvent molecules. A recent synthetic improvement, which used anhydrous THF under microwave heating, gave [Cr(H_2_biim)_3_](NO_3_)_3_ in 94% yield (Fig. 1).^35^

Beyond the detailed descriptions of (i) sophisticated hydrogen-bonding networks in which the latter homoleptic complexes are a part and (ii) some expected axial flattening produced by didentate five-membered chelating 2,2′-biimidazole bound to Cr^III^,^36–38^ no effort has been focused on the thermodynamic deprotonation processes and the associated control of the photophysical properties. Moreover, to the best of our knowledge, no attempt to prepare heteroleptic [L_2_Cr(H_2_biim)]^3+^ has been made, while related systems with inert 4d and 5d metal ions have been designed regularly to access the two crucial p*K*_a_ values for their use as complex-as-ligand (Table S1 in the ESI†).^25,39–47^ In order to provide new perspectives for exploiting 2,2′-biimidazole as a bridging ligand between photophysically-active Cr^III^ and promising open-shell lanthanides, we report here on the molecular structures and photophysical properties of the accessible and isolable homoleptic complexes [Cr(H_2_biim)_3_]^3+^, [Cr(Hbiim)_3_] and [Cr(Hbiim)_2_(biim)]^−^ in solution and in the solid state. The second part proposes the synthesis of the unprecedented heteroleptic [(phen)_2_Cr(H_2_biim)]^3+^ complex with its detailed acid–base and photophysical properties, which become accessible in the solid state and in solution.

## Results and discussion

### Preparation and structures of the homoleptic [Cr(Me_2_biim)_3_]^3+^, [Cr(H_2_biim)_3_]^3+^, [Cr(Hbiim)_3_] and [Cr(Hbiim)_2_(biim)]^−^ complexes

**Fig. 2:**
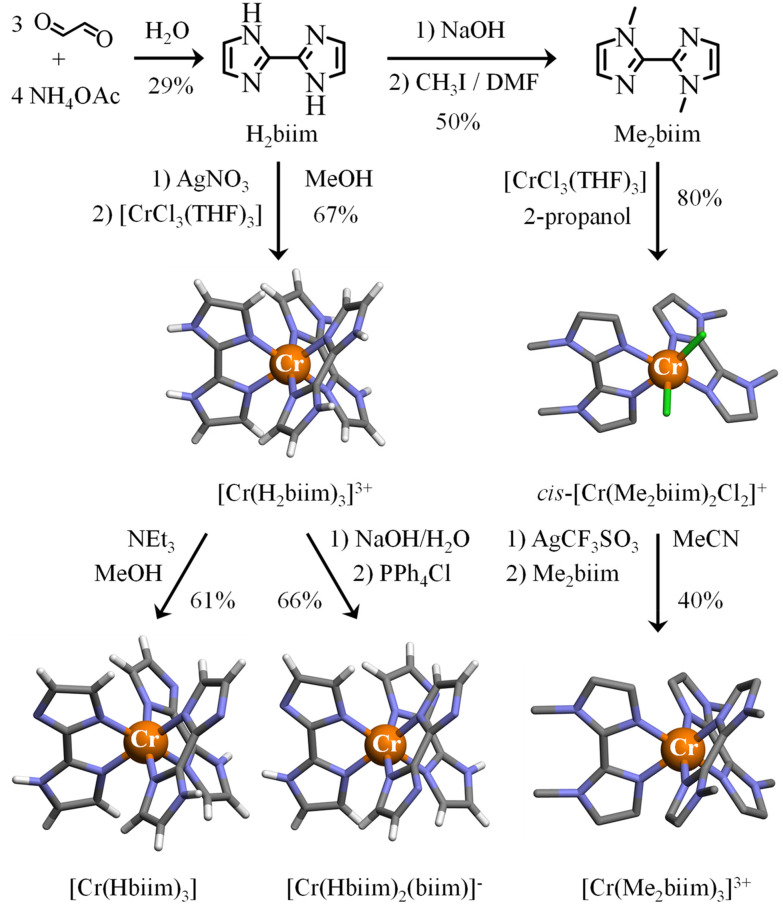
Synthesis of ligands 2,2′-biimidazole (H_2_biim) and 1,1′-dimethyl-2,2′-bi-1*H*-imidazole (Me_2_biim) and their homoleptic [Cr(H_2_biim)_3_]^3+^, [Cr(Hbiim)_3_], [Cr(Hbiim)_2_(biim)]^−^ and [Cr(Me_2_biim)_3_]^3+^ complexes. The molecular structures of the metallic complexes are those found in the associated crystal structures. Color code: C = grey, N = blue, H = white, and Cr = orange. The counter-ions and hydrogen atoms (for Me_2_biim ligands) are omitted for clarity.

2,2′-Biimidazole (H_2_biim) was synthesized from ammonium acetate and glyoxal under aqueous conditions with moderate yield.^48^ Its methyl derivative 1,1′-dimethyl-2,2′-bi-1*H*-imidazole (Me_2_biim) could be obtained by deprotonation, followed by methylation with methyl iodide (Fig. 2, top).^49^

The original literature synthesis of [Cr(H_2_biim)_3_](NO_3_)_3_ involved the reaction of CrCl_3_·3THF with [Ag(H_2_biim)]NO_3_ in MeOH^36^ or in THF^35^ (Fig. 1) because the formation of highly insoluble AgCl drove the reaction to completion. To simplify the procedure, the intermediate [Ag(H_2_biim)]NO_3_ was not isolated in this work, but was formed *in situ* by mixing AgNO_3_ and H_2_biim in MeOH. Then CrCl_3_·3THF was added into the resulting solution of [Ag(H_2_biim)]NO_3_. The purification method was identical to that used in ref. 35 and [Cr(H_2_biim)_3_](NO_3_)_3_ was isolated in good yield (Fig. 2). Recrystallization by vapor diffusion of Et_2_O into a methanolic solution provided crystals suitable for X-ray diffraction, the structural resolution of which at 120 K confirmed the crystal structure previously reported for [Cr(H_2_biim)_3_](NO_3_)_3_ at 290 K (Fig. 2, Tables S2–S3 and Fig. S1†).^36^ The synthesis of [Cr(Hbiim)_3_] was first described by Gruia *et al.*,^36^ where they used a stochiometric amount of NaOMe to deprotonate [Cr(H_2_biim)_3_]^3+^ in MeOH. However, the complete insolubility of the formed neutral [Cr(Hbiim)_3_] complex did not allow any reproducible recrystallization techniques. To overcome this problem, a methanolic solution of [Cr(H_2_biim)_3_]^3+^ was treated in this work with vapor diffusion of an excess of volatile triethylamine, the limited p*K*_a_ of which (10.74)^50^ prevented any double deprotonation of the bound Hbim ligand and finally gave crystals of [Cr(Hbiim)_3_] with good yield (61%) and in a reproducible way (Fig. 2, Tables S4–S6 and Fig. S2†). Further deprotonation of [Cr(Hbiim)_3_] could not be obtained by Gruia *et al.*,^36^ but some partial reports of analogs of [Cr(biim)_3_]^3−^, *i.e.* Ba_1.5_[Co(biim)_3_]^41^ and K_3_[Ru(biim)_3_]^51^ that were obtained using harsh basic conditions (aqueous NaOH 2 M/BaCl_2_ and ^*t*^BuOK 1.2 M in MeOH, respectively) have been noted. Consequently, [Cr(Hbiim)_3_] was dissolved in an excess of aqueous NaOH (0.5 M) until a clear yellow solution was formed. The addition of a concentrated solution of PPh_4_Cl immediately resulted in the formation of an orange precipitate. Recrystallization from MeOH by vapor diffusion of ^*t*^BuOMe provided block-shaped crystals of [Cr(Hbiim)_2_(biim)]PPh_4_(CH_3_OH) suitable for X-ray diffraction analysis in a good yield (66%) (Fig. 2, Tables S7–S9 and Fig. S3†). A detailed geometrical analysis of these homoleptic complexes (Appendix 2 in the ESI†) concludes that the [Cr(H_*x*_biim)_3_]^*n*+^ units display standard pseudo-octahedral arrangements of the six bound nitrogen donor atoms, with a compression along the pseudo-*C*_3_ axis due to the 79.6–80.4° ligand bite angles (Table A2-1 in Appendix 2†), which is characteristic of five-membered chelating polyaromatic ligands^23^ as reported for related [Cr(bipy)_3_]^3+^ (bipy = 2,2′-bipyridine, 79.1°)^52^ and [Cr(phen)_3_]^3+^ (phen = 1,10-phenanthroline, 81.0°) complexes.^53^ The Cr–N distances in [Cr(H_*x*_biim)_3_]^*n*+^ do not vary drastically (2.028–2.037 Å, compared with averages of 2.042 Å for [Cr(bipy)_3_]^3+^ and 2.051 Å for [Cr(phen)_3_]^3+^) and do not show obvious correlations with the degree of deprotonation of the bound ligand. Due to the chelation of semi-rigid didentate polyaromatic ligands, some standard trigonal distortions characterize all these complexes (Table A2-1 in Appendix 2†).

The main difference between [Cr(bipy)_3_]^3+^ and [Cr(phen)_3_]^3+^ on one hand, and the family of [Cr(H_*x*_biim)_3_]^*n*+^ complexes, is associated with the bound didentate 2,2′-biimidazole ligands which may act as N–H hydrogen-bond donors when they are protonated and as N^−^ hydrogen-bond acceptors when they are deprotonated. For the fully protonated [Cr(H_2_biim)_3_](NO_3_)_3_ complex, the weak N–H⋯O hydrogen bonds observed between the bound ligand and the nitrate counter-anion (Fig. A2-2†) are responsible for the *circa* 400 cm^−1^ decreases of the N–H stretching frequency in the vibrational spectrum with respect to the free ligand (*ν*(NH) ≈ 3000 cm^−1^ in Fig. S14 and S18-top, Table S29†). The formation of strong intermolecular N–H⋯N hydrogen bonds observed in the deprotonated complexes [Cr(Hbiim)_3_] (Fig. A2-4†) and [Cr(Hbiim)_2_(biim)]PPh_4_ (Fig. A2-6†) further weakens the N–H bond force constants and stepwise shifts the associated stretching frequency toward *ν*(NH) ≈ 2400 cm^−1^ (Fig. S15 and S18 center, Table S30†) and *ν*(NH) ≈ 2300 cm^−1^ (Fig. S16 and S18 bottom, Table S31†), respectively. In this context, the replacement of hydrogen atoms with methyl groups to give [Cr(Me_2_biim)_3_](CF_3_SO_3_)_3_*via* the Kane-Maguire synthetic strategy (Fig. 2) maintains the pseudo-octahedral structure of the [CrN_6_] core (Tables S16–S17 and Fig. S7†),^54^ but it limits intermolecular hydrogen bonds in the crystal structure (Fig. A2-10 in Appendix 2†).

The spectroscopic properties of the ligands H_2_biim and Me_2_biim and the chromium complexes [Cr(H_2_biim)_3_](NO_3_)_3_, [Cr(Hbiim)_2_(biim)]PPh_4_ and [Cr(Me_2_biim)_3_](CF_3_SO_3_)_3_ could be recorded in methanol or in acetonitrile. Unfortunately, the neutral complex [Cr(Hbiim)_3_] is not soluble enough in common organic solvents to perform reliable measurements in solution. The electrospray ionization mass spectrometry (ESI-MS) spectra (Fig. S19–S38 and Tables S33–S35†) show complicated mixtures in the gas phase containing various amounts of intact 1 : 3 complexes with variable degrees of deprotonation ([Cr(H_*x*_bim)_3_]^*n*+^) together with (i) partial ligand dissociation to give 1 : 2 complexes ([Cr(H_*x*_bim)_2_]^*n*+^ + H_2_biim) and (ii) metal reduction into Cr(ii)-based systems. All peaks could be identified from high-resolution MS spectra with a special emphasis on the detection of the ‘full’ deprotonated [Cr(Hbiim)_2_(biim)]^−^ anion (negative mode) for [Cr(Hbiim)_2_(biim)]PPh_4_ (Fig. S25 and Table S34†). It is concluded that these deprotonatable complexes suffer from the ionization process and exist as fragmented mixtures in the gas phase.

### Photophysical properties of the homoleptic [Cr(Me_2_biim)_3_]^3+^, [Cr(H_2_biim)_3_]^3+^, [Cr(Hbiim)_3_] and [Cr(Hbiim)_2_(biim)]^−^ complexes

**Fig. 3:**
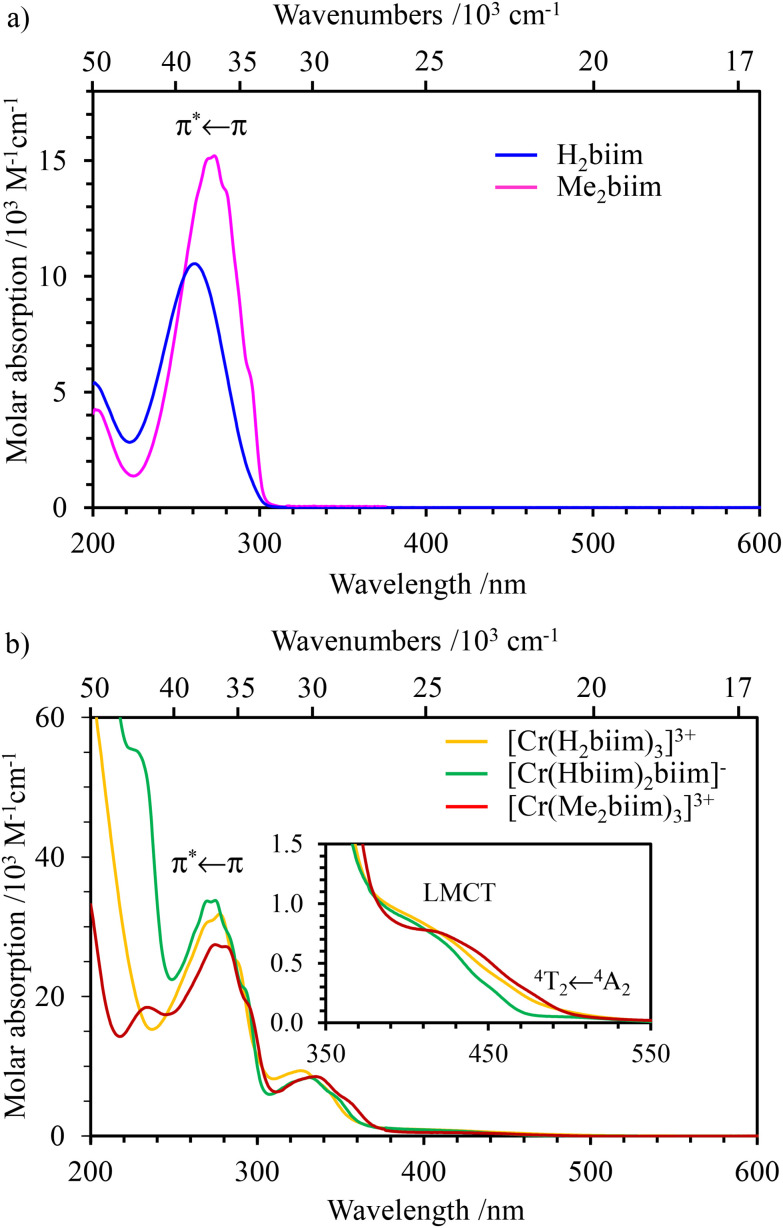
UV-visible absorption spectra of (a) non-coordinated ligands H_2_biim (purple) and Me_2_biim (dark blue) and (b) complexes [Cr(H_2_biim)_3_](NO_3_)_3_ (orange), [Cr(Hbiim)_2_(biim)]PPh_4_ (green) and [Cr(Me_2_biim)_3_](CF_3_SO_3_)_3_ (red) in MeOH at 293 K.

**Fig. 4:**
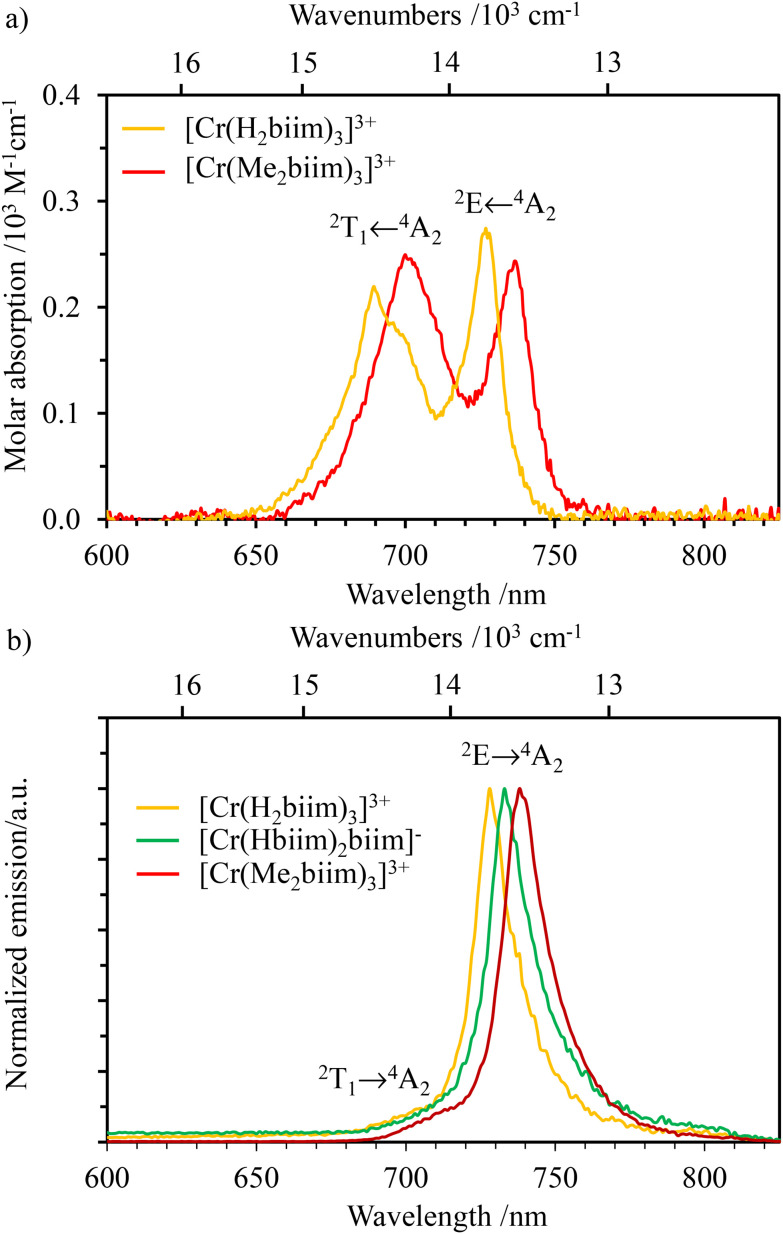
NIR (a) absorption spectra of [Cr(H_2_biim)_3_](NO_3_)_3_ (water) and [Cr(Me_2_biim)_3_](CF_3_SO_3_)_3_ (acetonitrile) and (b) emission (*λ*_exc_ = 330 nm) spectra of complexes [Cr(H_2_biim)_3_](NO_3_)_3_ (orange), [Cr(Hbiim)_2_(biim)]PPh_4_ (green) and [Cr(Me_2_biim)_3_](CF_3_SO_3_)_3_ (red) in MeOH at 293 K.

The absorption (Fig. 3 and 4a) and emission (Fig. 4b and S39†) spectra of the ligands H_2_biim and Me_2_biim and the complexes [Cr(H_2_biim)_3_](NO_3_)_3_, [Cr(Hbiim)_2_(biim)]PPh_4_ and [Cr(Me_2_biim)_3_](CF_3_SO_3_)_3_ could be recorded in MeOH. Unfortunately, the neutral complex [Cr(Hbiim)_3_] is not soluble enough to perform spectroscopy except for solid-state investigations (Fig. S40†). The UV parts of the absorption spectra are dominated by intraligand-centered π* ← π transitions (ILCT), which are split and red-shifted upon coordination to Cr^3+^ in the associated complexes. Additional LMCT (Ligand-to-Metal Charge Transfer) transitions are known to also contribute within the 330–400 nm range for these [CrN_6_] chromophores (Fig. 3).^29,55–57^ The spin-allowed, but parity forbidden ligand-field Cr(^4^T_2_ ← ^4^A_2_) transition (intensity 10 < *ε* < 100 M^−1^ cm^−1^, highlighted in Fig. 3b) can be detected as a minor contribution to the low-energy tail of the charge transfer bands.^58–63^ Careful spectral deconvolutions are required (Fig. S41–S43 and Tables S36–S39†) to safely assign *E*(^4^T_2_), *i.e.*, the energy of the Cr(^4^T_2_) excited level with respect to the ground state *E*(^4^A_2_) = 0 (Table 1, column 2).

**Table 1:** Energy of intrashell d–d transitions (in MeOH solution for *E*(^4^T_2_) and in the solid state for *E*(^2^T_1_) and *E*(^2^E)), ligand-field strength *Δ* (eqn (1)) and Racah parameters *B* (eqn (2)) and *C* (eqn (3)) for [Cr(H_2_biim)_3_](NO_3_)_3_, [Cr(Hbiim)_2_(biim)]PPh_4_, [Cr(Me_2_biim)_3_](CF_3_SO_3_)_3_, and [Cr(phen)_2_(H_2_biim)]^3+^ and for closely related pseudo-octahedral [Cr^III^N_6_] chromophores^*a*^

Complex	*E*(^4^T_2_)/cm^−1^	*E*(^2^T_1_)/cm^−1^	*E*(^2^E)/cm^−1^	*Δ*/cm^−1^	*B*/cm^−1^	*C*/cm^−1^	*E*(^4^T_2_) − *E*(^2^E)/cm^−1^	*Δ*/*B*	*C*/*B*	Ref.
[Cr(H_2_biim)_3_]^3+^	20 268	14 376	13 717	20 268	717	2845	6551	28.3	4.0	This work
[Cr(Hbiim)_3_]	—	14 263	13 670	—	—	—	—	—	—	This work
[Cr(Hbiim)_2_(biim)]^−^	19 956	14 096	13 482	19 956	686	2828	6474	29.1	4.1	This work
[Cr(Me_2_biim)_3_]^3+^	21 026	14 164	13 538	21 026	712	2779	7488	29.6	3.9	This work
[Cr(phen)_2_(H_2_biim)]^3+^	23 202	14 381	13 723	23 202	766	2697	9479	30.3	3.5	This work
[Cr(bpy)_3_]^3+^	23 400	14 450	13 800	23 400	765	2730	9600	30.6	3.6	60
[Cr(phen)_3_]^3+^	22 075	14 451	13 736	22 075	779	2700	8339	28.3	3.5	53
[Cr(tpy)_2_]^3+^	18 750	13 584	12 953	18 750	790	2512	5797	23.7	3.2	61
[Cr(ddpd)_2_]^3+^	22 990	13 550	12 903	22 990	756	2419	10 087	30.4	3.2	23
[Cr(dqp)_2_]^3+^	24 937	13 864	13 405	24 937	656	2791	11 532	38.0	4.3	24
[Cr(dpc)_2_]^+^	19 200	—	9370	19 200	470	1880	9830	40.9	4.0	62
[Cr(CN)_6_]^3−^	26 600	—	12 400	26 600	480	2800	14 200	55.4	5.8	63
[Cr(bik)_3_]^3+^	23 094	14 771	14 044	23 094	804	2737	9050	28.7	3.4	57
[Cr(bim)_3_]^3+^	21 008	14 859	14 104	21 008	781	2842	6904	26.9	3.6	57
[Cr(bie)_3_]^3+^	20 747	14 837	14 124	20 747	754	2902	6623	27.5	3.8	57

Interestingly, for pseudo-octahedral d^3^ complexes, *E*(^4^T_2_) provides a straightforward estimation of the ligand-field splitting (eqn (1)),^64,65^ which covers a narrow 19 956 ≤ *Δ* ≤ 21 026 cm^−1^ range for [Cr(H_2_biim)_3_]^3+^, [Cr(Hbiim)_2_(biim)]^−^ and [Cr(Me_2_biim)_3_]^3+^ in solution (Table 1).

1 *Δ* = *E*(^4^T_2_) − *E*(^4^A_2_)

As established for the programming of spin crossover processes while tuning the ligand-field strength,^66^ the replacement of didentate 2,2′-bipyridine (bpy) or 1,10-phenanthroline (phen), made of two connected 6-membered heterocyclic rings, with 2,2′-biimidazole (H_2_biim), made of two connected five-membered heterocyclic rings, is accompanied by an increase of the trigonal distortion in their pseudo-octahedral complexes. This is measured by a stepwise increase in the *θ* angular distortion (*θ*[Cr(phen)_3_]^3+^ = 47.5° < *θ*[Cr(bpy)_3_]^3+^ = 63.6° < *θ*[Cr(Me_2_biim)_3_]^3+^ = 80.4° computed with eqn (A2-2) and gathered in Table A2-1, see Appendix 2†), which results in a concomitant stepwise reduction of the ligand-field strength *Δ*[Cr(bpy)_3_]^3+^ ≈ *Δ*[Cr(phen)_3_]^3+^ ≈ 22 500 cm^−1^ > *Δ*[Cr(Me_2_biim)_3_]^3+^ ≈ 20 500 cm^−1^ > *Δ*[Cr(tpy)_2_]^3+^ = 18 750 cm^−1^ (Table 1).

**Fig. 5:**
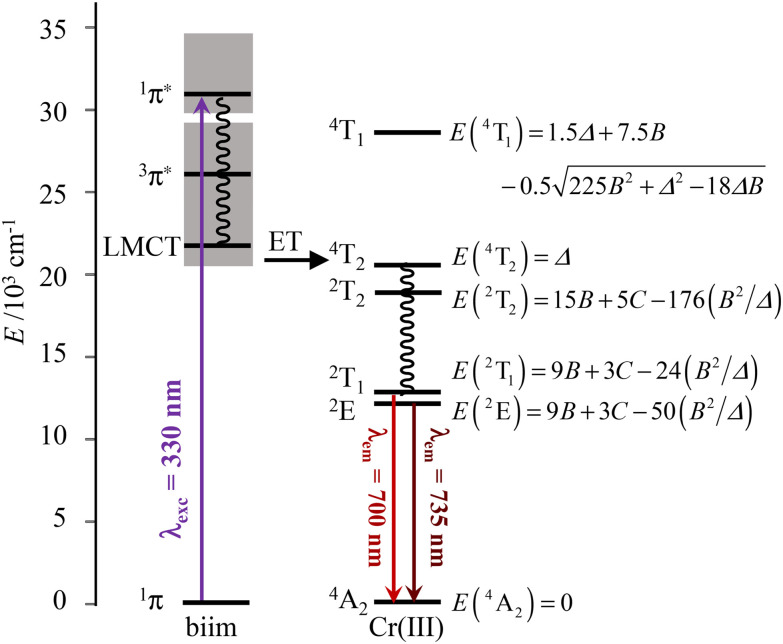
Jablonski diagram of homoleptic [Cr(H_2_biim)_3_]^3+^ or [Cr(Me_2_biim)_3_]^3+^ complexes showing the antenna effect upon UV excitation at 330 nm. The modeling of the energies of the Cr levels is taken from ref. 64.

The interelectronic repulsion is estimated using the Racah parameters *B* (eqn (2)) and *C* (eqn (3)), which requires the energy of the lowest doublet levels *E*(^2^E) and *E*(^2^T_1_) to be accessible (Fig. 5).^64,65^2
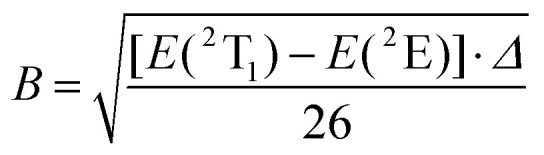
3
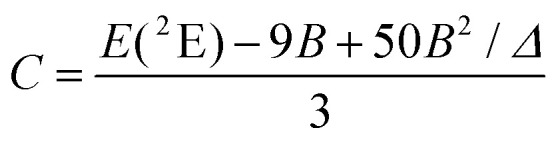


Assuming an *O*_h_ symmetry, the ground state absorption bands Cr(^2^E ← ^4^A_2_) and Cr(^2^T_1_ ← ^4^A_2_) have very weak intensities (0.05 < *ε* < 1 M^−1^ cm^−1^) due to the breaking of both parity and spin conservation rules. These transitions could be detected in the NIR domain of the solid-state absorption spectra of the four investigated complexes (Fig. S40†), while related solution data could be recorded only for the most soluble [Cr(H_2_biim)_3_](NO_3_)_3_ (*c* ≥ 10^−2^ M in water) and [Cr(Me_2_biim)_3_](CF_3_SO_3_)_3_ (*c* ≥ 10^−2^ M in acetonitrile) complexes (Fig. 4a). Introducing *E*(^4^T_2_), *E*(^2^E) and *E*(^2^T_1_) into eqn (1)–(3) provides the Racah parameters *B* and *C* collected in Table 1. Interestingly, 686 ≤ *B* ≤ 717 cm^−1^ found for [Cr(H_2_biim)_3_](NO_3_)_3_, [Cr(Hbiim)_2_(biim)]PPh_4_ and [Cr(Me_2_biim)_3_](CF_3_SO_3_)_3_ points to a global increase of the nephelauxetic effect when didentate 2,2′-bipyridine or 1,10-phenanthroline type ligands bound to Cr^3+^ (765 ≤ *B* ≤ 790 cm^−1^) are replaced with 2,2′-biimidazole type ligands.

For absorption spectra recorded in solution (Fig. 4a), it is possible to calculate the radiative rate constant of the emissive levels ^2^E and ^2^T_1_ using the Strickler–Berg eqn (4) ^67,68^, which is derived from Einstein's relationship for spontaneous emission (Table 2, column 2 and Table S40†).^69^4
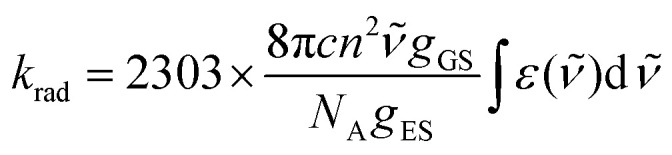


**Table 2:** Experimental Cr(^2^E) lifetimes (*τ*) and radiative (*k*_rad_) and non-radiative (*k*_non-rad_) relaxation rate constants and intrinsic quantum yields (*ϕ*^intrinsic^_complex_ = *k*_rad_/(*k*_rad_ + *k*_non-rad_)) of chromium complexes

Complex	*k*_rad_/s^−1^	*τ* ^293 K, Ar^ a/μs	*k* ^293 K, Ar^_non-rad_ a/10^3^ s^−1^	*ϕ* ^intrinsic^_complex_ 293 K, Ar	*τ* ^293 K, air^ b/μs	*k* ^293 K, air^_non-rad_ b/10^3^ s^−1^	*ϕ* ^intrinsic^_complex_ 293 K, air	*τ* ^77 K^ c/μs	*k* ^77 K^_non-rad_/s^−1^	*ϕ* ^intrinsic^_complex_ 77 K	Ref.
[Cr(H_2_biim)_3_]^3+^	75(4)	0.47(2)^d^	2120(106)	3(1) × 10^−5^	0.44(2)^d^	2260(113)	3(1) × 10^−5^	2950(150)^e^	263(13)	2.0(6) × 10^−1^	This work
[Cr(Hbiim)_2_(biim)]^−^	—	0.178(9)^d^	—	—	0.141(7)^d^	—	—	2780(140)^e^	—	—	This work
[Cr(Me_2_biim)_3_]^3+^	73(4)	4.28(4)^d^	234(12)	3(1) × 10^−4^	3.31(9)^d^	302(1)	2.4(8) × 10^−4^	3005(150)^e^	260(13)	2.2(7) × 10^−1^	This work
[Cr(phen)_2_(H_2_biim)]^3+^	65(3)	38(1)^f^	26(7)	2.5(8) × 10^−3^	14(1)^f^	71(7)	9(3) × 10^−4^	2940(140)^g^		1.9(6) × 10^−1^	This work
[Cr(bpy)_3_]^3+^	182	74^h^	13.3	1.4(4) × 10^−2^	52^h^	19	9(3) × 10^−3^	5000^i^	18	9(3) × 10^−1^	54, 58 and 70–72
		39^d^	25.5	7(2) × 10^−3^	29^d^	34	5(2) × 10^−3^	6500^j^	≈0	≈1	
[Cr(phen)_3_]^3+^	319	356^h^	2.49	1.1(4) × 10^−1^	74^h^	13	2.4(7) × 10^−2^	2100^g^	157	6.7(2) × 10^−1^	53, 54, 58 and 70–73
		34^d^	29.1	1.1(3) × 10^−2^	22^d^	45	7(2) × 10^−3^	5300^j^	≈0	≈1	
		224^f^	4.15	7(2) × 10^−2^	37^f^	27	1.2(3) × 10^−2^				
		270^k^	3.38	9(3) × 10^−2^							
[Cr(tpy)_2_]^3+^	—	—	—	—	0.14^f^	—	—	540^i^	—	—	61, 74 and 75
								670^g^	—		
[Cr(dqp)_2_]^3+^	30	1270^h^	0.76	4(1) × 10^−2^	83^h^	12	2.5(8) × 10^−3^	3070^i^	296	9(3) × 10^−2^	24
		2140^f^	0.44	6(2) × 10^−2^	31^f^	32	9(3) × 10^−4^		296	9(3) × 10^−2^	
[Cr(CN)_6_]^3−^	97	—	—	—	0.12^i^	8330	1.2(4) × 10^−5^	3950^i^	156	4(1) × 10^−1^	72
[Cr(bik)_3_]^3+^	31	209^f^	4.75	7(2) × 10^−3^	128^f^	7.8	4(1) × 10^−3^	8220^g^	91	2.6(8) × 10^−1^	57
[Cr(bim)_3_]^3+^	37	131^f^	7.60	5(2) × 10^−3^	73^f^	14	2.7(8) × 10^−3^	6750^g^	111	2.5(8) × 10^−1^	57
[Cr(bie)_3_]^3+^	60	7^f^	143	4(1) × 10^−4^	8^f^	125	5(2) × 10^−4^	5250^g^	130	3(1) × 10^−1^	57

a Recorded in degassed solution at room temperature.

b Recorded in air-equilibrated solution at room temperature.

c Recorded in a frozen solvent mixture at 77 K.

d In MeOH.

e In MeOH/H_2_O.

f In CH_3_CN.

g In CH_3_CN/C_2_H_5_CN.

h In H_2_O.

i In H_2_O/DMSO.

j In aq. HClO_4_/MeOH.

k In aq. HCl.

Here *c* is the speed of light in vacuum (cm s^−1^), *n* is the refractive index of the solvent, *N*_A_ is the Avogadro number (mol^−1^), *g*_GS_ is the degeneracy of the ground state (*g*(^4^A_2_) = 4), *g*_ES_ is the degeneracy of the excited state (*g*(^2^T_1_) = 6 and *g*(^2^E) = 4), *ν̃* is the barycenter of the transition in the wavenumber (cm^−1^) and 
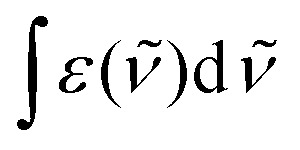
 is the area under the absorption spectrum of each transition (M^−1^ cm^−2^, Fig. S44 and S45†). The radiative rate constants of the emissive Cr(^2^E) excited level estimated for [Cr(H_2_biim)_3_]^3+^ (*k*_rad_ = 75(4) s^−1^) and [Cr(Me_2_biim)_3_] (*k*_rad_ = 73(4) s^−1^) are smaller than those reported for [Cr(bpy)_3_]^3+^ (*k*_rad_ = 182 s^−1^) and [Cr(phen)_3_]^3+^ (*k*_rad_ = 319 s^−1^, Table 2),^58,69–73^ but fall within the expected range for pseudo-octahedral [CrN_6_] chromophores (Table S40†).^70–77^ Upon room-temperature ligand-based excitation at 330 nm in solution, the complexes [Cr(H_2_biim)_3_](NO_3_)_3_, [Cr(Hbiim)_2_(biim)]PPh_4_ and [Cr(Me_2_biim)_3_](CF_3_SO_3_)_3_ show the expected downshifted NIR spin–flip Cr(^2^E → ^4^A_2_) phosphorescence at 730–740 nm together with weak shoulders corresponding to Cr(^2^T_1_ → ^4^A_2_) within the 700–710 nm domain (Fig. 4b and 5). The latter dual emission disappears at 77 K (Fig. S39†) as a result of the depopulation of the high-energy Cr(^2^T_1_) level, and a single band Cr(^2^E → ^4^A_2_) contributes to phosphorescence. Very similar results are observed in the solid state upon 330 nm excitation for the four complexes [Cr(H_2_biim)_3_](NO_3_)_3_, [Cr(Hbiim)_3_], [Cr(Hbiim)_2_(biim)]PPh_4_ and [Cr(Me_2_biim)_3_](CF_3_SO_3_)_3_ (Fig. S40b†), which ultimately demonstrate (i) the expected negligible Stokes shifts affecting the spin–flip Cr(^2^T_1_,^2^E ↔ ^4^A_2_) transitions (Fig. S46†) and (ii) the efficient sensitization of the spin–flip phosphorescence by all the accessible ligand-based excited states (Fig. S47†).

Upon pulsed laser excitation at 355 nm at 77 K in frozen MeOH/H_2_O solutions, the characteristic lifetime of the emissive Cr(^2^E) levels for [Cr(H_2_biim)_3_](NO_3_)_3_ (*τ*^77 K^ = 3.0(1) ms), [Cr(Hbiim)_2_(biim)]PPh_4_ (*τ*^77 K^ = 2.7(1) ms) and [Cr(Me_2_biim)_3_](CF_3_SO_3_)_3_ (*τ*^77 K^ = 3.0(1) ms) tends toward the radiative lifetime *τ*_rad_ = 13.4(7) ms, in agreement with minor non-radiative vibrational quenching constants at this temperature for rigid triple-helical units, as similarly reported for [Cr(bpy)_3_]^3+^ and [Cr(phen)_3_]^3+^ (Table 2 column 8). At room temperature, the Cr(^2^E) lifetimes drastically drop below the microsecond range for [Cr(H_2_biim)_3_](NO_3_)_3_ and [Cr(Hbiim)_2_(biim)]PPh_4_, which possess high-energy N–H oscillators, while the decrease of the lifetime for the methylated analogue [Cr(Me_2_biim)_3_](CF_3_SO_3_)_3_ (*τ*^293 K, Ar^ = 4.28(4) μs) is less dramatic and mirrors those reported for [Cr(bpy)_3_]^3+^ and [Cr(phen)_3_]^3+^ (Table 2 and Fig. S48–S56†). Consequently, the non-radiative rate constant *k*^293 K, Ar^_non-rad_ = 1/*τ*^293 K, Ar^ − *k*_rad_ = 2.1(1) × 10^6^ s^−1^ measured for [Cr(H_2_biim)_3_](NO_3_)_3_ in MeOH corresponds to the largest vibrational quenching process along the series of [Cr(N^∩^N)_3_]^3+^ chromophores collected in Table 2. Finally, the presence of ^3^O_2_ in solution has only a minor effect on the lifetime, indicating that quenching by oxygen is not a major contributor to energy relaxation in these systems (*τ*^293 K, air^ in Table 2, column 6).

### Acid–base properties of homoleptic [Cr(H_2_biim)_3_]^3+^, [Cr(Hbiim)_3_] and [Cr(Hbiim)_2_(biim)]^−^ complexes

**Fig. 6:**
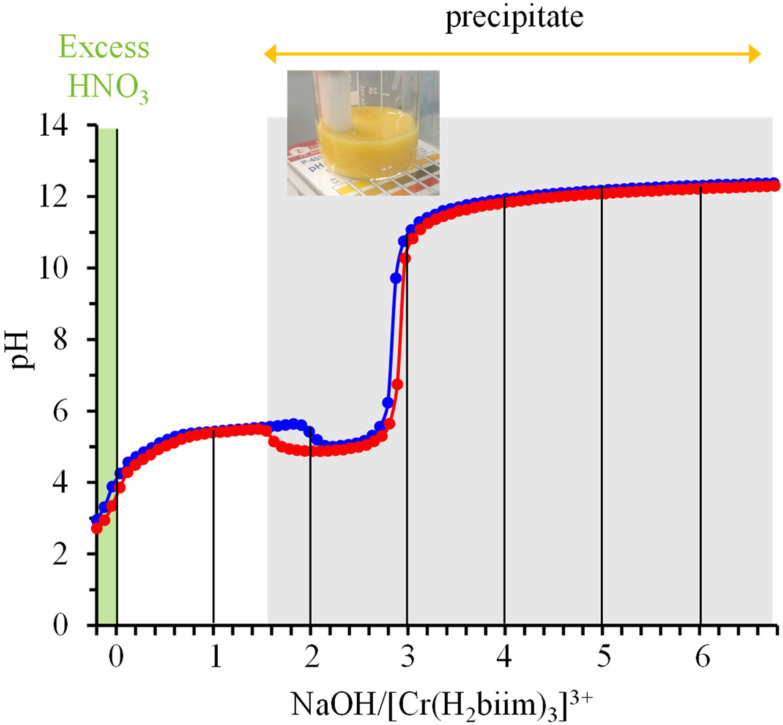
Reproducible acid–base titrations (blue trace for titration 1 and red trace for titration 2) of a solution of [Cr(H_2_biim)_3_](NO_3_)_3_ + 0.2 eq. HNO_3_ with NaOH (water, 293 K) highlighting the formation of an insoluble yellow precipitate.

In order to extract the acid–base thermodynamic constants connecting [Cr(H_2_biim)_3_]^3+^ with its successive deprotonated forms [Cr(H_2_biim)_2_(Hbiim)]^2+^ (*K*_a1_), [Cr(H_2_biim)(Hbiim)_2_]^+^ (*K*_a2_), [Cr(Hbiim)_3_] (*K*_a3_) and [Cr(Hbiim)_2_(biim)]^−^ (*K*_a4_), we performed two successive pH-metric titrations of aqueous solutions of [Cr(H_2_biim)_3_](NO_3_)_3_ using NaOH as the titrant. An initial addition of 0.2 equivalents of HNO_3_ ensured that the complex exists initially in its fully protonated form [Cr(H_2_biim)_3_]^3+^ (Fig. 6). The pH curve profile is different from the standard pH-metric titration expected for a weak polyacid with a strong base due to the concomitant precipitation of insoluble [Cr(Hbiim)_3_] (Fig. 6). It is therefore not possible to extract the searched p*K*_a_ values and one can only estimate qualitatively p*K*_a1_, p*K*_a2_ < 7. Moreover, the preservation of [Cr(Hbiim)_3_] as a precipitate in the presence of a large excess of base indicates its negligible deprotonation for pH ≤ 12 in water and p*K*_a4_ > 12.

### Preparation and structures of the heteroleptic [Cr(phen)_2_(H_*x*_biim)]^(1+*x*)+^ (*x* = 2–0) complexes

**Fig. 7:**
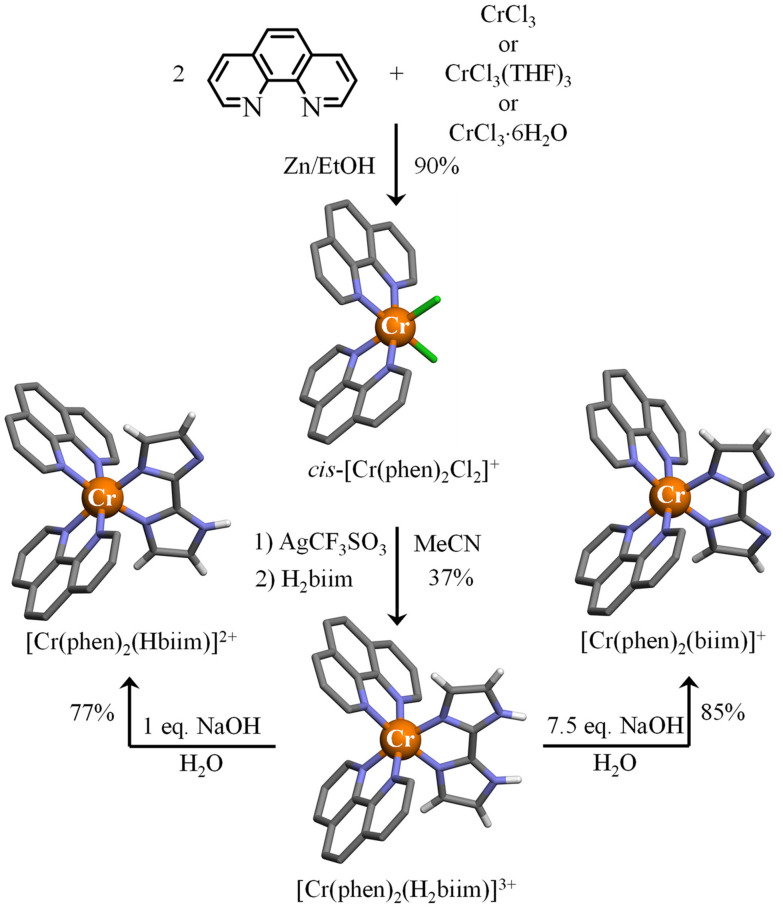
Synthesis of the heteroleptic *cis*-[Cr(phen)_2_Cl_2_]^+^ (CCDC-1865022),^53^ [Cr(phen)_2_(H_2_biim)]^3+^, [Cr(phen)_2_(Hbiim)]^2+^ and [Cr(phen)_2_(biim)]^+^ complexes. The molecular structures of the metallic complexes are those found in the associated crystal structures. Color code: C = grey, N = blue, H = white, and Cr = orange. The counter-ions and hydrogen atoms (for 1,10-phenanthroline ligands) are omitted for clarity.

In order to limit the number of bound deprotonatable ligands and the concomitant formation of insoluble neutral complexes, we followed the Kane-Maguire synthetic strategy for preparing inert heteroleptic [Cr(phen)_2_(H_2_biim)]^3+^ (Fig. 7).^54^ Taking advantage of the *trans* influence,^78^ the complexation of 2.0 eq. of 1,10-phenanthroline (phen) to CrCl_3_ yields almost quantitatively *cis*-[Cr(phen)_2_Cl_2_]^+^ under reducing conditions for catalyzing Cr(iii)/Cr(ii) exchange processes and ligand-exchange dynamics.^53^ The replacement of inert Cr–Cl bonds with labile Cr–OSO_2_CF_3_ bonds^79–81^ was performed under soft conditions by using Ag(O_3_SCF_3_) instead of an excess of triflic acid.^24^ The addition of H_2_biim finally provided [Cr(phen)_2_(H_2_biim)](CF_3_SO_3_)_3_ in moderate yield (37%, Fig. 7). Subsequent deprotonation with aqueous NaOH under stoichiometric conditions gave [Cr(phen)_2_(Hbiim)](CF_3_SO_3_)_2_, whereas the use of an excess of base yielded the doubly deprotonated complex [Cr(phen)_2_(biim)](CF_3_SO_3_). X-ray quality monocrystals could be grown by slow diffusion of diethyl ether into solutions of the complexes either in acetonitrile to give [Cr(phen)_2_(H_2_biim)](CF_3_SO_3_)_3_(H_2_O)_0.25_ (Fig. 7 and Tables S18–S20†) or in methanol to provide [Cr(phen)_2_(biim)](CF_3_SO_3_)(CH_3_OH)_1.5_ (Fig. 7 and Tables S24–S26†). X-ray quality prisms of [Cr(phen)_2_(Hbiim)](CF_3_SO_3_)_2_(H_2_O)_0.5_ (Fig. 7 and Tables S21–S23†) were isolated from a cooled (4 °C) aqueous solution.

The molecular structures of the three [Cr(phen)_2_(H_*x*_biim)]^(1+*x*)+^ cations (*x* = 2–0) display pseudo-octahedral [CrN_6_] chromophores with bond lengths and trigonal distortions typical of chromium complexes bound by three didentate 5-membered chelate polyaromatic ligands (Appendix 3†), similar to the discussion in the previous section for the homoleptic analogues [Cr(H_2_biim)_3_]^3+^, [Cr(Hbiim)_3_] and [Cr(Hbiim)_2_(biim)]^−^ (see Appendix 2†). For the heteroleptic complexes, one notices that the Cr–N bond lengths are shorter for the bound biimidazole ligands compared to those of the bound phen ligands (*d*_Cr–N(biim)_ < *d*_Cr–N(phen)_, Table 3). The contraction of the *d*_Cr–N(biim)_ bond lengths upon stepwise deprotonation can be assigned to the increased basicity of the bound biimidazole ligand. The compensating longer *d*_Cr–N(phen)_ bond lengths result from the reduced charge borne by the central chromium metal. In terms of intermolecular hydrogen bonds, the bound protonated H_2_biim ligand in [Cr(phen)_2_(H_2_biim)](CF_3_SO_3_)_3_(H_2_O)_0.25_ acts as a NH donor for acceptor oxygen atoms of triflate counter-anions and interstitial water molecules. For the mono-deprotonated bound Hbiim^−^ ligand in [Cr(phen)_2_(Hbiim)](CF_3_SO_3_)_2_(H_2_O)_0.5_, intermolecular hydrogen bonds between two adjacent complexes through N–H⋯N bonds are observed (Fig. A3-3†), as previously described for [Cr(Hbiim)_3_] (Fig. A2-4†). Finally, the totally deprotonated bound biim^2−^ ligand in [Cr(phen)_2_(biim)](CF_3_SO_3_)(CH_3_OH)_1.5_ is not involved in hydrogen bonding.

**Table 3:** Comparison of the average Cr–N bond lengths (*d̄*_Cr–N_)^a^ of complexes [Cr(phen)_2_(H_2_biim)](CF_3_SO_3_)_3_(H_2_O)_0.25_, [Cr(phen)_2_(Hbiim)](CF_3_SO_3_)_2_(H_2_O)_0.5_ and [Cr(phen)_2_(biim)](CF_3_SO_3_)(CH_3_OH)_1.5_ in their crystal structures

Complex	*d̄*_Cr–N_/Å	*d̄*_Cr–N(phen)_/Å	*d̄*_Cr–N(biim)_/Å
[Cr(phen)_2_(H_2_biim)](CF_3_SO_3_)_3_	2.04(2)	2.054(3)	2.024(7)
[Cr(phen)_2_(Hbiim)](CF_3_SO_3_)_2_	2.05(2)	2.062(6)	2.018(0)
[Cr(phen)_2_(biim)]CF_3_SO_3_	2.05(4)	2.07(1)	1.997(5)

a The standard deviations refer to deviations from the computed averages.

In the IR spectra of the heteroleptic complexes, the O–H stretching vibrations of co-crystallized water or methanol molecules involved in hydrogen bonding (3500 ≤ *ν*_OH_ ≤ 2500 cm^−1^) hinder a straightforward interpretation of N–H stretching bands associated with the bound H_*x*_biim ligands (*x* = 2–0; Fig. S57†). On the other hand, and as previously mentioned for the related homoleptic complexes, the ESI-MS spectra recorded in acetonitrile do not vary significantly with the degree of protonation of the bound 2,2′-biimidazole ligands for the different [Cr(phen)_2_(H_*x*_biim)]^(1+*x*)+^ cations (*x* = 2–0, Fig. S58†). High-resolution ESI-MS analyses confirm the formation of [Cr(phen)_2_(Hbiim)]^2+^ as the major gas-phase cation, regardless of the degree of protonation of the bound 2,2′-biimidazole ligand in the selected complex (Table S41 and Fig. S58–62†).

### Acid–base properties of the heteroleptic [Cr(phen)_2_(H_2_biim)]^3+^ complex

**Fig. 8:**
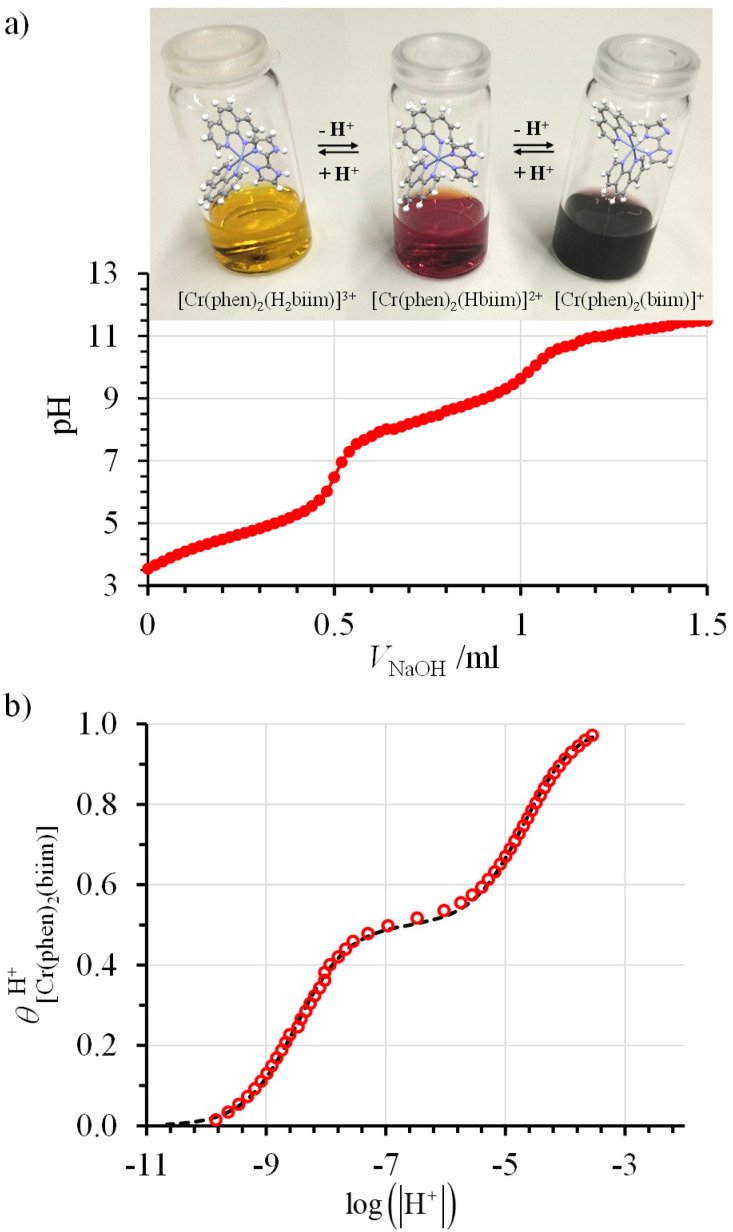
(a) Titration of 51 mg (52 μmol) of [Cr(phen)_2_(H_2_biim)](CF_3_SO_3_)_3_ (10 mL aqueous KNO_3_ 0.1 M, *c* = 5.2 mmol L^−1^) with NaOH 0.1 N highlighting the color changes and (b) associated binding isotherm depicted as plots of experimental (red circles, eqn (5)) and fitted (dashed black trace, eqn (8)) occupancy factors as a function of log(|H^+^|).

The successive deprotonation of bound 2,2′-biimidazole in [Cr(phen)_2_(H_2_biim)]^3+^ has been quantitatively studied by pH metric titrations in water at fixed ionic strength (0.1 M KNO_3_, Fig. 8a). The two successive pH jumps (5 ≤ pH ≤ 8 and 9 ≤ pH ≤ 11) are accompanied by concomitant abrupt changes in colors from yellow ([Cr(phen)_2_(H_2_biim)]^3+^) to dark orange ([Cr(phen)_2_(Hbiim)]^2+^) and finally to dark brown ([Cr(phen)_2_(biim)]^+^, Fig. 8a). The associated occupancy factors 
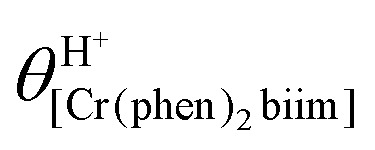
 experimentally obtained with eqn (5) (red circles in Fig. 8b) display a two-step binding isotherm typical of the anti-cooperative successive fixation of two protons according to equilibria (6)–(7) and modeled with eqn (8). The best fits for the two successive deprotonation steps of [Cr(phen)_2_(H_2_biim)]^3+^ (black trace in Fig. 8b) correspond to p*K*_a1_ = 4.67(3) and p*K*_a2_ = 8.59(11), which are approximately eight orders of magnitude more acidic than those measured for free 2,2′-biimidazole (p*K*_a1_ = 12.31 and p*K*_a2_ = 16.33),^25^5
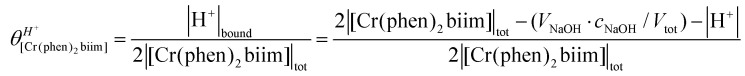
6
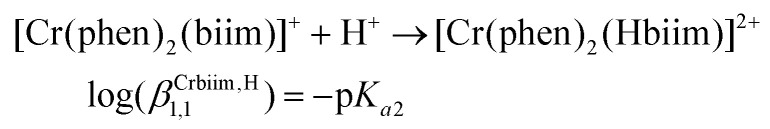
7
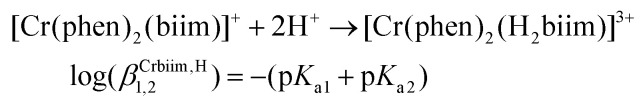
8
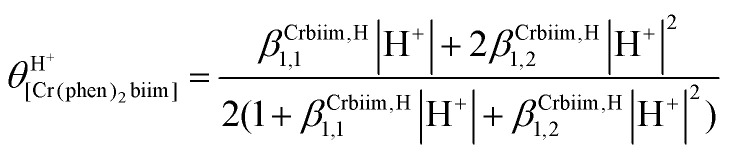


**Fig. 9:**
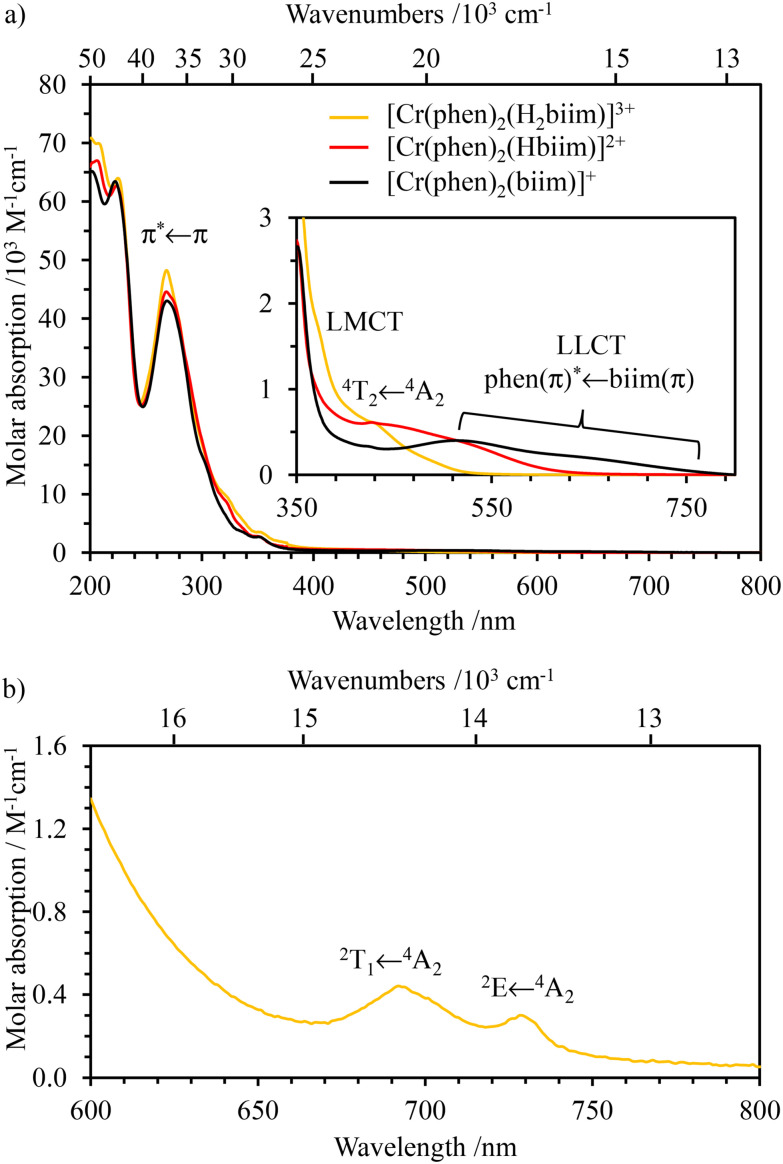
Absorption spectra of (a) [Cr(phen)_2_(H_2_biim)]^3+^ (orange trace), [Cr(phen)_2_(Hbiim)]^2+^ (red trace) and [Cr(phen)_2_(biim)]^+^ (black trace) in CH_3_CN (*c* ≈ 10^−5^ mol L^−1^) and (b) [Cr(phen)_2_(H_2_biim)]^3+^ in CH_3_CN (*c* ≈ 10^−2^ mol L^−1^). LMCT = ligand to metal charge transfer and LLCT = ligand to ligand charge transfer.

Compared with [Co^III^(en)_2_(H_2_biim)]^3+^ (p*K*_a1_ = 5.5 and p*K*_a2_ = 9.9; ionic radius = 0.545 Å),^41^ the one order of magnitude lower p*K*_a_ measured for [Cr^III^(phen)_2_(H_2_biim)]^3+^ (ionic radius = 0.615 Å) suggests that the {Cr^III^(phen)_2_}^3+^ scaffold (with respect to {Co^III^(en)_2_}^3+^) better stabilizes the negative charges brought by the bound deprotonated 2,2′-biimidazole ligand. Consequently, upon successive deprotonation, one can reasonably predict the appearance of low energy (phen)π* ← (H_*x*_biim)π (*x* = 2–0) intramolecular ligand-to-ligand charge transfer (LLCT) bands in the absorption spectra of [Cr^III^(phen)_2_(H_*x*_biim)]^(1+*x*)+^ upon deprotonation. These transitions are confirmed by TD-DFT calculations (Fig. A4-2 to A4-10 and Tables A4-6 to A4-8 in Appendix 4†) and indeed observed in solution (Fig. 9).

Finally, taking advantage of the pH-dependence of the absorption spectra, the determination of the p*K*_a_ values in water at ionic strength close to zero (*I* ≈ 0) could be carried out with the help of spectrophotometry to give p*K*_a1_ = 3.9(1) and p*K*_a2_ = 7.8(1) (Fig. S63†), which are close to those determined at *I* = 0.1 M (KNO_3_).

### Photophysical properties of the heteroleptic [Cr(phen)_2_(H_*x*_biim)]^(1+*x*)+^ (*x* = 2–0) complexes

The three heteroleptic complexes exhibit similar and intense absorption bands in the UV range (below 350 nm, Fig. 9a), which can be assigned to intraligand π–π* transitions (ILCT) completed by variable amounts of interligand (LLCT), ligand to-metal (LMCT) and metal-to-ligand (MLCT) charge transfer bands (see Appendix 4† for TD-DFT calculations). The spectra differ in the visible domain (350–750 nm, highlighted in Fig. 9a) mainly due to a shift of the interligand (phen)π* ← (H_*x*_biim)π (*x* = 0–2, LLCT) transitions toward lower energies upon successive deprotonations (Fig. S64 and Fig. A4-4, A4-7 and A4-9†). This shift is responsible for the color change accompanying the stepwise deprotonation of [Cr(phen)_2_(H_2_biim)]^3+^ (Fig. 8a). According to theoretical TD-DFT calculations (Table 1, entries 1–3) and CASSCF(7,12)/FIC-NEVPT2 (Table A4-2†) and in line with the trend observed in the homoleptic complexes, the ligand-field strength *Δ*, reminiscent of Cr(^4^T_2_ ← ^4^A_2_) transitions in octahedral geometry, also decreases with stepwise deprotonation: [Cr(phen)_2_(H_2_biim)]^3+^ (412.9 nm, *Δ* ≈ 24 219 cm^−1^) > [Cr(phen)_2_(Hbiim)]^2+^ (434 nm, *Δ* ≈ 23 042 cm^−1^) > [Cr(phen)_2_(biim)]^+^ (457.8 nm, *Δ* ≈ 21 844 cm^−1^). This reduction in the ligand-field strength is accompanied by (i) a decrease of the computed total spin density at the chromium center (Fig. A4-1†) which reflects the reduced total positive charge borne by this atom and (ii) a negligible change in the estimated Racah parameters *B* and *C* (Table A4-1†). One can thus predict that the stronger interactions with the deprotonated 2,2-biimidazole ligand (*d̄*_Cr–N(biim)_ is becoming shorter) in [Cr(phen)_2_(H_*x*_biim)]^(1+*x*)+^ (*x* = 2–0) are more than compensated by the removal of the bound 1,10-phenanthroline ligands (*d̄*_Cr–N(phen)_ is becoming longer) as exemplified in the molecular structures in the crystalline state (push–pull effect, Table 3).

The NIR absorption spectrum of [Cr(phen)_2_(H_2_biim)]^3+^ (Fig. 9b) exhibits two well-resolved absorption bands that are assigned to the spin–flip transitions Cr(^2^E ← ^4^A_2_) and Cr(^2^T_1_ ← ^4^A_2_) assuming *O*_h_ symmetry and with *ε* = 0.27 and 0.19 M^−1^ cm^−1^, respectively. For the deprotonated derivatives [Cr(phen)_2_(Hbiim)]^2+^ and [Cr(phen)_2_(biim)]^+^, the larger residual interligand charge transfer bands mask these weak forbidden spin–flip transitions (Fig. S65†). Consequently, Racah parameters *B* = 766 cm^−1^ and *C* = 2697 cm^−1^ (eqn (2) and (3), Table 1, entry 4) together with the radiative rate constant of *k*_rad_ = 65(3) s^−1^ (eqn (4), and Table 2, column 1), typical of [CrN_6_] chromophores, could be estimated only for [Cr(phen)_2_(H_2_biim)]^3+^.

**Fig. 10:**
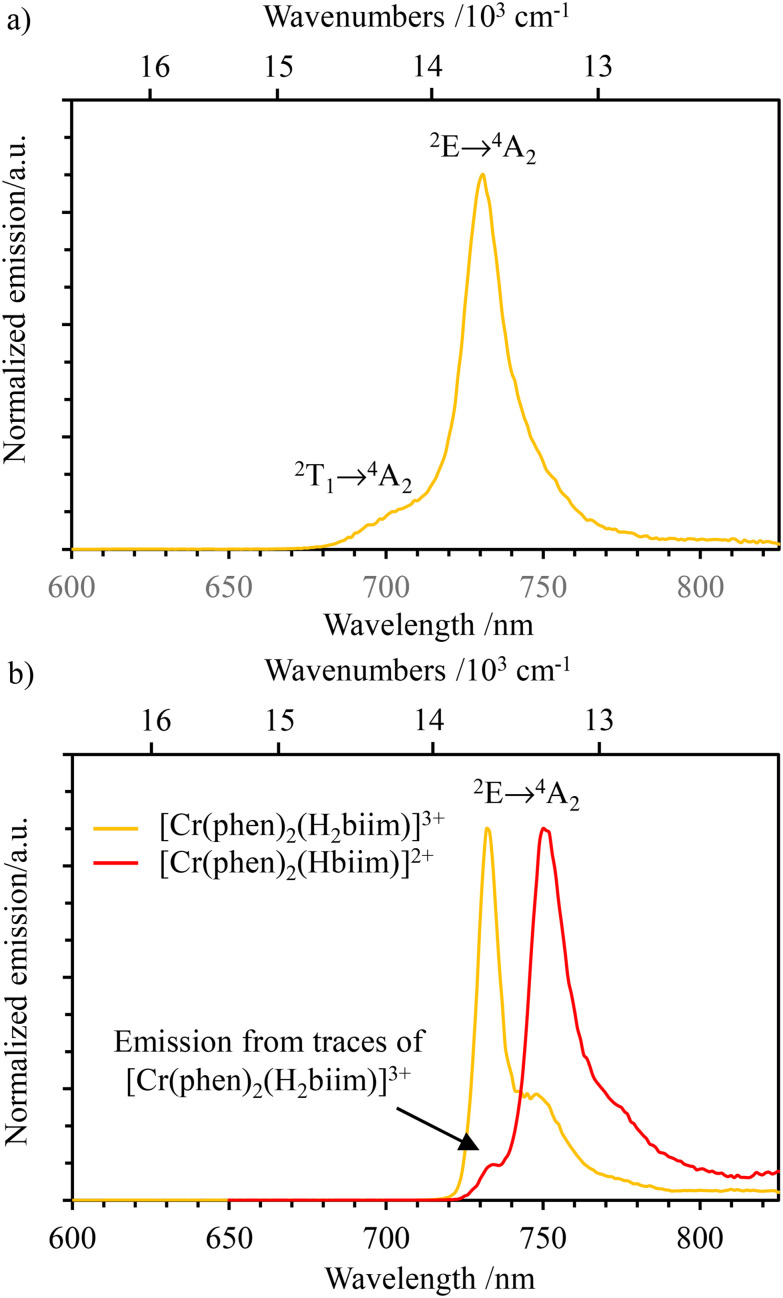
Emission spectra (*λ*_exc_ = 350 nm) of (a) [Cr(phen)_2_(H_2_biim)]^3+^ at 293 K in CH_3_CN and (b) [Cr(phen)_2_(H_2_biim)]^3+^ (orange trace) and [Cr(phen)_2_(Hbiim)]^2+^ (red trace) at 77 K in frozen CH_3_CN/C_2_H_5_CN (6 : 4).

At room temperature in CH_3_CN, only [Cr(phen)_2_(H_2_biim)]^3+^ is emissive and shows the typical dual Cr(^2^T_1_ → ^4^A_2_) and Cr(^2^E → ^4^A_2_) emission observed for many Cr(iii) complexes (Fig. 10a). In addition to [Cr(phen)_2_(H_2_biim)]^3+^, the NIR emission of the complex [Cr(phen)_2_(Hbiim)]^2+^ can be detected at low temperature in frozen solvent mixtures (CH_3_CN/C_2_H_5_CN 6 : 4 at 77 K, Fig. 10b), whereas [Cr(phen)_2_(biim)]^+^ remains non-emissive. Compared with the emission band of [Cr(phen)_2_(H_2_biim)]^3+^ (*λ*_max_ = 732 nm; *ν̃* = 13 661 cm^−1^), the first deprotonated analog [Cr(phen)_2_(Hbiim)]^2+^ shows a red-shifted Cr(^2^E → ^4^A_2_) transition at *λ*_max_ = 750 nm; *ν̃* = 13 333 cm^−1^ (Fig. 10b). Additionally, the spectrum of [Cr(phen)_2_(Hbiim)]^2+^ contains a band foot at 733 nm originating from a small amount of [Cr(phen)_2_(H_2_biim)]^3+^, which is inevitably present in solution due to the proton-transfer equilibrium (9):9
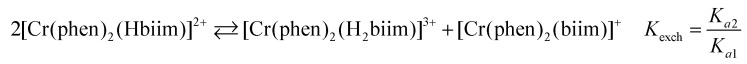


Introducing *K*_a1_ and *K*_a2_ gives *K*_exch_ = 1.2(2) × 10^−4^, from which the ratio of the equilibrium concentration 
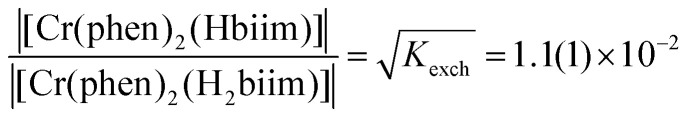
 implies contamination of [Cr(phen)_2_(Hbiim)]^2+^ by *circa* 1% with the more emissive (protonated) [Cr(phen)_2_(H_2_biim)]^3+^ complex (red trace in Fig. 10b).

**Fig. 11:**
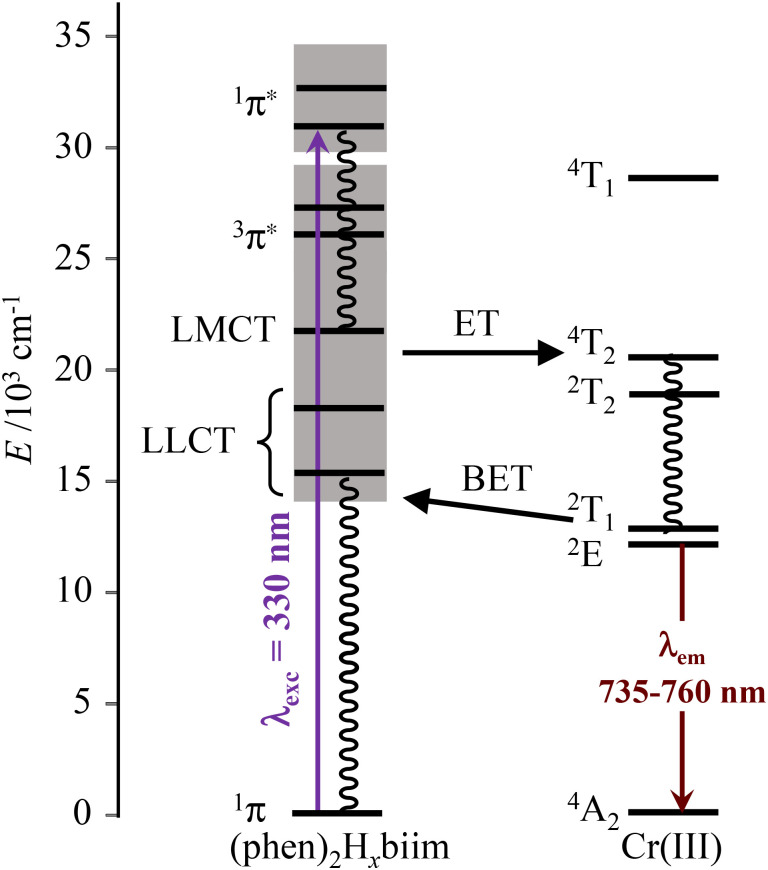
Jablonski diagram of heteroleptic [Cr^III^(phen)_2_(H_*x*_biim)]^(1+*x*)+^ complexes showing the antenna effect upon UV excitation at 330 nm, the existence of low-energy ligand-to-ligand charge transfer excited levels and their potential unfavorable effect on the global quantum yields *via* back energy transfer (BET) processes.

Excited state lifetimes for the NIR emission arising from the Cr(^2^E) excited state upon excitation at 355 nm were recorded in solution at room temperature and at 77 K for [Cr(phen)_2_(H_2_biim)]^3+^, and only at 77 K for [Cr(phen)_2_(Hbiim)]^2+^ (Fig. S67–S70†). In frozen solutions at 77 K, [Cr(phen)_2_(H_2_biim)]^3+^ displays a mono-exponential decay of 2.94 ms, which is typical of Cr(iii)-polyimine complexes (Table 2). The emission decay curve of [Cr(phen)_2_(Hbiim)]^2+^ could not be fit with a mono-exponential function. Since the complex exists as a 98 : 1 : 1 mixture of [Cr(phen)_2_(Hbiim)]^2+^, [Cr(phen)_2_(H_2_biim)]^3+^ and [Cr(phen)_2_(biim)]^+^ (eqn (9)), one expects multi-exponential decays weighted by the mole fractions and the quantum yields of each contributor. A rough bi-exponential fit (Fig. S70†) is compatible with the experimental decay curves showing a long component (1.77(9) ms), which is reminiscent of [Cr(phen)_2_(H_2_biim)]^3+^, and a short contribution (723(30) μs) which is tentatively assigned to [Cr(phen)_2_(Hbiim)]^2+^, for which the smaller energy gap between the emissive doublet state level Cr(^2^E) and the silent LLCT band probably boosts the efficiency of non-radiative decay (Fig. 11 and Appendix 4†). At room temperature, only the protonated complex [Cr(phen)_2_(H_2_biim)]^3+^ is emissive and it exhibits a mono-exponential emission decay of 38 μs in deaerated CH_3_CN. This excited state lifetime is orders of magnitude longer than those of the three isolated homoleptic parent complexes [Cr(H_*x*_biim)_3_]^*n*+^ (Table 2) due to the replacement of two 2,2′-biimidazole ligands with two 1,10-phenanthroline units, which are devoid of high-energy N–H stretching vibrations. The lifetime of [Cr(phen)_2_(H_2_biim)]^3+^ is in the same range as that of [Cr(bpy)_3_]^3+^; however, it is one order of magnitude shorter than that of [Cr(phen)_3_]^3+^ (Table 2). The emission lifetime is reduced to 14 μs in aerated solution because of some additional quenching *via* energy transfer to the ^3^O_2_ molecules present in solution.

Finally, the global luminescence quantum yield of the complex [Cr(phen)_2_(H_2_biim)]^3+^ upon ligand-based excitation at *λ*_exc_ = 450 nm was determined experimentally using the relative method (Fig. S71†). We found *ϕ*^global^_complex_ = 2.8(3) × 10^−3^ in deaerated acetonitrile and *ϕ*^global^_complex_ = 9.5(9) × 10^−4^ in the presence of dioxygen at room temperature. One further notes that the intrinsic Cr(iii)-centered quantum yields calculated with the help of the emission lifetimes *τ*_tot_(^2^E) and *k*_rad_(^2^E) collected at room temperature (Table 2 and eqn (10)) amount to *ϕ*^intrinsic^_complex_ = 2.5 × 10^−3^ in deaerated acetonitrile and *ϕ*^intrinsic^_complex_ = 9.1 × 10^−4^ in aerated acetonitrile.10
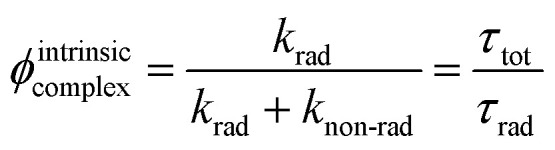


Since (i) *ϕ*^global^_complex_ = *η*_sens_·*ϕ*^intrinsic^_complex_ and (ii) *ϕ*^global^_complex_ ≃ *ϕ*^intrinsic^_complex_, one concludes that the ligand-to-metal sensitization process is close to being quantitative (*η*_sens_ ≈ 100%). These values are in the same range as those reported for [Cr(bpy)_3_]^3+^ (*ϕ*^global, no air^_complex_ = 1.7 × 10^−3^ in deaerated acetonitrile and *ϕ*^global, air^_complex_ = 8.9 × 10^−4^ in aerated water),^70,71,79,80^ but four times smaller than that of [Cr(phen)_3_]^3+^ (*ϕ*^global, no air^_complex_ = 1.2 × 10^−2^ in deaerated water + 1 M HCl),^53,81^ which makes [Cr(phen)_2_(H_2_biim)]^3+^ a moderate emitter for a Cr(iii) complex.^72^

## Conclusions

In line with the only minor interest attracted by the homoleptic [Cr(H_*x*_biim)_3_]^*n*+^ during the past few decades,^35–38^ we confirm here that their low solubility in any common solvent upon stepwise deprotonation, together with their poorly attracting photophysical properties (a short phosphorescence lifetime and negligible intrinsic quantum yield), makes them poorly adapted to be involved in the complex-as-ligand strategy for programming the assemblies of polymetallic optically-active Cr-based complexes. The solution to the problem arises from the successful synthesis of the soluble heteroleptic [Cr(phen)_2_(H_*x*_biim)]^(1+*x*)+^ (*x* = 2–0) complexes *via* a modified Kane-Maguire strategy. The bound H_2_biim ligand can be stepwise deprotonated in solution at pH compatible for further complexation processes with d-block or f-block cations. The deprotonation processes are accompanied by characteristic color changes resulting from the appearance of low-energy intramolecular interligand (phen)π* ← (H_*x*_biim)π (*x* = 2–0, LLCT) ligand-to-ligand charge transfer transitions confirmed by theoretical TD-DFT calculations. The latter reversible process makes the putative heterometallic dyads [Cr(phen)_2_(biim)–M^*z*+^]^(*z*+1)+^ (M^*z*+^ is an open-shell d- or f-block cation) reminiscent of protonated [Cr(phen)_2_(H_2_biim)]^3+^ in terms of photophysical properties, which paves the way for their use as sensitizers in luminescent polymetallic assemblies.

## Conflicts of interest

There are no conflicts to declare.

## Supplementary Material

DT-053-D4DT01608D-s001

DT-053-D4DT01608D-s002
