# Supplementary material for: Taming 2,2′-biimidazole ligands in trivalent chromium complexes
Source: Dalton Trans. 2024 Jul 6;53(38):15801–14. doi: 10.1039/d4dt01608d (PMC11443315; doi:10.1039/d4dt01608d)
Supplement: DT-053-D4DT01608D-s001 [file DT-053-D4DT01608D-s001.pdf]

Electronic Supplementary Material (ESI) for Dalton Transactions.
This journal is © The Royal Society of Chemistry 2024

Taming 2,2'-biimidazole ligands in trivalent chromium complexes†

Julien Chong, Amina Benchohra, Céline Besnard, Laure Guénée, Arnulf Rosspeintner, Carlos M. Cruz, Juan-Ramón Jiménez, and Claude Piguet*

Supporting Information

(131 pages)

Table of content

Appendix 1 Experimental section S4-S12

- Solvents and starting materials S4
- Spectroscopic and analytical measurements S4
- X-ray crystallography S4
- Synthesis of ligands and complexes. S5

Appendix 2 Crystal structures of homoleptic chromium complexes with 2,2'biim S13-S23

- Crystal structure of $[Cr(H_2biim)_3](NO_3)_3$ **1** S13
- Crystal structure of $[Cr(Hbiim)_3]$ **2** S14
- Crystal structure of $[Cr(Hbiim)_2(biim)]PPh_4(MeOH)$ **3** S16
- Crystal structure of *cis*-$[Cr(Me_2biim)_2Cl_2]Cl$ **5** S18
- Crystal structure of $[Cr(Me_2biim)_3](CF_3SO_3)_3$ **6** S19
- Structural analysis S19

Appendix 3 Crystal structures of heteroleptic chromium complexes with 2,2'biim S24-S27

- Crystal structure of $[Cr(phen)_2(H_2biim)](CF_3SO_3)_3(H_2O)_{0.25}$ **7** S24
- Crystal structure of $[Cr(phen)_2(Hbiim)](CF_3SO_3)_2.(H_2O)_{0.5}$ **8** S24
- Crystal structure of $[Cr(phen)_2(biim)]CF_3SO_3(CH_3OH)_{1.5}$ **9** S26

Appendix 4 Theoretical calculations of the electronic structures of heteroleptic complexes $[Cr(phen)_2(H_xbiim)]^{(1+x)+}$. S28-S49

- Theoretical studies S28
- Optimized geometries S29
- Ab initio ligand field analysis S29
- Complete active space analysis of excited states S30
- Electronic transitions S35
- Cartesian coordinates S44

Supporting Data Tables S1-S41 and Figures S1-S71 S50-S131

- pK_a values (Table S1) S50
- Crystal data of [Cr(H_2biim)$_3$](NO_3)$_3$ **1** (Tables S2-S3, Fig. S1) S52
- Crystal data of [Cr(Hbiim)$_3$] **2** (Tables S4-S6, Fig. S2) S54
- Crystal data of [Cr(Hbiim)$_2$(biim)]PPh_4(MeOH) **3** (Tables S7-S9, Fig. S3) S57
- Crystal data of [Cr(Hbiim)$_2$(biim)]PPh_4 **4** (Tables S10-S12, Fig. S4) S60
- Crystal data of *cis*-[Cr(Me_2biim)$_2$$Cl_2$]Cl **5** (Tables S13-S15, Figs S5-S6) S63
- Crystal data of [Cr(Me_2biim)$_3$](CF_3SO_3)$_3$ **6** (Tables S16-S17, Fig. S7) S66
- Crystal data of [Cr(phen)$_2$(H_2biim)](CF_3SO_3)$_3$ **7** (Tables S18-S20, Fig S8) S68
- Crystal data of of [Cr(phen)$_2$(Hbiim)](CF_3SO_3)$_2$ **8** (Tables S21-S23, Figs S9-S10) S71
- Crystal data of of of [Cr(phen)$_2$(biim)]CF_3SO_3 **9** (Tables S24-S26, Figs S9-S11) S74
- FT-IR spectra of homoleptic complexes (Tables S27-S32, Figs. S13-S18) S77
- ESI-MS spectra of homoleptic complexes (Tables S33-S35 , Figs S19-S38) S84
- Photophysics for homoleptic complexes **1**-**6** (Tables S36-S40 , Figs S39-S56) S106
- FT-IR spectra of heteroleptic complexes (Fig. S57) S120
- ESI-MS spectra of heteroleptic complexes (Table S41, Figs S58-S62) S121
- Photophysical data for heteroleptic complexes **7**-**9** (Figs S63-S71) S125

Appendix 1 Experimental section

Solvents and starting materials

Chemicals were purchased from Sigma-Aldrich and Acros and used without further purification unless otherwise stated. Dry reagents were either purchased as packed under inert atmosphere with acroseal and molecular sieves present, or distilled by appropriate procedures. Dry solvents were distilled over either calcium hydride or metallic sodium.[A1-1] Silica-gel plates (Merck, 60 F_{254}) were used for thin-layer chromatography, SiliaFlash® silica gel P60 (0.04-0.063 mm), and Acros silica gel 60 (0.035-0.07 mm) were used for preparative column chromatography.

Spectroscopic and analytical measurements

^{1}H and ^{13}C NMR spectra were recorded at 298 K on a Bruker Avance 400 MHz spectrometer equipped with BCU temperature control for variable temperature measurements. Chemical shifts are given in ppm with respect to TMS. Pneumatically-assisted electrospray (ESI-MS) mass spectra were recorded from $\sim 1\cdot 10^{-4}$ M (ligands) and $\sim 1\cdot 10^{-3}$ M (complexes) solutions on an Applied Biosystems API 150EX LC/MS System equipped with a Turbo Ionspray source. Elemental analyses were performed by K. L. Paglia from the Microchemical Laboratory of the University of Geneva. Electronic spectra in the UV-Vis region were recorded at 293 K from solutions in CH_3CN with a Perkin-Elmer Lambda 1050 using quartz cells of 0.1 or 1.0 mm path length. Solid-state absorption spectra were recorded with a Perkin-Elmer Lambda 900 using capillaries.

The emission spectra were recorded using a Fluorolog (Horiba Jobin-Yvon) instrument equipped with an iHR320 imaging spectrometer, a 450 W xenon lamp illuminator (FL-1039A/40A) and a water-cooled photomultiplier tube (PMT Hamamatsu R928P). The emission spectra were corrected for the wavelength-dependent sensitivity of the PMT. Time-resolved data were collected using a digital oscilloscope (Tektronix MDO4104C) coupled to a water-cooled photomultiplier tube (PMT Hamamatsu R928P) or to a time-gated photomultiplier module (Hamamatsu H11526-20-NF). Pulsed excitation at 355 nm was achieved using the third harmonic of a pulsed Nd:YAG laser (Quantel Q-Smart 850). Variable temperature measurements were done using a closed-cycle cryosystem (Janis, CCS-900/204N) with the sample sitting in the exchange gas (helium) to achieve efficient cooling. Complexes of known corrected molecular weight were dissolved in acetonitrile to obtain ~1 mM solutions that were immobilized in 2 mm diameter cylindrical quartz cuvettes. The cuvettes, sealed with fast drying silver agar gel were mounted on a metallic copper sample holder.

X-ray crystallography

X-ray crystallography

Summary of crystal data, intensity measurements and structure refinements for complexes $[Cr(H_2biim)_3](NO_3)_3$ **1**, $[Cr(Hbiim)_3]$ **2**, $[Cr(Hbiim)_2(biim)]PPh_4(CH_3OH)$ **3**,

$[Cr(Hbiim)_2(biim)]PPh_4$ **4**, $[Cr(Me_2biim)_3](CF_3SO_3)_3$ **5**, *cis*-$[Cr(Me_2biim)_2Cl_2]Cl(CH_3OH)_3$ **6**, $[Cr(phen)_2(H_2biim)](CF_3SO_3)_3(H_2O)_{0.25}$ **7**, $[Cr(phen)_2(Hbiim)](CF_3SO_3)_2(H_2O)_{0.5}$ **8** and $[Cr(phen)_2(biim)]CF_3SO_3(CH_3OH)_{1.5}$ **9** are collected in Tables S2-S26 with pertinent bond lengths, bond angles. ORTEP views with pertinent numbering schemes are gathered in Figures S1-S11. The crystals were mounted on Hampton cryoloops with protection oil. X-ray data collections were performed with a XtaLAB Synergy-S diffractometer equipped with a hybrid pixel array "hypix arc 150" detector(compounds **1-8**) or a Supernova diffractometer with an Atlas CCD camera (compound **9)**. The structures were solved by using direct methods.[A1-2,A1-3]. Full-matrix least-square refinements on F^2 were performed with SHELX2014.[A1-4] CCDC 2355642-2355650 contain the supplementary crystallographic data for this paper. These data can be obtained free of charge from The Cambridge Crystallographic Data Centre via https://www.ccdc.cam.ac.uk/structures/.

Synthesis of ligands and complexes.

Synthesis of 2,2'-Biimidazole (H_2biim) according to literature[A1-5] with modifications.

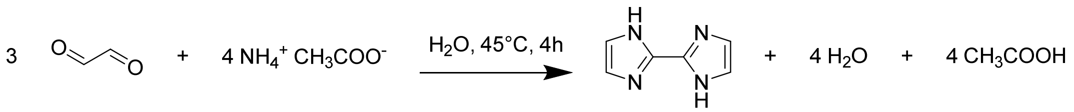

In a flask, 100g (1.3 mol) of ammonium acetate were suspended in 20ml of H_2O. Aqueous glyoxal (40%-wt, 50mL, 0.44 mol) was added dropwise over the course of 2h at 45°. The brown suspension was stirred at 45° for an extra hour. The brown suspension was filtered on Büchner and washed with 200mL of H_2O then with 200mL of acetone. The obtained brown crude solid product was recrystallized in 300mL of hot DMF. The product was further purified by repeating the recrystallization process a second time. The desired product was obtained as white needle-shaped crystals (5.58 g, 0.042 mol, 29% yield).

ESI-LRMS (*m*/*z*); calculated for $C_6H_7N_4^+$, 135.066 [H_2biim+H^+], found: 135.2.

^{1}H NMR (400 MHz, DMSO) δ 12.67 (s, 2H), 7.06 (s, 4H).

Synthesis of 1,1′-Dimethyl-2,2′-bi-1*H*-imidazole (Me_2biim) according to literature[A2-6] with modifications.

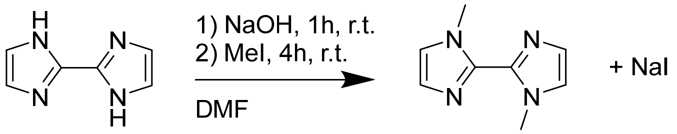

In a flask, 1.50 g (11.2 mmol) of 2,2'-biimidazole were mixed with 30 mL of DMF. To the suspension, 1.00 g (25.0 mmol, 2.24 equivalents) of solid NaOH was added. After 1 h of stirring at room temperature, 1.45 mL (23.5 mmol, 2.10 equivalents) of MeI were added. The mixture was stirred 4 h at room temperature, then 100 mL of H_2O were added to the reaction mixture, which was then extracted with 3 x 100mL of CH_2Cl_2. The organic phase was washed with additional 100mL of H_2O,

then dried with Na_2SO_4. CH_2Cl_2 and the remaining DMF were evaporated under reduced pressure yielding an orange solid. The solid was recrystallized in 50 mL of acetone and 899 mg (5.54 mmol, 50% yield) of the desired product were obtained as a white crystalline solid.

ESI-LRMS (*m/z*); calculated for $C_8H_{11}N_4^+$: 163.097 [Me_2biim+H^+], found 163.0.

^1H NMR (400 MHz, $CDCl_3$) δ 7.11 (d, *J* = 1.2 Hz, 2H), 6.95 (d, *J* = 1.2 Hz, 2H), 4.04 (s, 6H).

Synthesis of [Cr(H_2biim)$_3$](NO_3)$_3$ according to literature[A1-7,A1-8] with modifications.

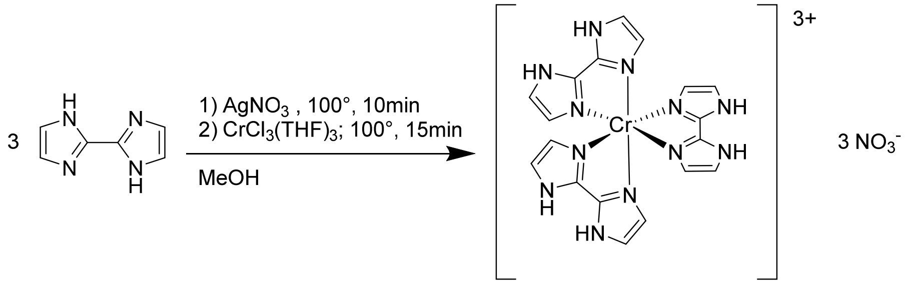

In a sealed microwave vial, 302 mg (2.25 mmol, 4.2 equivalents) of 2,2'-biimidazole and 385mg (2.27 mmol, 4.2 equivalents) of $AgNO_3$ were added in 10 mL of MeOH. The vial was heated 10 min at 100° in the microwave oven to form the intermediate Ag(H_2biim)NO_3 as a white precipitate. 200mg (0.535 mmol) of anhydrous $CrCl_3(THF)_3$ were added and the vial was heated 15 min at 100°C in the microwave oven. The grey precipitate of AgCl was removed by filtration and abundantly washed with 50 mL of MeOH, then discarded. The orange filtrate was evaporated to 20 mL then put in the freezer (-18°C) overnight, 228 mg (0.357 mmol, 67% yield) of orange powder were collected after filtration. Single crystals could be obtained by vapor diffusion of Et_2O in a methanolic solution of the product.

Elemental analysis: calculated for [Cr(H_2biim)$_3$](NO_3)$_3$·0.9 H_2O: C 32.92, H 3.04, N 32.00; Found C 32.61, H 2.70, N 31.68.

ESI-MS (*m/z*); calculated for $C_{18}H_{16}CrN_{12}^+$: 452.1021 [Cr(H_2biim)(Hbiim)$_2$]$^+$, found 452.103; calculated for $C_{18}H_{17}CrN_{12}^{2+}$: 226.5547 [Cr($H_2$biim)$_2$(Hbiim)]$^{2+}$, found 226.555 .

Synthesis of [Cr(Hbiim)$_3$].

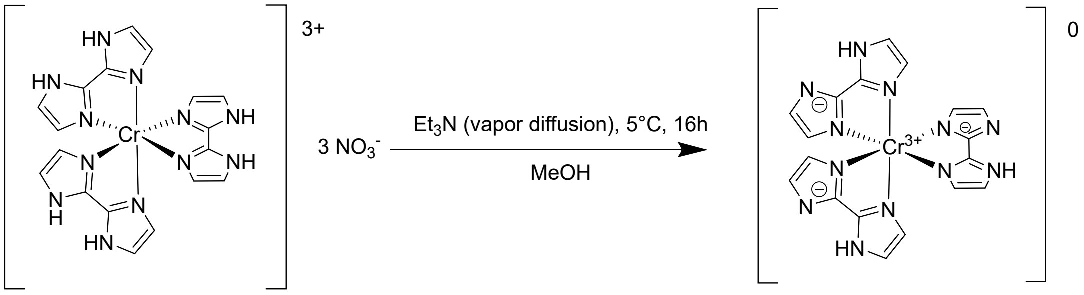

In a beaker, 100 mg (0.156 mmol) of [Cr(H_2biim)$_3$](NO_3)$_3$ were dissolved in 30 mL of MeOH. The beaker was placed in a bigger closed container, filled with 3mL of Et_3N in a way that allow vapor diffusion of Et_3N into the solution of complex. After 16h of vapor diffusion in the fridge (4°C), the

crystalline suspension was filtered and washed with 50 mL of MeOH yielding to 43 mg (0.0953 mmol, 61% yield) of a crystalline orange powder.

Elemental analysis: calculated for $[Cr(Hbiim)_3]\cdot 0.8\ H_2O\cdot 0.15\ CH_3OH$: C 46.32, H 3.68, N 35.72; Found C 46.15, H 3.38, N 35.43.

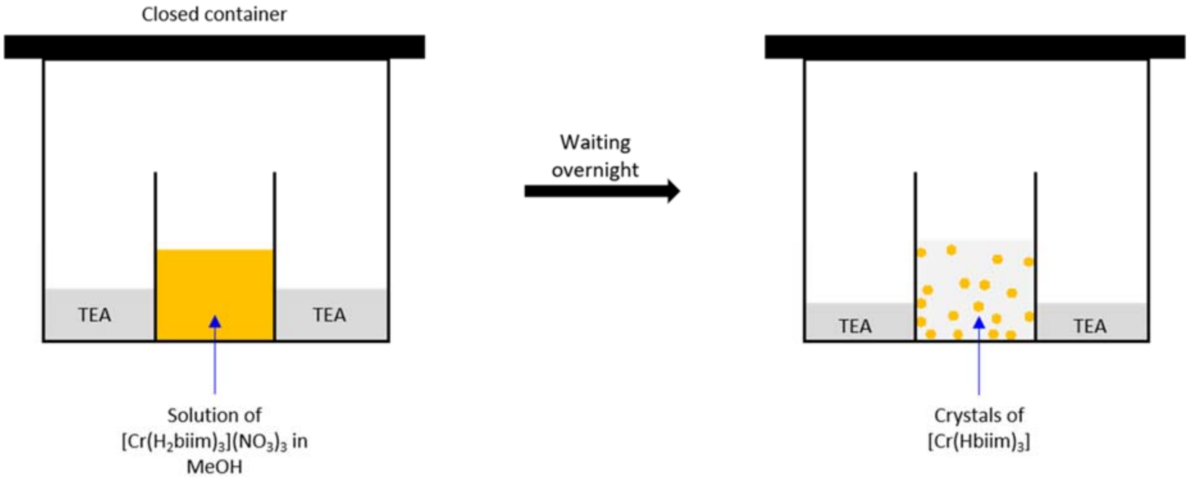

Figure A1-1 Scheme of the vapor diffusion performed for the synthesis of $[Cr(Hbiim)_3]$, TEA = Et_3N.

Synthesis of $[Cr(Hbiim)_2(biim)]PPh_4$.

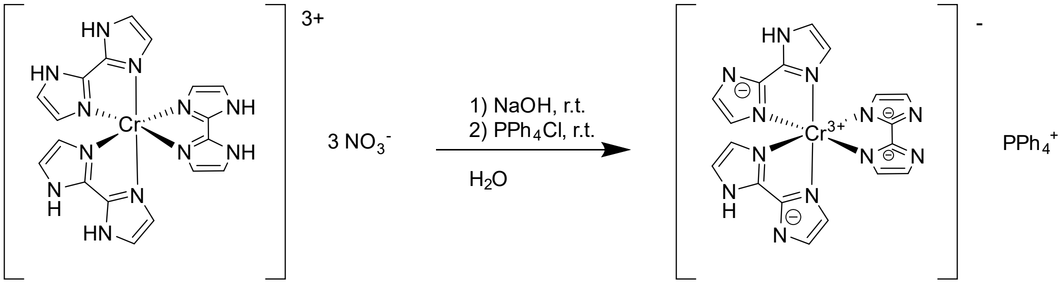

In a beaker, 50 mg (0.078 mmol) of $Cr(H_2biim)_3(NO_3)_3$ was dissolved in 2 mL of H_2O to give a clear yellow/orange solution. 2.0 mL of aqueous NaOH 1M (2.0 mmol, 26 equivalents). To the clear orange solution was added 1.0 mL of PPh_4Cl 10% w/V in H_2O (0.27 mmol, 3.4 equivalents), an orange precipitate immediately formed. The suspension was filtered, the orange solid was washed with 2 x 15 mL of H_2O then dried. The obtained powder was dissolved in the minimum of MeOH (10 mL). Vapor diffusion of ^{t}BuOMe into the solution led to the formation of block shaped crystals after 3 days. These crystals were then collected by filtration. 41 mg of orange crystals were obtained (0.052 mmol, yield 66%).

Elemental analysis: calculated for $[Cr(Hbiim)_2(biim)]PPh_4\cdot 0.45\ H_2O$: C 62.86, H 4.41, N 21.07; found C 62.86, H 4.04, N 20.92.

ESI-MS (*m/z*); calculated for $C_{18}H_{14}CrN_{12}^-$: 450.0875 $[Cr(Hbiim)_2(biim)]^-$, found 450.087.

Synthesis of [Cr(Me2biim)3](CF3SO3)3.

Method 1:

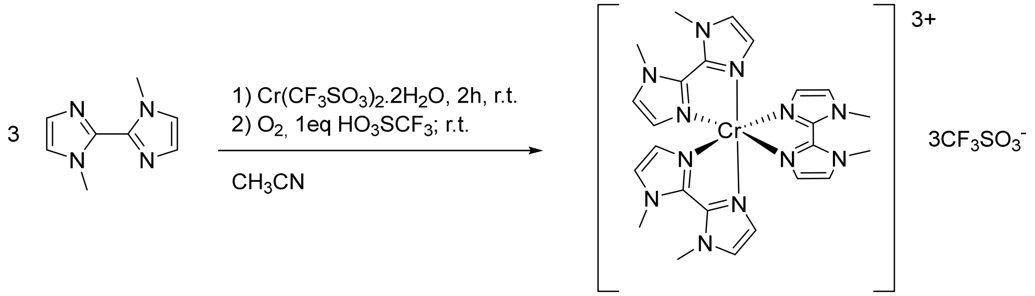

Under argon atmosphere, 100 mg (0.62 mmol, 3.3 equivalents) of Me_2biim were added in 2.4 mL of a blue solution of a $Cr(CF_3SO_3)_2{\cdot}2H_2O$ in CH_3CN $7.8{\cdot}10^{-2}$ M (0.19 mmol) previously prepared in glovebox with distilled CH_3CN saturated in argon.[A1-9] The solution immediately turned red, then the solution was stirred 10 min at room temperature. The solution was then open to air and stirred 5 min to allow the oxidation in Cr^{3+}, then 5 mL of a solution of HO_3SCF_3 $4.6{\cdot}10^{-2}$ M in CH_3CN were added. The CH_3CN was removed under reduced pressure and replaced with 10 mL of MeOH. The yellow solid insoluble in MeOH was separated from the red solution by filtration and washed with 2 x 3 mL of cold MeOH and 2 mL of Et_2O The powder was dissolved in ACN and recrystallized by vapor diffusion of Et_2O into the solution. 24 mg (0.024 mmol, 13% yield) of yellow block shaped crystals of $[Cr(Me_2biim)_3](CF_3SO_3)_3$ were collected.

Method 2:

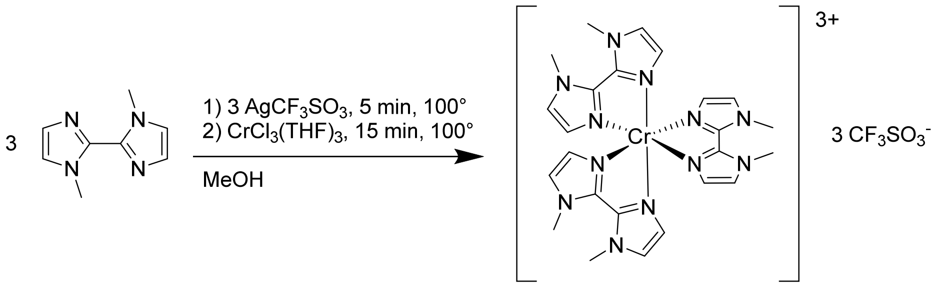

In a sealed microwave vial, 102 mg (0.629 mmol, 3.36 equivalents) of Me_2biim and 0.155 mg (0.603 mmol, 3.22 equivalents) of $AgCF_3SO_3$ were added in 10mL of MeOH. The vial was heated 5 min at 100°C with the microwave, the solution was clear. 70 mg (0.187 mmol, 1 equivalent) of anhydrous $CrCl_3{\cdot}3THF$ were added and the vial was heated 15 min at 100°C with the microwave. The white precipitated AgCl was filtered and discarded. The dark orange solution was evaporated to 3 mL under reduced pressure and put in the freezer (-18 °C) for 1h. The yellow precipitate was separated from the brown solution by filtration and washed with 2 x 3 mL of cold MeOH and 2 mL of Et_2O. The powder was dissolved in CH_3CN and recrystallized by vapor diffusion of Et_2O into the solution. 33

mg (0.0335 mmol, 18% yield) yellow block shaped crystals of $[Cr(Me_2biim)_3](CF_3SO_3)_3$ were collected.

Method 3 (2 steps):

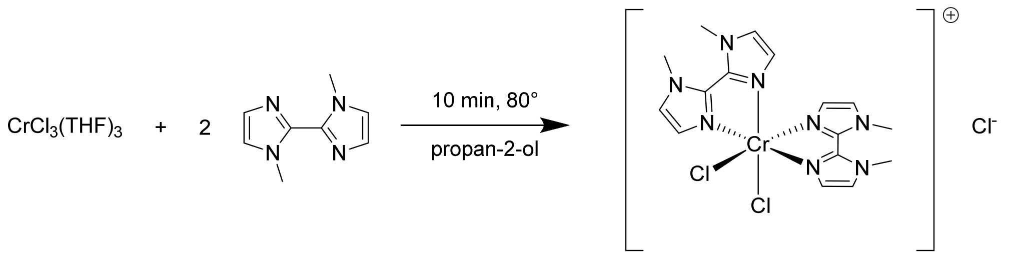

In a sealed microwave vial, 202 mg (1.25 mmol) of Me_2biim and 220 mg (0.559 mmol, 3.00 equivalents) of $CrCl_3{\cdot}3THF$ were added in 5 mL of isopropanol. The vial was then heated 10 min at 80°C in the microwave oven. The green powder was filtered, washed with 10 mL of isopropanol, 10 mL CH_2Cl_2 and 10 mL of Et_2O. 226 mg (0.468 mmol, 80% yield) of green powder of $[Cr(Me_2biim)_2Cl_2]Cl$ were obtained.

Elemental analysis: calculated for $[Cr(Me_2biim)_2Cl_2]Cl{\cdot}0.95H_2O$: C 38.45, H 4.42, N 22.42; found: C 38.10, H 4.08, N 22.26.

ESI-LRMS (*m*/*z*); calculated for $C_{16}H_{20}Cl_2CrN_8^+$: 446.058 $[Cr(Me_2biim)_2Cl_2]^+$, found 446.3.

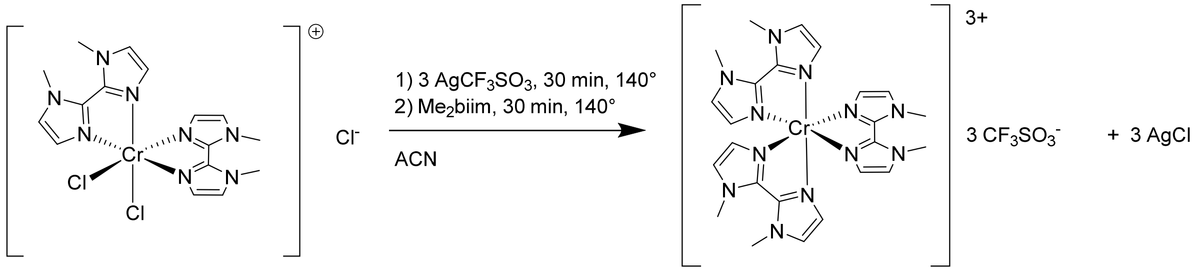

In a sealed microwave vial, 100 mg (0.207 mmol) of $[Cr(Me_2biim)_2Cl_2]Cl$, 159 mg (0.619 mmol) of $AgCF_3SO_3$ and 2 mL of ACN were added. The vial was heated 30 min at 140° in the microwave oven. A white precipitate formed with an orange solution containing the intermediate $[Cr(Me_2biim)_2(CF_3SO_3)_2]^+$ (*m*/*z* calc. for $[Cr(Me_2biim)_2(CF_3SO_3)_2]^+$ 674.025; found: 674.0).

The solution was separated from the precipitate by filtration and transferred into another vial containing 40 mg (0.25 mmol, 1.2 equivalents) of Me_2biim. The vial was heated 30 min at 110°C in the microwave oven. The orange solution was evaporated under reduced pressure, and dissolved in a minimum of MeOH (5 mL). The insoluble yellow solid was separated from the solution by filtration. The yellow powder was dissolved in a minimum of CH_3CN (5 mL) and recrystallized by vapor diffusion of Et_2O into the solution. After filtration, 82 mg (0.0832 mmol, 40% yield) of yellow crystals were obtained.

Elemental analysis: calculated for $[Cr(Me_2biim)_3](CF_3SO_3)_3$: C 32.90, H 3.07, N 17.05; found: C 32.64, H 3.12, N 16.99.

ESI-MS (*m*/*z*): calculated for $C_{26}H_{30}CrF_6N_{12}O_6S_2^+$: 836.1157 $[Cr(Me_2biim)_3(CF_3SO_3)_2]^+$, found: 836.115; calculated for $C_{25}H_{30}CrF_3N_{12}O_3S^{2+}$: 343.5816 $[Cr(Me_2biim)_3(CF_3SO_3)]^{2+}$, found: 343.580; calculated for $C_{24}H_{30}CrN_{12}^{3+}$: 179.4035 $[Cr(Me_2biim)_3]^{3+}$, found: 179.403.

Synthesis of $[Cr(phen)_2Cl_2]Cl$ according to literature procedure with modifications.[A1-10]

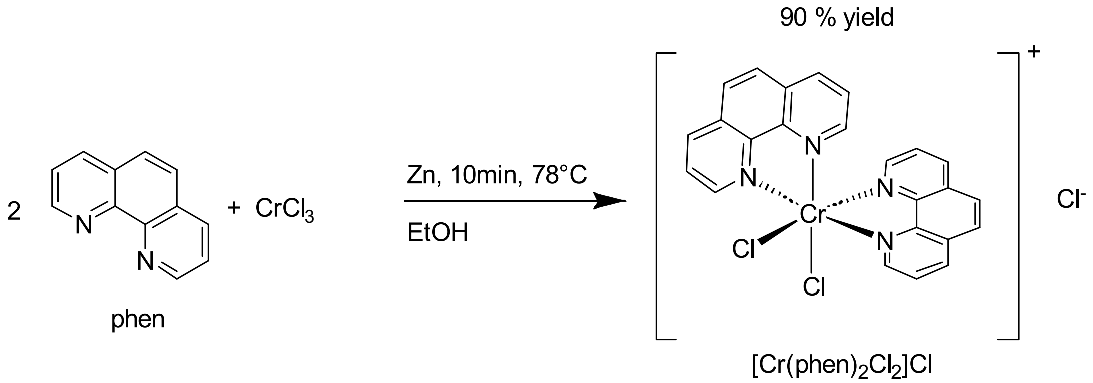

Anhydrous $CrCl_3$ (1.00 g, 6.32 mmol), 1,10-phenanthroline (3.00 g, 16.6 mmol) and metallic zinc pellets (2.2 g) were introduced in a 100 mL flask. To the mixture, 20 mL of EtOH were added and the mixture was heated 10 min at reflux. Formation of green crystals was observed during cooling of the flask to room temperature. The crystals were separated from the brown solution by filtration, washed with 30 mL of EtOH, and the Zn pellets were removed with tweezers (2.94 g, 5.67 mmol, 90% yield).

Elemental analysis: calculated for $[Cr(phen)_2Cl_2]Cl\cdot 1.55H_2O$: C 52.73, H 3.52, N 10.25; found: C 52.68, H 3.39, N 10.13.

ESI-LRMS (*m*/*z*): calculated for $[Cr(phen)_2Cl_2]^+$ ($C_{24}H_{16}Cl_2CrN_4^+$): 482.0152, found: 482.4.

Synthesis of $[Cr(phen)_2(H_2biim)](CF_3SO_3)_3$.

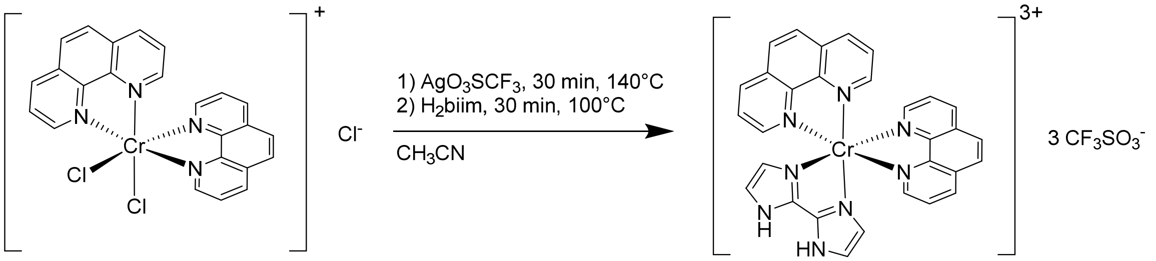

500 mg (0.964 mmol) of $[Cr(phen)_2Cl_2]Cl$, 2.09 g (8.13 mmol) of AgO_3SCF_3 and 10 mL of CH_3CN were introduced in a sealed microwave vial. The mixture was heated 30 min at 140°C in the microwave oven. A white precipitate of AgCl was formed with a red solution containing intermediate $[Cr(phen)_2(CF_3SO_3)_2]^+$ (*m*/*z* calc. for $[Cr(phen)_2(CF_3SO_3)_2]^+$ 709.981; found: 710).

The white precipitate was separated by filtration and discarded. The red solution and 250 mg (1.86 mmol) of H_2biim were introduced into a microwave vial, and the mixture was heated 30 min at 100 °C in the microwave oven. The dark orange solution was evaporated to dryness under reduced pressure and resolubilized in 10 mL of MeOH. The solution was filtered to remove some insoluble grey precipitate then precipitated in Et_2O and filtered. The orange powder was redissolved in 10 mL of a solution of HO_3SCF_3 10^{-2} mol/L in MeOH, a small quantity of precipitate did not dissolve and was filtered off. The yellow solution was recrystallized by vapor diffusion of Et_2O into the solution overnight. 372 mg of orange crystals were isolated by filtration (0.374 mmol, 37% yield).

Elemental analysis: calculated for [Cr(phen)$_2$(H_2biim)](CF_3SO_3)$_3$: C 39.89, H 2.23, N 11.28; found: C 39.71, H 2.26, N 11.29.

ESI-MS (*m*/*z*): calculated for [Cr(phen)$_2$(Hbiim)]$^{2+}$ ($C_{30}H_{21}CrN_8^{2+}$): 272.5641, found: 272.566; calculated for [Cr(phen)$_2$(Hbiim)](CF_3SO_3)$^+$ ($C_{31}H_{21}CrF_3N_8O_3S^+$): 694.0810, found: 694.082.

Synthesis of [Cr(phen)$_2$(Hbiim)](CF_3SO_3)$_2$.

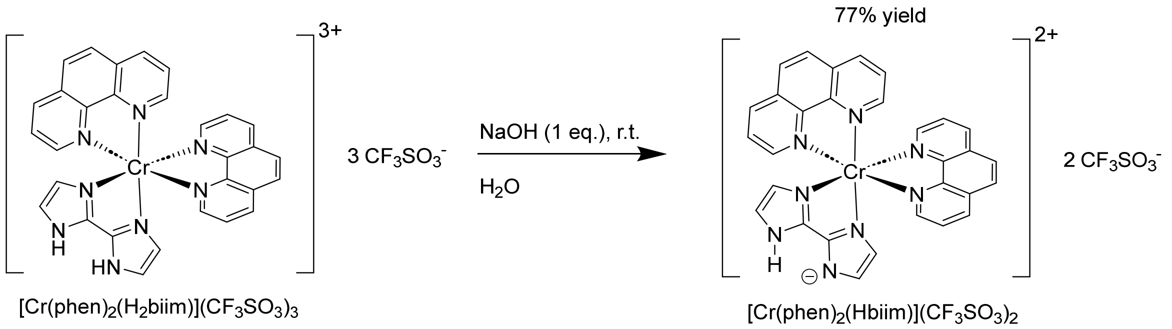

In a beaker, 200 mg (0.201 mmol) of [Cr(phen)$_2$(H_2biim)](CF_3SO_3)$_3$ were dissolved in 5 mL of H_2O. 200 μL of aqueous NaOH 1M (0.200 mmol) were added to the orange solution. The color of the solution immediately turned red and red crystals started to form. The beaker was put in the fridge (4°C) overnight. The red crystals were collected by filtration, washed with 2 x 2 mL of cold H_2O then dried in vacuum yielding 130 mg (0.154 mmol, 77% yield) of a red crystalline powder.

Elemental analysis: calculated for [Cr(phen)$_2$(Hbiim)](CF_3SO_3)$_2$: C 45.56, H 2.51, N 13.28; found: C 45.29, H 2.42, N 13.23.

ESI-MS (*m*/*z*): calculated for [Cr(phen)$_2$(Hbiim)]$^{2+}$ ($C_{30}H_{21}CrN_8^{2+}$): 272.5641, found: 272.566; calculated for [Cr(phen)$_2$(Hbiim)](CF_3SO_3)$^+$ ($C_{31}H_{21}CrF_3N_8O_3S^+$): 694.0810, found: 694.082.

Synthesis of [Cr(phen)$_2$(biim)]CF$_3$SO$_3$.

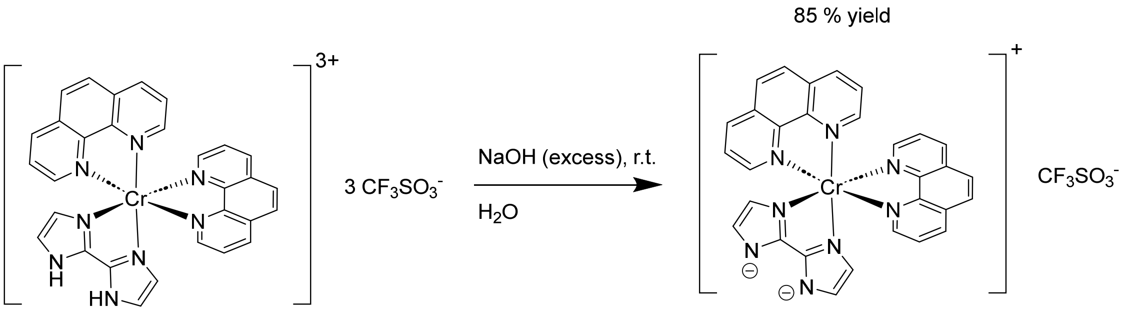

[Cr(phen)$_2$(H$_2$biim)](CF$_3$SO$_3$)$_3$ [Cr(phen)$_2$(biim)]CF$_3$SO$_3$

In a beaker, 200 mg (0.201 mmol) of [Cr(phen)$_2$(H$_2$biim)](CF$_3$SO$_3$)$_3$ were dissolved in 5 mL of H_2O. 1.50 mL of aqueous NaOH 1M (1.50 mmol) were added to the orange solution. The color of the solution immediately turned dark brown and black crystals started to form. The beaker was put in the fridge (4°C) overnight. The black crystals were collected by filtration, washed with 2 x 2 mL of cold H_2O then dried in vacuum yielding 118 mg (0.170 mmol, 85% yield) of black crystalline powder. Elemental analysis: calculated for [Cr(phen)$_2$(biim)]CF$_3$SO$_3$ + 0.55 H_2O: C 52.93, H 3.02, N 15.93; found: C 53.07, H 3.21, N 16.05.

ESI-MS (*m*/*z*): calculated for [Cr(phen)$_2$(Hbiim)]$^{2+}$ ($C_{30}H_{21}CrN_8{}^{2+}$): 272.5641, found: 272.566; calculated for [Cr(phen)$_2$(Hbiim)](CF$_3$SO$_3$)$^+$ ($C_{31}H_{21}CrF_3N_8O_3S^+$): 694.0810, found: 694.082.

References

A1-1 W. L. F. Armarego, C. L. L.Chai, Purification of Laboratory Chemicals, Elsevier, 2009.

A1-2 G. M. Sheldrick, *Acta Cryst. A,* 2008, **64**, 112-122.

A1-3 M. C. Burla, M. Camalli, B. Carrozzini, G. L. Cascarano, C. Giacovazzo, G. Polidori and R. Spagna, *Acta Cryst. A*, 1999, **55**, 991-999.

A1-4 G. M. Sheldrick, *Acta Cryst. C,* 2015, **71**, 3-8.

A1-5 M. N. Feng, G. Y. Zhao, H. L. Gao and S. J. Zhang, *Aust. J. Chem.*, 2015, **68**, 1513-1517.

A1-6 H. M. Zhang, X. G. Yan, J. Zhao, X. L. Yang, Z. Huang, G. J. Zhou and Y. Wu, *RSC Adv.*, 2015, **5**, 88758-88766.

A1-7 L. M. Gruia, F. D. Rochon and A. L. Beauchamp, *Can. J. Chem.*, 2006, **84**, 949-959.

A1-8 M. J. Silvero, W. J. Peláez, P. F. Garcia and G. A. Argüello, *RSC Adv.*, 2014, **4**, 15507-15510.

A1-9 M. Cantuel, G. Bernardinelli, D. Imbert, J.-C. G. Bünzli, G. Hopfgartner and C. Piguet, *J. Chem. Soc., Dalton Trans.*, 2002, 1929-1940.

A1-10 B. Doistau, G. Collet, E. Acuna Bolomey, V. Sadat-Noorbakhsh, C. Besnard and C. Piguet, *Inorg. Chem.*, 2018, **57**, 14362-14373.

Appendix 2 Crystal structures of homoleptic chromium complexes with 2,2'biimidazole.

Crystal structure of [Cr(H_2biim)$_3$](NO_3)$_3$ 1 (Tables S2-S3).

Single crystal of [Cr(H_2biim)$_3$](NO_3)$_3$ suitable for X-ray diffraction were obtained by vapor diffusion of Et_2O into a methanolic solution of the complex.

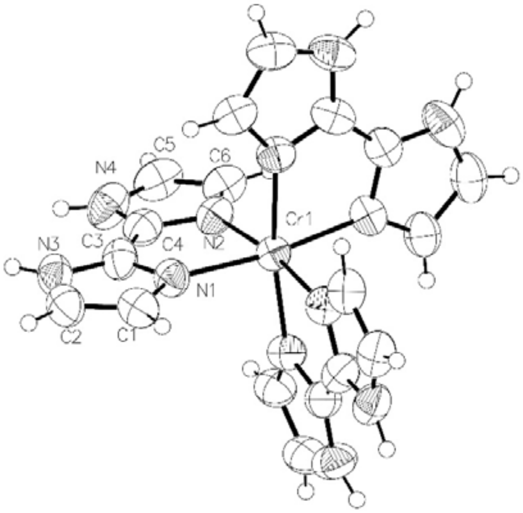

Figure A2-1 ORTEP view of the complex [Cr(H_2biim)$_3$]$^{3+}$ in the crystal structure of [Cr(H_2biim)$_3$](NO_3)$_3$.

The complex crystallizes in the trigonal space group R3c, which differs only slightly from the previously reported structure of the same complex in the R-3c space group.[A2-1] (Our data were also consistent with a model in the space group R-3c, at the expense of larger ellipsoids, especially for O2 and the position corresponding to N3/N4. The model in R3c was then preferred.) The Cr atom is surrounded by 6 N atoms in the first coordination sphere, which are arranged in a pseudo octahedral geometry. The distortion from ideal octahedral geometry will be discussed later. The unit cell does not contain any solvent molecule. The most noticeable feature is the presence of a hydrogen bonds network between the NO_3^- anions and the hydrogens located on the nitrogen atoms of the ligand (Figure **A2-2**A2-2).

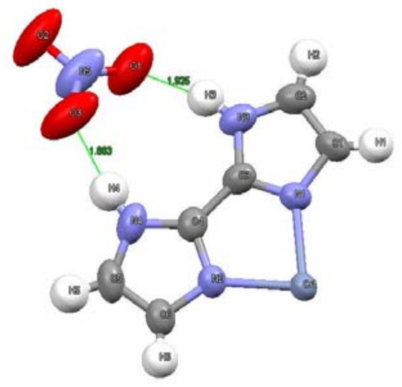

Figure A2-2 View of the asymmetric unit in the crystal structure of $[Cr(H_2biim)_3](NO_3)_3$.

The O···H distance between the NO_3^- and the imidazole is 1.9 Å and the N-O distance is 2.75 Å in the N-H···O hydrogen bond. This is a good indication of the acidic nature of the biimidazole-NH groups when the ligand is coordinated to the Cr^{3+} ion.

Crystal structure of $[Cr(Hbiim)_3]$ 2 (Tables S4-S6).

The neutral complex $[Cr(Hbiim)_3]$ is highly insoluble in common organic solvents. Therefore, it was difficult to recrystallize this compound. To overcome this problem, we decided to form crystals by vapor diffusion of triethylamine into a methanolic solution of $[Cr(H_2biim)_3](NO_3)_3$. The triethylamine could deprotonate slowly enough $[Cr(H_2biim)_3]^{3+}$ to form crystals of $[Cr(Hbiim)_3]$ instead of a precipitate. Crystals suitable for X-ray diffraction were obtained (Figure A2-3). Unfortunately, the solvents molecules present in the large cavities between the complexes are highly disordered, so the global structure refinement was really difficult to perform. The only option was to perform a SQUEEZE bypass procedure as implemented in Olex2 software,[A2-2] which basically consists in removing the electron density created by the solvents molecules, but making the structure not fully reliable for detailed structure analysis. A solvent mask was calculated, and 1103 electrons were found in a volume of $3805A^3$ in one void per unit cell. This is consistent with the presence of $10[CH_3OH]$ per formula unit which account for 1080 electrons per unit cell.

This problem persisted even after several attempts to crystallize the complex in different crystallization conditions. Gruia et al[A2-1] encountered the same problem when they tried to resolve the structure of $[Cr(Hbiim)_3](C_6H_{14}O)_{2.6}$. The only reliable structure available was $[Cr(Hbiim)_3](C_6H_6)(H_2O)_2$ that they unexpectedly obtained in condition difficult to replicate.[A2-1] This structure was used for calculation of the octahedral distortion which is detailed later.

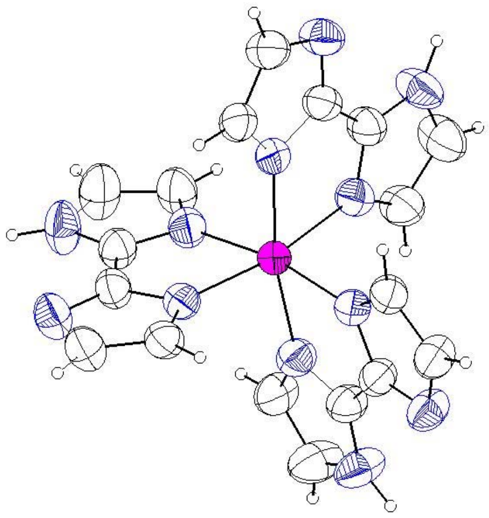

Figure A2-3 ORTEP view of the complex [Cr(Hbiim)3].

The packing of the complexes is mainly driven by hydrogen bonds interactions. In this case, the half-deprotonated biimidazole is a hydrogen bond donor and acceptor at the same time allowing a complementary interaction with another half-deprotonated biimidazole of a neighboring complex (Figure A2-4). Each complex form 3 pairs of hydrogen bonds (one pair for each ligand) with 3 neighboring complexes. Again, the N···H and the N···H-N distance are relatively short (1.89 Å and 2.76 Å respectively, Table S5) showing strong interactions. A consequence of the hydrogen bonding network is the very low solubility of the complex in common solvents.

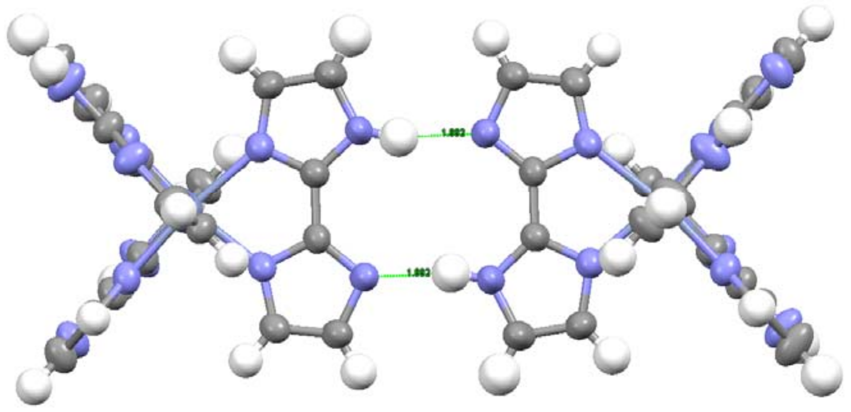

Figure A2-4 View of the hydrogens bonds interactions between two neighboring complexes in the crystal structure of [Cr(Hbiim)3].

Crystal structure of [Cr(Hbiim)$_2$(biim)]PPh$_4$(MeOH) 3 (Tables S7-S9).

The crystal structure of [Cr(Hbiim)$_2$(biim)]PPh$_4$(MeOH) was recorded with single crystals obtained by vapor diffusion of ^{t}BuOMe into a methanolic solution of the complex. The complex crystallizes in the $P2_1/c$ space group with one MeOH molecule in the asymetric unit (Figure A2-5).

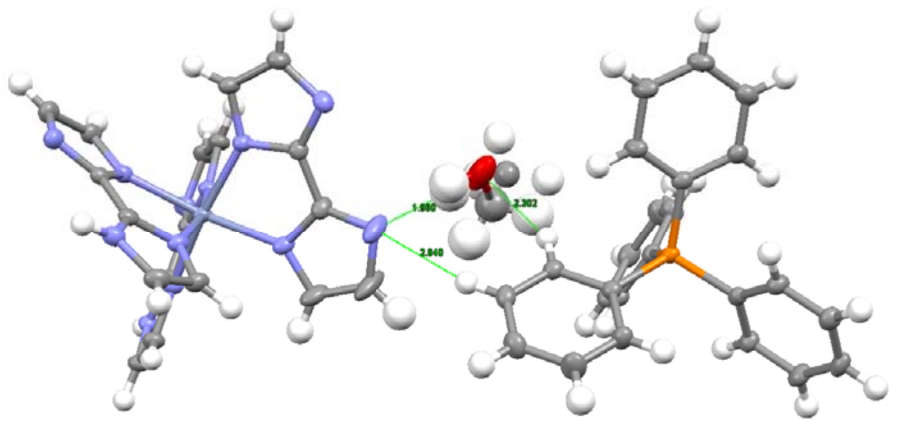

Figure A2-5 View of the asymmetric unit in the crystal structure of [Cr(Hbiim)$_2$(biim)]PPh$_4$(MeOH) where the hydrogens bonds are highlighted.

The MeOH molecule forms a hydrogen bond with one of the nitrogen of the biim^{2-} ligand ($d_{N\cdots H}$ = 1.96 Å; $d_{N\cdots H\text{-}O}$ = 2.78 Å, Table S8). Similarly to complex [Cr(Hbiim)$_3$], one of the Hbiim$^-$ ligand in [Cr(Hbiim)$_2$(biim)]$^-$ forms the same type of complementary hydrogen bonds with a Hbiim$^-$ of an adjacent complex (Figure A2-6a). The second Hbiim$^-$ ligand forms another type of complementary interaction with one of the imidazolate of the biim^{2-} ligand of an adjacent complex (Figure A2-6b). As it can be seen on Figure A2-6b, the N-H of the Hbiim$^-$ forms a hydrogen bond with the N$^-$ of the imidazolate ($d_{N\cdots H}$ = 1.89 Å), while the N$^-$ of the Hbiim$^-$ interacts weakly with the aromatic C-H of the same imidazolate ring of biim^{2-}($d_{N\cdots H}$ = 2.57 Å).

a)

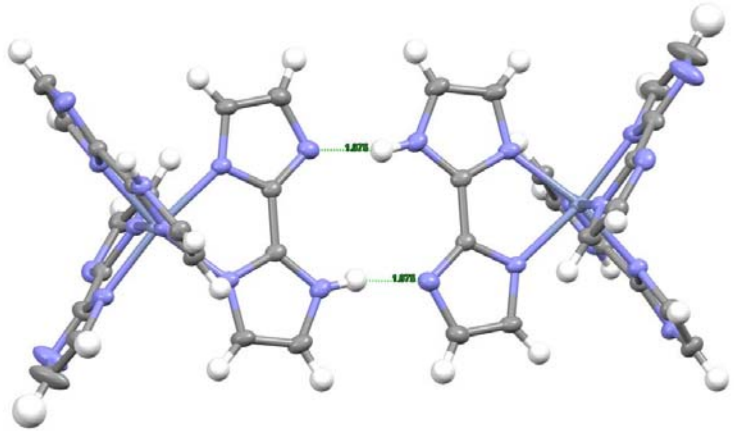

b)

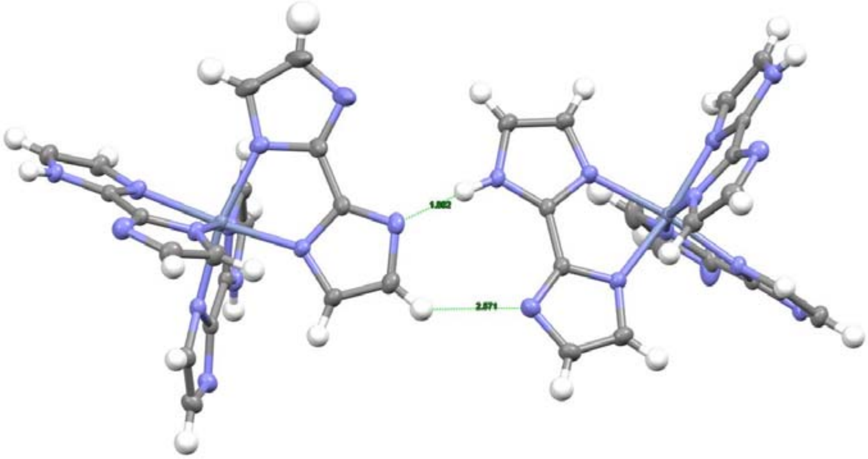

Figure A2-6 a) View of the hydrogen bonds formed between two $Hbiim^-$ ligands of adjacent complexes in the crystal structure of $[Cr(Hbiim)_2(biim)]PPh_4(MeOH)$. b) View of the hydrogen bonds formed between one $Hbiim^-$ ligand and one $biim^{2-}$ of an adjacent complex in the crystal structure of $[Cr(Hbiim)_2(biim)]PPh_4(MeOH)$.

Surprisingly, when the crystalline powder of $[Cr(Hbiim)_2(biim)]PPh_4(MeOH)$ is separated from the solvent mixture and dried, single crystals suitable for X-ray diffraction could be extracted from the powder. The structure of these crystals is essentially the same as the previous one but without the MeOH molecule to give $[Cr(Hbiim)_2(biim)]PPh_4$ **4** (Figure A2-7, Tables S10-S12). The X-ray diffraction powder pattern indicates that the dried powder only contains crystals without MeOH.

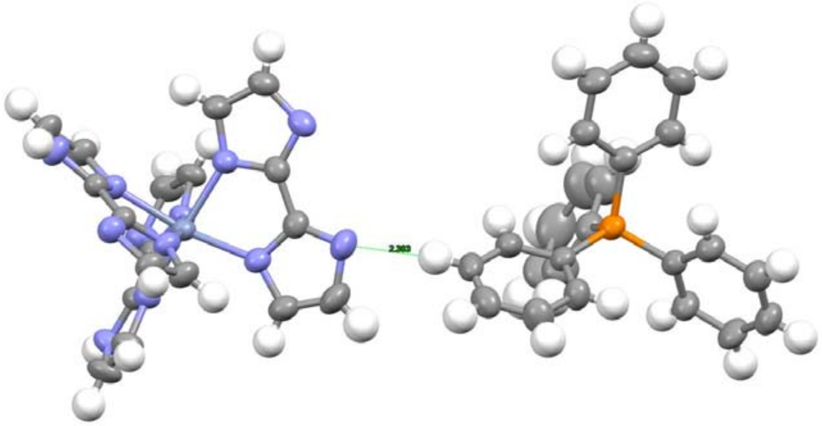

Figure A2-7 View of the asymmetric unit in the crystal structure of dry [Cr(Hbiim)$_2$(biim)]PPh$_4$ where the hydrogen bond between the biim^{2-} and the PPh$_4^+$ cation is highlighted.

Crystal structure of *cis*-[Cr(Me$_2$biim)$_2$Cl$_2$]Cl 5 (Tables S13-S15).

The structure of the intermediate *cis*-[Cr(Me$_2$biim)$_2$Cl$_2$]Cl that was isolated as an intermediate product along the synthesis of [Cr(Me$_2$biim)$_3$](CF$_3$SO$_3$)$_3$ (Figure 2) could also be resolved by X-ray diffraction. Single crystals were obtained by slow evaporation of a concentrated methanolic solution of [Cr(Me$_2$biim)$_2$Cl$_2$]Cl.

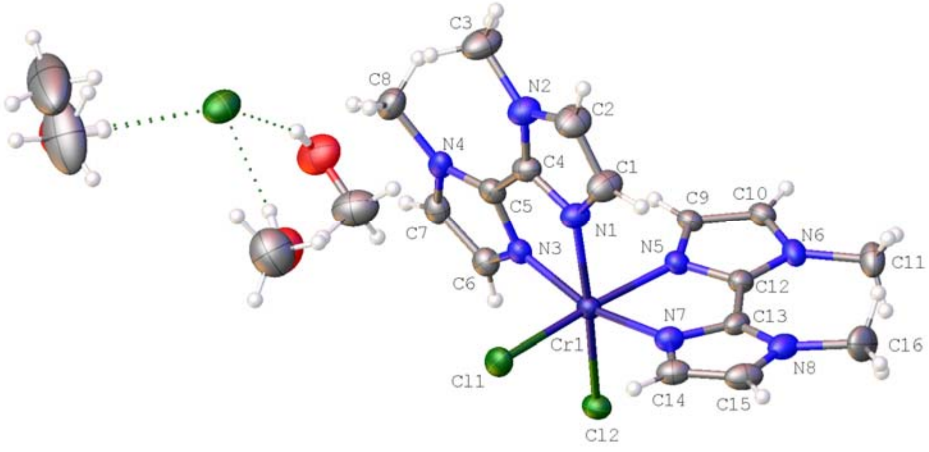

Figure A2-8 ORTEP view of the full asymmetric unit of *cis*-[Cr(Me$_2$biim)$_2$Cl$_2$]Cl(MeOH)$_3$.

In this complex, two Me$_2$biim ligands and two Cl$^-$ are coordinated to the chromium ion, while a third Cl$^-$ lies outside the first coordination sphere and is surrounded by 3 molecules of MeOH (Figure A2-8). The complex adopts the cis-configuration.

Crystal structure of [Cr(Me$_2$biim)$_3$](CF$_3$SO$_3$)$_3$ 6 (Tables S16-S17).

For the complex [Cr(Me$_2$biim)$_3$](CF$_3$SO$_3$)$_3$, single crystals suitable for X-ray diffraction could be obtained by slow evaporation of a methanolic solution of the complex.

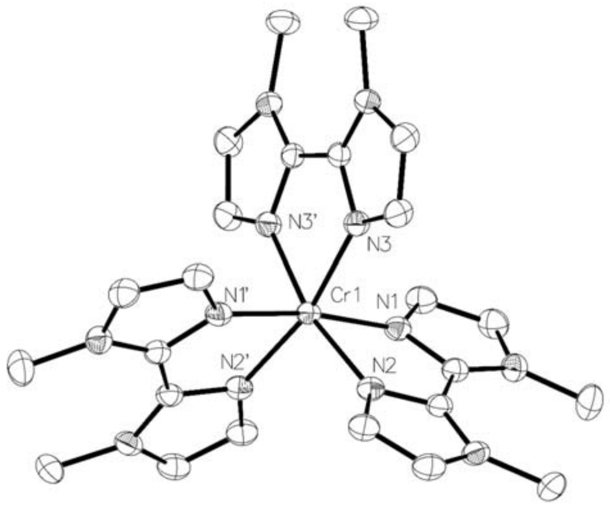

Figure A2-9 ORTEP view of the complex $[Cr(Me_2biim)_3]^{3+}$ in the crystal structure of $[Cr(Me_2biim)_3](CF_3SO_3)_3$. Hydrogen atoms and $CF_3SO_3^-$ are omitted for clarity.

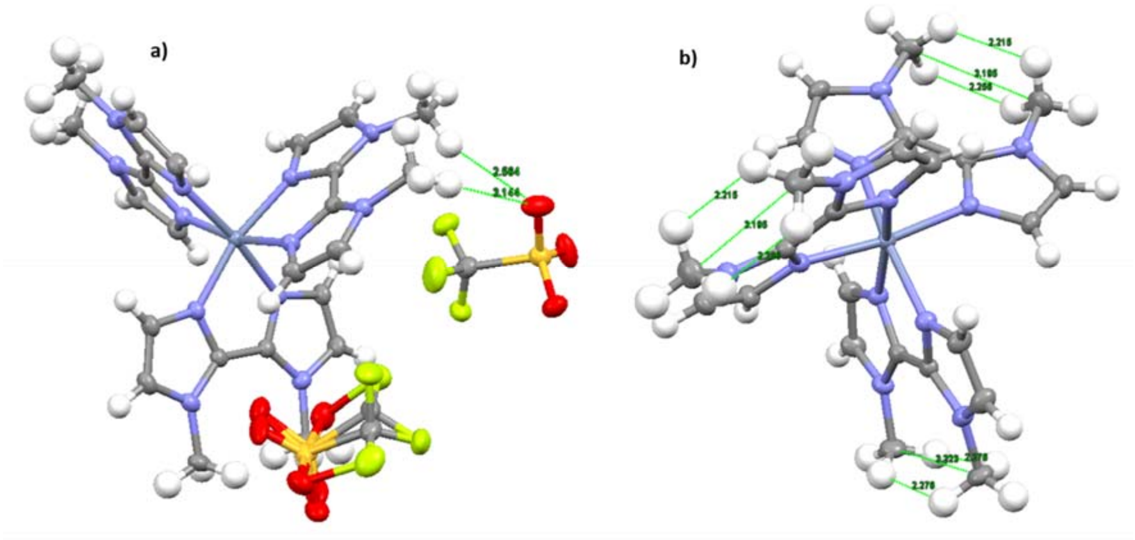

Figure A2-10 a) View of the intermolecular interactions between the triflates and the hydrogens of the methyls. b) View of the intramolecular steric interactions between the methyl groups of Me_2biim.

No molecule of solvent is present in the crystal structure, the triflate anions weakly interact with the hydrogens of the methyl groups of the ligands (Figure A2-10a). Some steric interactions are observed between the adjacent methyl groups of each ligand that are in close proximity ($d_{H\cdots H}$ = 2.2-2.3 Å; $d_{C\cdots C}$ = 3.2 Å) and adopt a conformation that minimize distance between the hydrogen atoms (Figure A2-10b).

Structural analysis

For all the structures presented above, the two imidazole rings in the didentate ligands (H_2biim, $Hbiim^-$, $biim^{2-}$ and Me_2biim), are almost coplanar, with no or little rotation around the C_2-$C_{2'}$ axis

(N_1-C_2-$C_{2'}$-$N_{1'}$ dihedral angles < 7.0°). The central Cr^{3+} ion is surrounded by 6 atoms in the first coordination sphere, with a pseudo-octahedral geometry. The amount of deviation from a perfect octahedral geometry was quantified in three different ways. The first way consists to look at the N-Cr-N' cisoids angles, in a perfect octahedral geometry, the 6 nitrogen atoms around the Cr^{3+} ion would form 12 N-Cr-N' cisoid angles φ_i exactly 90° each. When the geometry deviates from pure octahedral, the cisoid angles deviates from 90°, and this deviation can be measured with the parameter Σ [A2-3] using the following equation A2-1:

$$\boldsymbol{\Sigma} = \sum_{i=1}^{12} |\phi_i - 90| \qquad \text{(A2-1)}$$

In the case of a metal surrounded by 3 didentate ligands, it is possible to calculate the parameter Θ using equation A2-2.

$$\boldsymbol{\Theta} = \sum_{i=1}^{6} |\boldsymbol{\theta}_i - 60| \qquad \text{(A2-2)}$$

The definition of the angles θ_i is described in Figure A2-11. This parameter is a good measure of the trigonal distortion in the case of a complex of a metal surrounded by three didentate ligands. If the geometry of the first coordination sphere is perfectly octahedral, then $\Theta = 0°$ but if the geometry is distorted, $\Theta > 0°$.

The last parameter analyzed is the continuous shape measure[A2-4,A2-5] (abbreviated as CShM) of the first coordination sphere using the software SHAPE.[A2-6] CShM is defined as its distance to an ideal shape, independent of size and orientation. In this case, the CShM was compared to the octahedron. For the first coordination sphere of the complexes that can be approximately described by an octahedron, the coordinates of the 6 atoms are given by their position vectors $\overrightarrow{\boldsymbol{Q_k}}$ (k = 1, 2, ..., 6), whereas the coordinates for the perfect octahedron closest in size and orientation is given by the vectors $\overrightarrow{\boldsymbol{P_k}}$($k$ = 1, 2, ...,6). The distance $S_Q(P)$ of the molecular structure Q to the perfect polyhedron P is then defined as

$$\boldsymbol{S_Q(P)} = \mathbf{min} \frac{\sum_{k=1}^{N} |\overrightarrow{Q_k} - \overrightarrow{P_k}|^2}{\sum_{k=1}^{N} |\overrightarrow{Q_k} - \overrightarrow{Q_0}|^2} \times \mathbf{100} \qquad \text{(A2-3)}$$

where $\overrightarrow{\boldsymbol{Q_0}}$ is the coordinate vector of the geometrical center of the investigated structure. With this equation, we obtain $0 < S_Q(P) < 100$. The closer $S_Q(P)$ is to 0, the closer to a perfect octahedron is the analyzed structure; whereas is the coordination sphere is more distorted, $S_Q(P)$ increases.

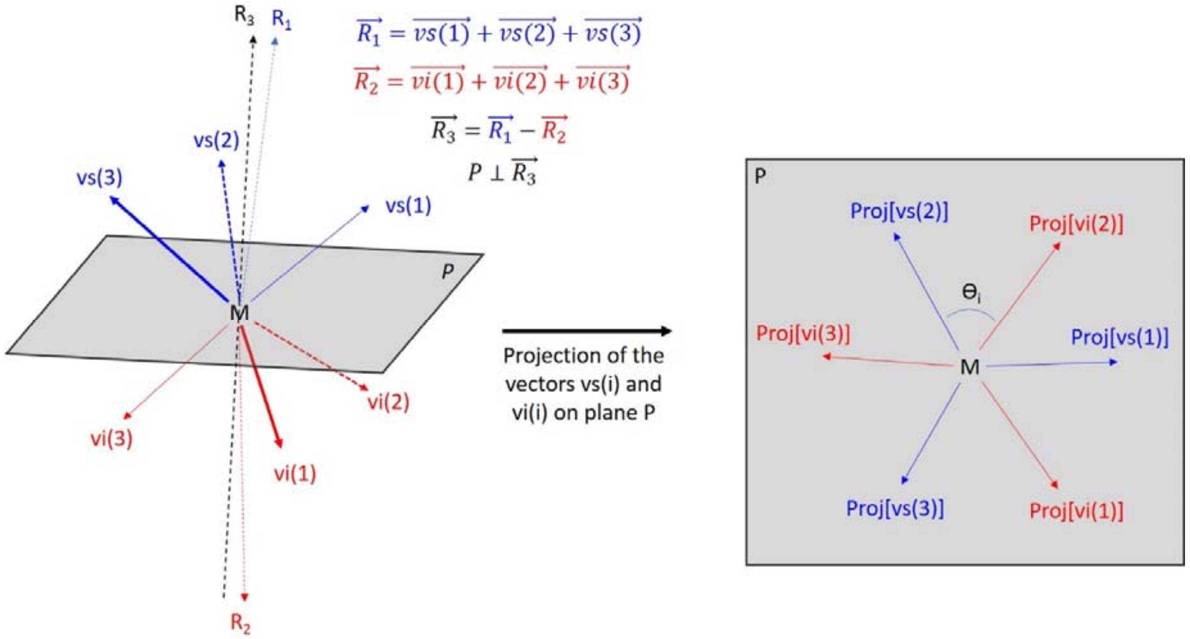

Figure A2-11 Geometrical representation of the angles θ_i used to calculate Θ in the case of a complex $[M(\mathbf{L}_i)_3]^{n+}$. Here, vs(i) and vi(i) corresponds to the position vector relative to M of the two coordinating atoms of each didentate ligand $\mathbf{L}_i$. Two vectors R_1 and R_2 are defined as the sum of vs(i) and vi(i) respectively. A plane P is defined as the plane containing M and perpendicular to the vector $R_3 = R_1 - R_2$ (left part of the figure). Each vector vs(i) and vi(i) is projected on this plane, and 6 angles θ_i are defined as the angles between each pair of adjacent projected vectors Proj[vs(i)] or Proj[vi(i)] (right part of the figure).

Table A2-1 Average Cr-N bond lengths, bite angles and parameters Σ, Θ and $S_Q(P)$ for the complexes reported here and in literature.

Complex	Σ /°	Θ /°	$S_Q(P)$	Average bite angle /°	Average $d_{Cr\text{-}N}$ /Å	Standard deviation of $d_{Cr\text{-}N}$ / Å	Reference
$[Cr(H_2biim)_3](NO_3)_3$	59.2	59.8	0.748	80.4	2.028	0.004	This work
$[Cr(Hbiim)_3](C_6H_6)(H_2O)_2$	66.2	92.6	1.278	79.7	2.039	0.001	A2-1
$[Cr(Hbiim)_2(biim)]PPh_4$	66.6	84.8	1.184	79.7	2.034	0.019	This work
$[Cr(Hbiim)_2(biim)]PPh_4(MeOH)$	66.2	87.0	1.213	79.6	2.037	0.021	This work
$[Cr(Me_2biim)_3](CF_3SO_3)_3$	72.4	80.4	1.195	78.4	2.025	0.005	This work
cis-$[Cr(Me_2biim)_2Cl_2]Cl(MeOH)_3$ [a]	51.6	-	1.289	78.0	2.040	0.012	This work
$[Cr(phen)_2(H_2biim)](CF_3SO_3)_3$	56.5	53.6	0.713	80.9	2.044	0.016	This work
$[Cr(phen)_2(Hbiim)](CF_3SO_3)_2$	57.3	52.1	0.685	80.7	2.047	0.023	This work
$[Cr(phen)_2(biim)](CF_3SO_3)$	58.5	62.2	0.804	80.6	2.046	0.039	This work
$[Cr(bpy)_3](PF_6)_3$	67.4	63.6	0.929	79.1	2.042	0	A2-7
$[Cr(phen)_3](PF_6)_3$ [b]	57.9 [b]	47.5 [b]	0.65 [b]	81.0	2.051	0.008	A2-8

[a] For *cis*-$[Cr(Me_2biim)_2Cl_2]Cl(MeOH)_3$, the positions of the two coordinated Cl atoms were used to calculate Σ and $S_Q(P)$, but not for the bite angle or $d_{Cr\text{-}N}$. [b] Values are averaged for the two complexes Cr1A and Cr1B present in the unit cell.

We can see that for all the complexes in Table A2-1, the average bite angles are very close to 80° (78°< bite angle < 81°). This value is typical of 5-membered chelate ring ligands,[A2-9] and is the main contributor to the deviation from a perfect octahedral geometry. The average Cr-N bond length is also very similar in all complexes with very little variation. When we look at the parameters Σ, Θ and $S_Q(P)$ for the distortion from the perfect octahedral geometry, we can see that $[Cr(Me_2biim)_3]^{3+}$ is the most distorted according to Σ, while *cis*-$[Cr(Me_2biim)_2Cl_2]Cl(MeOH)_3$ has the smallest Σ value, this probably due to the fact that unlike the other complexes, it has only two geometrically constrained didentate ligands and two monodentate Cl^-. However, it has the highest value for $S_Q(P)$ due to the longer Cr-Cl distance compared to the Cr-N distance. Otherwise, $[Cr(phen)_3]^{3+}$ and $[Cr(H_2biim)_3]^{3+}$ appear slightly less distorted than the other homoleptic complexes reported in Table A2-1.

References

A2-1 L. M. Gruia, F. D. Rochon and A. L. Beauchamp, *Can. J. Chem.*, 2006, **84**, 949-959.

A2-2 A. L. Spek, *Acta Cryst. C*, 2015, **71**, 9-18.

A2-3 R. Ketkaew, Y. Tantirungrotechai, P. Harding, G. Chastanet, P. Guionneau, M. Marchivie and D. J. Harding, *Dalton Trans.*, 2021, **50**, 1086-1096.

A2-4 M. Pinsky and D. Avnir, *Inorg. Chem.*, 1998, **37**, 5575-5582.

A2-5 D. Casanova, J. Cirera, M. Llunell, P. Alemany, D. Avnir and S. Alvarez, *J. Am. Chem. Soc.*, 2004, **126**, 1755-1763.

A2-6 Stereochemical Analysis of Molecular Fragments | IQTC - The Institute of Theoretical and Computational Chemistry of the Universitat de Barcelona. https://www.iqtc.ub.edu/uncategorised/program-for-the-stereochemical-analysis-of-molecular-fragments-by-means-of-continous-shape-measures-and-associated-tools/ (accessed 2023-03-17).

A2-7 K. V. Goodwin, W. T. Pennington and J. D. Petersen, *Inorg. Chem.*, 1989, **28**, 2016-2018.

A2-8 B. Doistau, G. Collet, E. Acuna Bolomey, V. Sadat-Noorbakhsh, C. Besnard and C. Piguet, *Inorg. Chem.*, 2018, **57**, 14362-14373.

A2-9 S. Otto, M. Dorn, C. Förster, M. Bauer, M. Seitz and K. Heinze, *Coord. Chem. Rev.*, 2018, **359**, 102-111.

Appendix 3 Crystal structures of heteroleptic chromium complexes with 2,2'biimidazole.

Crystal structure of [Cr(phen)$_2$(H$_2$biim)](CF$_3$SO$_3$)$_3$(H$_2$O)$_{0.25}$ 7 (Tables S18-S20).

Single crystal of [Cr(phen)$_2$(H$_2$biim)](CF$_3$SO$_3$)$_3$(H$_2$O)$_{0.25}$ suitable for X-ray diffraction were obtained by vapor diffusion of Et$_2$O into a solution of the complex in acetonitrile.

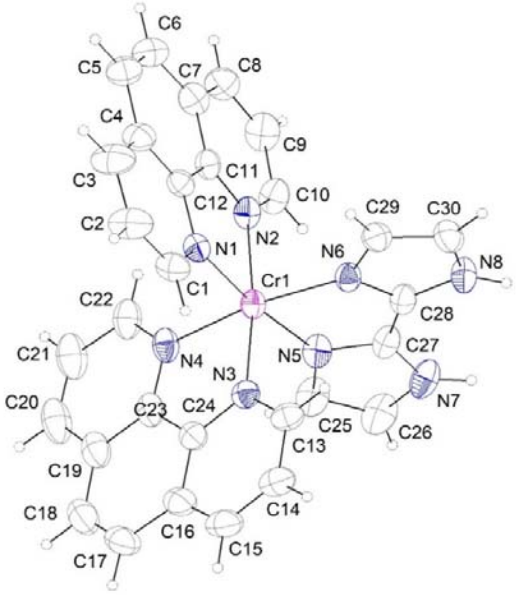

Figure A3-1 Ortep view of [Cr(phen)$_2$(H$_2$biim)](CF$_3$SO$_3$)$_3$(H$_2$O)$_{0.25}$ (thermal ellipsoids are drawn at 50% probability level) with numbering scheme. Triflate counter ions and water molecules are omitted for clarity.

In this complex, Cr atom is surrounded by 6 N atoms in the first coordination sphere, which are arranged in a pseudo-octahedral geometry. The distortion from ideal octahedral geometry is discussed later. The N-H groups form hydrogen bonds with the oxygen atoms of the triflate anions (Table S19). We found a disordered water molecule in the crystal structure (site occupancy ~ 0.25), the oxygen atom of which works as an acceptor for hydrogen bonding with a N-H unit of the complex. This is a clear indication of the acidic nature of the biimidazole-NH groups when the ligand is coordinated to the Cr^{3+} ion.

Crystal structure of [Cr(phen)$_2$(Hbiim)](CF$_3$SO$_3$)$_2$·(H$_2$O)$_{0.5}$ 8 (Tables S21-S23).

The crystal structure of [Cr(phen)$_2$(Hbiim)](CF$_3$SO$_3$)$_2$(H$_2$O)$_{0.5}$ was obtained by adding of one equivalent of NaOH into a concentrated aqueous solution of complex [Cr(phen)$_2$(H$_2$biim)](CF$_3$SO$_3$)$_3$ (c = 10^{-2} mol.L^{-1}). After the addition, the solution was placed at 4 °C and single crystals of [Cr(phen)$_2$(Hbiim)](CF$_3$SO$_3$)$_2$(H$_2$O)$_{0.5}$ suitable for X-ray diffraction appeared after a few minutes.

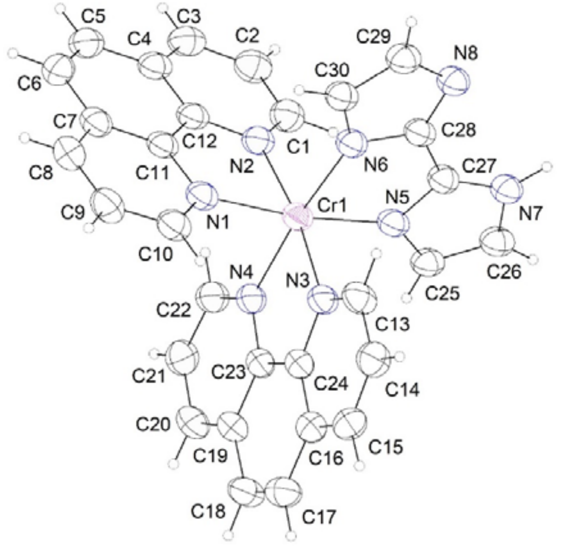

Figure A3-2 Ortep view of $[Cr(phen)_2(Hbiim)](CF_3SO_3)_2(H_2O)_{0.5}$ (thermal ellipsoids are drawn at 50% probability level) with numbering scheme. Triflate counter ions and water molecules are omitted for clarity.

There are two triflates in the asymetric unit (in agreement with the protonation of the ligand and the valence state of the Cr^{3+}). One triflate is disordered over two sites and modeled in two parts with refined occupancies 0.73/0.27. A water molecule is found close to this disordered triflate and was refined with occupancy fixed at 0.5. This water molecule could be involved in hydrogen bonding with the ordered triflate anion.

As it was observed for other complexes containing a $Hbiim^-$ ligand such as $[Cr(Hbiim)_3]$, there are intermolecular hydrogen bonds between two adjacent complexes through N-H···N bonds (Figure A3-3). The N···H and the N···H-N distances are short (1.90 Å and 2.76 Å respectively, Table S22) showing strong interactions. This was also shown in the IR spectra of the complexes were the N-H stretching frequency is shifted toward lower energy compared with that observed for a typical free N-H bond (Figure S57, red trace).

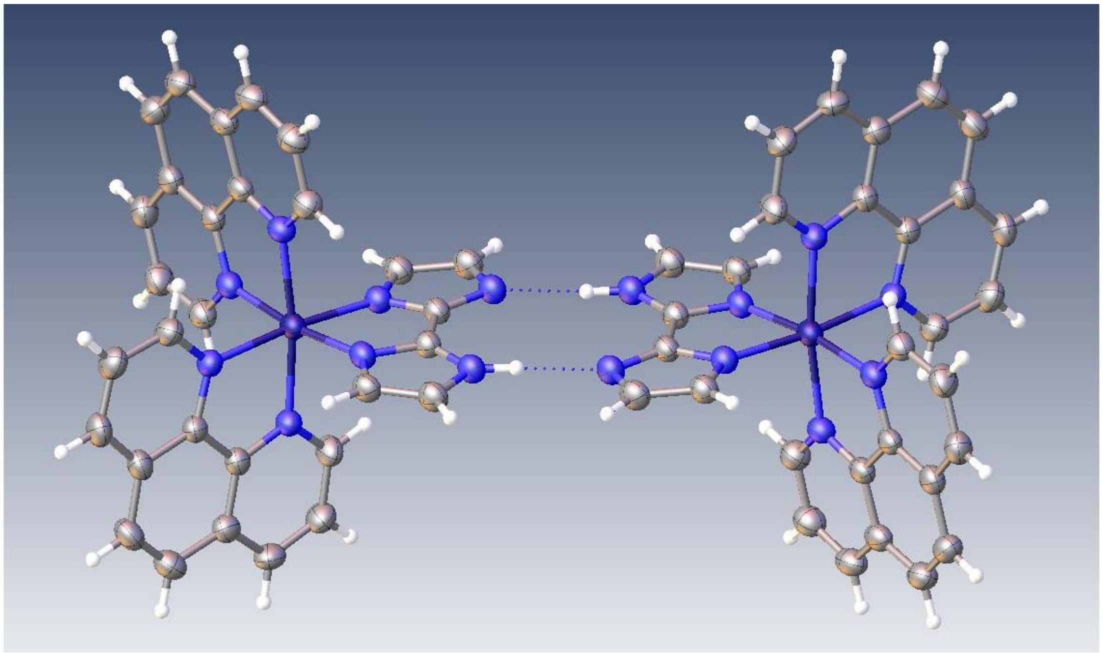

Figure A3-3 Intermolecular H-bonds found between two adjacent complexes in the crystal structure of $[Cr(phen)_2(Hbiim)](CF_3SO_3)_2(H_2O)_{0.5}$.

Crystal structure of $[Cr(phen)_2(biim)]CF_3SO_3(CH_3OH)_{1.5}$ (Table S24-S26).

Single crystal of $[Cr(phen)_2(biim)]CF_3SO_3(CH_3OH)_{1.5}$ suitable for X-ray diffraction were obtained by vapor diffusion of Et_2O into a solution of the complex in methanol.

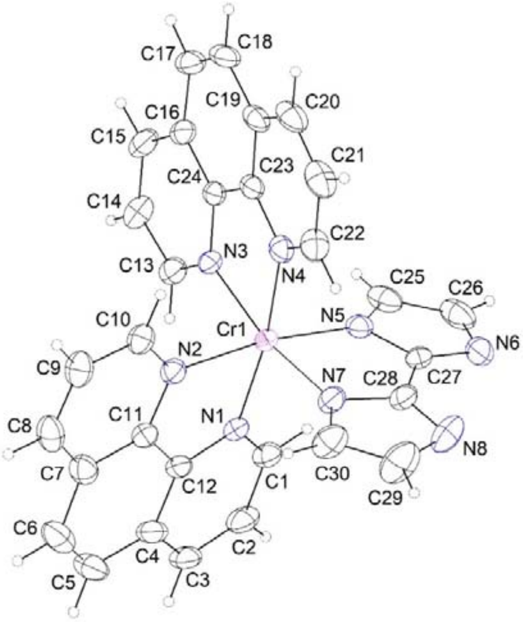

Figure A3-4 Ortep view of $[Cr(phen)_2(biim)]CF_3SO_3(CH_3OH)_{1.5}$ (thermal ellipsoids are drawn at 50% probability level) with numbering scheme. Triflate counter ions and methanol molecules are omitted for clarity.

The complex crystalizes in the triclinic P -1 space group with one triflate counterion and MeOH solvent molecules. One MeOH is fully ordered and makes H-bond with the triflate ion (Table S25). Another MeOH is disordered and refined with fixed 0.5 occupancy and with isotropic ADP.

Structural analysis

For the cationic $[Cr(phen)_2(H_x biim)]^{(1+x)+}$ complexes (x = 0-2), the average N-Cr-N bite angles of the ligands lie between 80 and 81°, a value typical of Cr(III) complexes with 5-membered chelate rings,[23] which is the main contributor to the deviation from a perfect octahedral geometry. When we look at the parameters Σ, Θ and $S_Q(P)$ for the distortion from the perfect octahedral geometry, we notice that the distortion is almost identical between these three complexes. In addition, they display similar distortion as their homoleptic parent complexes $[Cr(phen)_3]^{3+}$ and $[Cr(H_2biim)_3]^{3+}$ and are slightly less distorted than $[Cr(bpy)_3]^{3+}$, $[Cr(Hbiim)_3]$, $[Cr(Hbiim)_2(biim)]^-$ and $[Cr(Me_2biim)_3]^{3+}$ (Table A2-1). These small differences are weak compared to the difference in distortion between complexes with 6-membered and 5-membered chelate rings (Table A2-1).

Appendix 4 Theoretical calculations of the electronic structures of heteroleptic complexes $[Cr(phen)_2(H_x biim)]^{(1+x)+}$.

Theoretical studies

The Orca[A4-1] (version 5.0.2) software package was used to investigate the structural and electronic properties of the studied complexes. Starting from the X-ray diffraction structures, the ground, $^4A_2$ state, structure of the three complexes were obtained from DFT optimizations using the Becke three-parameters exchange function in combination with the Lee-Yang-Parr correlation functional (B3LYP). The Ahlrichs' polarized valence triple-ζ basis set def2-TZVPP was used for these optimizations. The D3 version of Grimme's dispersion with Becke-Johnson damping (GD3BJ) was applied. Solvent effects were included via the Conductor-like Polarizable Continuum Model (CPCM) as implemented in Orca 5.0.2 with the dielectric constant of acetonitrile. Optimized geometries were confirmed to be stationary points by analysis of their vibrational frequencies. Tight convergence criteria were selected for the optimization step. The resolution of identity approach for the Coulomb term in combination with the chain-of-spheres approximation for the exchange term (RIJCOSX) was applied. The zero-order relativistic approximation (ZORA) was used to describe relativistic effects in all calculations. Spin density information was extracted from the optimized geometries.

Ab initio ligand field (AILF) analysis was performed over the optimized geometries using Orca version 5.0.2. The complete-active-space self-consistent field method (CASSCF) together with the fully internally contracted N-electron valence perturbation theory to second order (FIC-NEVPT2) was used, selecting only the 3d orbitals as active space (CASSCF(3,5)/FIC-NEVPT2) and using the def2-TZVPP basis set in combination with the RI-JK approximation (def2/JK as auxiliary base). 10 quartet and 40 doublet roots were computed for the AILF analysis.

Subsequently, the active space was expanded to accurately model the ligand field. Dominant bonding/antibonding orbitals formed between ligand and chromium and a second d shell were considered, creating an active space of 7 electrons and 12 orbitals (CASSCF(7,12)/FIC-NEVPT2). 10 quartet and 9 doublet roots were computed to calculate the energies of the excited states.

The 200 lowest energetic transitions (100 for $[Cr(phen)_2(H_2biim)]^{3+}$) were calculated by TD-DFT using the Tamm-Dancoff approximation (TDA) as implemented in Orca 5.0.2, using the B3LYP functional, selecting the def2-TZVPP basis set and considering solvent effects. Charge transfer numbers were computed using TheoDORE 3.1.1.[A4-2]

Optimized geometries

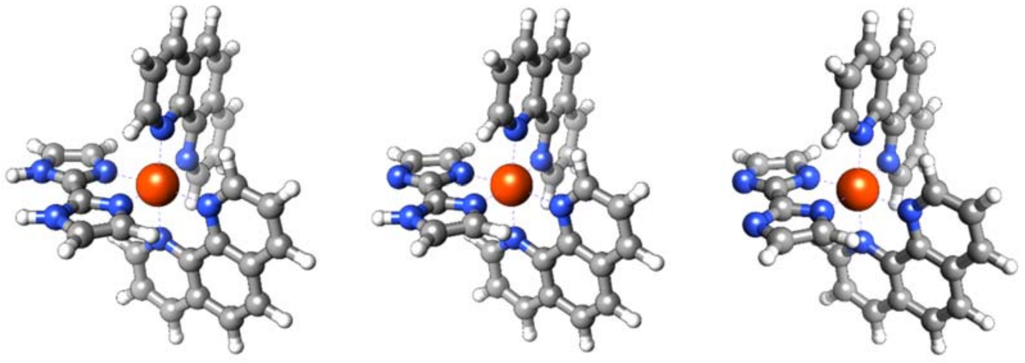

$[Cr(phen)_2(H_2biim)]^{3+}$ (2.935689) $[Cr(phen)_2(Hbiim)]^{2+}$ (2.917929) $[Cr(phen)_2(biim)]^{+}$ (2.895396)

Figure A4-1 DFT optimized geometries of the $[Cr(phen)_2(H_2biim)]^{3+}$, $[Cr(phen)_2(Hbiim)]^{2+}$ and $[Cr(phen)_2(biim)]^{+}$ species, and spin density at the Cr center (in brackets).

Ab initio ligand field analysis

Table A4-1 AILF parameters computed from CASSCF(3,5)/FIC-NEVPT2 (in cm^{-1}).

	B	*C*	*C/B*
$[Cr(phen)_2(H_2biim)]^{3+}$	978	3018	3.086
$[Cr(phen)_2(Hbiim)]^{2+}$	973	2988	3.070
$[Cr(phen)_2(biim)]^{+}$	964	2980	3.092

Complete active space analysis of excited states

Table A4-2 CASSCF(7,12)/FIC-NEVPT2 results. Energies in cm^{-1}.

	^2E (1)	^2E (2)	$^2T_1$ (1)	$^2T_1$ (2)	$^2T_1$ (3)	$^2T_2$ (1)	$^2T_2$ (2)	$^2T_2$ (3)	$^4T_2$ (1)	$^4T_2$ (2)	$^4T_2$ (3)
$[Cr(phen)_2(H_2biim)]^{3+}$	15526	15953	15547	16066	16130	23400	23540	23722	23266	23317	23383
$[Cr(phen)_2(Hbiim)]^{2+}$	15397	15425	15785	15831	15970	23251	23407	23510	22356	22365	24493
$[Cr(phen)_2(biim)]^{+}$	15197	15653	15086	15574	15632	22766	23037	23052	21307	21331	25909

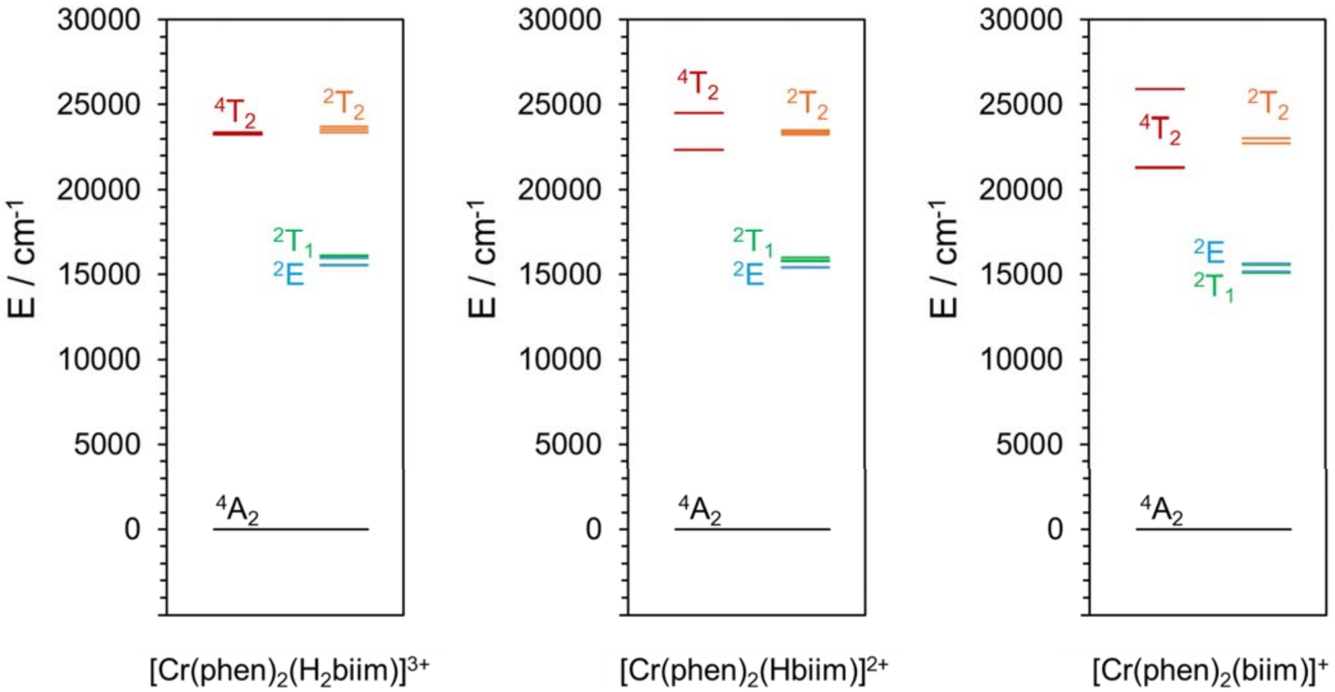

Table A4-3 Orbitals used in the CASSCF(7,12)/FIC-NEVPT2 calculations for $[Cr(phen)_2(H_2biim)]^{3+}$.

#	E (Hartrees)	Orbital	#	E (Hartrees)	Orbital
136	-0.891320	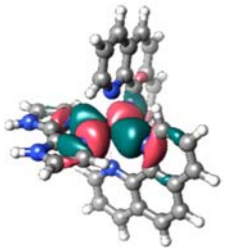	142	-0.203890	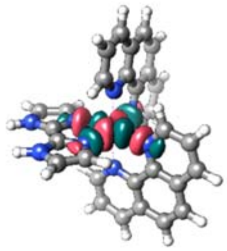
137	-0.884149	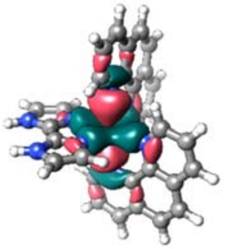	143	0.698149	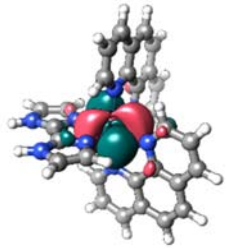
138	-0.420341	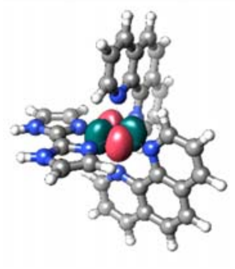	144	0.700649	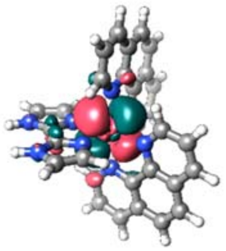
139	-0.419759	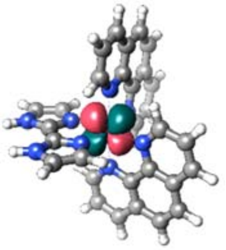	145	0.756566	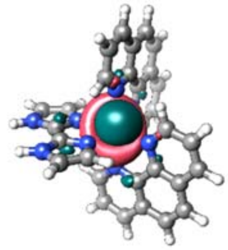
140	-0.418668	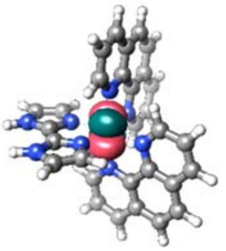	146	1.366547	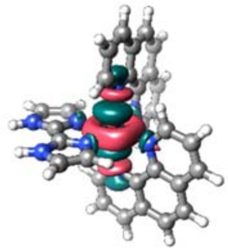

141	-0.205857	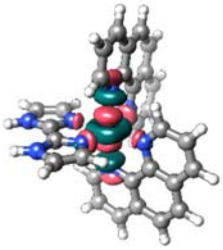	147	1.380778	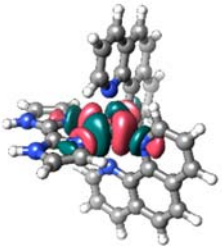

Table A4-4 Orbitals used in the CASSCF(7,12)/FIC-NEVPT2 calculations for $[Cr(phen)_2(Hbiim)]^{2+}$.

#	E (Hartrees)	Orbital	#	E (Hartrees)	Orbital
136	-0.779656	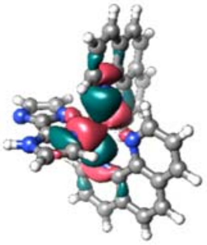	142	-0.081906	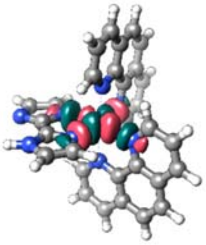
137	-0.764893	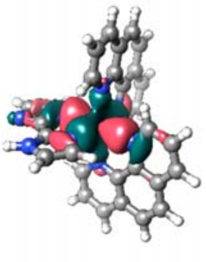	143	0.795968	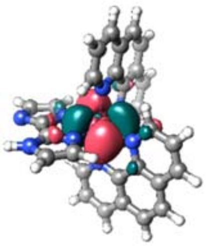
138	-0.309476	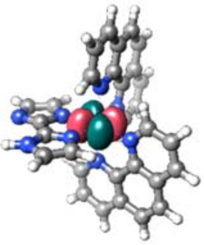	144	0.823302	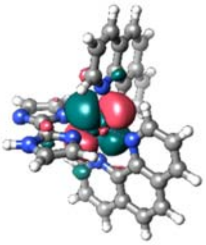
139	-0.301398	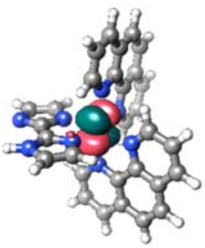	145	0.863583	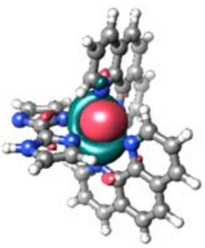

140	-0.297620	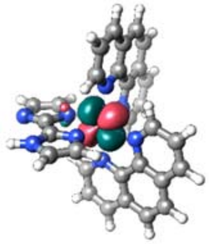	146	1.476010	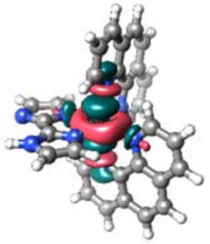
141	-0.094737	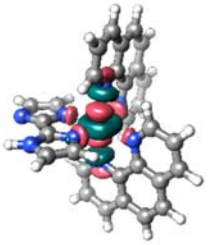	147	1.505554	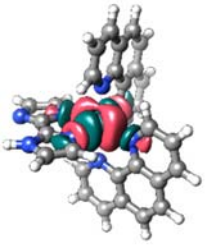

Table A4-5 Orbitals used in the CASSCF(7,12)/FIC-NEVPT2 calculations for $[Cr(phen)_2(biim)]^+$.

#	E (Hartrees)	Orbital	#	E (Hartrees)	Orbital
136	-0.661291	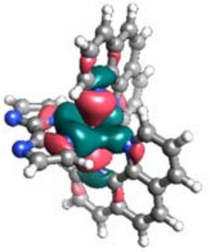	142	0.040646	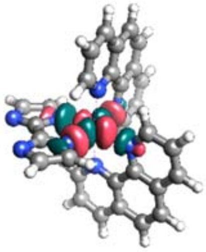
137	-0.632695	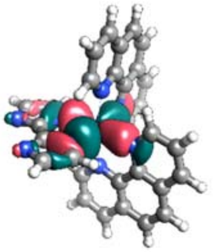	143	0.879064	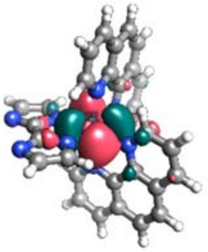
138	-0.194363	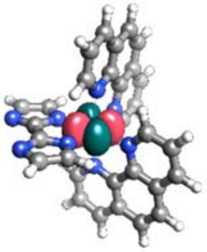	144	0.931572	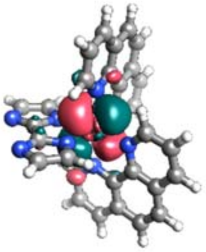

139	-0.177920	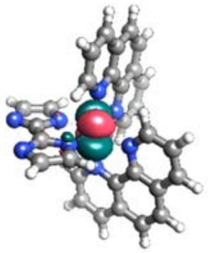	145	0.959369	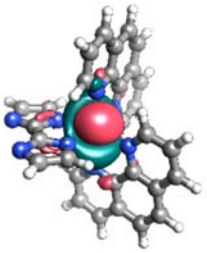
140	-0.177153	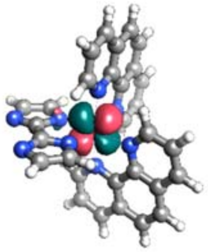	146	1.578649	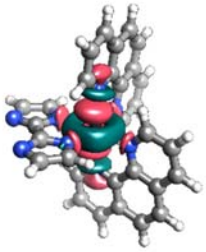
141	0.019769	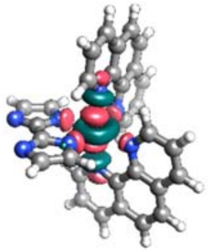	147	1.643522	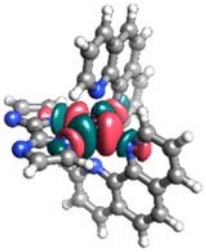

Electronic transitions

Table A4-6 Calculated 100 lowest electronic transitions for compound $[Cr(phen)_2(H_2biim)]^{3+}$, their energies (in nm) and oscillatory strength (in cgs units).

Transition number	Wavelength / nm	Osc. Strength / cgs	Transition number	Wavelength / nm	Osc. Strength / cgs
1	443.6	0.008684030	51	280.8	0.000452941
2	439.6	0.004200170	52	280.5	0.048391800
3	412.9	0.000003947	53	277.6	0.037550200
4	408.6	0.000502561	54	276.9	0.002606690
5	408.1	0.000539178	55	276.6	0.004495860
6	407.3	0.003104320	56	275.8	0.004360860
7	400.0	0.000081510	57	272.2	0.000053585
8	392.1	0.000468006	58	271.7	0.000138697
9	391.5	0.000372989	59	271.5	0.006154400
10	371.8	0.007424990	60	270.8	0.000433198
11	370.2	0.000681299	61	268.9	0.002043610
12	369.9	0.002925900	62	268.8	0.003389300
13	365.5	0.000626678	63	268.5	0.134595000
14	364.0	0.009016750	64	267.7	0.206803000
15	361.4	0.000112825	65	267.4	0.007183370
16	352.1	0.000090076	66	266.9	0.013824500
17	351.7	0.006467370	67	266.7	0.013959400
18	351.2	0.001258590	68	264.5	0.032832100
19	346.4	0.000270970	69	264.1	0.017612800
20	344.2	0.000002756	70	262.6	0.156393000
21	337.8	0.000625391	71	262.1	0.055324000
22	336.1	0.044363800	72	260.6	0.337505000
23	333.7	0.006406020	73	260.1	0.006237730
24	330.1	0.006118370	74	260.0	0.000077023
25	329.4	0.002729690	75	258.7	0.026949900
26	328.7	0.016821800	76	257.9	0.005224130
27	326.1	0.005594130	77	256.7	0.023204100
28	317.0	0.000495467	78	256.6	0.086377700
29	316.9	0.007398730	79	255.1	0.006831260
30	315.4	0.001657430	80	254.5	0.008499690
31	313.5	0.029874000	81	254.4	0.022430900
32	312.3	0.000800702	82	254.1	0.012325700
33	310.8	0.000979412	83	253.8	0.000202867
34	310.5	0.000389834	84	252.7	0.012082700

35	310.2	0.000124672	85	252.5	0.061711300
36	307.4	0.000016240	86	252.1	0.001091380
37	304.4	0.000410148	87	250.5	0.007870940
38	303.6	0.018642900	88	250.0	0.062026400
39	303.3	0.019057600	89	249.9	0.002682560
40	301.0	0.000372178	90	248.9	0.000626233
41	297.4	0.014590400	91	248.6	0.001237280
42	297.1	0.035206300	92	247.5	0.001268180
43	289.5	0.010439100	93	247.2	0.017915500
44	288.6	0.001388210	94	245.9	0.017769900
45	285.9	0.000430932	95	245.7	0.004281680
46	285.7	0.000730861	96	245.6	0.024821500
47	283.7	0.010229600	97	245.3	0.001892550
48	283.3	0.063454700	98	242.9	0.002968190
49	282.6	0.005133940	99	242.5	0.006601790
50	282.2	0.002848400	100	242.2	0.004056090

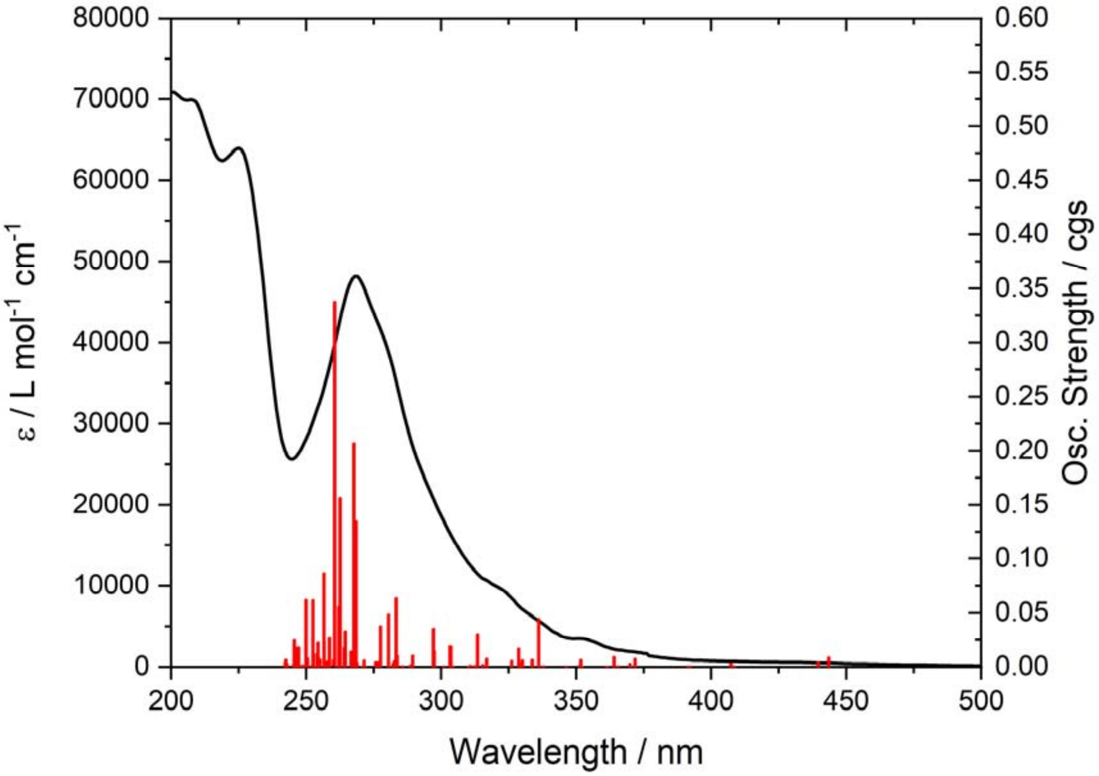

Figure A4-2 Experimental UV-Vis spectrum of compound $[Cr(phen)_2(H_2biim)]^{3+}$ in acetonitrile and calculated oscillatory strength of the 100 lowest energetic electronic transitions.

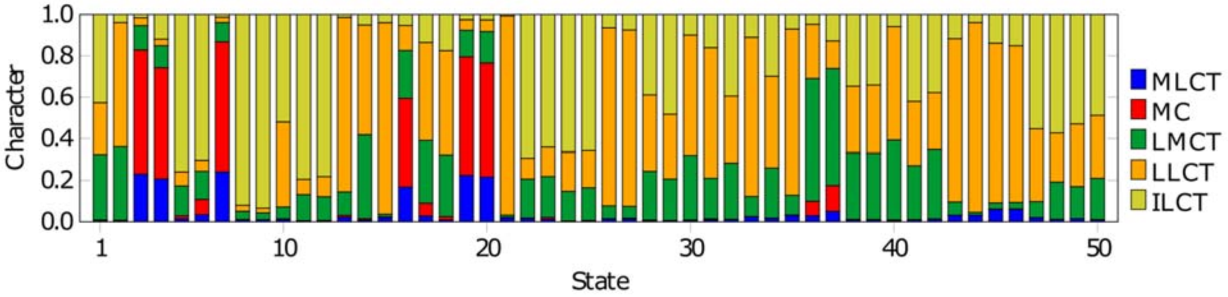

Figure A4-3 TD-DFT charge transfer numbers of $[Cr(phen)_2(H_2biim)]^{3+}$ defined from 0 to 1 of the first 50 electronic transitions.

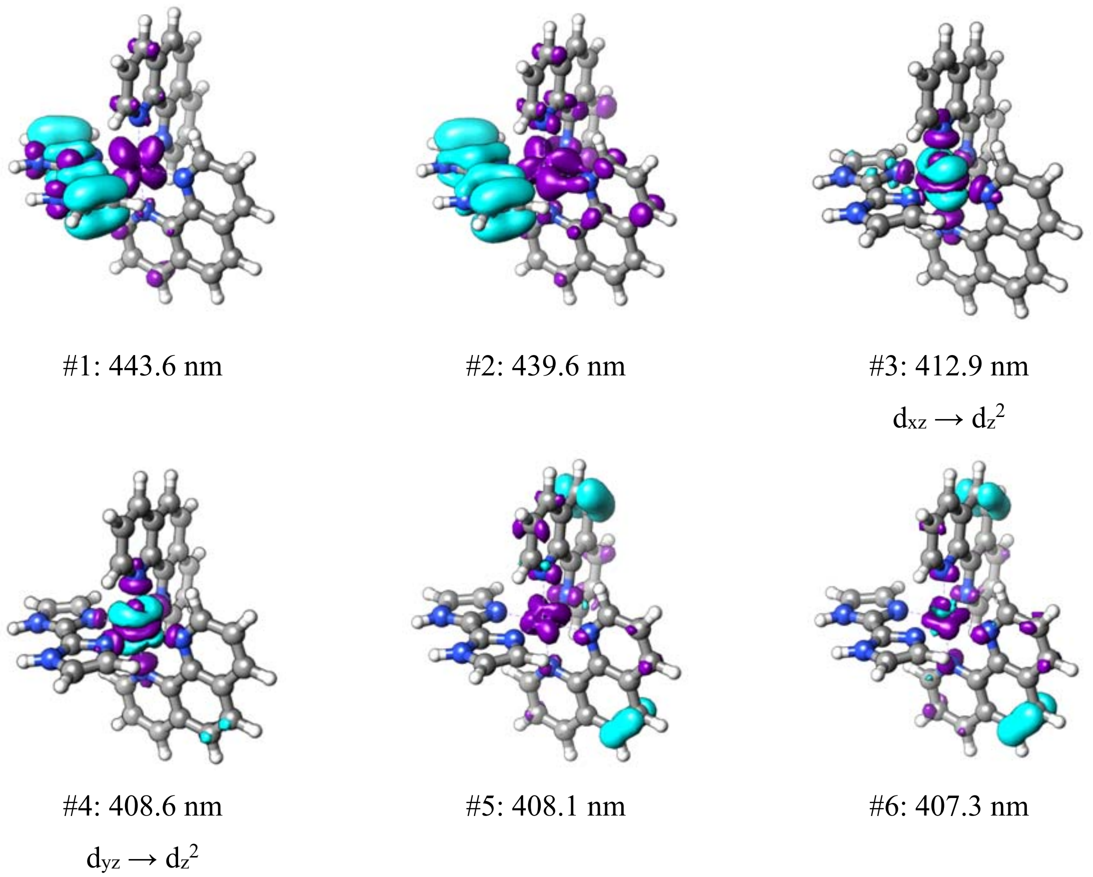

Figure A4-4 Electron density difference maps (EDDM) of the first six electronic transitions calculated for $[Cr(phen)_2(H_2biim)]^{3+}$ (isoval 0.002). Blue: density loss; Purple: density gain.

Table A4-7 Calculated 100 lowest electronic transitions for compound $[Cr(phen)_2(Hbiim)]^{2+}$, their energies (in nm) and oscillatory strength (in cgs units).

Transition number	Wavelength / nm	Osc. Strength / cgs	Transition number	Wavelength / nm	Osc. Strength / cgs
1	566.4	0.004596640	51	291.2	0.004050420
2	534.3	0.005268420	52	289.4	0.009219620
3	469.4	0.000118195	53	288.1	0.002303550
4	463.1	0.000141174	54	287.0	0.007927360
5	447.4	0.006781430	55	286.3	0.008708070
6	434.0	0.000048876	56	286.1	0.001068170
7	427.3	0.000980469	57	284.2	0.003812250
8	420.7	0.000088355	58	283.9	0.024528600
9	416.6	0.000627904	59	281.4	0.023803000
10	412.1	0.001962710	60	280.3	0.046702100
11	398.8	0.000451316	61	279.1	0.011031800
12	398.1	0.001755930	62	278.1	0.000094897
13	394.5	0.000434629	63	277.1	0.015710900
14	390.5	0.000081918	64	275.9	0.059112800
15	389.7	0.000614485	65	275.0	0.048348900
16	389.4	0.000821501	66	274.3	0.066134600
17	377.6	0.004545730	67	273.9	0.104217000
18	372.9	0.000872146	68	273.5	0.058007600
19	363.9	0.000932181	69	272.9	0.045260800
20	363.8	0.001636730	70	271.0	0.072973600
21	357.2	0.001609420	71	269.7	0.018385800
22	353.3	0.000130741	72	269.5	0.016121000
23	348.4	0.000377243	73	268.5	0.004174580
24	345.0	0.017727500	74	267.6	0.005838330
25	339.4	0.007911210	75	267.1	0.004906950
26	338.7	0.009075230	76	266.6	0.065096800
27	337.5	0.001279740	77	265.9	0.012984400
28	327.8	0.035997000	78	265.1	0.006392850
29	325.8	0.002736710	79	264.8	0.003847310
30	325.7	0.003508300	80	263.8	0.002875360
31	324.8	0.008019030	81	263.2	0.031525300
32	318.4	0.000112590	82	262.9	0.008410900
33	313.3	0.002812290	83	262.3	0.004274560
34	312.4	0.002506810	84	261.7	0.008883700
35	311.1	0.001411970	85	261.4	0.053049400

36	309.5	0.006370950	86	260.8	0.019480900
37	308.9	0.001882520	87	260.7	0.135591000
38	308.4	0.000651465	88	260.2	0.077877700
39	306.1	0.001157850	89	259.9	0.039955200
40	305.5	0.010225100	90	259.2	0.004400340
41	303.1	0.002825680	91	259.0	0.006065760
42	302.2	0.001841950	92	258.4	0.027450100
43	302.0	0.000410570	93	257.9	0.172672000
44	301.3	0.006216080	94	257.1	0.005134770
45	300.0	0.006261650	95	256.8	0.115770000
46	296.3	0.004163640	96	255.8	0.010901800
47	295.2	0.001886810	97	254.8	0.000557923
48	295.0	0.001080160	98	253.9	0.001951630
49	294.0	0.007871320	99	253.6	0.002959890
50	293.6	0.011954100	100	252.9	0.010650700

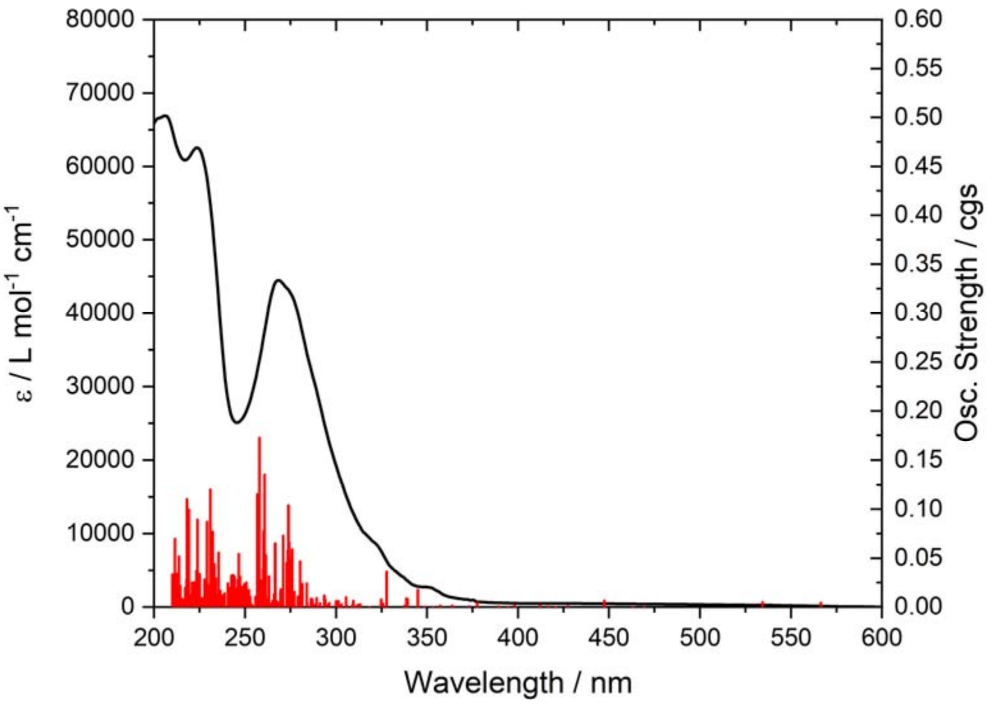

Figure A4-5 Experimental UV-Vis spectrum of compound [Cr(phen)$_2$(Hbiim)]$^{2+}$ in acetonitrile and calculated oscillatory strength of the 200 lowest energetic electronic transitions.

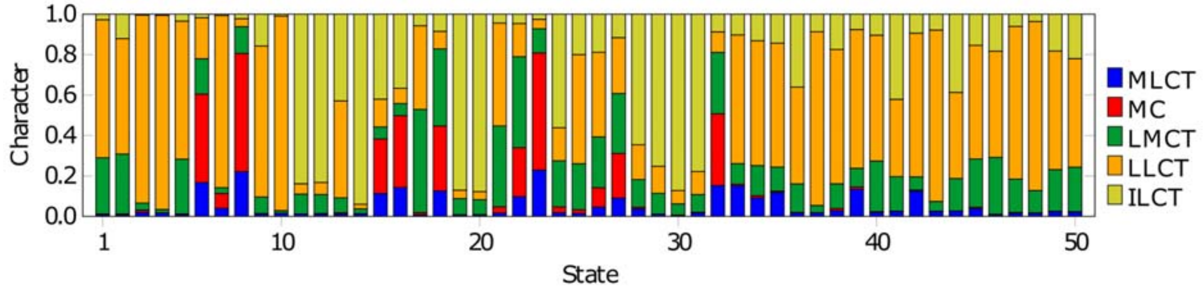

Figure A4-6 TD-DFT charge transfer numbers of $[Cr(phen)_2(Hbiim)]^{2+}$ defined from 0 to 1 of the first 50 electronic transitions.

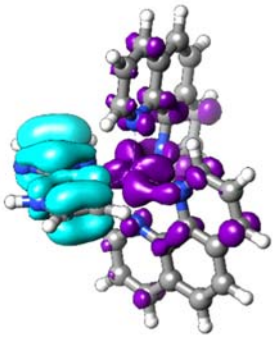

#1: 566.4 nm

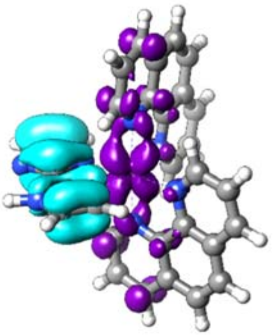

#2: 534.3 nm

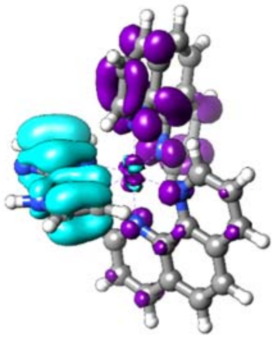

#3: 469.4 nm

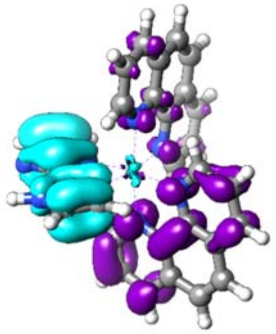

#4: 463.1 nm

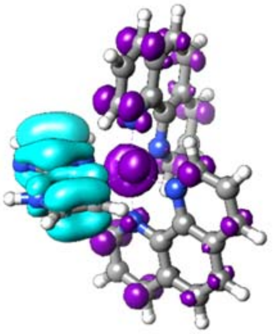

#5: 447.4 nm

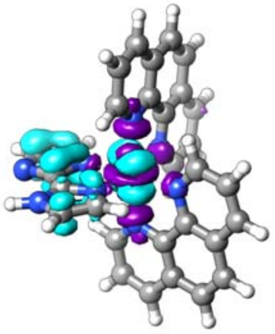

#6: 434.0 nm

$d_{xz} \rightarrow d_{z^2}$

Figure A4-7 Electron density difference maps (EDDM) of the first six electronic transitions calculated for $[Cr(phen)_2(Hbiim)]^{2+}$ (isoval 0.002). Blue: density loss; Purple: density gain.

Table A4-8 Calculated 100 lowest electronic transitions for compound $[Cr(phen)_2(biim)]^+$, their energies (in nm) and oscillatory strength (in cgs units).

Transition number	Wavelength / nm	Osc. Strength / cgs	Transition number	Wavelength / nm	Osc. Strength / cgs
1	743.2	0.005162030	51	312.6	0.001339400
2	672.4	0.003632770	52	312.2	0.001045720
3	608.5	0.000180943	53	309.8	0.003239630
4	598.7	0.000107971	54	306.2	0.000508441
5	559.5	0.004714040	55	305.7	0.003287360
6	545.9	0.001512890	56	304.2	0.000308149
7	522.8	0.000707693	57	303.8	0.012285100
8	515.3	0.001252780	58	303.4	0.005968410
9	457.8	0.000112575	59	303.3	0.000710819
10	448.8	0.001659430	60	302.9	0.000030120
11	435.7	0.000105183	61	300.7	0.009417540
12	431.8	0.004900050	62	300.0	0.010808800
13	414.5	0.001801360	63	297.5	0.000137734
14	393.2	0.000702125	64	296.6	0.000075741
15	392.8	0.000508584	65	295.9	0.003307420
16	390.9	0.000503117	66	295.2	0.000083379
17	390.7	0.000249496	67	294.5	0.004039320
18	388.8	0.000435071	68	294.2	0.000335155
19	377.9	0.000198224	69	293.1	0.009109290
20	377.7	0.000287311	70	292.9	0.000052618
21	367.3	0.000924150	71	292.3	0.007644430
22	365.7	0.001221750	72	289.0	0.000998633
23	360.4	0.000741620	73	287.3	0.009351400
24	360.0	0.001260070	74	287.1	0.008619100
25	356.2	0.000362403	75	286.9	0.002418320
26	355.8	0.000063135	76	286.1	0.001830420
27	348.8	0.003068860	77	285.3	0.015232700
28	347.2	0.000069999	78	285.0	0.007694390
29	346.9	0.000095189	79	284.6	0.001191160
30	344.5	0.006339210	80	283.8	0.005602730
31	339.4	0.015360100	81	282.6	0.000136658
32	337.4	0.001710730	82	280.6	0.000034403
33	335.6	0.017587200	83	280.4	0.004017230
34	332.0	0.005794030	84	279.8	0.005845910
35	331.2	0.001259990	85	279.4	0.001801960

36	330.1	0.009961350	86	278.5	0.005848540
37	329.3	0.001857910	87	277.5	0.001645100
38	325.3	0.000258103	88	277.2	0.029356300
39	324.0	0.000550063	89	276.9	0.005350750
40	323.2	0.001991950	90	275.0	0.023024100
41	322.6	0.008015820	91	274.8	0.029808000
42	320.0	0.004326270	92	273.0	0.046684200
43	319.3	0.007604530	93	272.8	0.007550800
44	319.2	0.000530403	94	272.3	0.011162500
45	318.9	0.002751250	95	271.7	0.138661000
46	317.9	0.005204030	96	271.2	0.008516900
47	317.0	0.000473341	97	271.1	0.013172000
48	316.7	0.001168860	98	270.3	0.094301200
49	316.6	0.002729880	99	269.0	0.059184700
50	315.9	0.003529530	100	267.4	0.001624330

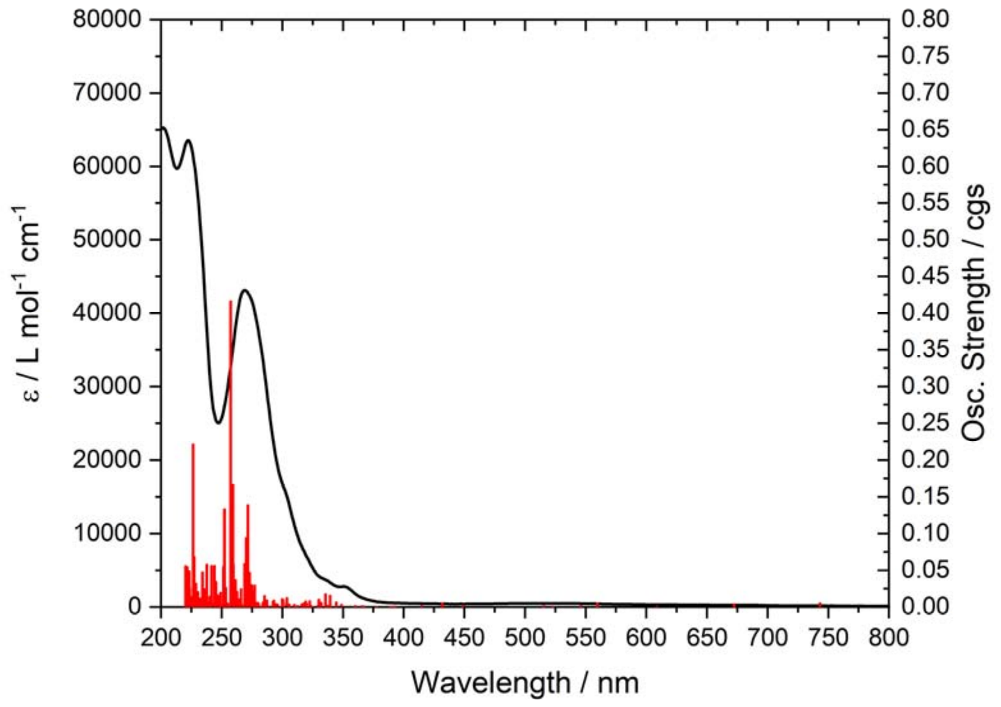

Figure A4-8 Experimental UV-Vis spectrum of compound $[Cr(phen)_2(biim)]^+$ in acetonitrile and calculated oscillatory strength of the 200 lowest energetic electronic transitions.

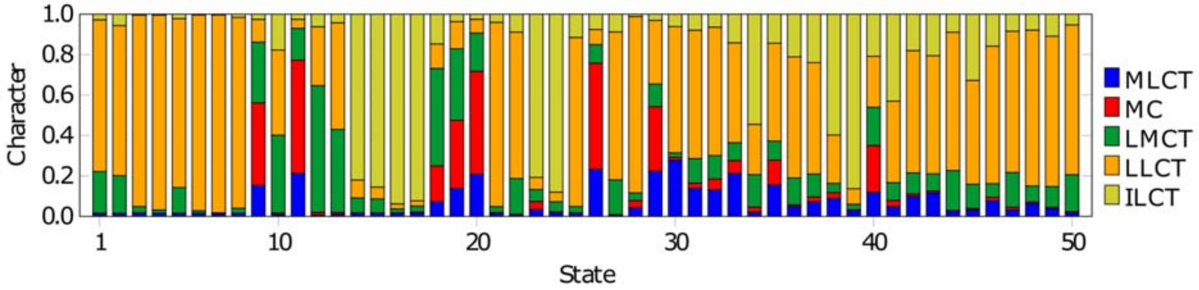

Figure A4-9 TD-DFT charge transfer numbers of $[Cr(phen)_2(biim)]^+$ defined from 0 to 1 of the first 50 electronic transitions.

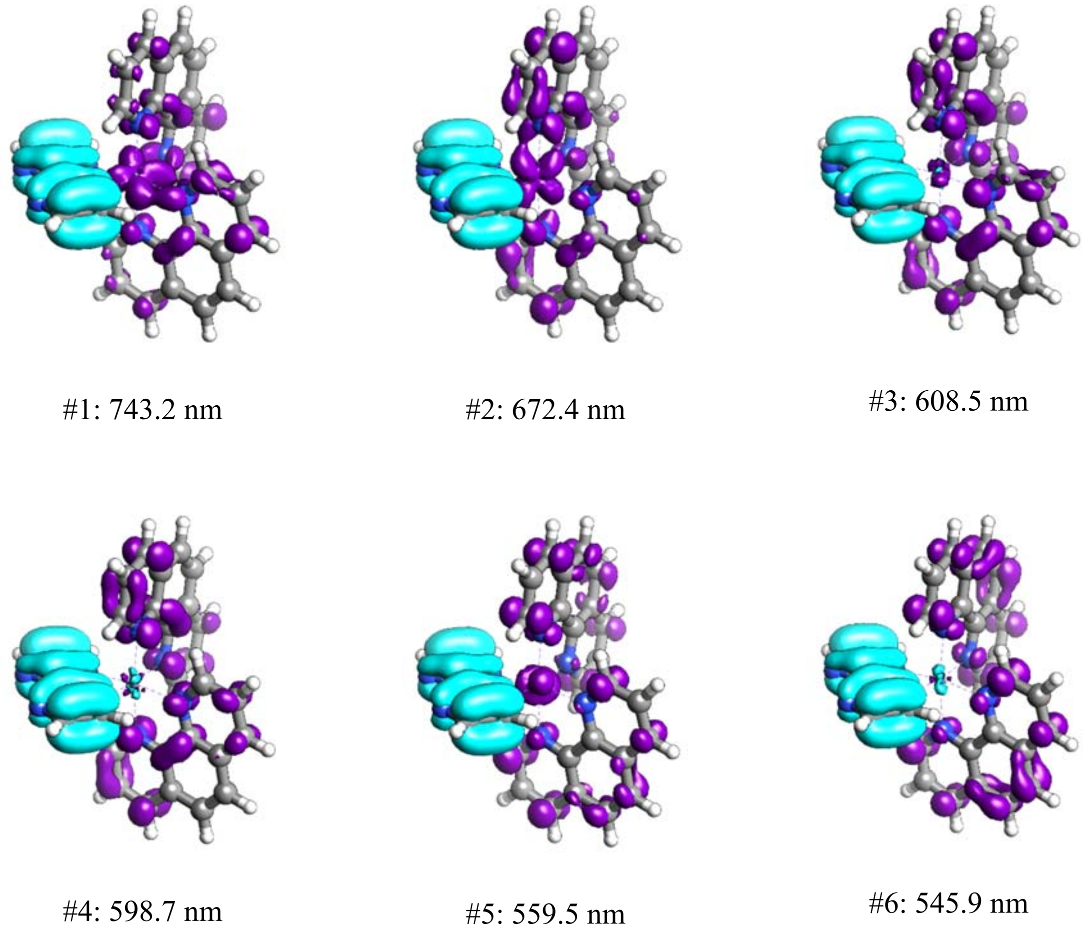

Figure A4-10 Electron density difference maps (EDDM) of the first six electronic transitions calculated for $[Cr(phen)_2(biim)]^+$ (isoval 0.002). Blue: density loss; Purple: density gain.

Cartesian Coordinates

$[Cr(phen)_2(H_2biim)]^{3+}$

Cr	13.39652581016843	8.44517494825328	1.92071204482586
N	13.16110566941928	7.60163285302686	0.05443726758416
N	11.72059196272172	9.47827970413944	1.30002892014835
N	15.17170600936965	7.46611862757432	2.31514743524783
N	14.72034540160341	9.87021935764767	1.23215826278000
N	13.36858499514523	9.21766927421614	3.81805857546584
N	12.22387862865891	7.04648335124162	2.85017741640787
N	12.70527644165193	9.02771314211632	5.89653026485544
H	12.25198034175289	8.67359166464811	6.72729152155713
N	11.22810633704440	6.37019199669175	4.67985727715973
H	10.92163187698761	6.32969569931651	5.64191883248424
C	13.90016501908737	6.66835105347498	-0.53314750006157
H	14.74501737471951	6.28732529981860	0.01974058092418
C	13.60784349143716	6.19024205063817	-1.81404394573382
H	14.24166730690082	5.43117416972404	-2.24725500728140
C	12.52393408559794	6.69214495901225	-2.49650265081674
H	12.27900328232070	6.33702803503998	-3.48848385729473
C	11.72249817556623	7.68182882535133	-1.89641419694530
C	10.57629423913011	8.26773025152439	-2.52082403805996
H	10.30697530566194	7.93317242926128	-3.51313588370708
C	9.84317793319645	9.21885981694994	-1.88649307076888
H	8.97726352869065	9.65780306711766	-2.36271122783801
C	10.19426135139827	9.66590029126946	-0.57369287581118
C	9.47898031247672	10.64544272287814	0.14099227190748
H	8.60821388931893	11.10382090649023	-0.30817201886379
C	9.89634300415127	11.00814107435498	1.40088157532445
H	9.36824663836717	11.75540444906713	1.97378596990061
C	11.02677538450638	10.40125465686067	1.95560866887884
H	11.37702849729144	10.67130918677373	2.94017356244955
C	11.31770499707503	9.10476313791050	0.05634010479362
C	12.08604876497781	8.10772566533585	-0.60801517651066
C	15.35556115589464	6.26787163840074	2.85710812892610
H	14.47229882964490	5.70021808023413	3.10806158915375

C	16.63308901340075	5.74971508926531	3.08926664122482
H	16.72804608273549	4.76906362684513	3.53066835592233
C	17.74044943972898	6.49320547582377	2.75167931854269
H	18.73809796495784	6.11122417105388	2.92155147280227
C	17.57375291616796	7.76704611789120	2.17607230988785
C	18.65812826689147	8.61513818062033	1.78693079391104
H	19.66753926185009	8.26116171997675	1.94511522066201
C	18.42902889886554	9.83336010911569	1.23211191661205
H	19.25189224308815	10.47066076857241	0.93896319235960
C	17.09611062919482	10.30823518027322	1.02139679759274
C	16.78951139478096	11.56042199478323	0.45603661246159
H	17.59154956998953	12.22037149204352	0.15388932190068
C	15.47388796784952	11.92896373384948	0.29576629219153
H	15.20549013508119	12.88249369649699	-0.13357823607352
C	14.45805535543072	11.05628008438268	0.69631123192477
H	13.42047497651334	11.32797144675562	0.57875633765056
C	16.01632075234569	9.49137234048436	1.39717786051310
C	16.25644300800531	8.21383167435735	1.97706956994113
C	13.89412370713185	10.26971913130362	4.52603686133274
H	14.51487401495426	11.01631516987925	4.06494306179859
C	13.48299261441130	10.16045406540350	5.82563742183044
H	13.66983317245683	10.77421140792869	6.68744736745899
C	12.65418936741197	8.47938269065378	4.67572422254118
C	12.02049122023091	7.30867850515478	4.14695407723365
C	11.52739382601162	5.90168366722048	2.55277896691309
H	11.52362175067603	5.47483759136406	1.56624445926539
C	10.90362547399969	5.47024187125888	3.69094494171935
H	10.27115093390264	4.62277661088148	3.88108478879736

$[Cr(phen)_2(Hbiim)]^{2+}$

Cr	5.19671338284875	3.26883000378374	3.22290211014426
N	4.92347274975093	1.30999984552485	3.84495337564951
N	6.17929404127130	2.28357369147123	1.69290924340993
N	4.35479163772777	4.10560302747063	4.91525837475380
N	6.91133310108650	3.53115331228581	4.37581059518318

N	5.37176381383366	5.11002641906732	2.36856341570322
N	3.47642227145019	3.29983664146817	2.19181084178134
N	4.47126038341536	6.65478435061743	1.10947217209872
H	3.81619217654845	7.12959890834676	0.50695745121125
N	2.10164084807460	4.48044349333245	0.84702749200558
C	6.78847671020184	2.80676367963847	0.63659258968591
H	6.81095253084053	3.88407185532232	0.56985219686620
C	7.37874375821003	2.00706837935564	-0.34709711065848
H	7.86470570531994	2.48088492690976	-1.18670079986089
C	7.33017191820995	0.63709818171356	-0.22867266475140
H	7.78026522671411	-0.00048426495665	-0.97760759972872
C	6.68532253318721	0.05716321226871	0.88026648892763
C	6.57729461752349	-1.35342595423481	1.09418444105754
H	7.01580751126206	-2.01772880200557	0.36237322084846
C	5.93993302795498	-1.84829644096031	2.18652416669986
H	5.86039504787103	-2.91522937682452	2.34329080085104
C	5.35657980583694	-0.97533496926235	3.15891460143527
C	4.68083088054507	-1.42261720319090	4.30991981193888
H	4.58395092529903	-2.48421929702253	4.49304074262613
C	4.15275540697943	-0.50369764630882	5.18695431682074
H	3.62928365876593	-0.81498259180481	6.07839608152026
C	4.29246066915479	0.86258929959350	4.92293518764920
H	3.88474511317172	1.60029167502856	5.59720882333790
C	5.45160347514106	0.41408125004638	2.96953906862429
C	6.12010177947174	0.93251642319811	1.82338076194830
C	3.07615067291597	4.37906353524352	5.14195109449985
H	2.37495658999690	4.10618087445132	4.36778901374513
C	2.64966291880157	4.98654414521567	6.32743049367494
H	1.59754548112334	5.18496204657510	6.46597686455073
C	3.57333508122992	5.32039096478011	7.29083583287031
H	3.26603972579661	5.79198328902875	8.21458195779340
C	4.93672845948833	5.04617031872320	7.07166504494776
C	5.97429111164343	5.35856866096454	8.00590244174019
H	5.69856102485599	5.83115420040861	8.93867351066019
C	7.27293979836300	5.07201255004994	7.73060101472819

H	8.05343586472023	5.31245263421986	8.43934494043984
C	7.64496151354292	4.44609266608557	6.49882628796565
C	8.97078584831029	4.12590880855508	6.15061973214382
H	9.77351049346321	4.35702415498781	6.83787535727395
C	9.22812481820001	3.52368665009139	4.94099184577042
H	10.23372582878034	3.26568999811955	4.64461847278413
C	8.16908663638528	3.23822047243860	4.07302391857453
H	8.34761612374446	2.76738131216230	3.11823149688546
C	6.64160023302489	4.12588396650775	5.56806831241897
C	5.27995593011715	4.43046757246170	5.85588520827503
C	6.23292022366514	6.18601061454809	2.33869119341342
H	7.16745074560977	6.18583830770596	2.87000279718000
C	5.68142555047012	7.15462074407971	1.55374857011091
H	6.02915601048999	8.13181997620907	1.27420955491130
C	4.30851410911423	5.42432721493318	1.61527980778927
C	3.25621634537874	4.45627095492433	1.49868778495775
C	1.53708663290269	3.26305801488314	1.13175823988579
H	0.57540923813681	2.97928559020574	0.73653914491894
C	2.37335067652728	2.53170245718495	1.95517112151253
H	2.25819160553273	1.54726527438319	2.37515874579852

$[Cr(phen)_2(biim)]^+$

Cr	1.58532413880107	0.87653104688773	7.96021250911689
N	2.15721311451817	2.22673960232797	9.42304515025510
N	-0.62271007607044	-1.36503923350943	10.54395326089676
N	-0.35271790679862	1.33567272875350	8.07941069133575
N	1.94159613635161	2.18140424300079	6.36531365684591
N	3.61897643453076	0.40707573394708	8.11399645521107
N	-2.34827939479064	0.87136369972341	9.01075633964135
N	1.04813803767815	-0.46890985865698	9.33212172437231
N	1.24200713321243	-0.39997048980287	6.36572761077782
C	1.68643512122529	1.56331552552632	2.71725349586611
H	1.81335228752174	2.09965010013357	1.78685520835405
C	-1.05918066550852	0.55965663439484	8.95556284094314
C	0.62905951301459	-2.41395658735477	5.25043473984467

H	0.33512758493697	-3.44906742455493	5.33946559555189
C	6.18098753844518	1.78292301806138	10.41640159973652
H	7.22816731327469	1.64587911136280	10.64869790148862
C	1.39355177461112	3.10428387671626	10.05970481898179
H	0.36086231742625	3.16166747918079	9.74936019981309
C	5.60483926408433	0.97658078229701	9.38407109538645
C	3.46335960562008	2.10539207777489	9.77351597508089
C	2.48154018810650	4.21249325557852	5.23802483299761
H	2.76987602357534	5.24944124550299	5.32358988971496
C	3.21873033021554	3.80934102509631	11.44698244601348
H	3.63000802621446	4.42643041539036	12.23451533981159
C	1.75360542207569	1.58424088510926	5.16003374866078
C	6.32101868928071	0.00993728555097	8.65299221194518
H	7.37034575202777	-0.14804649366404	8.86256789292420
C	0.76400069758250	-1.81274151080426	4.02028380588342
H	0.57877956078149	-2.36543824526733	3.10906581833128
C	1.31569420762511	0.25679061273147	2.71892944718661
H	1.14062875522561	-0.26856108284470	1.79007191698082
C	-1.26816077385734	2.20076348788529	7.53763227266057
H	-0.99577384439298	2.93780274559195	6.80153775734087
C	-2.48457562716775	1.91029267262819	8.11443476451381
H	-3.43769546384080	2.38268894801651	7.93805895298634
C	2.29263702427957	3.62185314833547	4.01009007724201
H	2.42852883908277	4.18437311015595	3.09621390960203
C	0.87682879224872	-1.67418546169123	6.41171417535554
H	0.78288595471639	-2.11528818993571	7.39268592430040
C	4.04886197667214	2.88300985437330	10.78736595844050
C	4.24391555825688	1.14084839029706	9.07094733042854
C	1.89627203353697	3.91639637985652	11.08242418522061
H	1.23500636886500	4.61624778016703	11.57098958740769
C	1.58696267076168	-1.47916506122270	10.08625299151701
H	2.63288942506913	-1.73121130112685	10.04438162169995
C	5.67567871632485	-0.72139861339072	7.68306685785865
H	6.19632583383538	-1.47020223020745	7.10518345460279
C	5.43462456755782	2.69505103488606	11.09119723197583

H	5.87460060225010	3.29902088249508	11.87287615933046
C	-0.28102895872168	-0.44540115774402	9.64957937474309
C	0.55718552695687	-2.02180781166562	10.82274768809012
H	0.59711794113531	-2.83511234092119	11.52959523524017
C	1.91517100464145	2.26750598755180	3.94203442879818
C	1.37388355881534	0.21003927637347	5.16037674075360
C	1.14736694398507	-0.46019076580220	3.94582137731068
C	2.29498856290924	3.45860121082095	6.40182939442775
H	2.43410883994841	3.90066183963255	7.37707174422750
C	4.31616661147313	-0.49697177786305	7.44021341504628
H	3.78839038986325	-1.06410149608536	6.68805916892864

References

A4-1 a) F. Neese, *WIREs Comput. Mol. Sci.* 2012, **2**, 73-78; b) F. Neese, *WIREs Comput. Mol. Sci.* 2022, e1606.

A4-2 J. Plasser, *J. Chem. Phys.* 2020, 084108.

Table S1. pK_a values for the two successive deprotonations of H_2biim bound in heteroleptic transition-metal complexes or a derivative. H_2bibzim is 2,2'-bibenzimidazole and H_2pybiim is 10-(1-H-imidazole-2-yl)-9H-pyreno[4,5-d]imidazole.

Complex	Metal	pK_{a1}	pK_{a2}	Reference
H_2biim	-	12.31	16.33	25
$[Ru(bpy)_2(H_2biim)]^{2+}$	Ru(II)	7.3	12	39
$[ReCl_2(PPh_3)_2(H_2biim)]^{+}$	Re(III)	4.8	9.8	40
$[Co(en)_2(H_2biim)]^{3+}$	Co(III)	5.9	9.9	41
$[Ru(NH_3)_4(H_2biim)]^{2+}$	Ru(II)	5.7	-	42
$[Ru(pap)_2(H_2biim)]^{2+}$	Ru(II)	4.2	8.0	43
$[Os(pap)_2(H_2biim)]^{2+}$	Os(II)	3.8	6.5	43
$[Mo(C_5H_5)_2(H_2biim)]^{2+}$	Mo(IV)	4.81	9.61	44
$[Ru(bpy)_2(H_2bibzim)]^{2+}$	Ru(II)	5.7	10.1	45
$[Os(bpy)_2(H_2bibzim)]^{2+}$	Os(II)	5.08	9.59	46
$[Ru(bpy)_2(H_2bibzim)]^{3+}$	Ru(III)	0.55	6.60	47
$[Os(bpy)_2(H_2bibzim)]^{3+}$	Os(III)	1.55	6.40	46
$[Ru(bpy)_2(H_2bibzim)]^{4+}$	Ru(IV)	<0	<0	46
$[Os(bpy)_2(H_2bibzim)]^{4+}$	Os(IV)	<0	<0	46
$[Ru(bpy)_2(H_2pybiim)]^{2+}$	Ru(II)	5.09	8.95	47
$[Os(bpy)_2(H_2pybiim)]^{2+}$	Os(II)	4.52	7.97	47

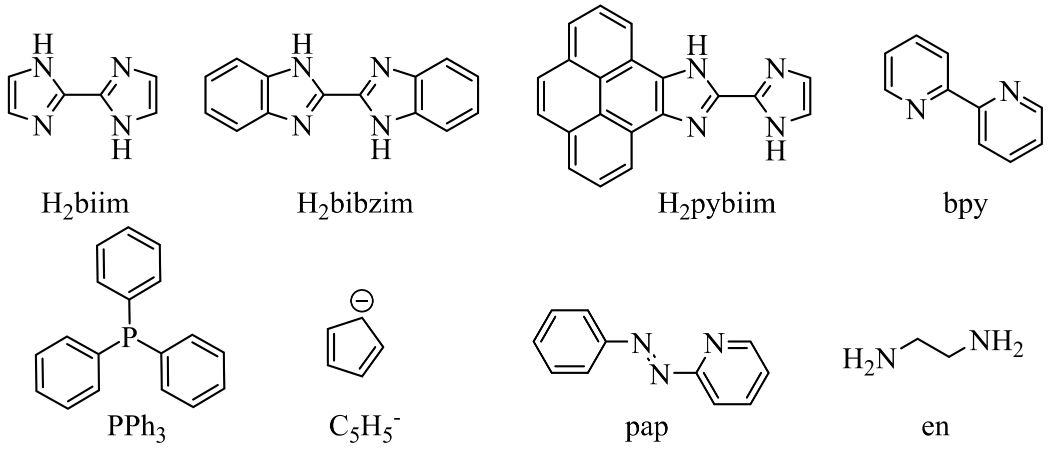

Table S2. Crystal data and structure refinement for $[Cr(H_2biim)_3](NO_3)_3$ **1**.

CCDC number	2355642	
Empirical formula	C18 H18 Cr N15 O9	
Formula weight	640.47	
Temperature	120.00(10) K	
Wavelength	1.54184 Å	
Crystal system	Trigonal	
Space group	R 3 c	
Unit cell dimensions	a = 17.2169(3) Å	α= 90°
	b = 17.2169(3) Å	β= 90°
	c = 15.7297(4) Å	γ = 120°
Volume	4037.95(17) $Å^3$	
Z	6	
Density (calculated)	1.580 Mg/m^3	
Absorption coefficient	4.199 mm^{-1}	
F(000)	1962	
Crystal size	0.604 x 0.063 x 0.047 mm^3	
Theta range for data collection	5.138 to 76.426°.	
Index ranges	-21<=h<=21, -21<=k<=21, -19<=l<=12	
Reflections collected	33096	
Independent reflections	1612 [R(int) = 0.0531]	
Completeness to theta = 67.684°	99.9 %	
Absorption correction	Gaussian	
Max. and min. transmission	1.000 and 0.289	
Refinement method	Full-matrix least-squares on F^2	
Data / restraints / parameters	1612 / 1 / 131	
Goodness-of-fit on F^2	1.126	
Final R indices [I>2sigma(I)]	R1 = 0.0502, wR2 = 0.1473	
R indices (all data)	R1 = 0.0533, wR2 = 0.1500	
Absolute structure parameter	0.419(19)	
Extinction coefficient	n/a	
Largest diff. peak and hole	0.648 and -0.451 e.$Å^{-3}$	

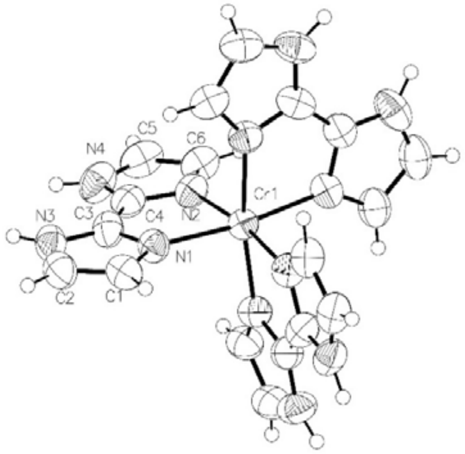

Figure S1 ORTEP view of the complex $[Cr(H_2biim)_3]^{3+}$ in the crystal structure of $[Cr(H_2biim)_3](NO_3)_3$ (thermal ellipsoids are drawn at 50%).

Comments on the crystal structure of $[Cr(H_2biim)_3](NO_3)_3$

The structure is almost centrosymmetric and could be refine in the space group R-3c, at the expense of larger ellipsoids, especially for O2 and the position corresponding to N3/N4. The model in R3c was then preferred. The Flack parameter refine to 0.42(2) which differs enough from 0.5 to privilege this twinned model. The space group contains element of the second kind so that the inversion twinning is anyway equivalent to an orientation ambiguity.

Table S3. Selected bond lengths (Å) and angles (°) for $[Cr(H_2biim)_3](NO_3)_3$.

Cr(1)-N(1)#1	2.031(6)
Cr(1)-N(1)	2.031(6)
Cr(1)-N(1)#2	2.031(6)
Cr(1)-N(2)#1	2.024(7)
Cr(1)-N(2)	2.024(7)
Cr(1)-N(2)#2	2.024(7)
N(1)-Cr(1)-N(1)#2	94.5(2)
N(1)#1-Cr(1)-N(1)	94.5(2)
N(1)#1-Cr(1)-N(1)#2	94.5(2)
N(2)-Cr(1)-N(1)#2	92.64(16)
N(2)-Cr(1)-N(1)	80.41(18)
N(2)#1-Cr(1)-N(1)#1	80.41(18)
N(2)-Cr(1)-N(1)#1	171.54(17)
N(2)#1-Cr(1)-N(1)	92.64(16)
N(2)#2-Cr(1)-N(1)	171.54(17)
N(2)#2-Cr(1)-N(1)#2	80.41(18)
N(2)#2-Cr(1)-N(1)#1	92.64(16)
N(2)#1-Cr(1)-N(1)#2	171.54(17)
N(2)#2-Cr(1)-N(2)	93.0(3)
N(2)#1-Cr(1)-N(2)	93.0(3)
N(2)#1-Cr(1)-N(2)#2	93.0(3)

Symmetry transformations used to generate equivalent atoms: #1 -y+1,x-y,z #2 -x+y+1,-x+1,z

Table S4. Crystal data and structure refinement for [Cr(Hbiim)$_3$] **2**.

CCDC number	2355643	
Empirical formula	C28 H55 Cr N12 O10	
Formula weight	771.84	
Temperature	100.00(11) K	
Wavelength	1.54184 Å	
Crystal system	Trigonal	
Space group	R -3	
Unit cell dimensions	a = 17.8046(3) Å	α= 90°.
	b = 17.8046(3) Å	β= 90°.
	c = 22.4548(4) Å	γ = 120°.
Volume	6164.6(2) $Å^3$	
Z	6	
Density (calculated)	1.247 Mg/m^3	
Absorption coefficient	2.820 mm^{-1}	
F(000)	2466	
Crystal size	0.16 x 0.097 x 0.061 mm^3	
Theta range for data collection	3.477 to 74.981°.	
Index ranges	-21<=h<=22, -21<=k<=21, -27<=l<=27	
Reflections collected	19714	
Independent reflections	2747 [R(int) = 0.0261]	
Completeness to theta = 67.684°	99.8 %	
Absorption correction	Analytical	
Max. and min. transmission	0.861 and 0.743	
Refinement method	Full-matrix least-squares on F^2	
Data / restraints / parameters	2747 / 0 / 94	
Goodness-of-fit on F^2	1.067	
Final R indices [I>2sigma(I)]	R1 = 0.0758, wR2 = 0.2422	
R indices (all data)	R1 = 0.0771, wR2 = 0.2440	
Extinction coefficient	n/a	
Largest diff. peak and hole	0.378 and -0.504 e.$Å^{-3}$	

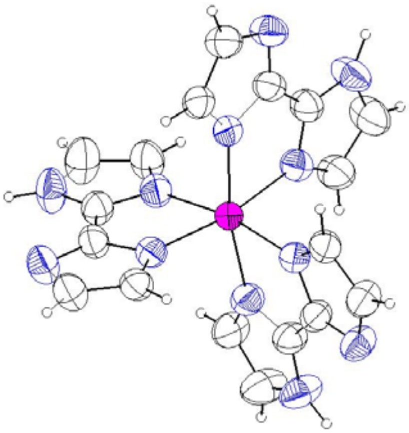

Figure S2 Ortep view of [Cr(Hbiim)3] (thermal ellipsoids are drawn at 50%).

Comments on the crystal structure of [Cr(Hbiim)3]

The complex crystallizes in rhombohedral structure (R-3) with Cr atom located on the 3-fold axis. There are huge voids in the cell that are occupied by highly disordered solvent molecules. We used "squeeze" bypass program and refined the solvent-free structure. A solvent mask was calculated, and 1103 electrons were found in a volume of 3805A^3 in 1 void per unit cell. This is consistent with the presence of 10[CH_3OH] per Formula Unit which account for 1080 electrons per unit cell. The refinement drops down to less than 8% of *R* factors. The neutral Cr complexes are interconnected through hydrogen bonds.

Table S5. Hydrogen bonds for [Cr(Hbiim)3] [Å and °].

D-H···A	d(D-H)	d(H···A)	d(D···A)	<(DHA)
N(4)-H(4)···N(2)#3	0.88	1.89	2.758(4)	167.7

Symmetry transformations used to generate equivalent atoms:
#1 -y+1,x-y+1,z #2 -x+y,-x+1,z #3 -x+4/3,-y+5/3,-z+2/3

Table S6. Selected bond lengths [Å] and angles [°] for [Cr(Hbiim)$_3$].

Cr(1)-N(1)#1	2.036(2)
Cr(1)-N(1)#2	2.036(2)
Cr(1)-N(1)	2.036(2)
Cr(1)-N(3)#1	2.047(2)
Cr(1)-N(3)#2	2.047(2)
Cr(1)-N(3)	2.047(3)
N(1)#1-Cr(1)-N(1)#2	93.74(9)
N(1)#1-Cr(1)-N(1)	93.74(9)
N(1)#2-Cr(1)-N(1)	93.74(9)
N(1)#1-Cr(1)-N(3)	94.74(10)
N(1)-Cr(1)-N(3)#2	94.74(10)
N(1)-Cr(1)-N(3)	79.80(10)
N(1)#2-Cr(1)-N(3)	169.65(9)
N(1)#1-Cr(1)-N(3)#1	79.80(10)
N(1)#2-Cr(1)-N(3)#2	79.80(10)
N(1)#2-Cr(1)-N(3)#1	94.74(10)
N(1)-Cr(1)-N(3)#1	169.65(10)
N(1)#1-Cr(1)-N(3)#2	169.65(10)
N(3)#1-Cr(1)-N(3)#2	92.59(10)
N(3)#1-Cr(1)-N(3)	92.59(10)
N(3)-Cr(1)-N(3)#2	92.59(10)

Symmetry transformations used to generate equivalent atoms: #1 -y+1,x-y+1,z #2 -x+y,-x+1,z

Table S7. Crystal data and structure refinement for [Cr(Hbiim)$_2$(biim)]PPh$_4$(MeOH) **3**.

CCDC number	2355644	
Empirical formula	C43 H38 Cr N12 O P	
Formula weight	821.82	
Temperature	100.00(13) K	
Wavelength	1.54184 Å	
Crystal system	Monoclinic	
Space group	$P\,2_1/c$	
Unit cell dimensions	a = 8.24157(6) Å	α= 90°.
	b = 24.44046(17) Å	β= 99.0717(6)°.
	c = 20.22184(13) Å	γ = 90°.
Volume	4022.29(5) Å^3	
Z	4	
Density (calculated)	1.357 Mg/m^3	
Absorption coefficient	3.128 mm^{-1}	
F(000)	1708	
Crystal size	0.2 x 0.13 x 0.13 mm^3	
Theta range for data collection	2.858 to 74.667°.	
Index ranges	-10<=h<=10, -27<=k<=30, -24<=l<=23	
Reflections collected	38665	
Independent reflections	7991 [R(int) = 0.0225]	
Completeness to theta = 67.684°	100.0 %	
Absorption correction	Analytical	
Max. and min. transmission	0.753 and 0.643	
Refinement method	Full-matrix least-squares on F^2	
Data / restraints / parameters	7991 / 0 / 525	
Goodness-of-fit on F^2	1.036	
Final R indices [I>2sigma(I)]	R1 = 0.0402, wR2 = 0.1114	
R indices (all data)	R1 = 0.0416, wR2 = 0.1123	
Extinction coefficient	n/a	
Largest diff. peak and hole	1.028 and -0.483 e.Å^{-3}	

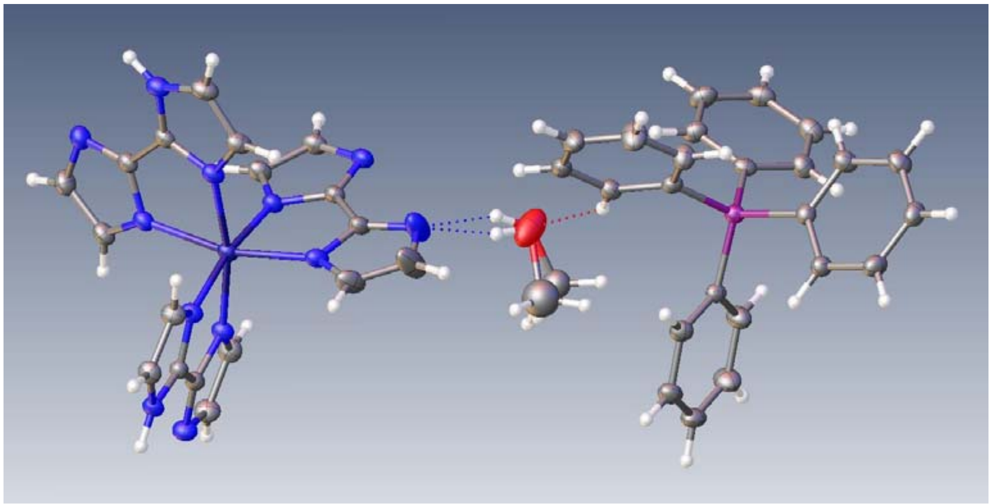

Figure S3 View of the asymmetric unit of [Cr(Hbiim)$_2$(biim)]PPh$_4$(MeOH) (*T*=100K).

Table S8. Hydrogen bonds for [Cr(Hbiim)$_2$(biim)]PPh$_4$(MeOH) [Å and °].

D-H···A	d(D-H)	d(H···A)	d(D···A)	<(DHA)
N(8)-H(8)···N(4)#1	0.88	1.89	2.770(2)	175.7
N(12)-H(12)···N(10)#2	0.88	1.87	2.733(2)	164.4
C(6)-H(6)···N(6)#3	0.95	2.57	3.316(3)	135.5
C(20)-H(20)···O(1)	0.95	2.30	3.139(3)	146.6
O(1)-H(1A)···N(2)	0.84	1.96	2.784(3)	166.6

Symmetry transformations used to generate equivalent atoms:
#1 x,-y+1/2,z-1/2 #2 -x,-y,-z+1 #3 x,-y+1/2,z+1/2

Table S9. Bond lengths [Å] and angles [°] for $[Cr(Hbiim)_2(biim)]PPh_4(MeOH)$.

Cr(1)-N(1)	2.0233(17)
Cr(1)-N(3)	2.0121(16)
Cr(1)-N(5)	2.0201(16)
Cr(1)-N(7)	2.0576(16)
Cr(1)-N(9)	2.0577(17)
Cr(1)-N(11)	2.0508(16)
N(1)-Cr(1)-N(7)	94.70(7)
N(1)-Cr(1)-N(9)	164.73(6)
N(1)-Cr(1)-N(11)	90.34(7)
N(3)-Cr(1)-N(1)	79.92(7)
N(3)-Cr(1)-N(5)	93.78(6)
N(3)-Cr(1)-N(7)	170.97(7)
N(3)-Cr(1)-N(9)	89.57(6)
N(3)-Cr(1)-N(11)	93.36(6)
N(5)-Cr(1)-N(1)	100.52(7)
N(5)-Cr(1)-N(7)	79.98(6)
N(5)-Cr(1)-N(9)	91.13(6)
N(5)-Cr(1)-N(11)	167.90(7)
N(7)-Cr(1)-N(9)	97.03(6)
N(11)-Cr(1)-N(7)	93.93(6)
N(11)-Cr(1)-N(9)	79.16(6)

Table S10. Crystal data and structure refinement for [Cr(Hbiim)$_2$(biim)]PPh$_4$ **4**.

CCDC number	2355645	
Empirical formula	C42 H34 Cr N12 P	
Formula weight	789.78	
Temperature	286(6) K	
Wavelength	1.54184 Å	
Crystal system	Monoclinic	
Space group	$P2_1/n$	
Unit cell dimensions	a = 8.11093(16) Å	α= 90°.
	b = 25.0497(6) Å	β= 99.692(2)°.
	c = 19.6595(6) Å	γ = 90°.
Volume	3937.33(17) Å^3	
Z	4	
Density (calculated)	1.332 Mg/m^3	
Absorption coefficient	3.155 mm^{-1}	
F(000)	1636	
Crystal size	0.12 x 0.1 x 0.06 mm^3	
Theta range for data collection	2.883 to 74.609°.	
Index ranges	-9<=h<=7, -30<=k<=30, -24<=l<=24	
Reflections collected	37800	
Independent reflections	7738 [R(int) = 0.0364]	
Completeness to theta = 67.684°	99.8 %	
Absorption correction	Analytical	
Max. and min. transmission	0.853 and 0.773	
Refinement method	Full-matrix least-squares on F^2	
Data / restraints / parameters	7738 / 0 / 505	
Goodness-of-fit on F^2	1.159	
Final R indices [I>2sigma(I)]	R1 = 0.0657, wR2 = 0.1265	
R indices (all data)	R1 = 0.0828, wR2 = 0.1342	
Extinction coefficient	n/a	
Largest diff. peak and hole	0.411 and -0.365 e.Å^{-3}	

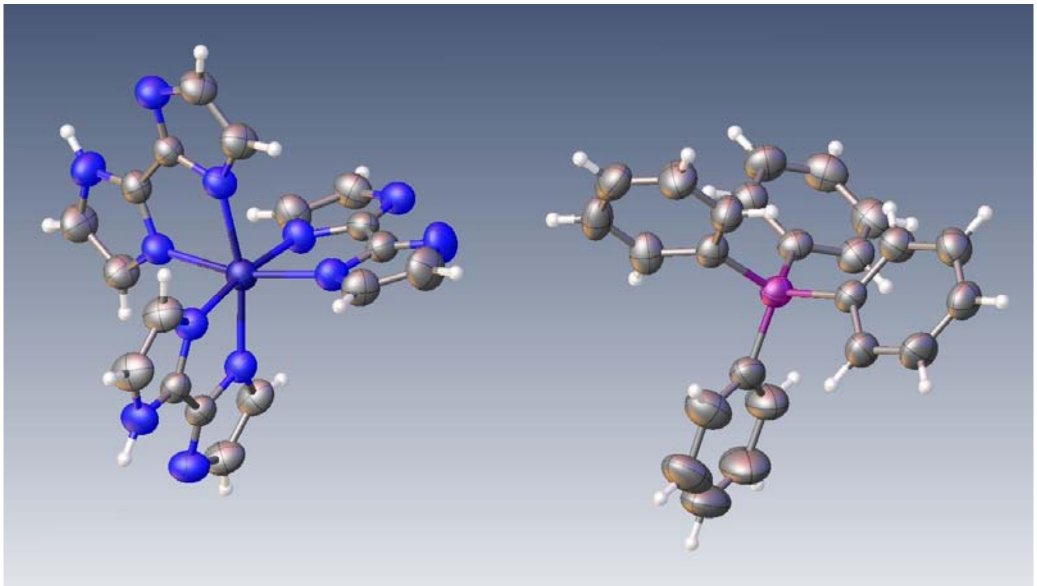

Figure S4 Ortep view of the asymmetric unit of [Cr(Hbiim)$_2$(biim)]PPh$_4$.

Table S11. Hydrogen bonds for [Cr(Hbiim)$_2$(biim)]PPh$_4$ [Å and °].

D-H···A	d(D-H)	d(H···A)	d(D···A)	<(DHA)
N(4)-H(4)···N(10)#1	0.86	1.91	2.744(4)	163.6
N(6)-H(6)···N(8)#2	0.86	1.88	2.730(4)	168.7
C(14)-H(14)···N(2)#3	0.93	2.49	3.237(5)	137.9

Symmetry transformations used to generate equivalent atoms:

#1 x-1/2,-y+3/2,z-1/2 #2 -x+1,-y+2,-z+1 #3 x+1/2,-y+3/2,z+1/2

Table S12. Bond lengths [Å] and angles [°] for $[Cr(Hbiim)_2(biim)]PPh_4$.

Cr(1)-N(1)	2.015(3)
Cr(1)-N(3)	2.018(3)
Cr(1)-N(5)	2.057(3)
Cr(1)-N(7)	2.055(3)
Cr(1)-N(9)	2.036(3)
Cr(1)-N(11)	2.021(3)
N(1)-Cr(1)-N(3)	80.19(10)
N(1)-Cr(1)-N(5)	90.56(10)
N(1)-Cr(1)-N(7)	167.35(11)
N(1)-Cr(1)-N(9)	93.12(11)
N(1)-Cr(1)-N(11)	100.87(11)
N(3)-Cr(1)-N(5)	94.77(10)
N(3)-Cr(1)-N(7)	93.27(10)
N(3)-Cr(1)-N(9)	172.18(11)
N(3)-Cr(1)-N(11)	97.55(11)
N(7)-Cr(1)-N(5)	79.12(10)
N(9)-Cr(1)-N(5)	89.30(10)
N(9)-Cr(1)-N(7)	94.05(11)
N(11)-Cr(1)-N(5)	164.43(11)
N(11)-Cr(1)-N(7)	90.68(11)
N(11)-Cr(1)-N(9)	79.63(11)

Table S13. Crystal data and structure refinement for $[Cr(Me_2biim)_2Cl_2]Cl(MeOH)_3$ **5**.

CCDC number	2355646	
Empirical formula	C19 H32 Cl3 Cr N8 O3	
Formula weight	578.87	
Temperature	120.00(10) K	
Wavelength	1.54184 Å	
Crystal system	Triclinic	
Space group	*P* -1	
Unit cell dimensions	a = 6.8363(2) Å	α= 99.106(2)°.
	b = 13.7717(3) Å	β= 95.598(2)°.
	c = 14.7817(3) Å	γ = 104.370(2)°.
Volume	1317.44(6) $Å^3$	
Z	2	
Density (calculated)	1.459 Mg/m^3	
Absorption coefficient	6.686 mm^{-1}	
F(000)	602	
Crystal size	0.21 x 0.02 x 0.01 mm^3	
Theta range for data collection	3.059 to 75.776°.	
Index ranges	-5<=h<=8, -16<=k<=17, -18<=l<=18	
Reflections collected	17103	
Independent reflections	5256 [R(int) = 0.0617]	
Completeness to theta = 67.684°	99.5 %	
Absorption correction	Analytical	
Max. and min. transmission	0.923 and 0.531	
Refinement method	Full-matrix least-squares on F^2	
Data / restraints / parameters	5256 / 0 / 327	
Goodness-of-fit on F^2	1.046	
Final R indices [I>2sigma(I)]	R1 = 0.0704, wR2 = 0.1859	
R indices (all data)	R1 = 0.0840, wR2 = 0.1943	
Extinction coefficient	n/a	
Largest diff. peak and hole	1.132 and -0.807 e.$Å^{-3}$	

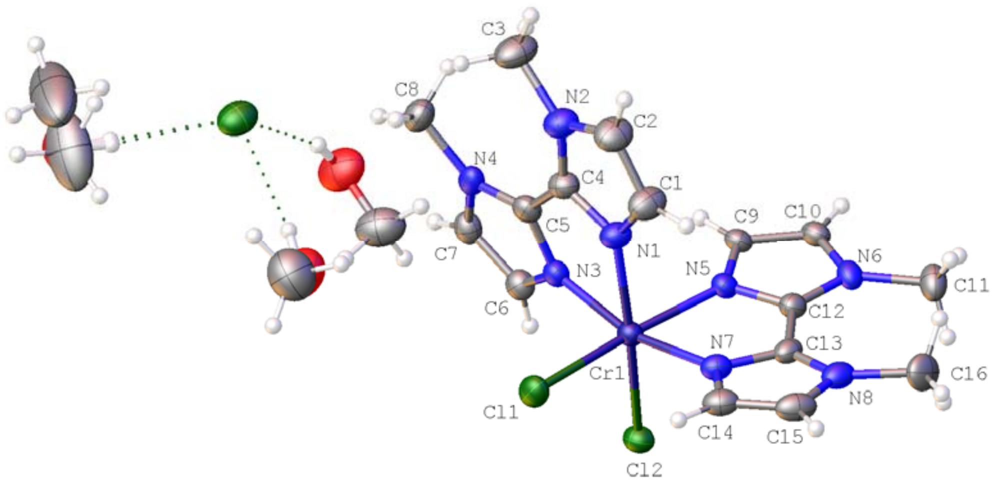

Figure S5 Ortep view of the full asymmetric unit of [Cr(Me$_2$biim)$_2$Cl$_2$]Cl(MeOH)$_3$ (thermal ellipsoids are drawn at 50% probability level).

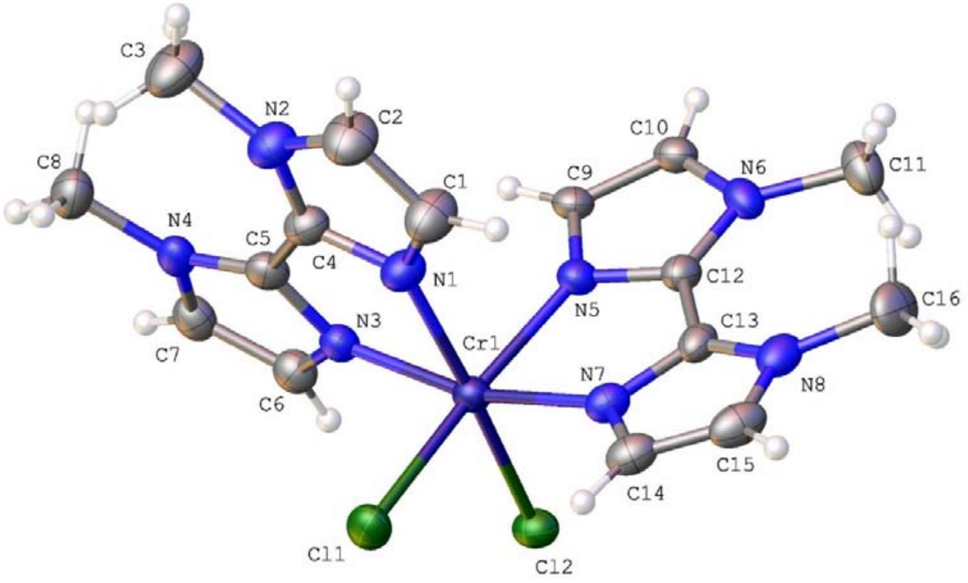

Figure S6 Ortep view of [Cr(Me$_2$biim)$_2$Cl$_2$]$^+$ (thermal ellipsoids are drawn at 50% probability level) with numbering scheme. Solvent molecules and counter ion Cl- are omitted.

Table S14. Hydrogen bonds for [Cr(Me$_2$biim)$_2$Cl$_2$]Cl(MeOH)$_3$ [Å and °].

D-H···A	d(D-H)	d(H···A)	d(D···A)	<(DHA)
O(1)-H(1A)···Cl(3)	0.84	2.34	3.148(4)	160.4
O(2)-H(2A)···Cl(3)	0.84	2.29	3.124(5)	171.5
O(3)-H(3BB)···Cl(3)	0.84	2.29	3.126(6)	171.3

Table S15. Bond lengths [Å] and angles [°] for $[Cr(Me_2biim)_2Cl_2]Cl(MeOH)_3$.

Cr(1)-Cl(1)	2.3219(11)
Cr(1)-Cl(2)	2.3346(11)
Cr(1)-N(1)	2.025(4)
Cr(1)-N(3)	2.054(3)
Cr(1)-N(5)	2.043(3)
Cr(1)-N(7)	2.038(3)
Cl(1)-Cr(1)-Cl(2)	92.54(4)
N(1)-Cr(1)-Cl(1)	89.68(11)
N(1)-Cr(1)-Cl(2)	172.38(11)
N(1)-Cr(1)-N(3)	78.17(14)
N(1)-Cr(1)-N(5)	88.51(14)
N(1)-Cr(1)-N(7)	90.74(14)
N(3)-Cr(1)-Cl(1)	95.24(10)
N(3)-Cr(1)-Cl(2)	94.36(10)
N(5)-Cr(1)-Cl(1)	171.68(10)
N(5)-Cr(1)-Cl(2)	90.31(10)
N(5)-Cr(1)-N(3)	92.33(13)
N(7)-Cr(1)-Cl(1)	93.99(10)
N(7)-Cr(1)-Cl(2)	96.38(10)
N(7)-Cr(1)-N(3)	165.50(14)
N(7)-Cr(1)-N(5)	77.92(14)

Table S16. Crystal data and structure refinement for $[Cr(Me_2biim)_3](CF_3SO_3)_3$ **6**.

CCDC number	2355647	
Empirical formula	C27 H30 Cr F9 N12 O9 S3	
Formula weight	985.81	
Temperature	120.00(10) K	
Wavelength	1.54184 Å	
Crystal system	Monoclinic	
Space group	*C* 2/*c*	
Unit cell dimensions	a = 16.6274(3) Å	α= 90°
	b = 19.7662(3) Å	β= 108.113(2)°
	c = 12.2436(2) Å	γ = 90°
Volume	3824.58(13) Å^3	
Z	4	
Density (calculated)	1.712 Mg/m^3	
Absorption coefficient	5.015 mm^{-1}	
F(000)	2004	
Crystal size	0.339 x 0.173 x 0.095 mm^3	
Theta range for data collection	3.581 to 73.162°.	
Index ranges	-15<=h<=20, -23<=k<=24, -14<=l<=14	
Reflections collected	26988	
Independent reflections	3723 [R(int) = 0.0255]	
Completeness to theta = 67.684°	100.0 %	
Absorption correction	Gaussian	
Max. and min. transmission	1.000 and 0.126	
Refinement method	Full-matrix least-squares on F^2	
Data / restraints / parameters	3723 / 0 / 306	
Goodness-of-fit on F^2	1.064	
Final R indices [I>2sigma(I)]	R1 = 0.0302, wR2 = 0.0829	
R indices (all data)	R1 = 0.0311, wR2 = 0.0835	
Extinction coefficient	n/a	
Largest diff. peak and hole	0.357 and -0.500 e.Å^{-3}	

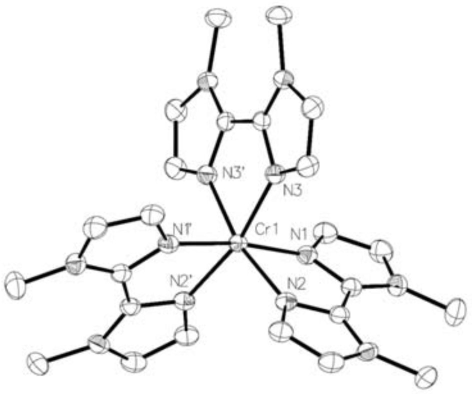

Figure S7 ORTEP view of the complex $[Cr(Me_2biim)_3]^{3+}$ in the crystal structure of $[Cr(Me_2biim)_3](CF_3SO_3)_3$. Hydrogen atoms and $CF_3SO_3^-$ are omitted for clarity and thermal ellipsoids are drawn at 50% probability level.

Table S17. Selected bond lengths (Å) and angles (°) for $[Cr(Me_2biim)_3](CF_3SO_3)_3$.

Cr(1)-N(1)#1	2.0242(14)
Cr(1)-N(1)	2.0242(14)
Cr(1)-N(2)#1	2.0207(14)
Cr(1)-N(2)	2.0207(13)
Cr(1)-N(3)	2.0304(14)
Cr(1)-N(3)#1	2.0304(14)
N(1)#1-Cr(1)-N(1)	171.88(8)
N(1)#1-Cr(1)-N(3)	92.70(5)
N(1)#1-Cr(1)-N(3)#1	93.60(5)
N(1)-Cr(1)-N(3)	93.60(5)
N(1)-Cr(1)-N(3)#1	92.70(5)
N(2)#1-Cr(1)-N(1)#1	78.51(5)
N(2)#1-Cr(1)-N(1)	96.07(5)
N(2)-Cr(1)-N(1)	78.51(5)
N(2)-Cr(1)-N(1)#1	96.07(5)
N(2)#1-Cr(1)-N(2)	97.45(8)
N(2)#1-Cr(1)-N(3)#1	92.77(5)
N(2)-Cr(1)-N(3)#1	167.14(5)
N(2)#1-Cr(1)-N(3)	167.14(5)
N(2)-Cr(1)-N(3)	92.77(5)
N(3)#1-Cr(1)-N(3)	78.28(8)

Symmetry transformations used to generate equivalent atoms: #1 -x+1,y,-z+3/2

Table S18. Crystal data and structure refinement for $[Cr(phen)_2(H_2biim)](CF_3SO_3)_3(H_2O)_{0.25}$ **7**.

CCDC number	2355648	
Empirical formula	C33 H22.50 Cr F9 N8 O9.25 S3	
Formula weight	998.27	
Temperature	109.99(10) K	
Wavelength	1.54184 Å	
Crystal system	Monoclinic	
Space group	$P\ 2_1/c$	
Unit cell dimensions	a = 17.8506(3) Å	α= 90°.
	b = 17.2070(3) Å	β= 93.9394(14)°.
	c = 12.78022(17) Å	γ = 90°.
Volume	3916.23(10) $Å^3$	
Z	4	
Density (calculated)	1.693 Mg/m^3	
Absorption coefficient	4.889 mm^{-1}	
F(000)	2014	
Crystal size	0.29 x 0.05 x 0.02 mm^3	
Theta range for data collection	2.481 to 75.422°.	
Index ranges	-21<=h<=22, -19<=k<=21, -14<=l<=15	
Reflections collected	34192	
Independent reflections	7876 [R(int) = 0.0320]	
Completeness to theta = 67.684°	100.0 %	
Absorption correction	Analytical	
Max. and min. transmission	0.928 and 0.470	
Refinement method	Full-matrix least-squares on F^2	
Data / restraints / parameters	7876 / 0 / 584	
Goodness-of-fit on F^2	1.057	
Final R indices [I>2sigma(I)]	R_1 = 0.0720, wR_2 = 0.1882	
R indices (all data)	R_1 = 0.0824, wR_2 = 0.1952	
Extinction coefficient	n/a	
Largest diff. peak and hole	1.215 and -0.874 e.$Å^{-3}$	

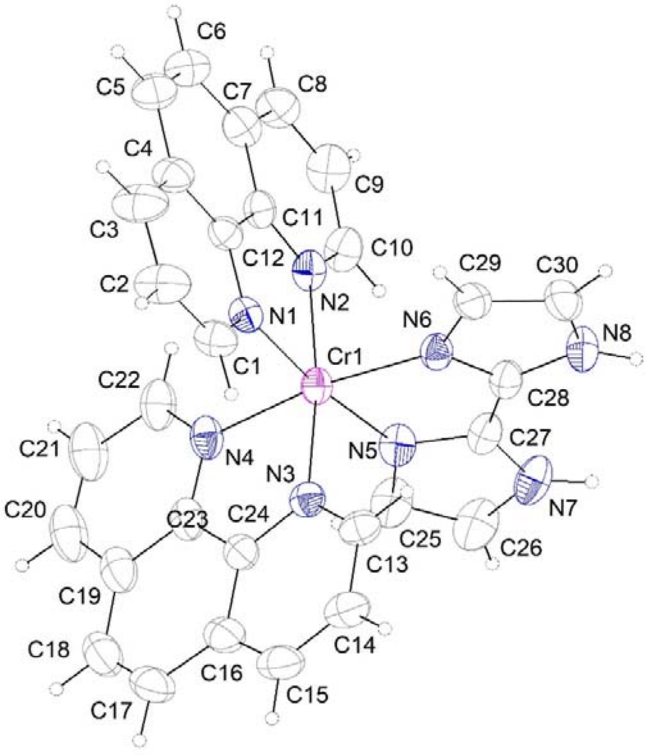

Figure S8 Ortep view of $[Cr(phen)_2(H_2biim)](CF_3SO_3)_3(H_2O)_{0.25}$ (thermal ellipsoids are drawn at 50% probability level) with numbering scheme. Triflate counter ions are omitted for clarity.

Comments on the crystal structure of $[Cr(phen)_2(H_2biim)](CF_3SO_3)_3\cdot(H_2O)_{0.25}$.

This Cr-complex was recrystallized in acetonitrile. We found a disordered water molecule in the crystal structure (site occupancy ~ 0.25) making hydrogen bond with a N-H of the complex.

Table S19. Hydrogen bonds for $[Cr(phen)_2(H_2biim)](CF_3SO_3)_3(H_2O)_{0.25}$ [Å and °].

D-H···A	d(D-H)	d(H···A)	d(D···A)	<(DHA)
N(7)-H(7)···O(9)	0.88	2.11	2.798(5)	134.6
N(7)-H(7)···O(11)	0.88	1.89	2.651(13)	143.3
N(8)-H(8)···O(1)	0.88	2.58	3.105(5)	119.5
N(8)-H(8)···O(2)	0.88	1.89	2.753(5)	168.6

Table S20. Selected bond lengths [Å] and angles [°] for $[Cr(phen)_2(H_2biim)](CF_3SO_3)_3(H_2O)_{0.25}$.

Cr(1)-N(1)	2.051(3)
Cr(1)-N(2)	2.057(3)
Cr(1)-N(3)	2.053(3)
Cr(1)-N(4)	2.054(3)
Cr(1)-N(5)	2.029(3)
Cr(1)-N(6)	2.019(3)
N(1)-Cr(1)-N(2)	80.79(13)
N(1)-Cr(1)-N(3)	94.52(13)
N(1)-Cr(1)-N(4)	95.16(13)
N(3)-Cr(1)-N(2)	172.89(13)
N(3)-Cr(1)-N(4)	81.05(14)
N(4)-Cr(1)-N(2)	93.99(14)
N(5)-Cr(1)-N(1)	173.47(14)
N(5)-Cr(1)-N(2)	95.29(14)
N(5)-Cr(1)-N(3)	89.86(14)
N(5)-Cr(1)-N(4)	90.30(14)
N(6)-Cr(1)-N(1)	94.03(13)
N(6)-Cr(1)-N(2)	90.53(13)
N(6)-Cr(1)-N(3)	95.15(13)
N(6)-Cr(1)-N(4)	170.31(13)
N(6)-Cr(1)-N(5)	80.74(13)

Table S21. Crystal data and structure refinement for $[Cr(phen)_2(Hbiim)](CF_3SO_3)_2(H_2O)_{0.5}$ **8**.

CCDC number	2355649	
Empirical formula	C32 H22 Cr F6 N8 O6.50 S2	
Formula weight	852.69	
Temperature	120.00(10) K	
Wavelength	1.54184 Å	
Crystal system	Triclinic	
Space group	*P* -1	
Unit cell dimensions	a = 10.6371(4) Å	α= 79.588(3)°.
	b = 13.6703(5) Å	β= 68.575(4)°.
	c = 14.0130(6) Å	γ = 68.511(3)°.
Volume	1762.42(13) $Å^3$	
Z	2	
Density (calculated)	1.607 Mg/m^3	
Absorption coefficient	4.594 mm^{-1}	
F(000)	864	
Crystal size	0.16 x 0.05 x 0.02 mm^3	
Theta range for data collection	3.393 to 76.272°.	
Index ranges	-13<=h<=12, -16<=k<=16, -17<=l<=17	
Reflections collected	43826	
Independent reflections	7102 [R(int) = 0.0623]	
Completeness to theta = 67.684°	99.9 %	
Absorption correction	Analytical	
Max. and min. transmission	0.904 and 0.609	
Refinement method	Full-matrix least-squares on F^2	
Data / restraints / parameters	7102 / 0 / 545	
Goodness-of-fit on F^2	1.074	
Final R indices [I>2sigma(I)]	R1 = 0.0544, wR2 = 0.1486	
R indices (all data)	R1 = 0.0651, wR2 = 0.1574	
Extinction coefficient	n/a	
Largest diff. peak and hole	0.655 and -0.712 e.$Å^{-3}$	

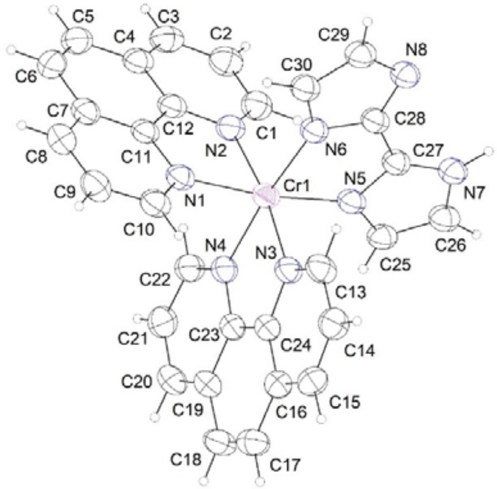

Figure S9 Ortep view of $[Cr(phen)_2(Hbiim)](CF_3SO_3)_2(H_2O)_{0.5}$ (thermal ellipsoids are drawn at 50% probability level) with numbering scheme. Triflate counter ions and water molecule are omitted for clarity pupose.

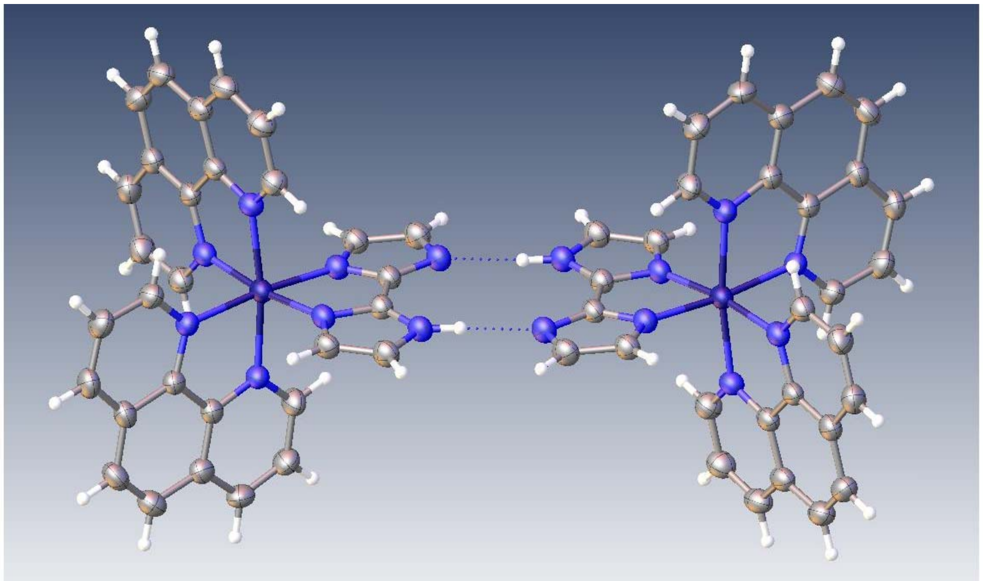

Figure S10 Intermolecular H-bonds found in $[Cr(phen)_2(Hbiim)](CF_3SO_3)_2(H_2O)_{0.5}$.

Comments on the crystal structure of $[Cr(phen)_2(Hbiim)](CF_3SO_3)_2(H_2O)_{0.5}$

There are 2 $CF_3SO_3^-$ in the asymetric unit (in agreement with the protonation of the ligand and the valence state of the Cr^{3+}). One $CF_3SO_3^-$ is disordered onto 2 sites and modeled in two parts with refined occupancies 0.73/0.27. A water molecule is found close to this disordered triflate and was refined with occupancy fixed to 0.5. This water molecule could do hydrogen bond with the other ordered $CF_3SO_3^-$ molecule. There are intermolecular hydrogen bonds between two complexes through N-H···N bonds (see Table S22 and Figure S10).

Table S22. Hydrogen bonds for $[Cr(phen)_2(Hbiim)](CF_3SO_3)_2(H_2O)_{0.5}$ [Å and °].

D-H···A	d(D-H)	d(H···A)	d(D···A)	<(DHA)
N(7)-H(7)···N(8)#1	0.88	1.90	2.758(3)	164.5

Symmetry transformations used to generate equivalent atoms: #1 -x,-y+1,-z

Table S23. Selected bond lengths [Å] and angles [°] for $[Cr(phen)_2(Hbiim)](CF_3SO_3)_2(H_2O)_{0.5}$.

Cr(1)-N(1)	2.059(3)
Cr(1)-N(2)	2.067(2)
Cr(1)-N(3)	2.055(2)
Cr(1)-N(4)	2.067(2)
Cr(1)-N(5)	2.018(3)
Cr(1)-N(6)	2.018(2)
N(1)-Cr(1)-N(2)	80.70(10)
N(1)-Cr(1)-N(4)	90.31(10)
N(3)-Cr(1)-N(1)	94.11(10)
N(3)-Cr(1)-N(2)	171.55(9)
N(3)-Cr(1)-N(4)	80.67(9)
N(4)-Cr(1)-N(2)	92.64(9)
N(5)-Cr(1)-N(1)	172.33(9)
N(5)-Cr(1)-N(2)	94.19(10)
N(5)-Cr(1)-N(3)	91.61(10)
N(5)-Cr(1)-N(4)	95.66(10)
N(5)-Cr(1)-N(6)	80.64(10)
N(6)-Cr(1)-N(1)	93.79(10)
N(6)-Cr(1)-N(2)	92.34(9)
N(6)-Cr(1)-N(3)	94.66(9)
N(6)-Cr(1)-N(4)	174.01(9)

Table S24. Crystal data and structure refinement for $[Cr(phen)_2(biim)]CF_3SO_3(CH_3OH)_{1.5}$ **9**.

CCDC number	2355650	
Empirical formula	C32.50 H26 Cr F3 N8 O4.50 S	
Chemical formula moiety	$C_{30}H_{20}CrN_8$, CF_3O_3S, 1.5(CH_4O)	
Formula weight	741.67	
Temperature	150.00(10) K	
Wavelength	1.54184 Å	
Crystal system	Triclinic	
Space group	*P* -1	
Unit cell dimensions	a = 11.7735(4) Å	α= 107.378(3)°.
	b = 11.8329(4) Å	β= 98.544(3)°.
	c = 12.6821(4) Å	γ = 96.353(3)°.
Volume	1644.79(9) Å^3	
Z	2	
Density (calculated)	1.498 Mg/m^3	
Absorption coefficient	4.068 mm^{-1}	
F(000)	760	
Crystal size	0.28 x 0.16 x 0.11 mm^3	
Theta range for data collection	3.721 to 70.623°.	
Index ranges	-14<=h<=13, -14<=k<=14, -15<=l<=15	
Reflections collected	12239	
Independent reflections	6162 [R(int) = 0.0311]	
Completeness to theta = 67.684°	99.8 %	
Absorption correction	Analytical	
Max. and min. transmission	0.690 and 0.504	
Refinement method	Full-matrix least-squares on F^2	
Data / restraints / parameters	6162 / 0 / 454	
Goodness-of-fit on F^2	1.045	
Final R indices [I>2sigma(I)]	R1 = 0.0563, wR2 = 0.1698	
R indices (all data)	R1 = 0.0603, wR2 = 0.1757	
Extinction coefficient	n/a	
Largest diff. peak and hole	1.616 and -0.623 e.Å^{-3}	

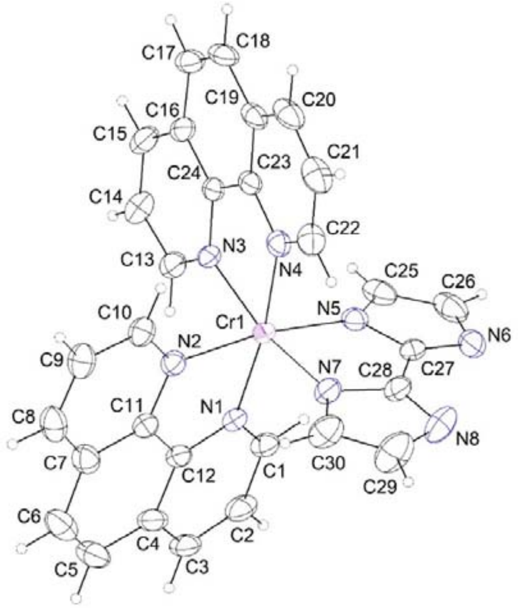

Figure S11 Ortep view of $[Cr(phen)_2(biim)]CF_3SO_3(CH_3OH)_{1.5}$ (thermal ellipsoids are drawn at 50% probability level).

Comments on the crystal structure of $[Cr(phen)_2(biim)]CF_3SO_3(CH_3OH)_{1.5}$

Deprotonated complex crystallizes with one $CF_3SO_3^-$ counterion and MeOH solvent molecules. One MeOH is fully ordered and makes H-bond with the $CF_3SO_3^-$ ion (see Table S25). Another MeOH is disordered and refined with fixed 0.5 occupancy and with isotropic ADPs. The chemical formula moiety of the asymmetric unit is $C_{30}H_{20}CrN_8$, CF_3O_3S, $1.5(CH_4O)$.

Table S25. Hydrogen bonds for $[Cr(phen)_2(biim)]CF_3SO_3 \cdot (CH_3OH)_{1.5}$ [Å and °].

D-H···A	d(D-H)	d(H···A)	d(D···A)	<(DHA)
O(2S)-H(2S)···O(1)	0.84	2.25	2.951(6)	141.1

Table S26. Selected bond lengths [Å] and angles [°] for $[Cr(phen)_2(biim)]CF_3SO_3(CH_3OH)_{1.5}$.

Cr(1)-N(4)	2.067(3)
Cr(1)-N(7)	1.993(3)
Cr(1)-N(2)	2.069(2)
Cr(1)-N(3)	2.086(2)
Cr(1)-N(5)	2.000(2)
Cr(1)-N(1)	2.063(2)
N(4)-Cr(1)-N(2)	92.83(10)
N(4)-Cr(1)-N(3)	79.90(10)
N(7)-Cr(1)-N(4)	91.56(10)
N(7)-Cr(1)-N(2)	93.18(10)
N(7)-Cr(1)-N(3)	169.81(10)
N(7)-Cr(1)-N(5)	81.42(10)
N(7)-Cr(1)-N(1)	92.61(10)
N(2)-Cr(1)-N(3)	92.79(9)
N(5)-Cr(1)-N(4)	94.30(10)
N(5)-Cr(1)-N(2)	171.16(10)
N(5)-Cr(1)-N(3)	93.59(9)
N(5)-Cr(1)-N(1)	92.83(10)
N(1)-Cr(1)-N(4)	172.20(9)
N(1)-Cr(1)-N(2)	80.37(10)
N(1)-Cr(1)-N(3)	96.52(9)

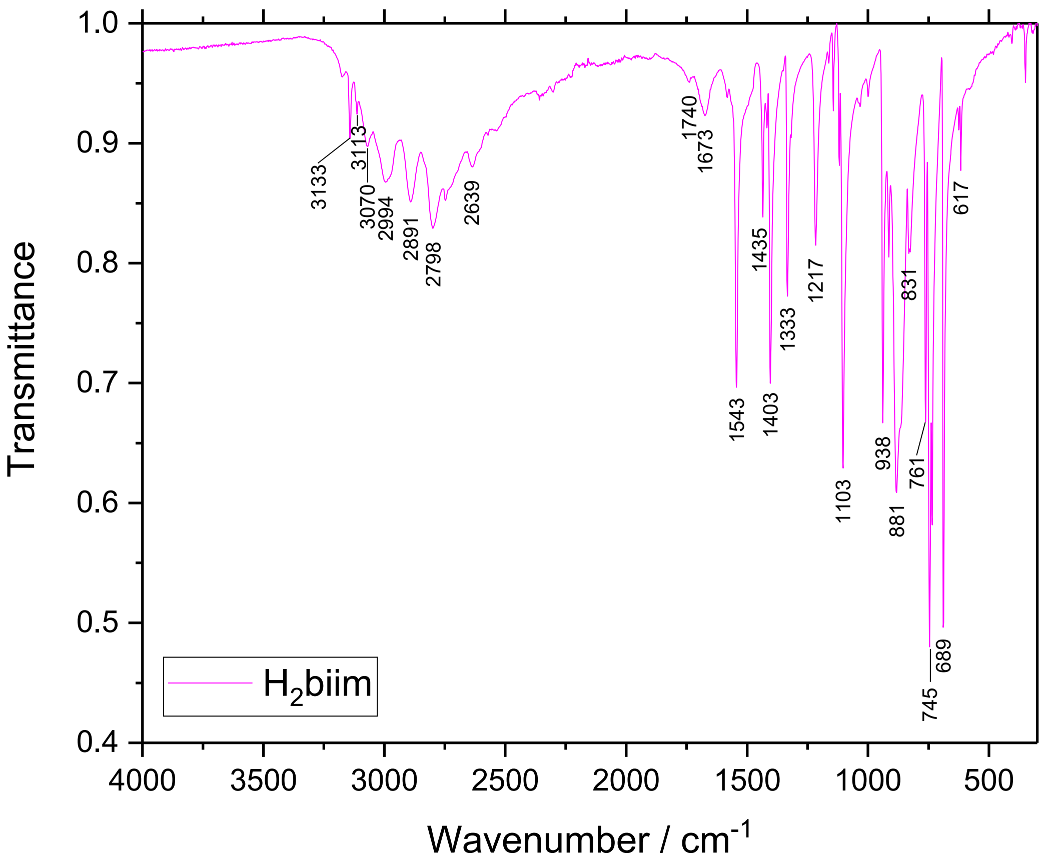

Figure S12 FT-IR spectrum of the ligand H_2biim in the solid state.

Table S27 Observed IR peaks and their respective attribution based on literature.[36]

Observed position of peaks / cm^{-1}	Attribution
3133; 3113	ν C-H (aromatic)
3400-2250 (large band)	ν N-H
1543; 1435; 1403; 1333; 914; 881; 689; 617	Ring vibration
1216	ν C2-C2'
1103; 939	δ C,N-H
825	γ N-H
762; 746; 735	γ C-H

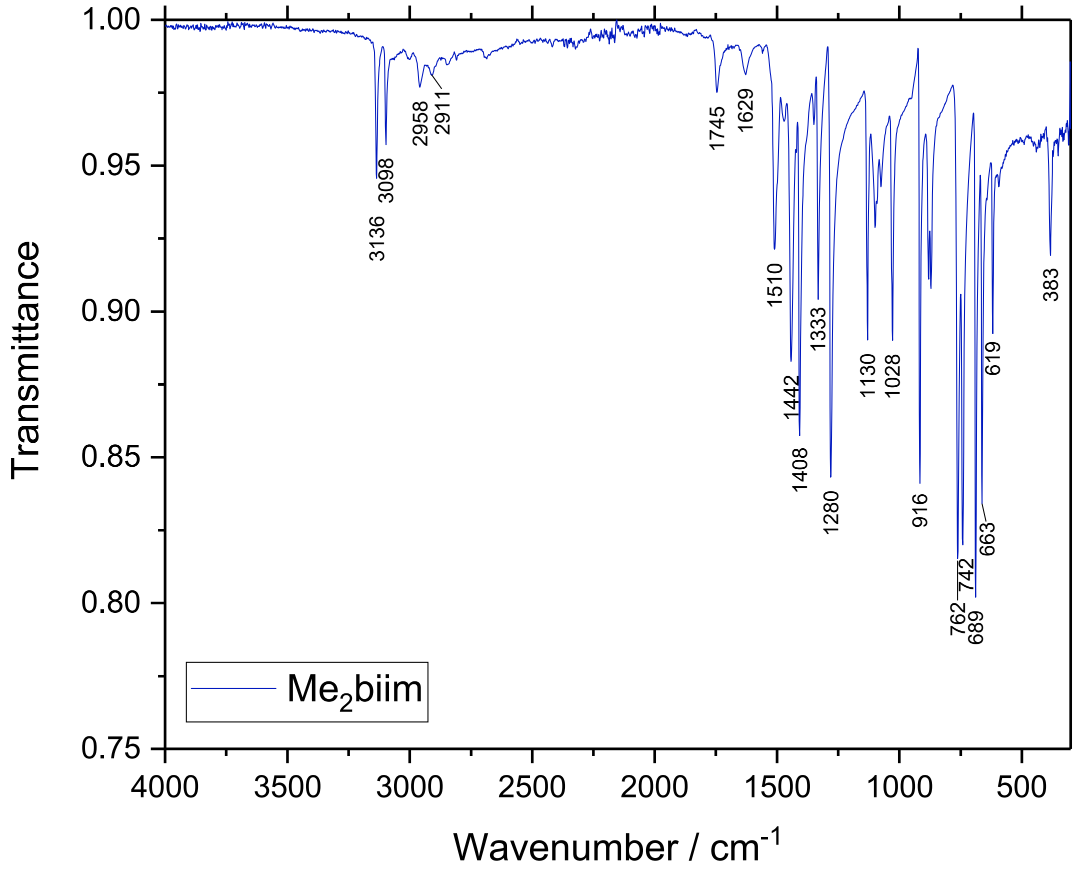

Figure S13 FT-IR spectrum of the ligand Me_2biim in the solid state.

Table S28 Observed IR peaks and their respective attribution based on literature.[36]

Observed position of peaks / cm^{-1}	Attribution
3136; 3098	ν C-H (aromatic)
3000; 2958; 2911	ν C-H ($-CH_3$)
1745; 1629	Harmonic
1442; 1408	δ CH_3
1509; 1333; 1280; 916; 688; 663; 619	R
1130; 1099	δ C-H
1074; 1028	ρ CH_3
762; 742	γ C-H

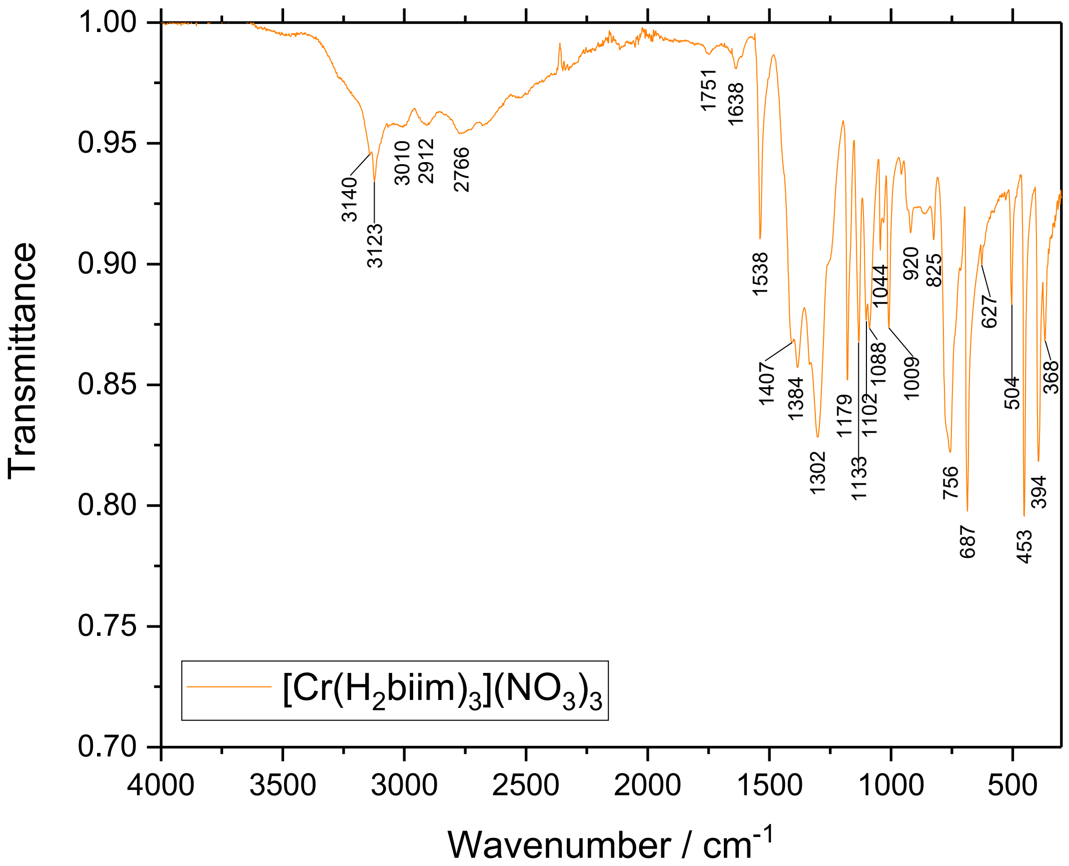

Figure S14 FT-IR spectrum of the complex $[Cr(H_2biim)_3](NO_3)_3$ in the solid state.

Table S29 Observed IR peaks and their respective attribution based on literature.[36]

Observed position of peaks / cm^{-1}	Attribution
3140; 3123	ν C-H (aromatic)
3400-2250 (large band)	ν N-H
1751-627	Ligand vibrations
1384; 1302; 825	NO_3^- vibrations
504; 453	Chelate ring deformation
394; 368	ν Cr-N

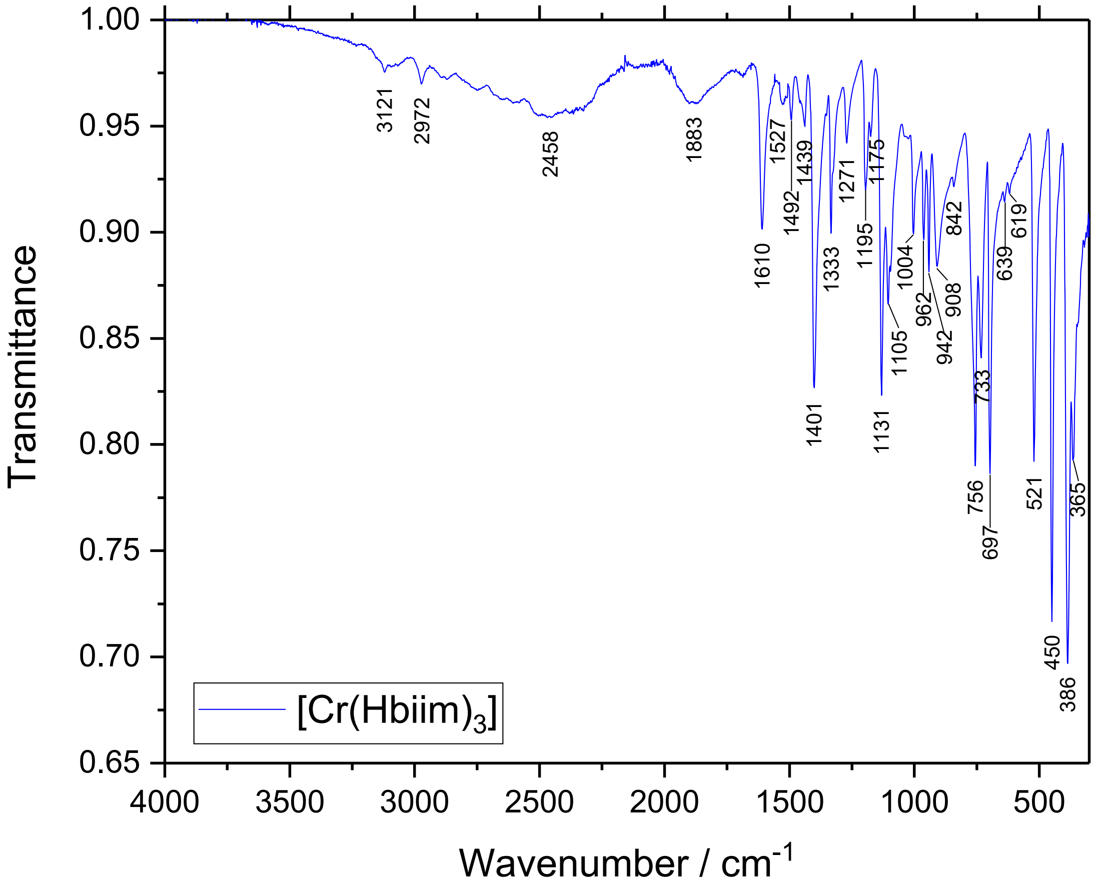

Figure S15 FT-IR spectrum of the complex [Cr(Hbiim)$_3$] in the solid state.

Table S30 Observed IR peaks and their respective attribution based on literature.[36]

Observed position of peaks / cm^{-1}	Attribution
3121	ν C-H (aromatic)
3200-2200 (large band)	ν N-H
2972	ν C-H (MeOH)
1883	2γ N-H
1756-619	Ligand vibrations
521; 450	Chelate ring deformations
386; 365	ν Cr-N

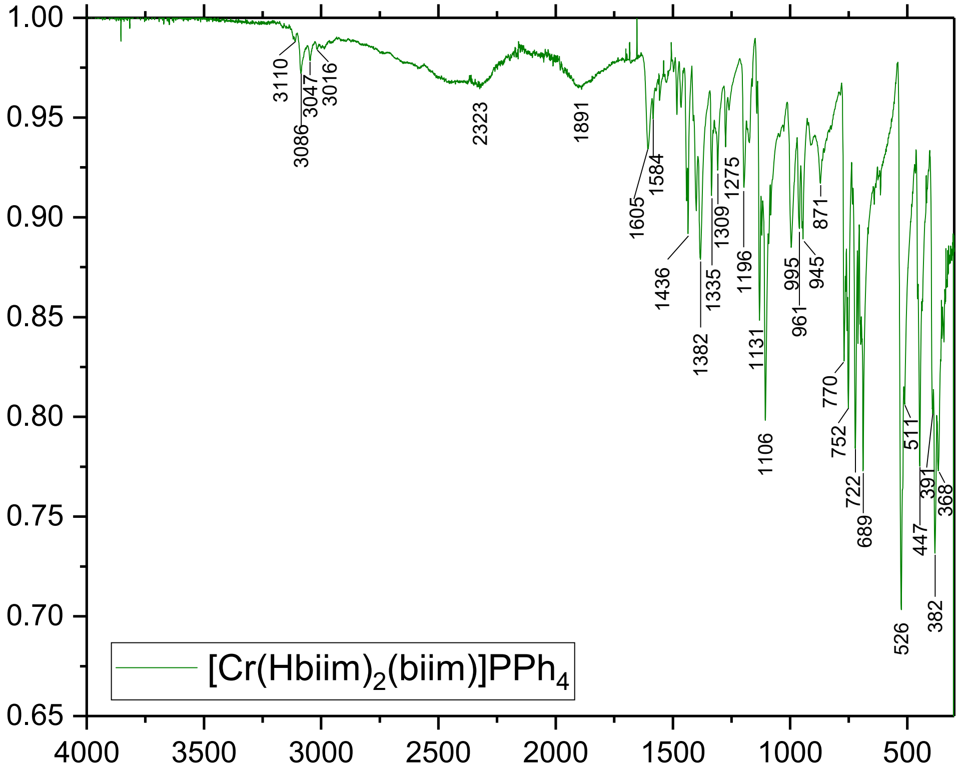

Figure S16 FT-IR spectrum of the complex $[Cr(Hbiim)_2(biim)]PPh_4$ in the solid state.

Table S31 Observed IR peaks and their respective attribution based on literature.[36]

Observed position of peaks / cm^{-1}	Attribution
3110; 3086	ν C-H (aromatic)
3000-2200 (large band)	ν N-H
1891	2γ N-H
3047; 1584; 1483; 1436; 1309; 1106; 995; 770; 752; 722; 689; 526	PPh_4^+
1605-600	Ligand vibrations
511; 447	Chelate ring deformations
391; 382; 368	ν Cr-N

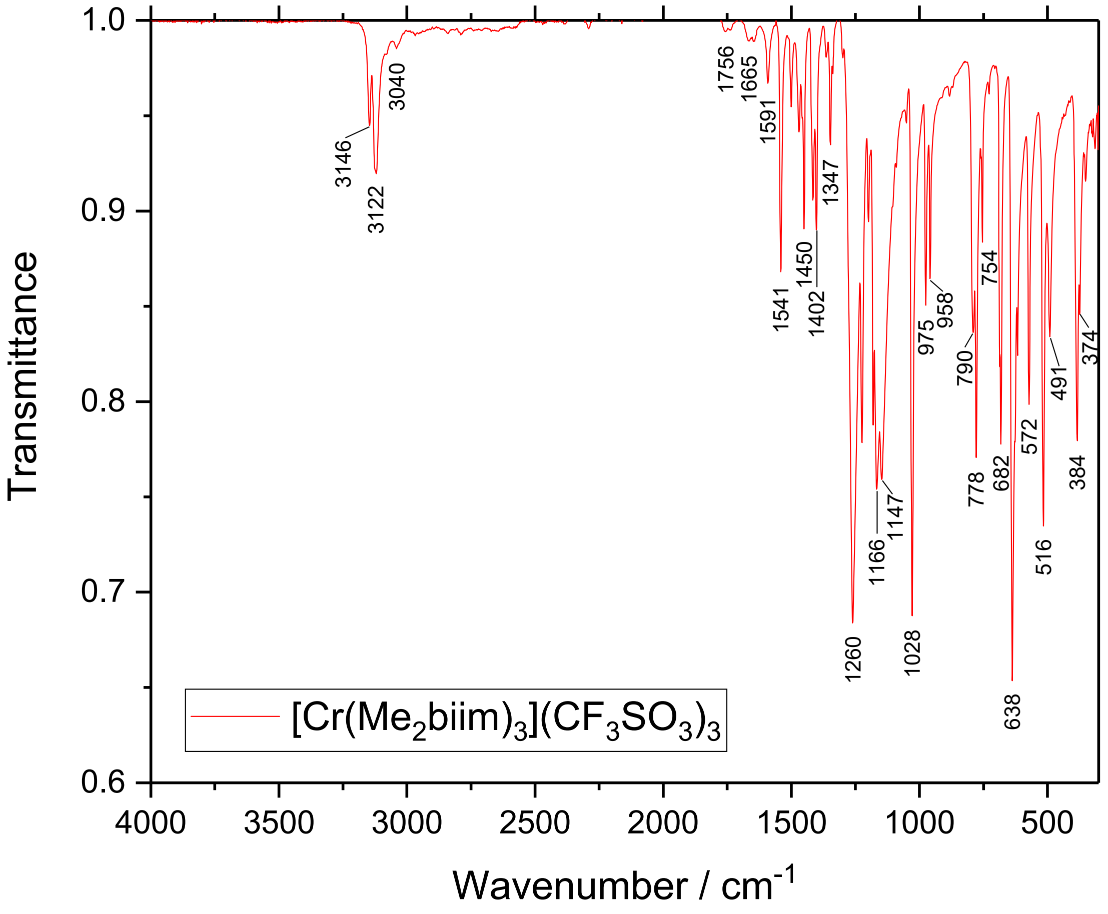

Figure S17 FT-IR spectrum of the complex $[Cr(Me_2biim)_3](CF_3SO_3)_3$ in the solid state.

Table S32 Observed IR peaks and their respective attribution based on literature.[36]

Observed position of peaks / cm^{-1}	Attribution
3146; 3122	ν C-H (aromatic)
3040	ν C-H ($-CH_3$)
1756-616	Ligand vibrations
1260; 1224; 1167; 778; 1028; 638; 572; 516;	$CF_3SO_3^-$
491	Chelate ring deformation
384; 374	ν Cr-N

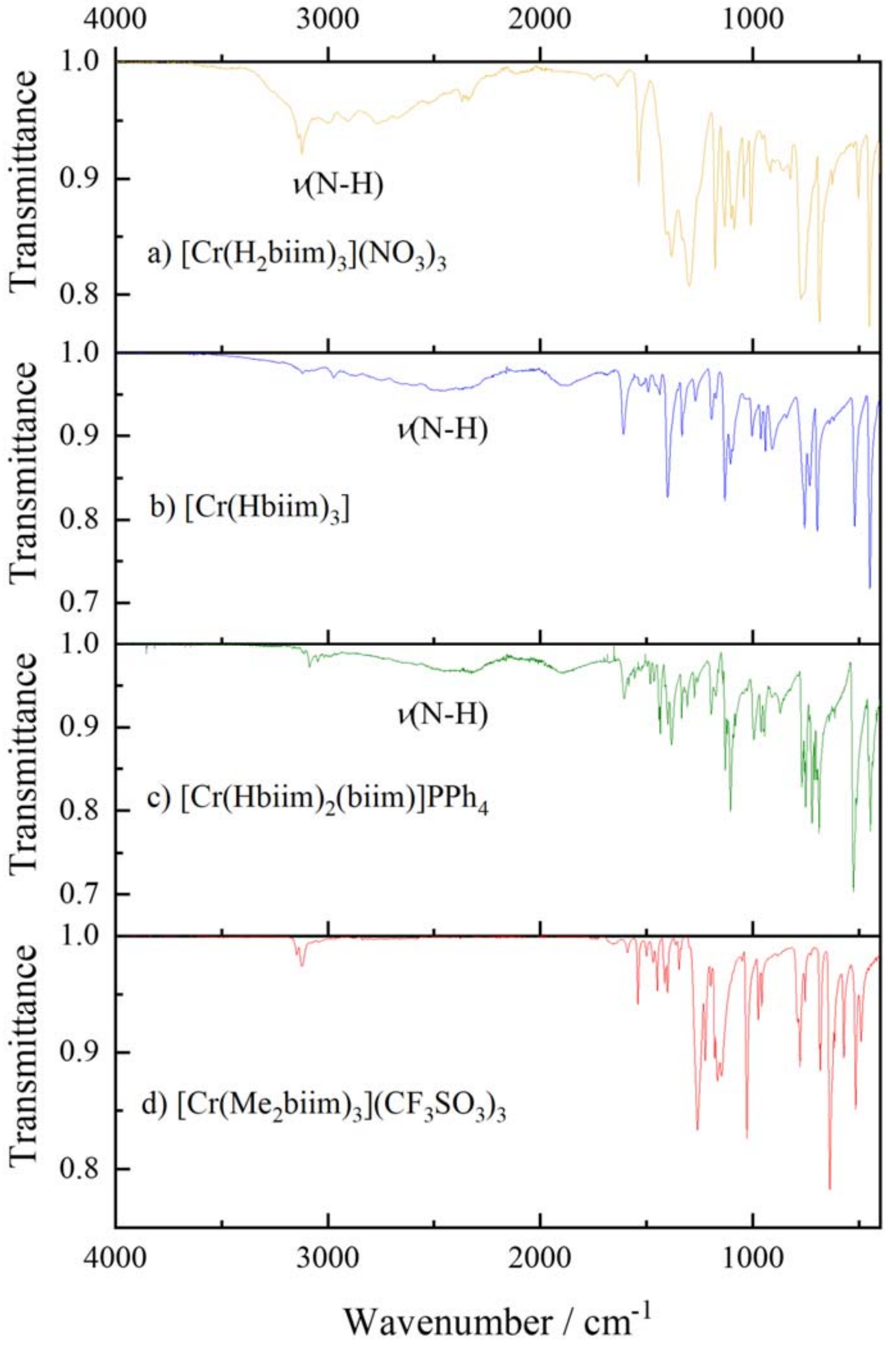

Figure S18 Stacked solid state FT-IR spectrum of a) $[Cr(H_2biim)_3](NO_3)_3$ (orange), b) $[Cr(Hbiim)_3]$ (blue), c) $[Cr(Hbiim)_2(biim)]PPh_4$ (green) and d) $[Cr(Me_2biim)_3](CF_3SO_3)_3$ (red).

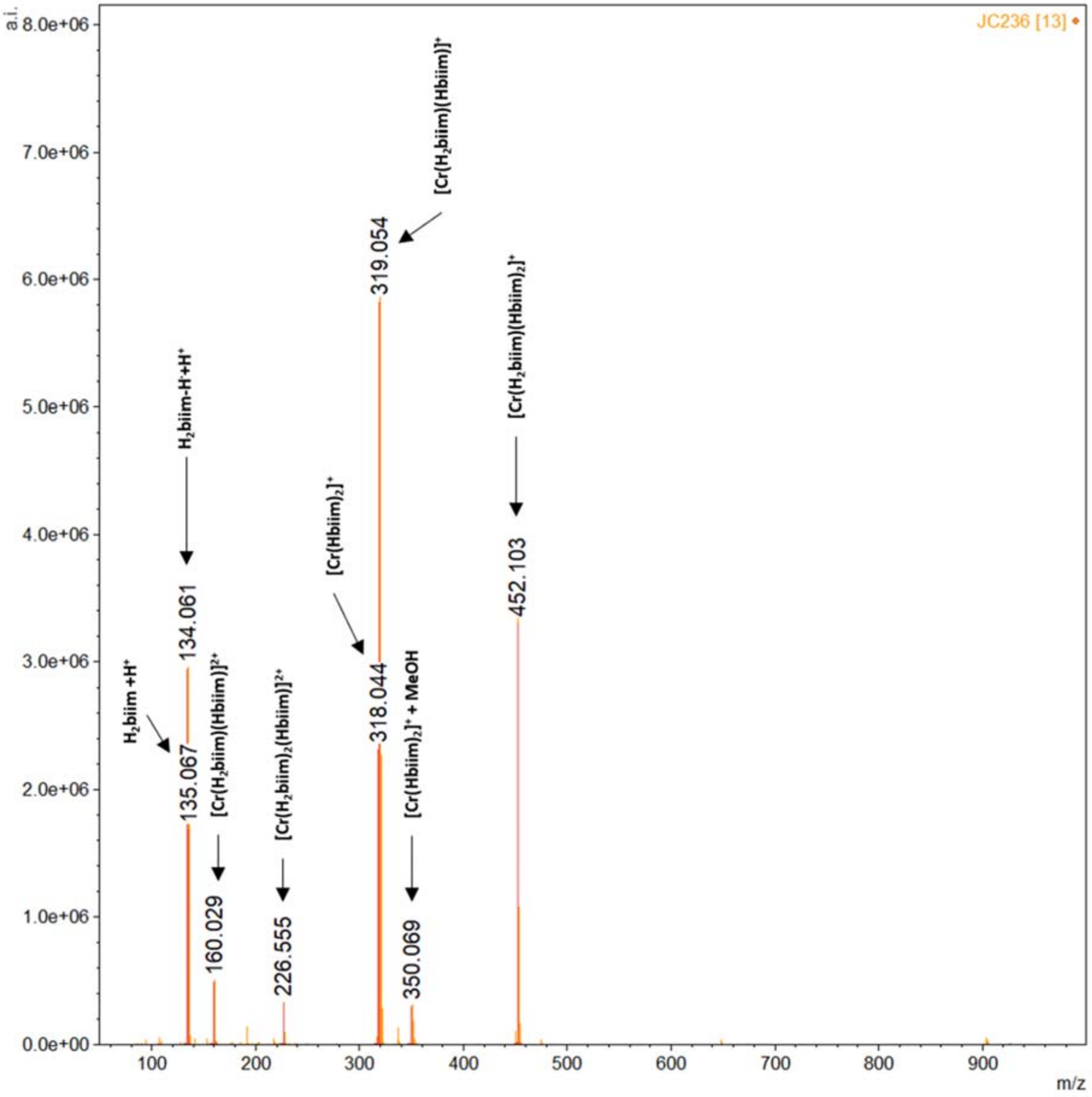

Figure S19 ESI-MS full spectrum of the complex [Cr(H_2biim)$_3$](NO_3)$_3$ in MeOH.

Table S33 Observed m/z, intensity and attribution of each peak observed in the MS spectrum of [Cr(H_2biim)$_3$](NO_3)$_3$ in MeOH.

Peak n°	*m/z*	Intensity / a.u.	Attribution (fragment)	Molecular formula	Calc. *m/z*
1	134.061	2.95E+06	H_2biim-H$^{\cdot}$+H$^+$	$C_6H_6N_4^+$	134.0587
2	135.067	1.69E+06	H_2biim +H$^+$	$C_6H_7N_4^+$	135.0665
3	159.525	1.08E+05	[Cr(H_2biim)(Hbiim)]$^{2+}$	$C_{12}H_{11}CrN_8^{2+}$	159.5250
4	160.029	4.93E+05	[Cr(H_2biim)$_2$]$^{2+}$	$C_{12}H_{12}CrN_8^{2+}$	160.0289
5	226.555	3.28E+05	[Cr(H_2biim)$_2$(Hbiim)]$^{2+}$	$C_{18}H_{17}CrN_{12}^{2+}$	226.5547
6	318.044	2.32E+06	[Cr(Hbiim)$_2$]$^+$	$C_{12}H_{10}CrN_8^+$	318.0428
7	319.054	5.83E+06	[Cr(H_2biim)(Hbiim)]$^+$	$C_{12}H_{11}CrN_8^+$	319.0506
8	350.069	3.04E+05	[Cr(Hbiim)$_2$]$^+$ + MeOH	$C_{13}H_{14}CrN_8O^+$	350.0690
9	452.103	3.30E+06	[Cr(H_2biim)(Hbiim)$_2$]$^+$	$C_{18}H_{16}CrN_{12}^+$	452.1021

Peaks n°1+2:

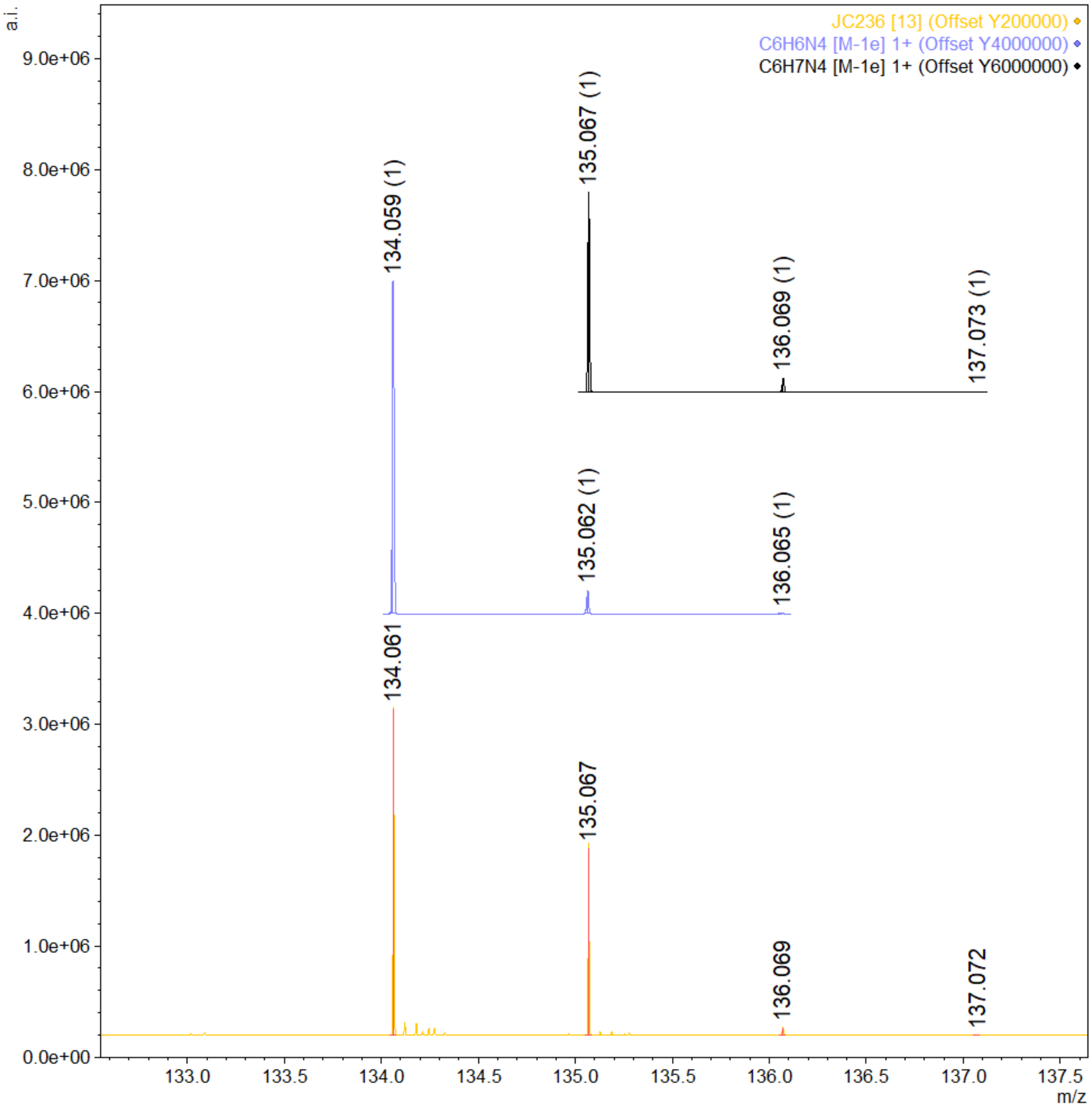

Figure S20 Experimental ESI-MS spectrum of $[Cr(H_2biim)_3](NO_3)_3$ in MeOH (orange). Calculated peaks for $H_2biim\text{-}H^{\cdot}+H^+$, $C_6H_6N_4^+$ (purple); and for $H_2biim +H^+$, $C_6H_7N_4^+$ (black).

Peaks n°3+4:

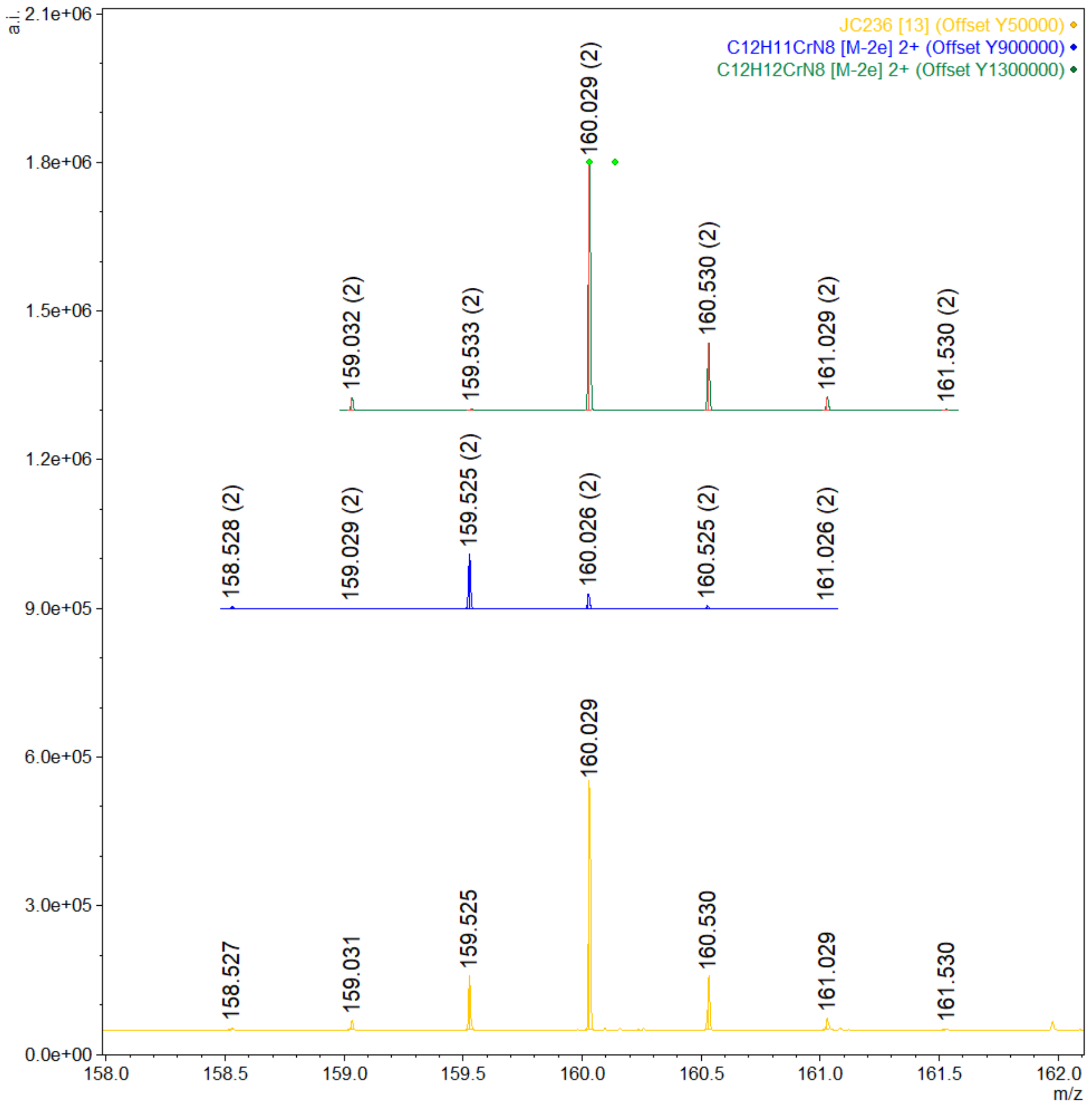

Figure S21 Experimental ESI-MS spectrum of $[Cr(H_2biim)_3](NO_3)_3$ in MeOH (orange). Calculated peaks for $[Cr(H_2biim)(Hbiim)]^{2+}$, $C_{12}H_{11}CrN_8^{2+}$ (blue); and for $[Cr(H_2biim)_2]^{2+}$; $C_{12}H_{12}CrN_8^{2+}$ (green).

Peak n°5:

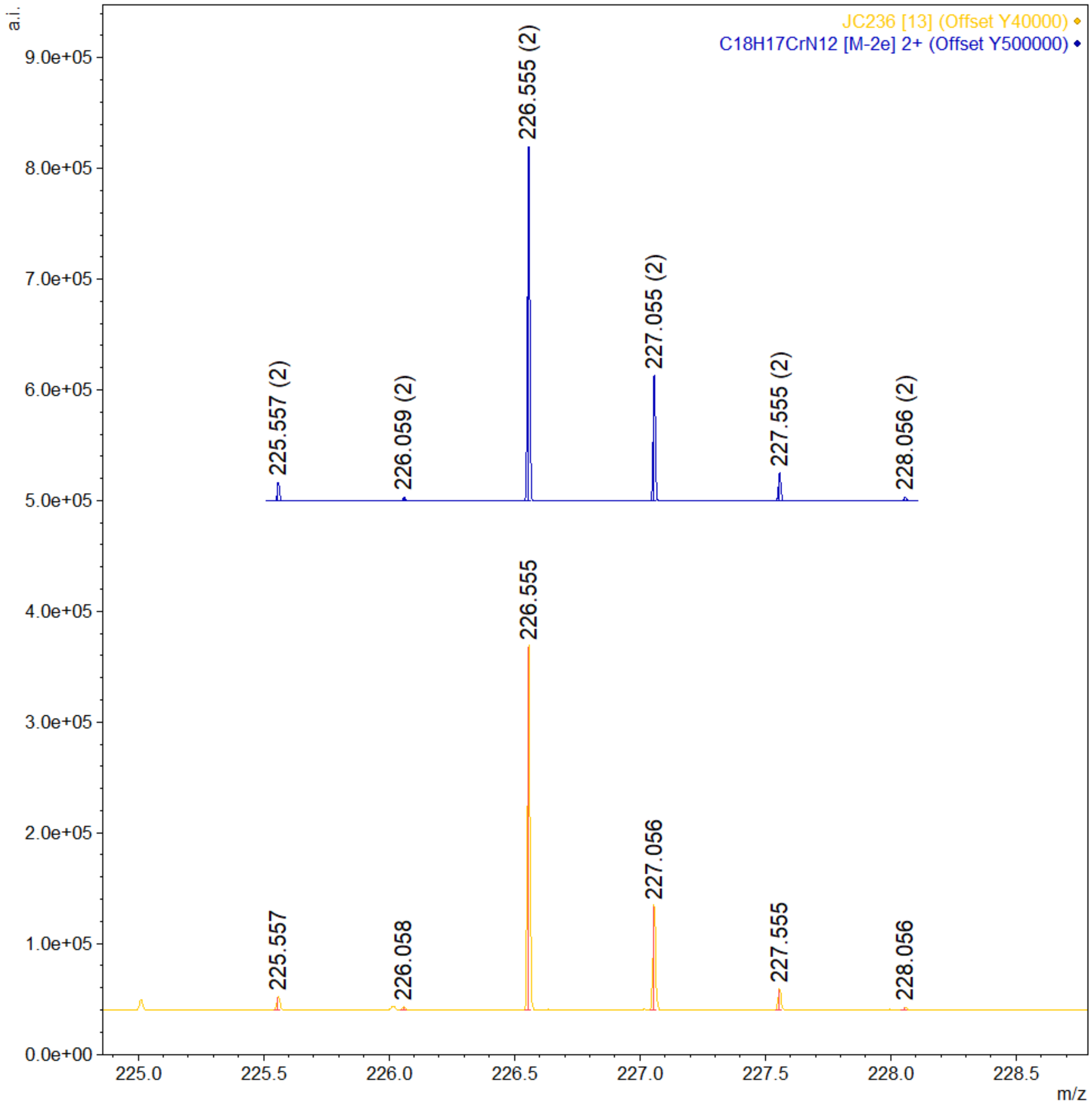

Figure S22 Experimental ESI-MS spectrum of $[Cr(H_2biim)_3](NO_3)_3$ in MeOH (orange). Calculated peaks for $[Cr(H_2biim)_2(Hbiim)]^{2+}$, $C_{18}H_{17}CrN_{12}^{2+}$ (dark blue).

Peaks n°6+7:

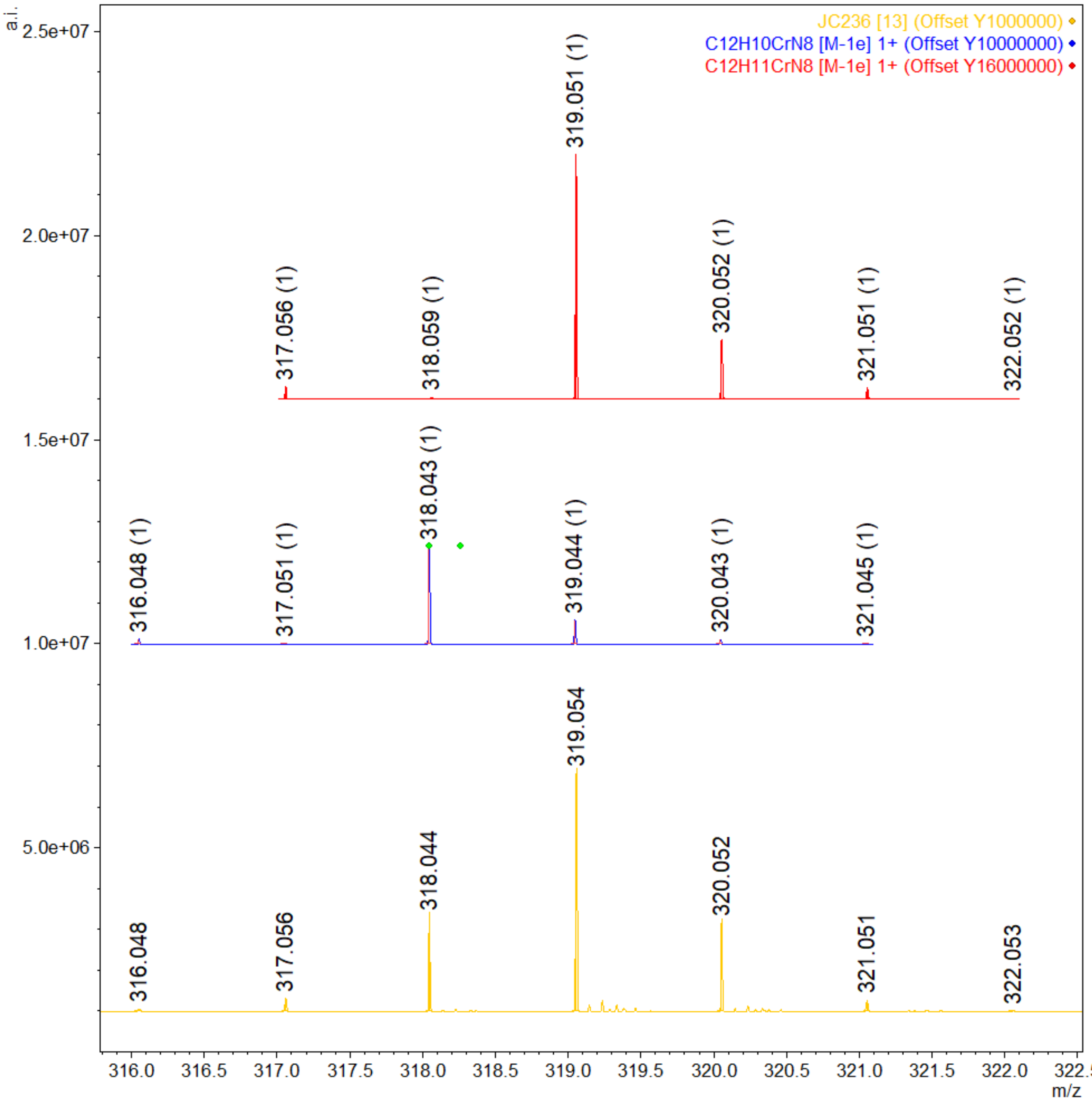

Figure S23 Experimental ESI-MS spectrum of $[Cr(H_2biim)_3](NO_3)_3$ in MeOH (orange). Calculated peaks for $[Cr(Hbiim)_2]^+$, $C_{12}H_{10}CrN_8^+$ (blue); and for $[Cr(H_2biim)(Hbiim)]^+$, $C_{12}H_{11}CrN_8^+$ (red).

Peak n°9:

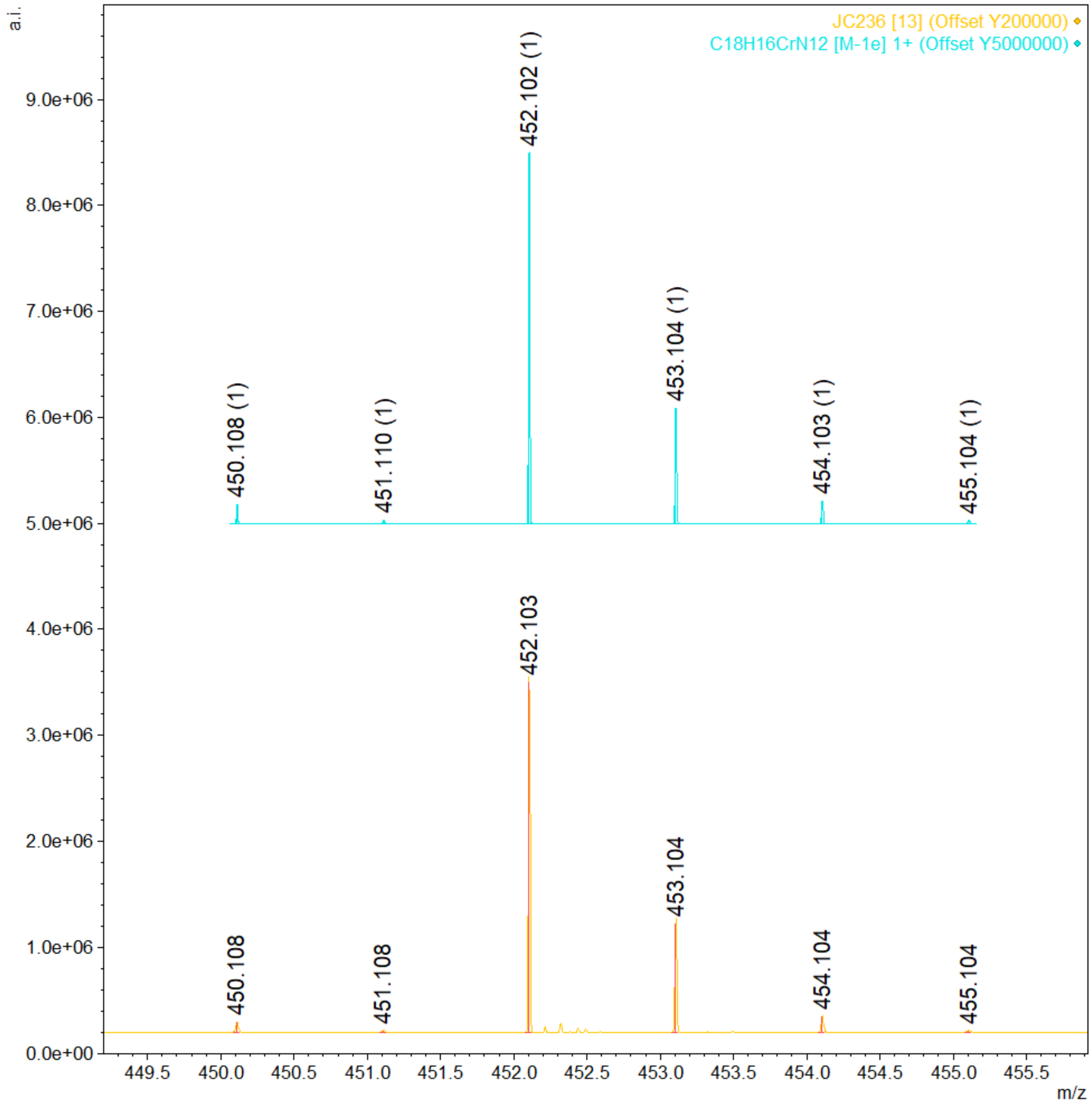

Figure S24 Experimental ESI-MS spectrum of $[Cr(H_2biim)_3](NO_3)_3$ in MeOH (orange). Calculated peaks for $[Cr(H_2biim)(Hbiim)_2]^{2+}$, $C_{18}H_{16}CrN_{12}^+$ (cyan).

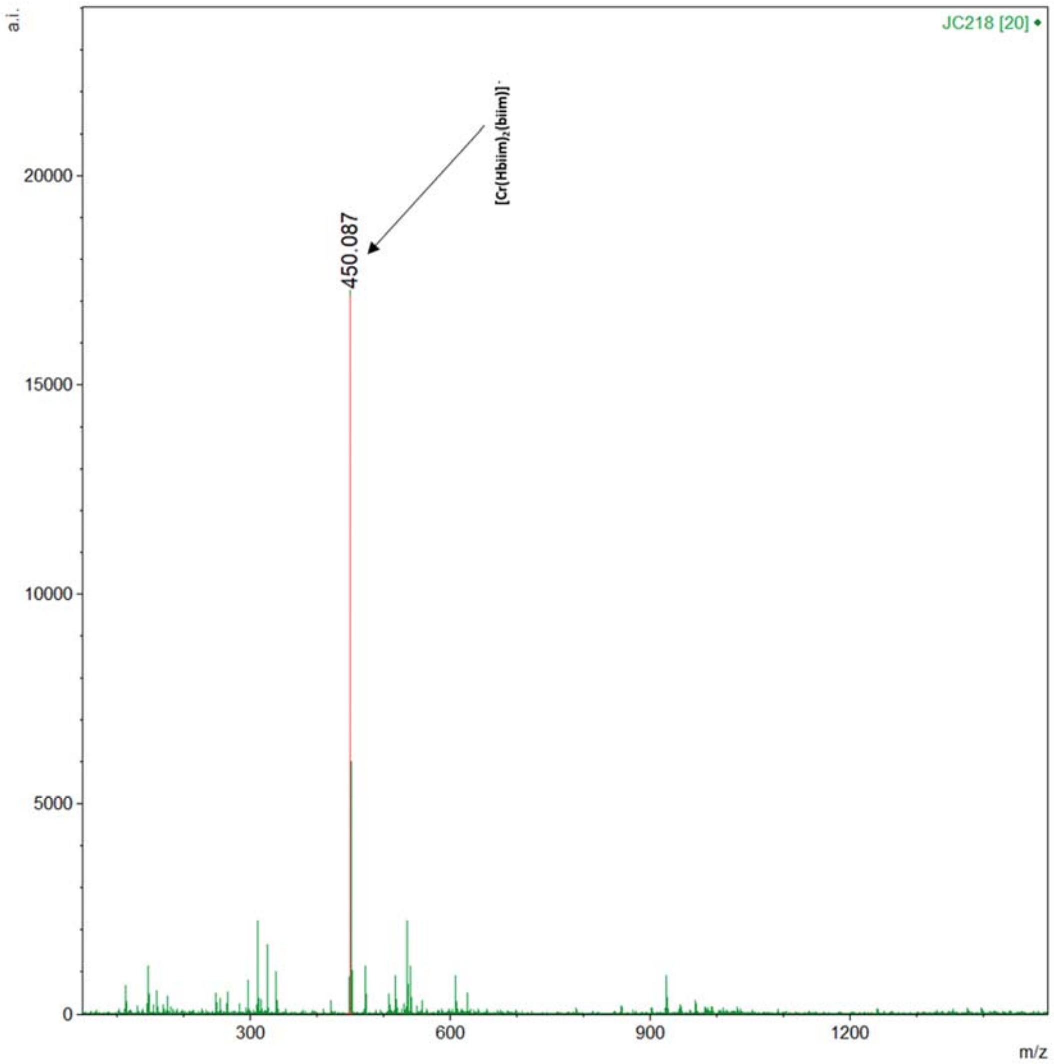

Figure S25 ESI-MS full spectrum of the complex [Cr(Hbiim)$_2$(biim)]PPh$_4$ in MeOH, negative mode.

Table S34 Observed m/z, intensity and attribution of each peak observed in the ESI-MS spectrum of [Cr(Hbiim)$_2$(biim)]PPh$_4$ in MeOH.

Peak n°	*m/z*	Intensity	Attribution	Molecular formula	Calc. *m/z*
1	450.087	1.71E+04	[Cr(Hbiim)$_2$(biim)]$^-$	$C_{18}H_{14}CrN_{12}^-$	450.0875

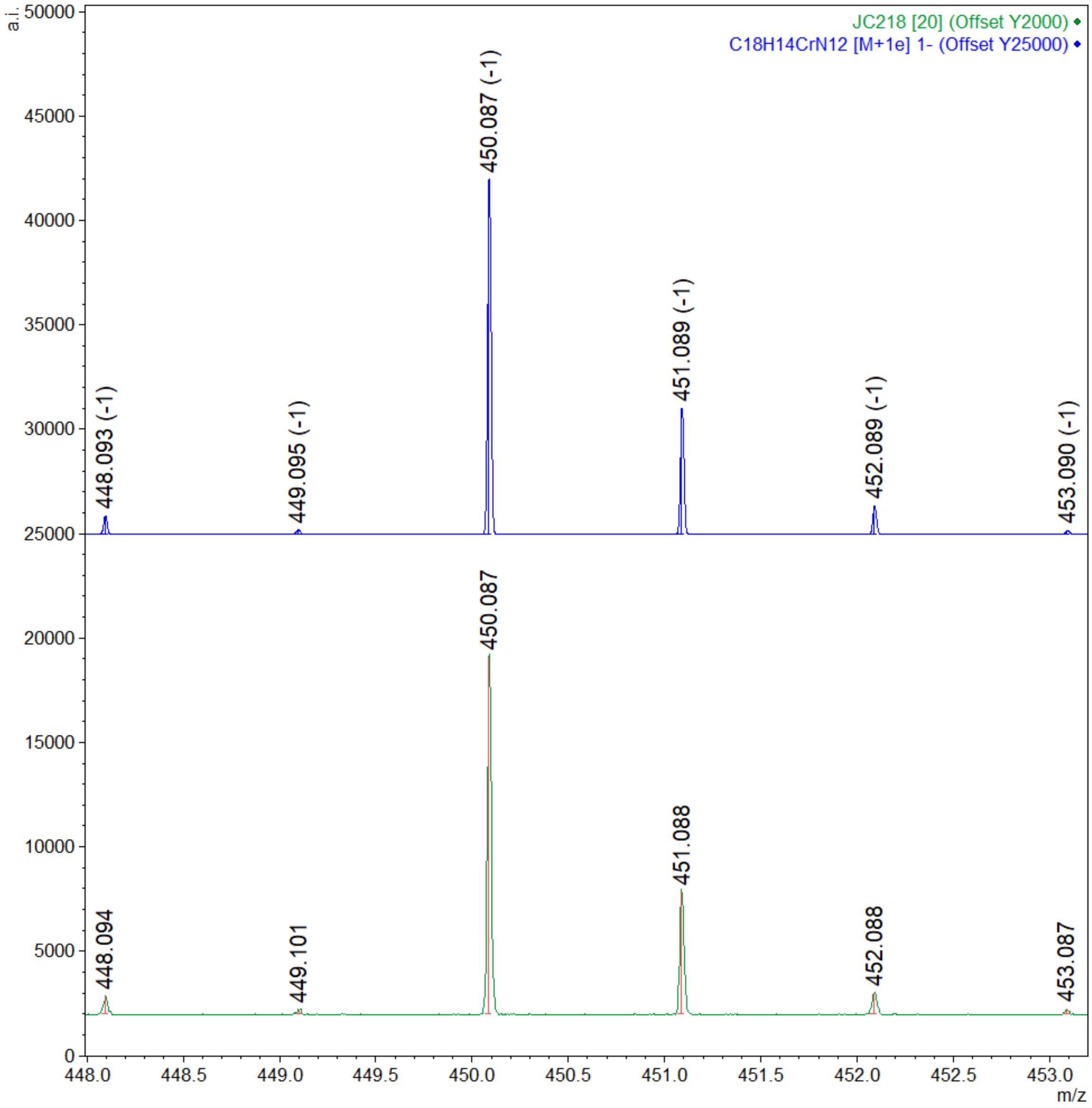

Figure S26 Experimental ESI-MS spectrum of $[Cr(Hbiim)_2(biim)]PPh_4$ in MeOH (green). Calculated peaks for $[Cr(Hbiim)_2(biim)]^-$, $C_{18}H_{14}CrN_{12}^-$ (blue).

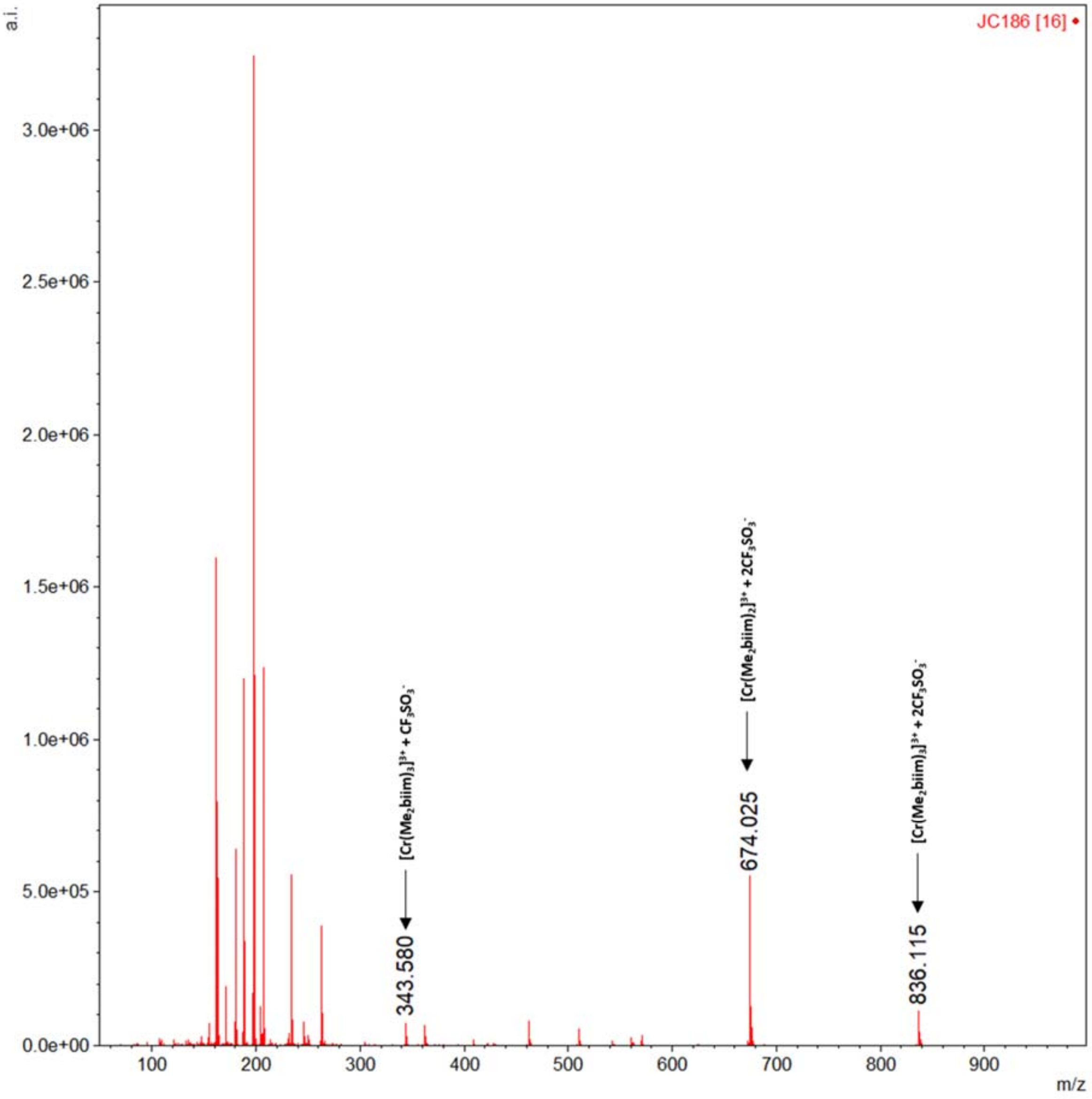
a.i.
JC186 [16]
3.0e+06
2.5e+06
2.0e+06
1.5e+06
1.0e+06
5.0e+05
0.0e+00
100
200
300
400
500
600
700
800
900
m/z
343.580
$[Cr(Me_2biim)_3]^{3+} + CF_3SO_3^-$
674.025
$[Cr(Me_2biim)_2]^{3+} + 2CF_3SO_3^-$
836.115
$[Cr(Me_2biim)_3]^{3+} + 2CF_3SO_3^-$

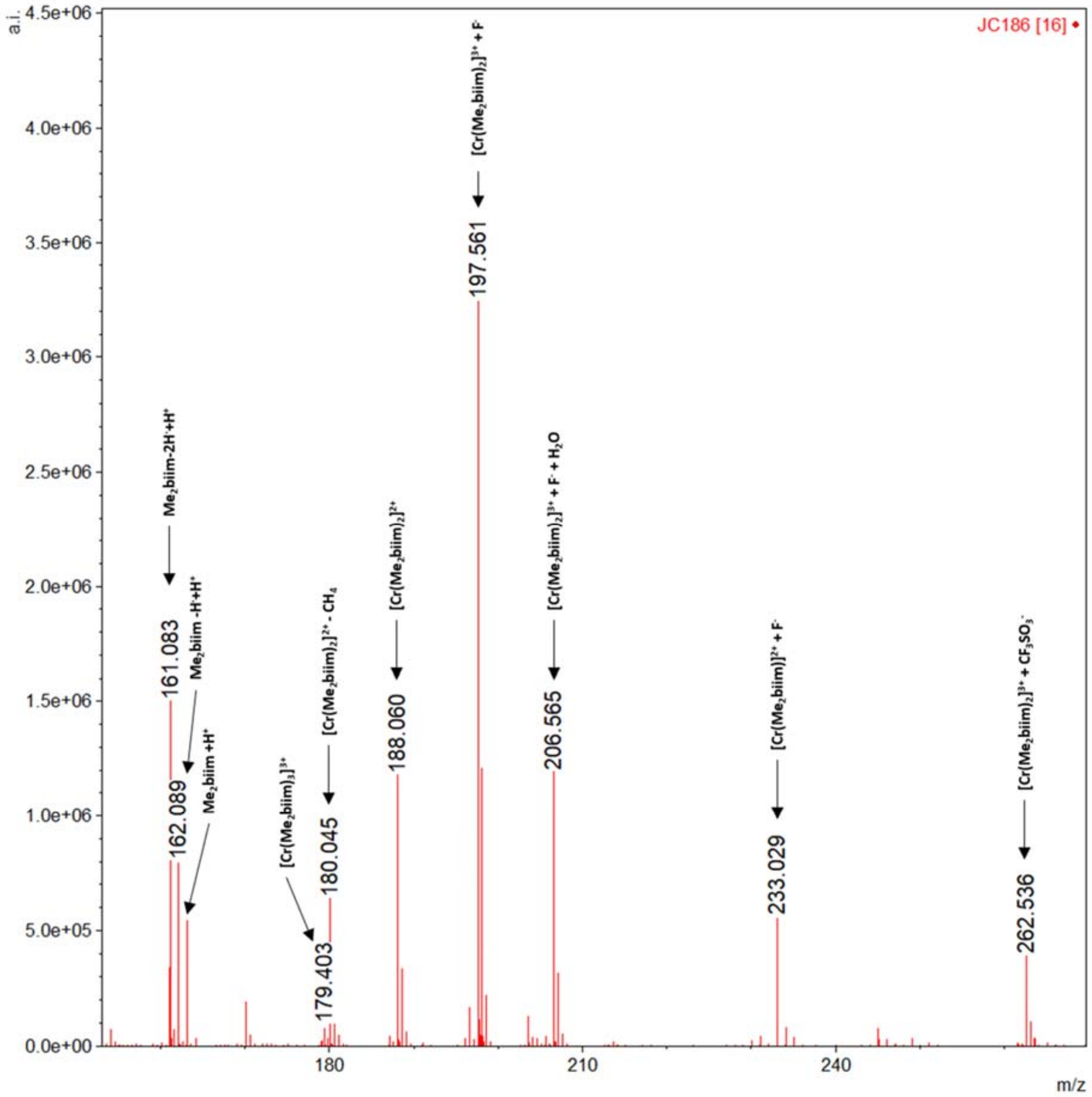

Figure S27 ESI-MS full spectrum of the complex $[Cr(Me_2biim)_3](CF_3SO_3)_3$ in CH_3CN (top), mode positive; zoomed spectrum (bottom).

Table S35 Observed m/z, intensity and attribution of each peak observed in the ESI-MS spectrum of $[Cr(Me_2biim)_3](CF_3SO_3)_3$ in CH_3CN.

Peak n°	*m*/*z*	Intensity	Attribution	Molecular formula	Calc. *m*/*z*
1	161.083	1.48E+06	Me_2biim-2H$^{\cdot}$+H^+	$C_8H_9N_4^+$	161.0822
2	162.089	7.82E+05	Me_2biim -H$^{\cdot}$+H^+	$C_8H_{10}N_4^+$	162.0900
3	163.097	5.36E+05	Me_2biim +H^+	$C_8H_{11}N_4^+$	163.0978
4	179.403	7.27E+04	$[Cr(Me_2biim)_3]^{3+}$	$C_{24}H_{30}CrN_{12}^{3+}$	179.4035
5	180.045	6.19E+05	$[Cr(Me_2biim)_2]^{2+}$ - CH_4	$C_{15}H_{16}CrN_8^{2+}$	180.0446
6	188.060	1.16E+06	$[Cr(Me_2biim)_2]^{2+}$	$C_{16}H_{20}CrN_8^{2+}$	188.0602
7	197.561	3.23E+06	$[Cr(Me_2biim)_2]^{3+}$ + F^-	$C_{16}H_{20}CrFN_8^{2+}$	197.5595
8	206.565	1.17E+06	$[Cr(Me_2biim)_2]^{3+}$ + F^- + H_2O	$C_{16}H_{22}CrFN_8O^{2+}$	206.5647
9	233.029	5.42E+05	$[Cr(Me_2biim)]^{2+}$ + F^-	$C_8H_{10}CrFN_4^+$	233.0289
10	262.536	3.75E+05	$[Cr(Me_2biim)_2]^{3+}$ + $CF_3SO_3^-$	$C_{17}H_{20}CrF_3N_8O_3S^{2+}$	262.5363
11	343.580	7.34E+04	$[Cr(Me_2biim)_3]^{3+}$ + $CF_3SO_3^-$	$C_{25}H_{30}CrF_3N_{12}O_3S^{2+}$	343.5816
12	674.025	5.48E+05	$[Cr(Me_2biim)_2]^{3+}$ + $2CF_3SO_3^-$	$C_{18}H_{20}CrF_6N_8O_6S_2^+$	674.0251
13	836.115	1.09E+05	$[Cr(Me_2biim)_3]^{3+}$ + $2CF_3SO_3^-$	$C_{26}H_{30}CrF_6N_{12}O_6S_2^+$	836.1157

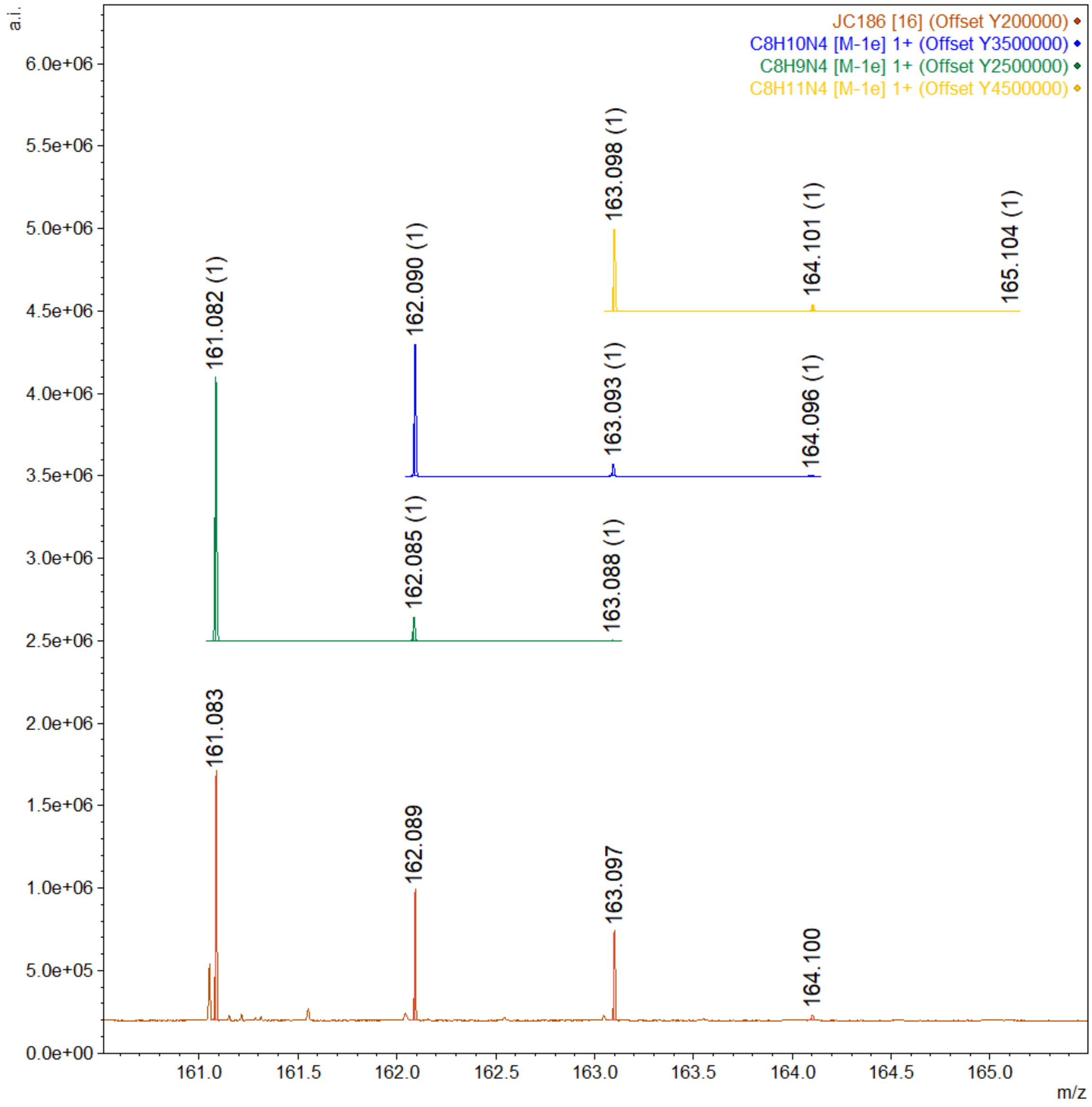

Figure S28 Experimental ESI-MS spectrum of [Cr(Me$_2$biim)$_3$](CF$_3$SO$_3$)$_3$ in CH$_3$CN (red). Calculated peaks for Me$_2$biim-2H$^{\cdot}$+H$^+$, $C_8H_9N_4^+$ (green); for Me$_2$biim -H$^{\cdot}$+H$^+$, $C_8H_{10}N_4^+$ (blue); and for Me$_2$biim +H$^+$, $C_8H_{11}N_4^+$ (orange).

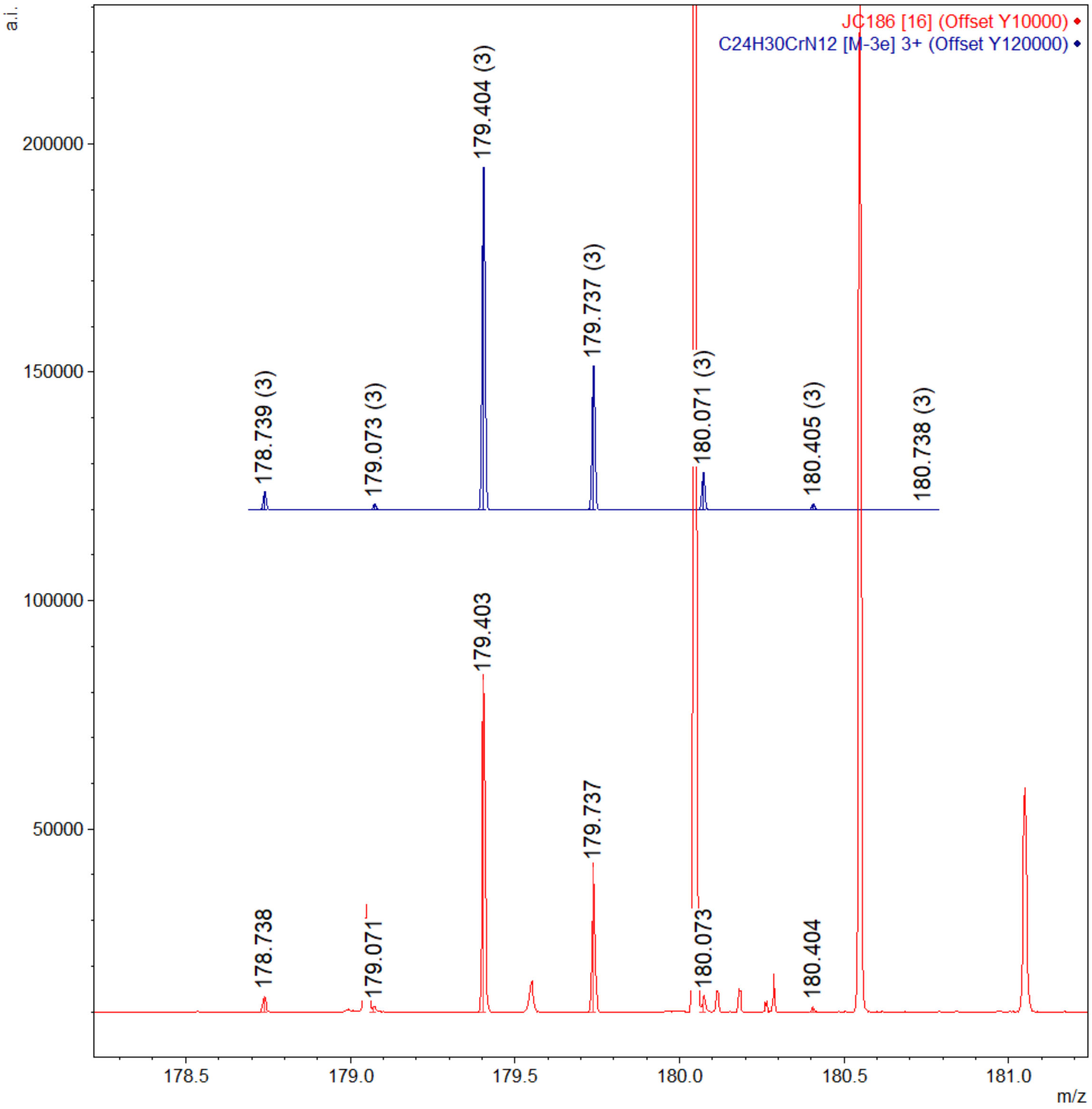

Figure S29 Experimental ESI-MS spectrum of $[Cr(Me_2biim)_3](CF_3SO_3)_3$ in CH_3CN (red). Calculated peaks for $[Cr(Me_2biim)_3]^{3+}$, $C_{24}H_{30}CrN_{12}^{3+}$ (dark blue).

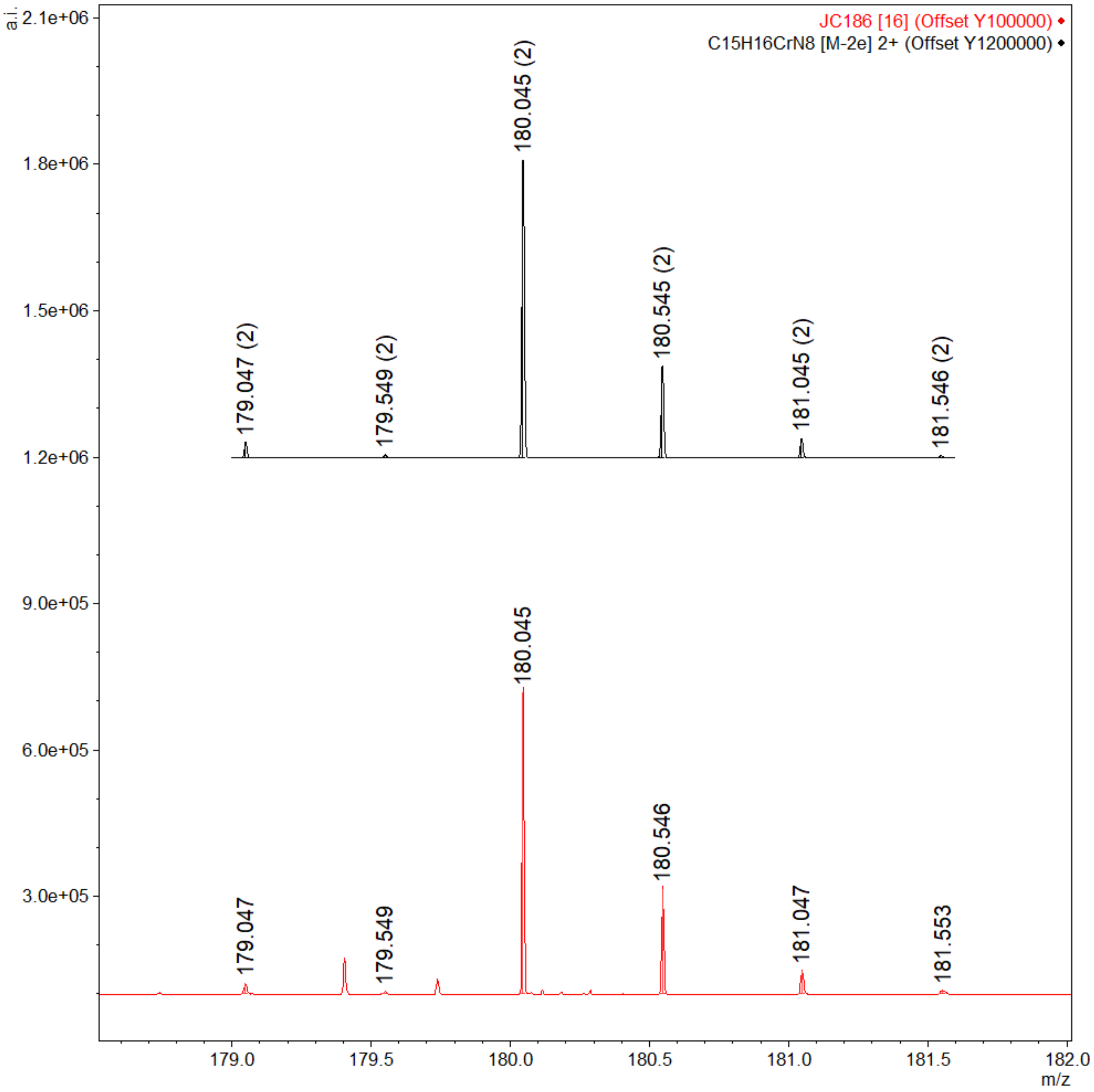

Figure S30 Experimental ESI-MS spectrum of [Cr(Me_2biim)$_3$](CF_3SO_3)$_3$ in CH_3CN (red). Calculated peaks for [Cr(Me_2biim)$_2$]$^{2+}$ - CH_4, $C_{15}H_{16}CrN_8^{2+}$ (black).

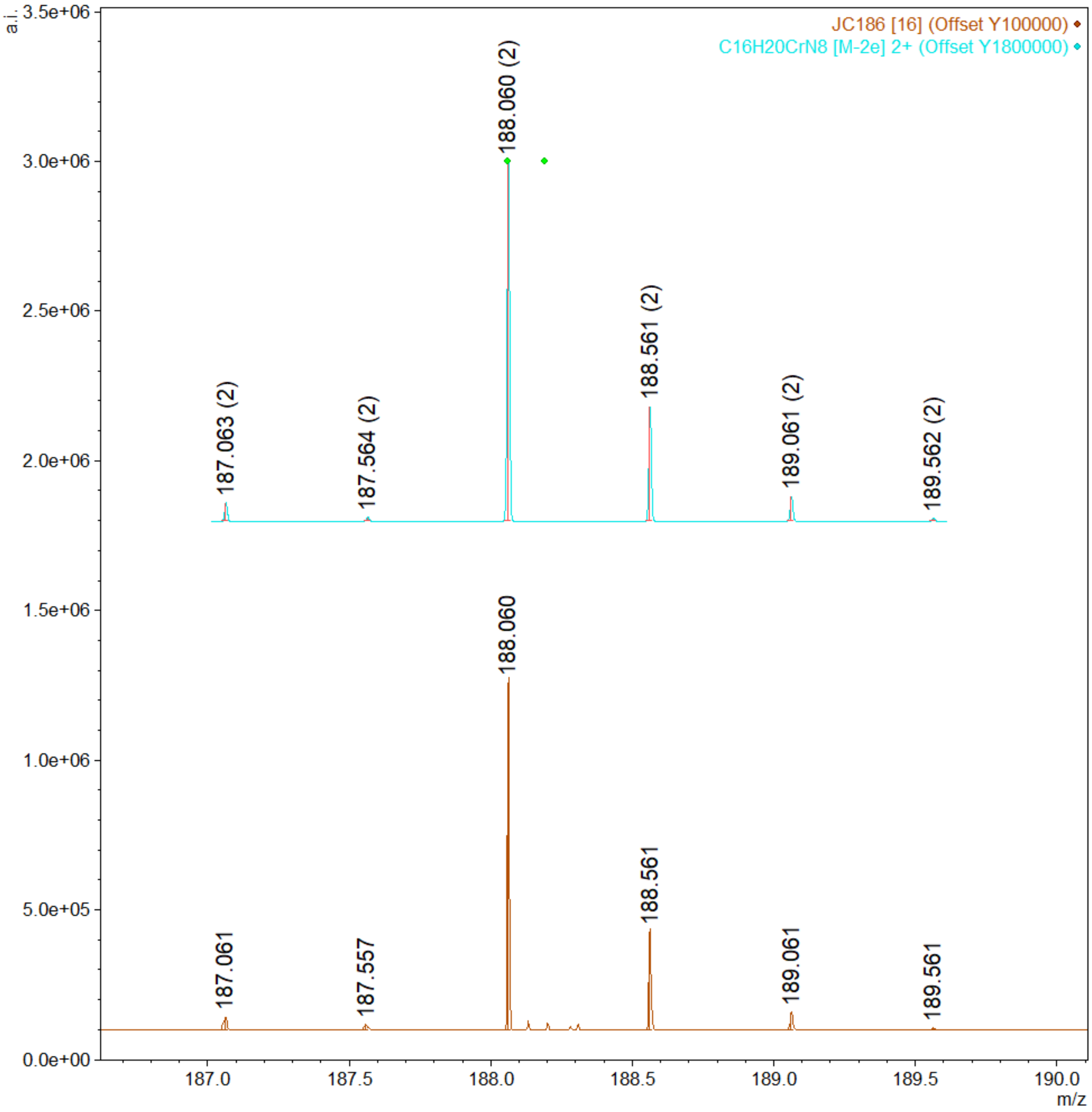

Figure S31 Experimental ESI-MS spectrum of $[Cr(Me_2biim)_3](CF_3SO_3)_3$ in CH_3CN (red). Calculated peaks for $[Cr(Me_2biim)_2]^{2+}$, $C_{16}H_{20}CrN_8^{2+}$ (cyan).

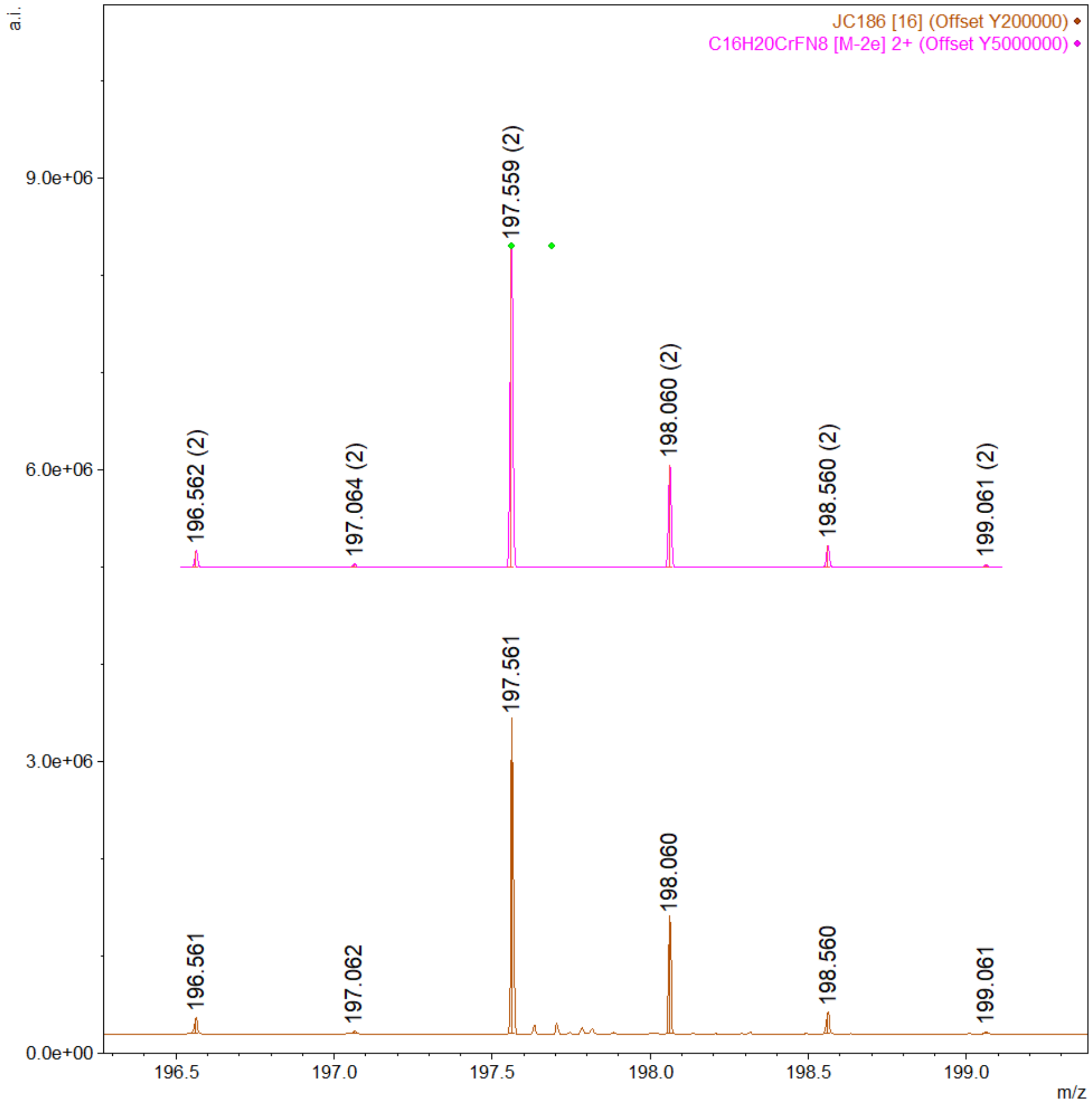

Figure S32 Experimental ESI-MS spectrum of $[Cr(Me_2biim)_3](CF_3SO_3)_3$ in CH_3CN (red). Calculated peaks for $[Cr(Me_2biim)_2]^{3+} + F^-$, $C_{16}H_{20}CrFN_8^{2+}$ (purple).

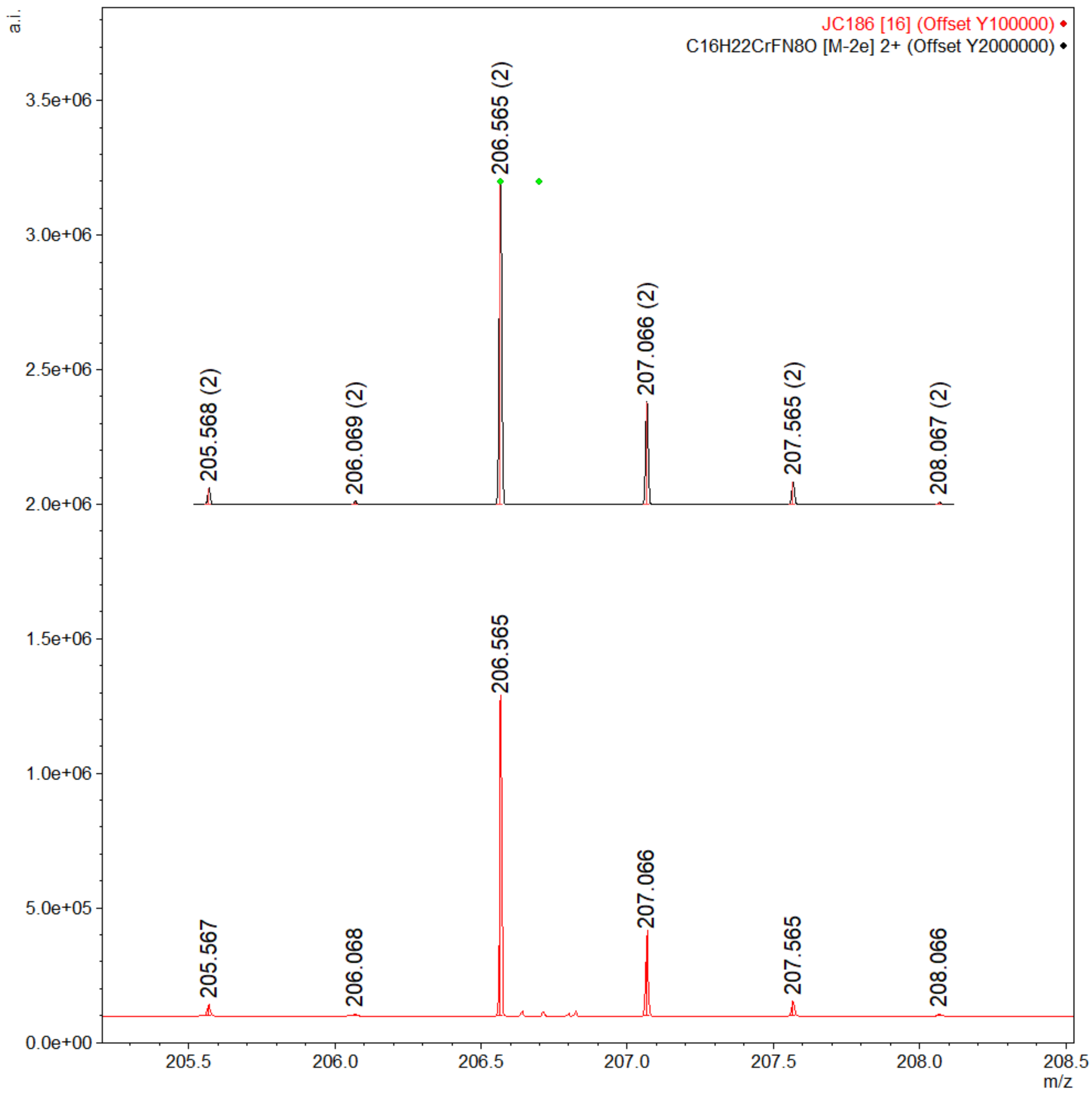

Figure S33 Experimental ESI-MS spectrum of [Cr(Me_2biim)$_3$](CF_3SO_3)$_3$ in CH_3CN (red). Calculated peaks for [Cr(Me_2biim)$_2$]$^{3+}$ + F^- + H_2O, $C_{16}H_{22}CrFN_8O^{2+}$ (black).

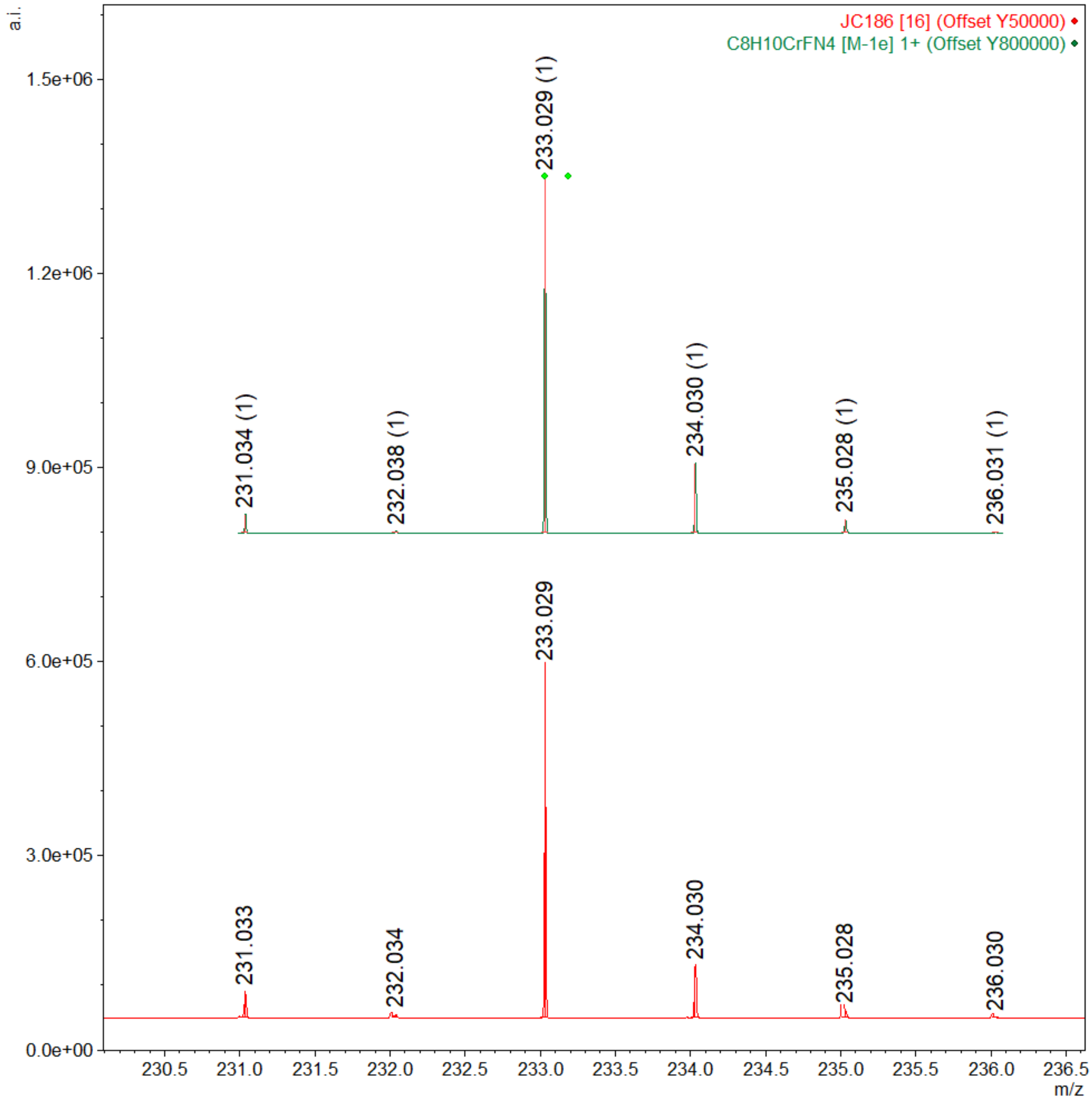

Figure S34 Experimental ESI-MS spectrum of $[Cr(Me_2biim)_3](CF_3SO_3)_3$ in CH_3CN (red). Calculated peaks for $[Cr(Me_2biim)]^{2+} + F^-$, $C_8H_{10}CrFN_4^+$ (green).

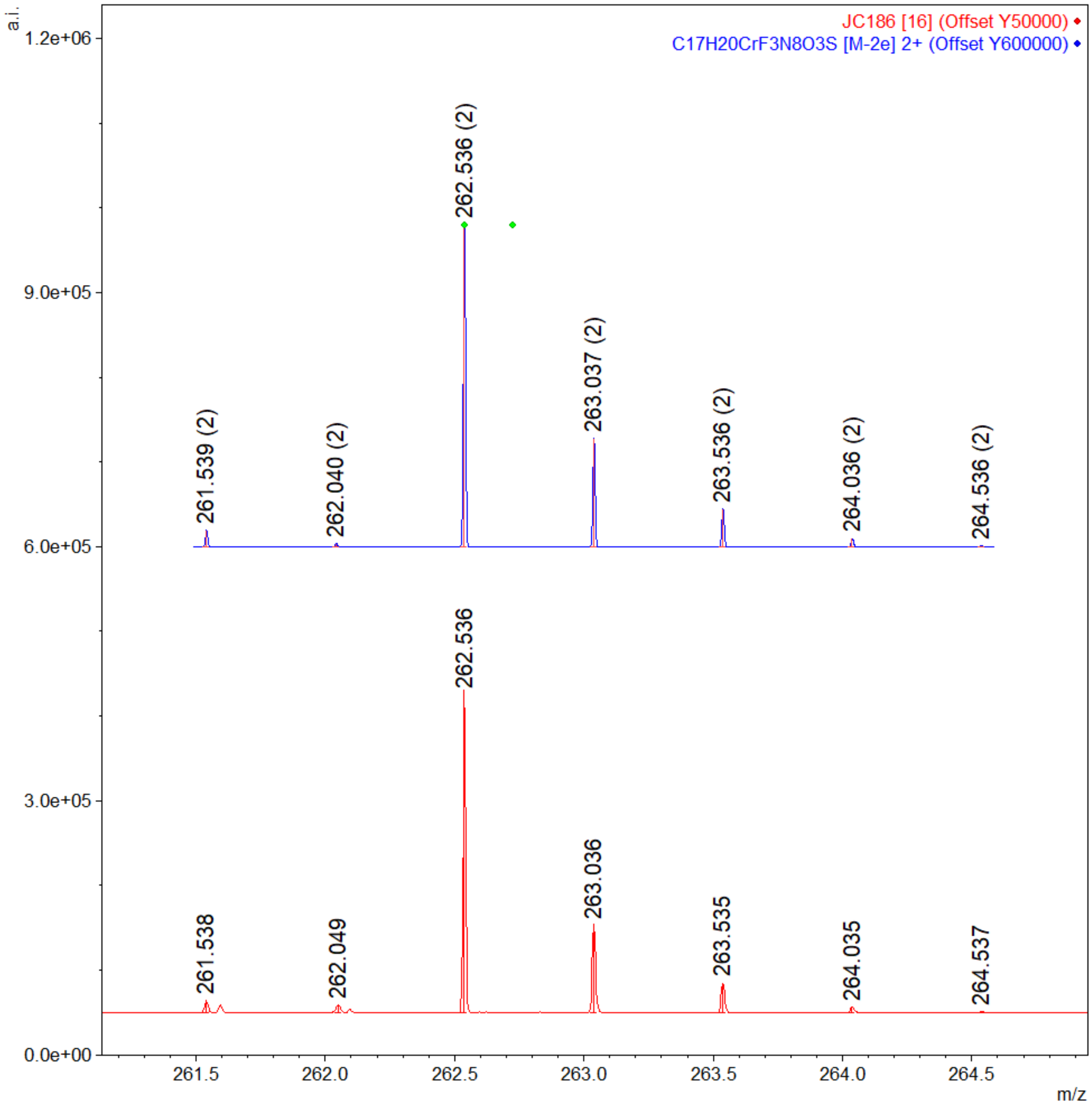

Figure S35 Experimental ESI-MS spectrum of $[Cr(Me_2biim)_3](CF_3SO_3)_3$ **in** CH_3CN (red). Calculated peaks for $[Cr(Me_2biim)_2]^{3+}$ + $CF_3SO_3^-$, $C_{17}H_{20}CrF_3N_8O_3S^{2+}$ (blue).

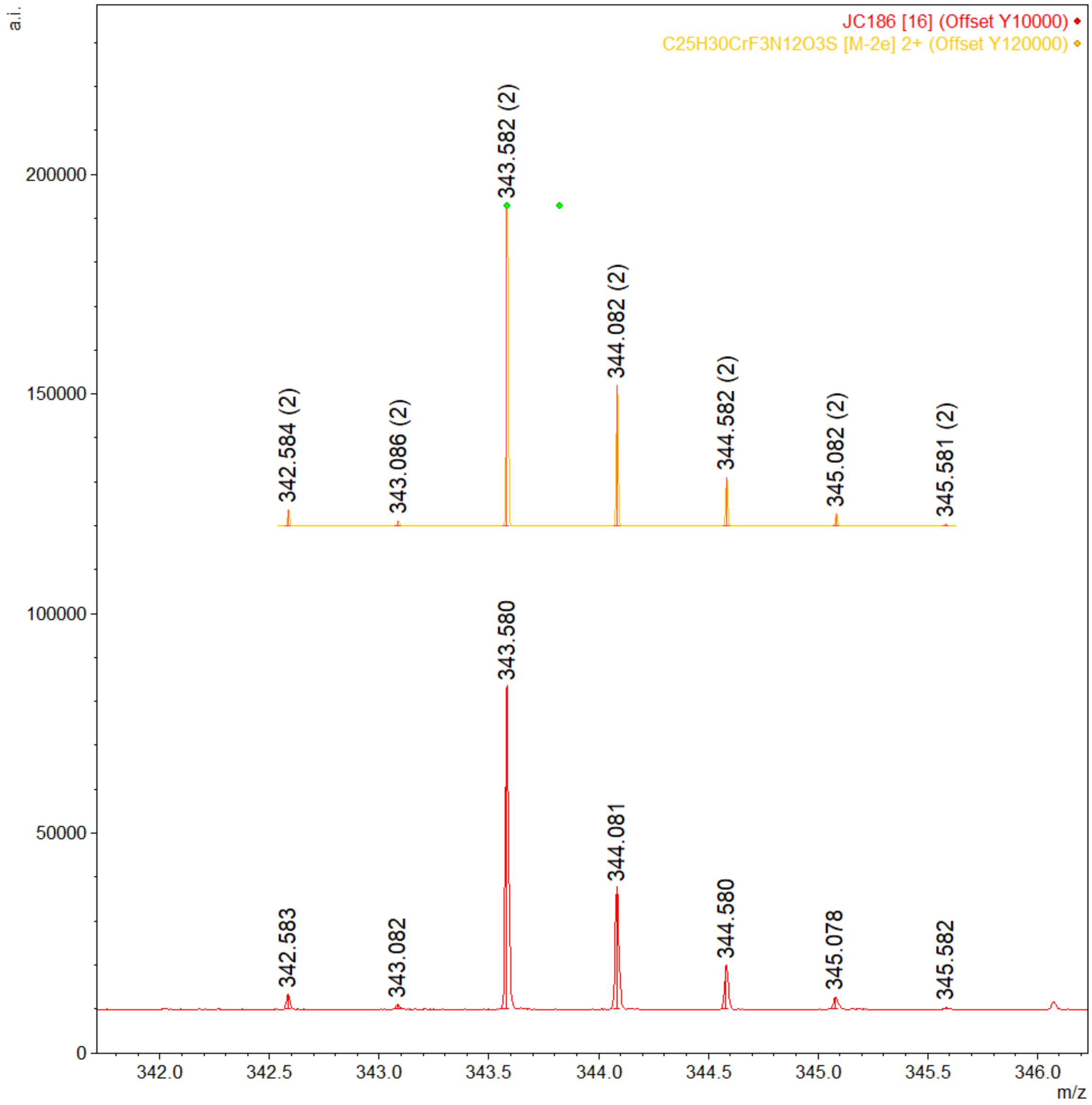

Figure S36 Experimental ESI-MS spectrum of $[Cr(Me_2biim)_3](CF_3SO_3)_3$ in CH_3CN (red). Calculated peaks for $[Cr(Me_2biim)_3]^{3+}$ + $CF_3SO_3^-$, $C_{25}H_{30}CrF_3N_{12}O_3S^{2+}$ (orange).

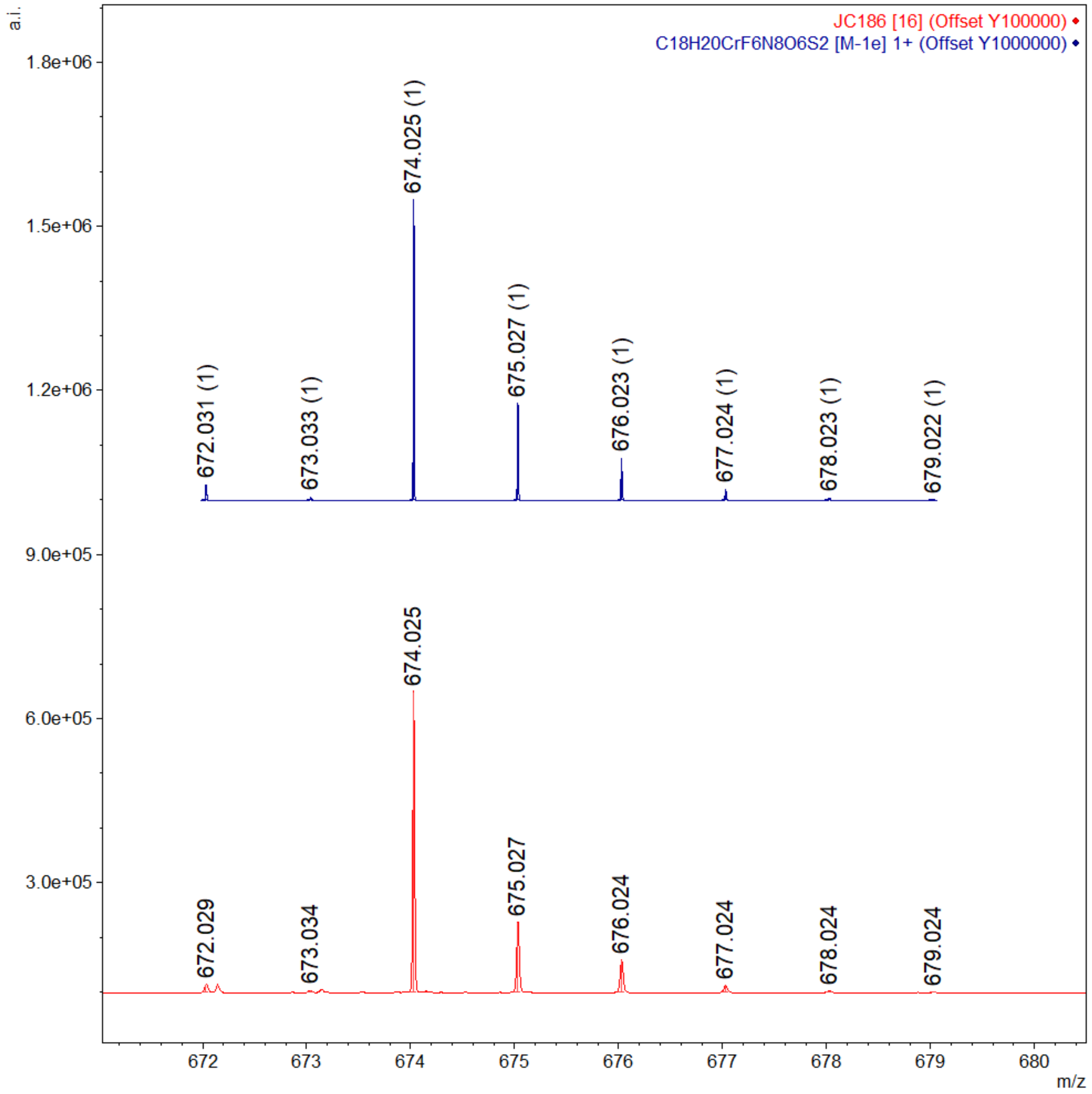

Figure S37 Experimental ESI-MS spectrum of $[Cr(Me_2biim)_3](CF_3SO_3)_3$ in CH_3CN (red). Calculated peaks for $[Cr(Me_2biim)_2]^{3+} + 2CF_3SO_3^-$, $C_{18}H_{20}CrF_6N_8O_6S_2^+$ (dark blue).

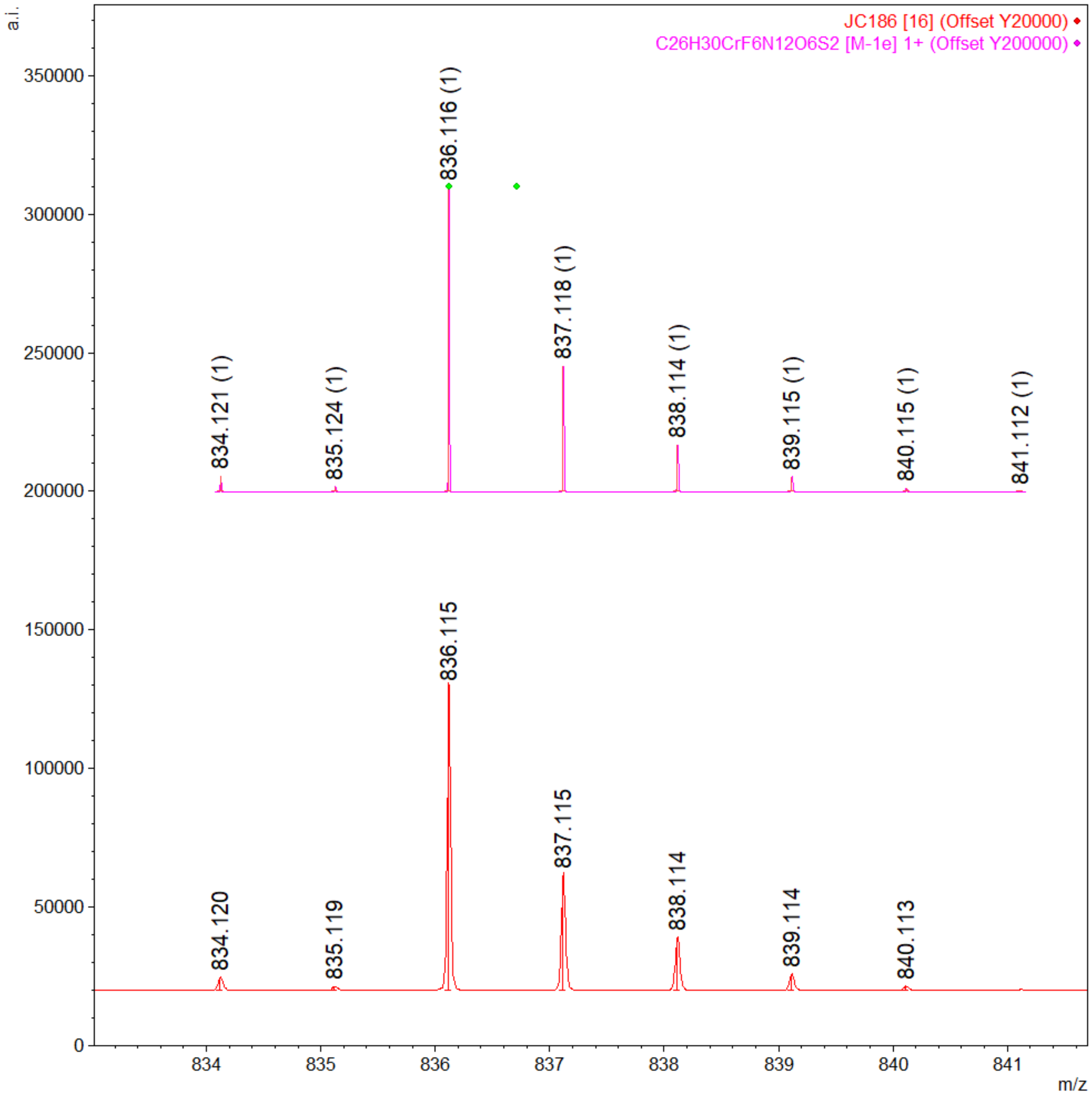

Figure S38 Experimental ESI-MS spectrum of $[Cr(Me_2biim)_3](CF_3SO_3)_3$ in CH_3CN (red). Calculated peaks for $[Cr(Me_2biim)_3]^{3+} + 2CF_3SO_3^-$, $C_{26}H_{30}CrF_6N_{12}O_6S_2^+$ (purple).

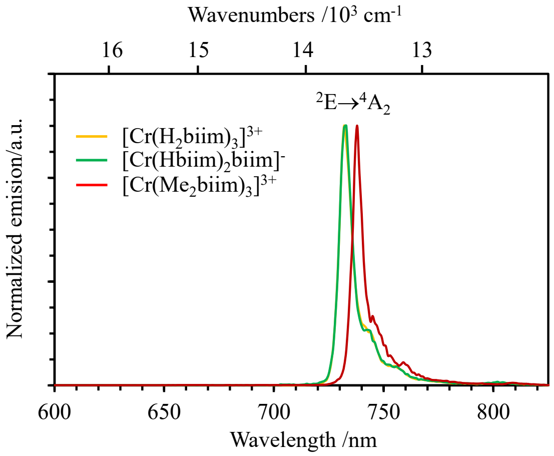

Figure S39 NIR emission spectra (λ_{exc} = 330 nm) of complexes $[Cr(H_2biim)_3](NO_3)_3$ (orange), $[Cr(Hbiim)_2(biim)]PPh_4$ (green) and $[Cr(Me_2biim)_3](CF_3SO_3)_3$ (red) in MeOH:H_2O (1:1) at 77 K.

a)

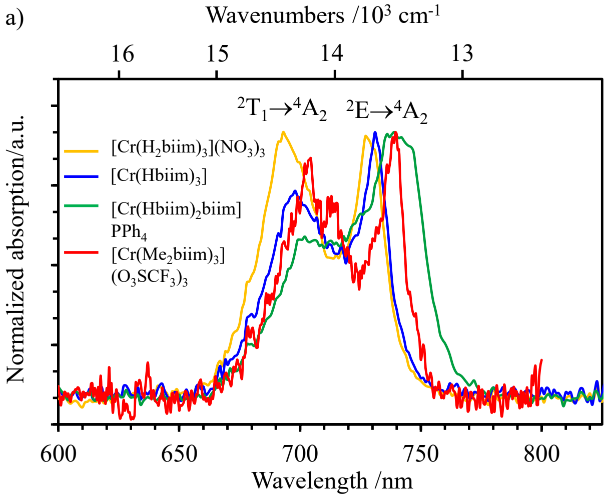

b)

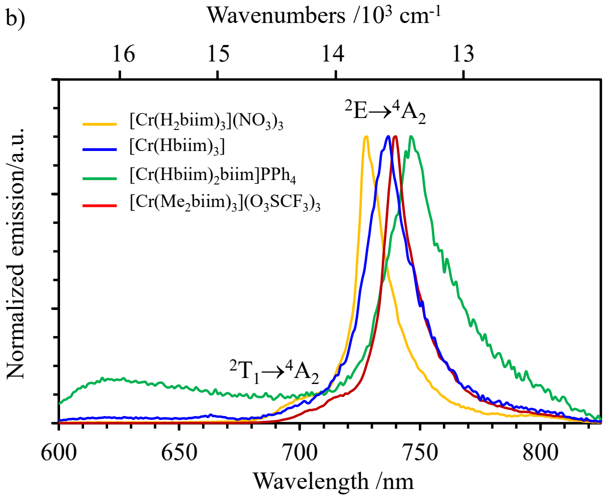

Figure S40 NIR a) absorption and b) emission spectra (λ_{exc} = 330 nm) of complexes $[Cr(H_2biim)_3](NO_3)_3$ (orange), $[Cr(Hbiim)_2(biim)]PPh_4$ (green), $[Cr(Me_2biim)_3](CF_3SO_3)_3$ (red) and $[Cr(H_2biim)_3]$ (blue) in the solid state at 293 K.

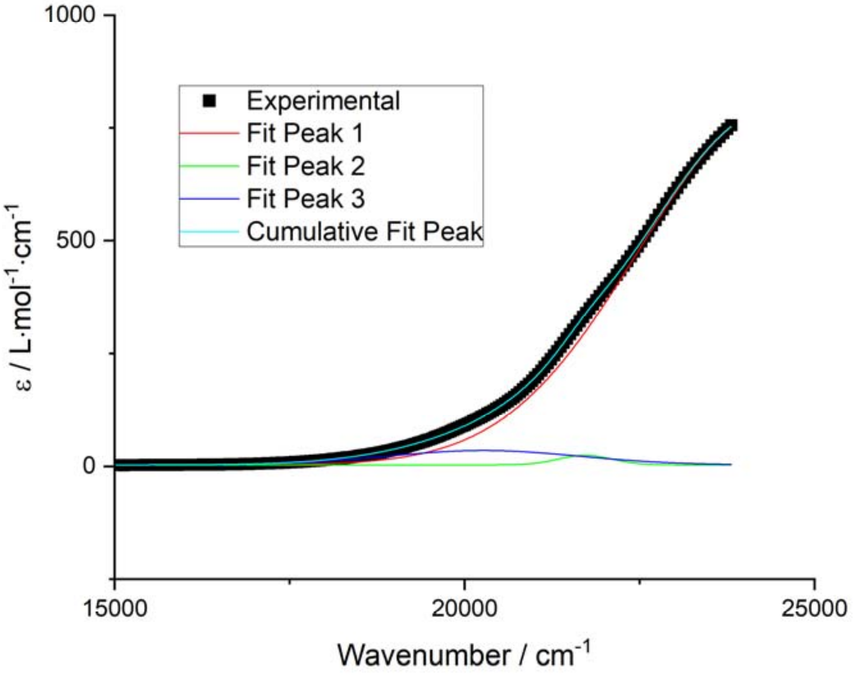

Figure S41 Experimental absorption spectrum of $[Cr(H_2biim)_3](NO_3)_3$ in MeOH ($3.09 \cdot 10^{-4}$ M) in the visible region showing the Gaussian deconvolution fit with 3 peaks. Peak 3 is attributed to the transition $^4T_2 \leftarrow {}^4A_2$.

Table S36 Fitting parameters for the Gaussian deconvolution of the absorption spectrum of $[Cr(H_2biim)_3](NO_3)_3$ in MeOH.

Model	Gaussian		
Equation	$$y = y_0 + \frac{A}{w\sqrt{\frac{\pi}{4\ln(2)}}} \cdot e^{\frac{-4\ln(2)\cdot(x-x_c)^2}{w^2}}$$		
Plot	Peak 1	Peak 2	Peak 3
y_0 / L.mol^{-1}.cm^{-1}	2.84	2.84	2.84
x_c / cm^{-1}	24406 ± 22	21722 ± 6	20268 ±106
A / L.mol^{-1}.cm^{-2}	3.77E6 ± 6.62E5	2.06E4 ± 1.12E3	1.10E5 ± 1.47E4
W / cm^{-1}	4512 ± 64	907 ± 24	3231 ±85
Reduced Chi-square	0.42217		
R-Square (COD)	0.99999		
Adj. R-Square	0.99999		

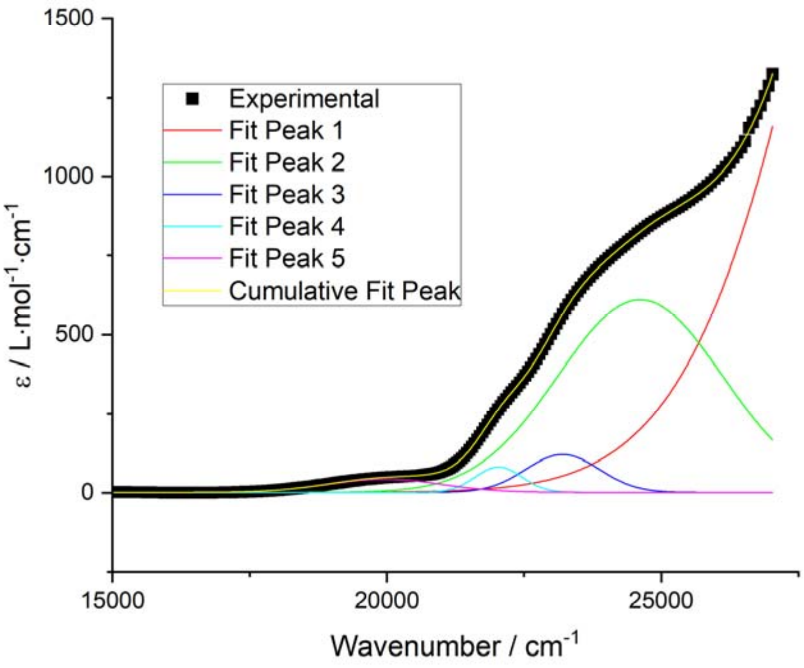

Figure S42 Experimental absorption spectrum of [Cr(Hbiim)$_2$(biim)]PPh$_4$ in MeOH (1.50 · 10^{-4} M) in the visible region showing the Gaussian deconvolution fit with 5 peaks. Peak 5 is attributed to the transition $^4T_2 \leftarrow {}^4A_2$.

Table S37 Fitting parameters for the Gaussian deconvolution of the absorption spectrum of [Cr(Hbiim)$_2$(biim)]PPh$_4$ in MeOH.

Model	Gaussian				
Equation	$$y = y_0 + \frac{A}{w\sqrt{\frac{\pi}{4\ln(2)}}} \cdot e^{\frac{-4\ln(2)\cdot(x-x_c)^2}{w^2}}$$				
Plot	Peak 1	Peak 2	Peak 3	Peak 4	Peak 5
y_0 / L.mol^{-1}.cm^{-1}	1.0	1.0	1.0	1.0	1.0
x_c / cm^{-1}	32019 ± 5270	24615 ± 166	23201 ± 12	22036 ± 10	19956 ± 40
A / L.mol^{-1}.cm^{-2}	3.66E7 ± 7.26E7	2.29E4 ± 7.22E5	2.02E5 ± 1.62E4	8.57E4 ± 6.70E3	1.09E5 ± 6.43E3
W / cm^{-1}	6873 ± 4247	3524 ± 241	1585 ± 49	1004 ± 24	2463 ± 55
Reduced Chi-square	1.4505				
R-Square (COD)	0.99999				
Adj. R-Square	0.99999				

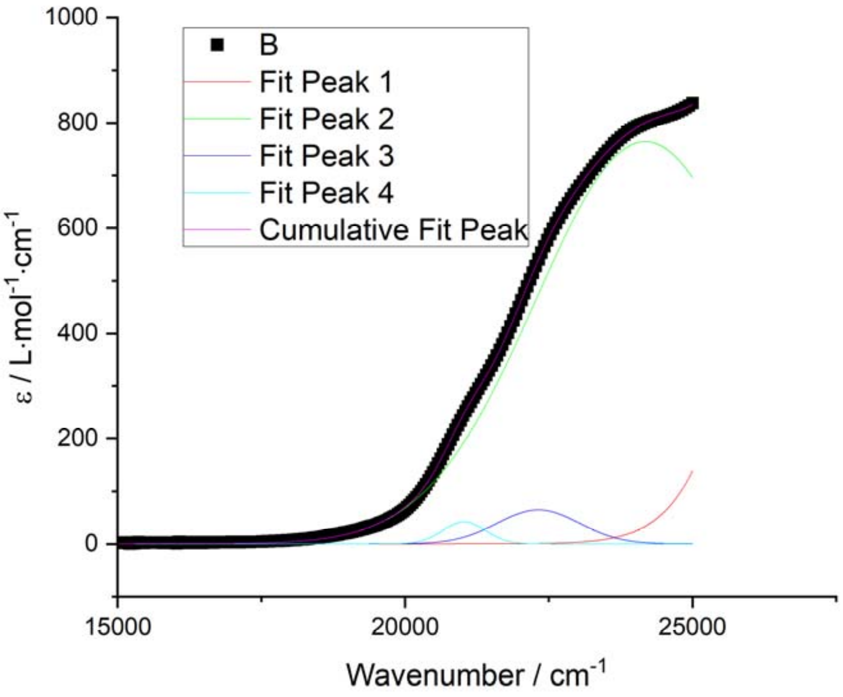

Figure S43 Experimental absorption spectrum of $[Cr(Me_2biim)_3](CF_3SO_3)_3$ in MeOH ($1.85 \cdot 10^{-4}$ M) in the visible region showing the Gaussian deconvolution fit with 4 peaks. Peak 4 is attributed to the transition $^4T_2 \leftarrow {}^4A_2$.

Table S38 Fitting parameters for the Gaussian deconvolution of the absorption spectrum of $[Cr(Me_2biim)_3](CF_3SO_3)_3$ in MeOH.

Model	Gaussian			
Equation	$$y = y_0 + \frac{A}{w\sqrt{\frac{\pi}{4\ln(2)}}} \cdot e^{\frac{-4\ln(2)\cdot(x-x_c)^2}{w^2}}$$			
Plot	Peak 1	Peak 2	Peak 3	Peak 4
y_0 / L.mol^{-1}.cm^{-1}	0	0	0	0
x_c / cm^{-1}	30354 ± 0	24176 ± 10	22318 ±20	21026 ±16
A / L.mol^{-1}.cm^{-2}	4.69E7 ± 0	3.64E6 ± 7.40E3	1.16E5 ± 8.63E4	3.94E4 ± 3.31E3
w / cm^{-1}	4298 ± 0	4475 ± 13	1696 ± 78	888 ± 37
Reduced Chi-square	5.94263			
R-Square (COD)	0.99993			
Adj. R-Square	0.99993			

Table S39 Energy (x_c), maximum absorption coefficient (ε_{max}) and band width (w) of the Cr($^4T_2$←$^4A_2$) transitions in [Cr(H_2biim)$_3$](NO_3)$_3$, [Cr(Hbiim)$_2$(biim)]PPh_4 and [Cr(Me_2biim)$_3$](CF_3SO_3)$_3$.

Complex	[Cr(H_2biim)$_3$](NO_3)$_3$	[Cr(Hbiim)$_2$(biim)]PPh_4	[Cr(Me_2biim)$_3$](CF_3SO_3)$_3$
x_c ($^4A_2$→$^4T_2$) / cm^{-1}	20268	19956	21026
ε_{max} ($^4A_2$→$^4T_2$) / $M^{-1}\cdot cm^{-1}$	32.0	41.6	41.7
w / cm^{-1}	3231	2463	888

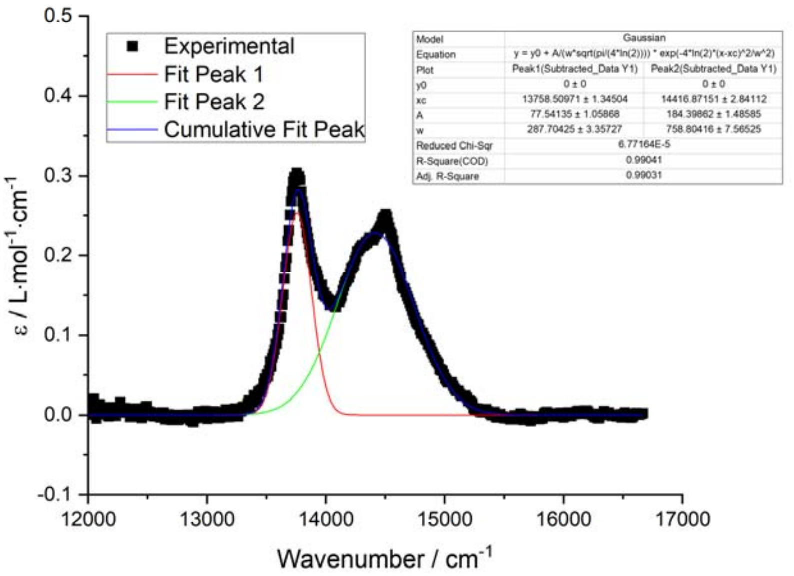

Figure S44 Gaussian deconvolution of the absorption spectrum of $[Cr(H_2biim)_3](NO_3)_3$ in H_2O, c = $1.22 \cdot 10^{-2}$ M.

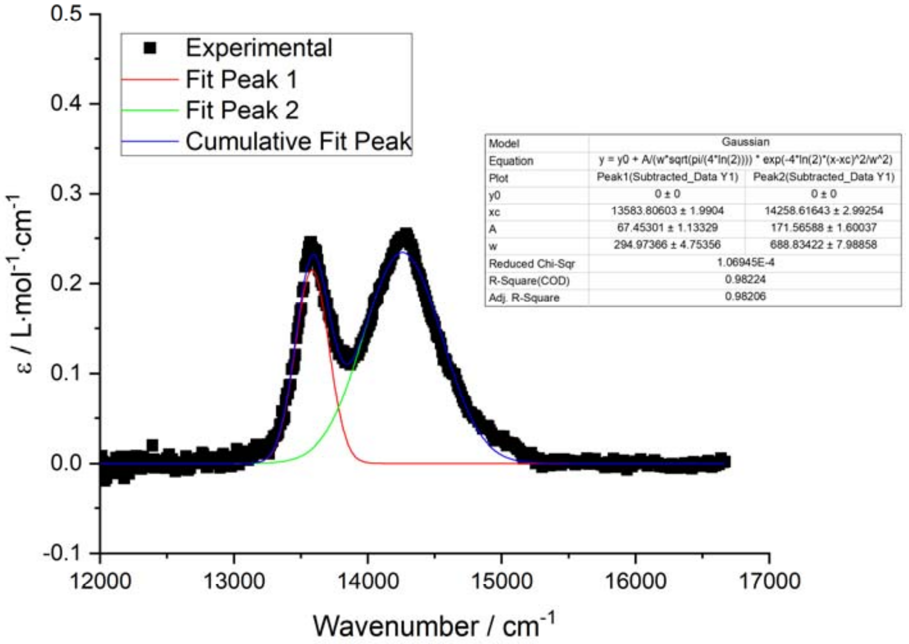

Figure S45 Gaussian deconvolution of the absorption spectrum of $[Cr(Me_2biim)_3](CF_3SO_3)_3$ in CH_3CN, $c = 8.93 \cdot 10^{-3}$ M.

Table S40 Energies $\tilde{\nu}$ obtained with the Gaussian deconvolution of the absorption spectra in solution and radiative rate constant k_{rad} obtained with equation for $[Cr(H_2biim)_3](NO_3)$ and $[Cr(Me_2biim)_3](CF_3SO_3)_3$ together with a selection of complexes reported in literature.[a]

Complex	$\tilde{\nu}(^2E)$ / cm^{-1}	$\tilde{\nu}(^2T_1)$ / cm^{-1}	$\varepsilon_{max}(^2E)$/ M$^{-1}\cdot$cm^{-1}	$\varepsilon_{max}(^2T_1)$ / M$^{-1}\cdot$cm^{-1}	$k_{rad}(^2E)$ / s^{-1}	$k_{rad}(^2T_1)$ / s^{-1}	Ref.
$[Cr(H_2biim)_3](NO_3)_3$	13758	14416	0.27	0.22	75	119	This work
$[Cr(Me_2biim)_3](CF_3SO_3)_3$	13583	14258	0.24	0.25	73	123	This work
$[Cr(phen)_2(H_2biim)](CF_3SO_3)_3$	13723	14381	0.19	0.27	65	135	This work
$[Cr(phen)_2(phen\text{-}bbzpy)](CF_3SO_3)_3$	13725	14492	0.16	0.17	33	43	21
$[Cr(dqp)_2](CF_3SO_3)_3$	13354	13758	0.1	0.58	30	89	24,76
$[Cr(dqp\text{-}OMe)_2](CF_3SO_3)_3$	13113	13702	0.07	0.32	31	110	77
$[Cr(dqp\text{-}Br)_2](CF_3SO_3)_3$	13271	13730	0.09	0.32	34	77	77
$[Cr(dqp\text{-}CCH)_2](CF_3SO_3)_3$	13290	13710	0.14	0.43	29	88	77
$[Cr(dqp)(tpy)](CF_3SO_3)_3$	13351	14326	0.15	0.18	55	58	76
$[Cr(ddpd)(dqp)](CF_3SO_3)_3$	13097	13661	0.23	0.30	63	91	76
$[Cr(dqp)(dqp\text{-}OMe)](CF_3SO_3)_3$	13280	13755	0.06	0.34	11	62	76

[a]
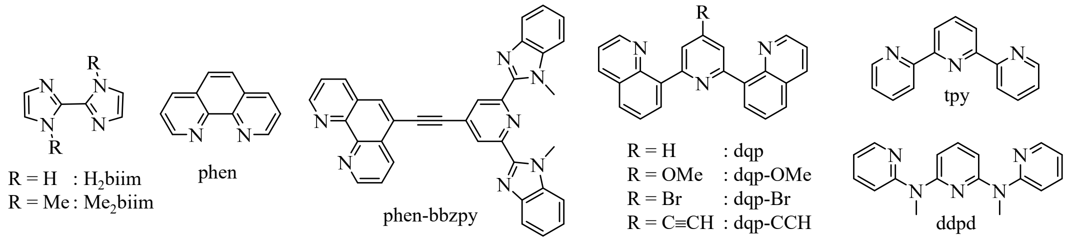

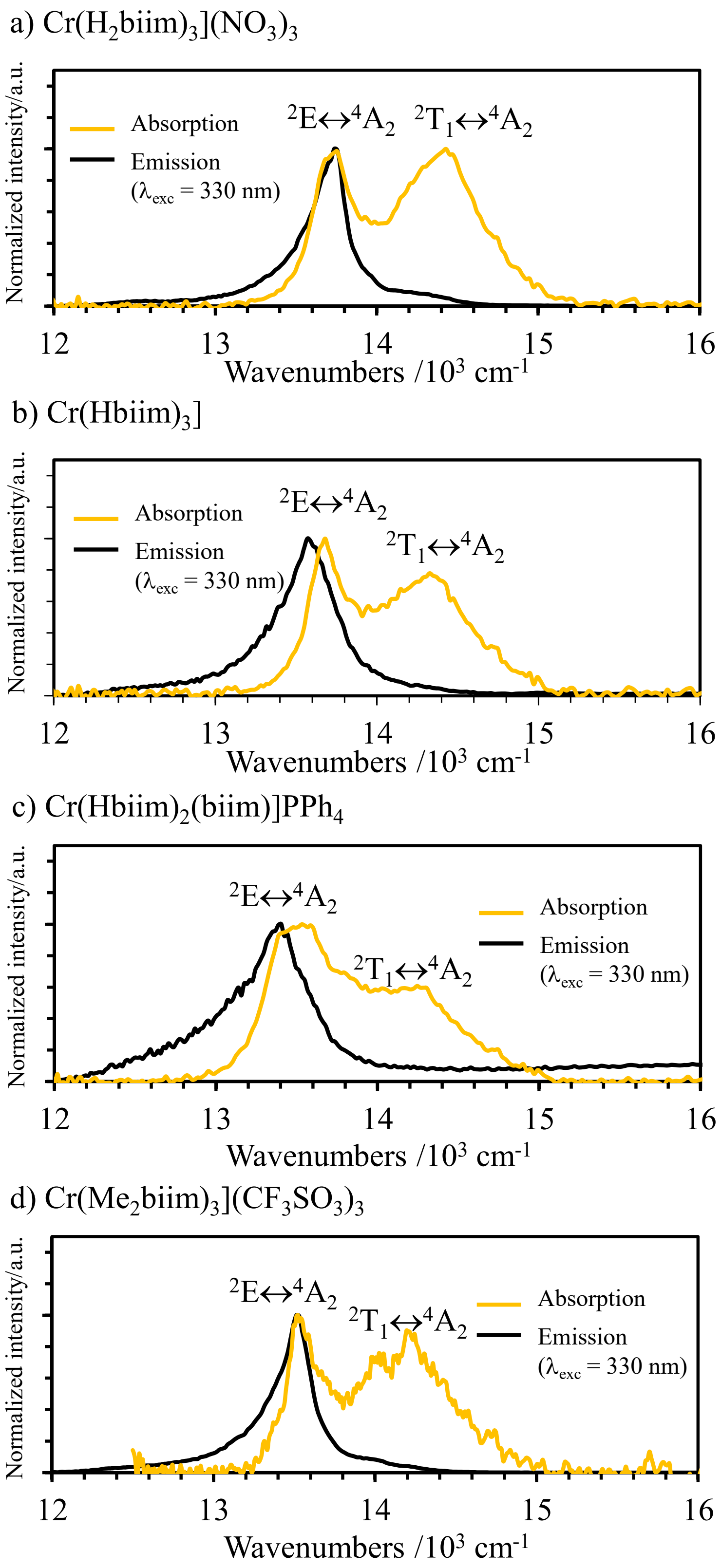

Figure S46 Overlay of NIR absorption and emission spectra of a) $[Cr(H_2biim)_3](NO_3)_3$, b) $[Cr(Hbiim)_3]$, c) $[Cr(Hbiim)_2(biim)](PPh_4)$ and d) $[Cr(Me_2biim)_3](CF_3SO_3)_3$ in the solid state at 293 K.

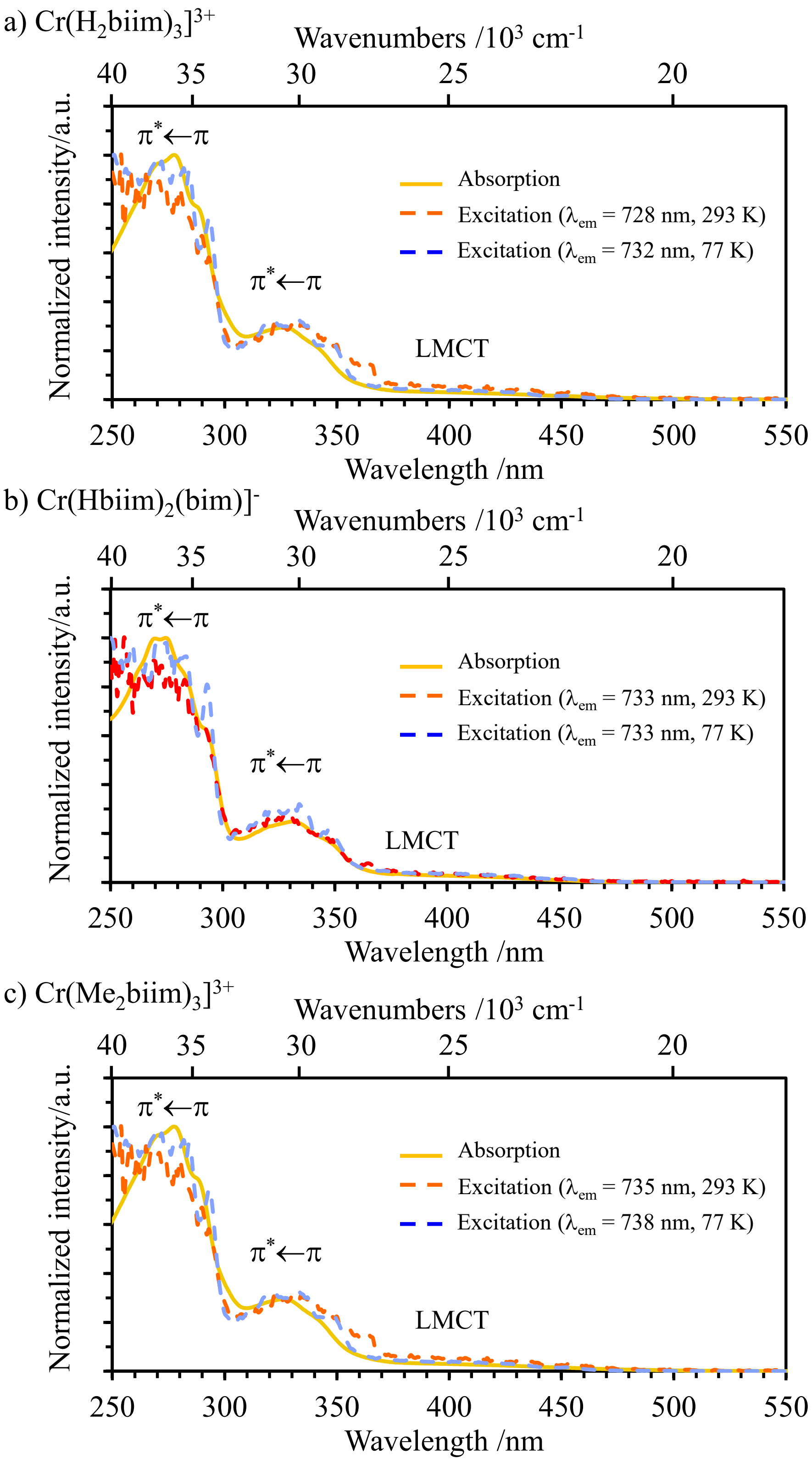

Figure S47 Overlay of absorption (MeOH) and excitation (MeOH at 293 K, MeOH:H_2O 1:1 at 77 K) spectra of a) $[Cr(H_2biim)_3](NO_3)_3$, b) $[Cr(Hbiim)_2(biim)](PPh_4)$ and c) $[Cr(Me_2biim)(CF_3SO_3)_3$.

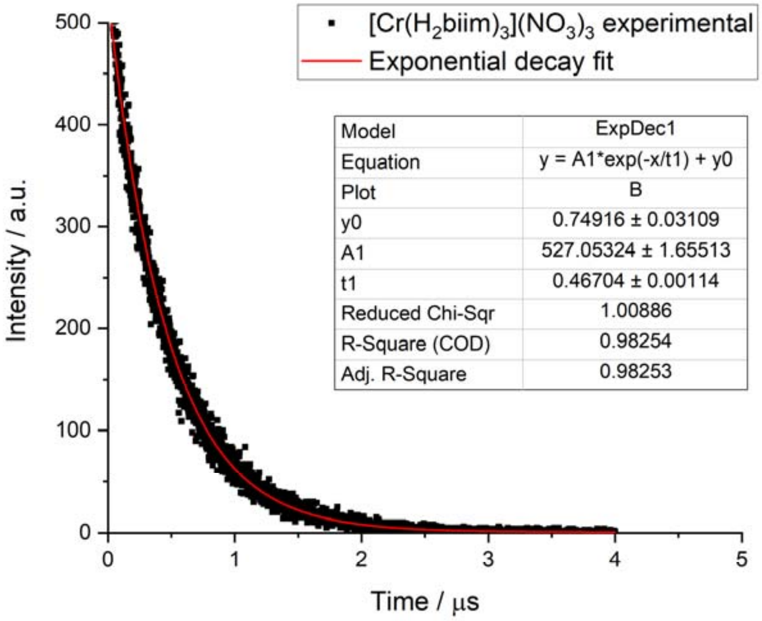

Figure S48 Emission decay curve as function of time for the emission of Cr($^2E \rightarrow {}^4A_2$) transition of $[Cr(H_2biim)_3](NO_3)_3$ in MeOH at 293K in deaerated solutions. λ_{exc} = 355 nm; λ_{em} = 728 nm.

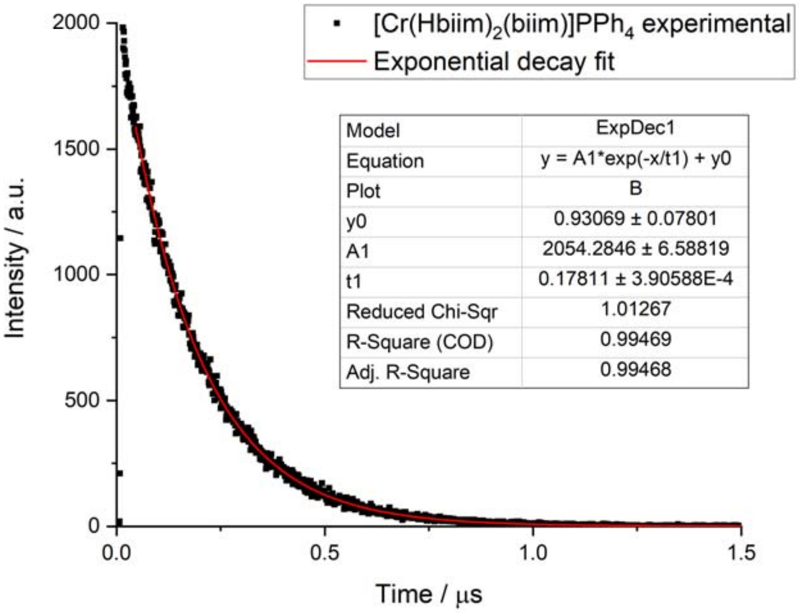

Figure S49 Emission decay curve as function of time for the emission of Cr($^2E \rightarrow {}^4A_2$) transition of $[Cr(Hbiim)_2(biim)](PPh_4)$ in MeOH at 293K in deaerated solutions. λ_{exc} = 355 nm ; λ_{em} = 733 nm.

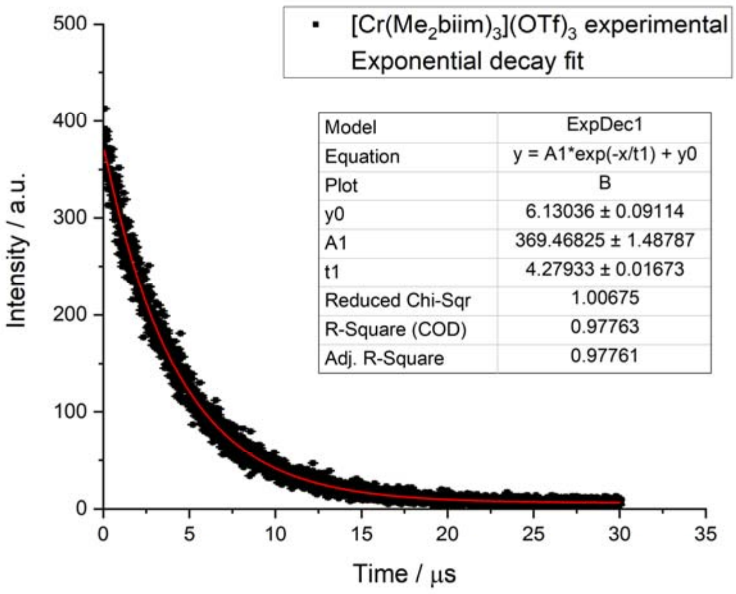

Figure S50 Emission decay curve as function of time for the emission of Cr($^2E \rightarrow ^4A_2$) transition of [Cr(Me_2biim)(CF_3SO_3)$_3$ in MeOH at 293K in deaerated solutions. λ_{exc} = 355 nm ; λ_{emi} = 738 nm.

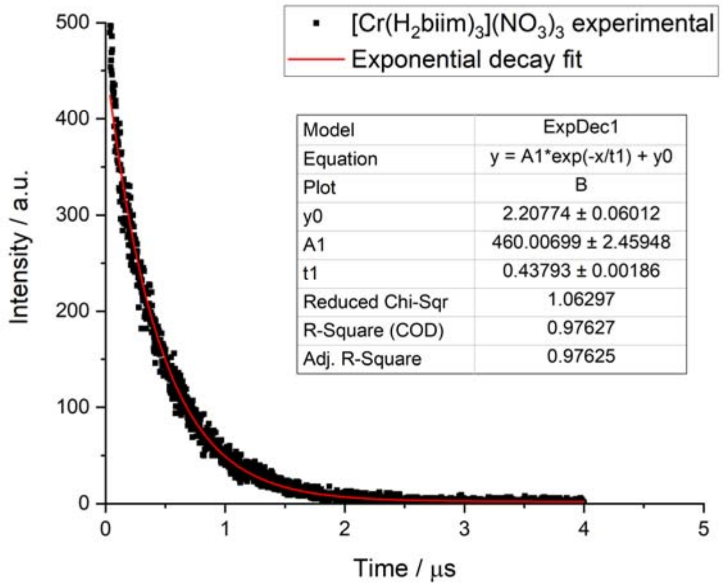

Figure S51 Emission decay curve as function of time for the emission of Cr($^2E \rightarrow ^4A_2$) transition of [Cr(H_2biim)$_3$](NO_3)$_3$ in MeOH at 293K in air-equilibrated solutions. λ_{exc} = 355 nm ; λ_{em} = 728 nm.

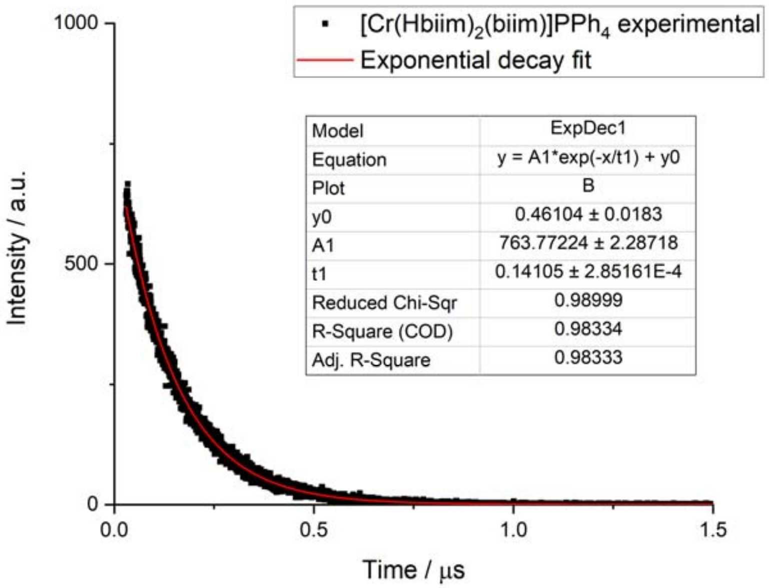

Figure S52 Emission decay curve as function of time for the emission of Cr($^2E \rightarrow ^4A_2$) transition of [Cr(Hbiim)$_2$(biim)](PPh$_4$) in MeOH at 293K in air-equilibrated solutions. λ_{exc} = 355 nm ; λ_{em} = 733 nm.

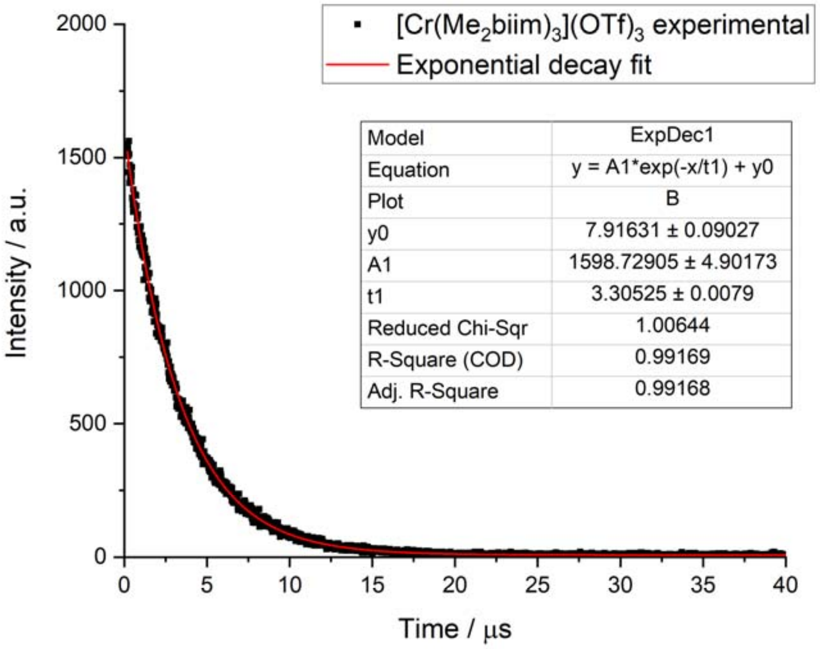

Figure S53 Emission decay curve as function of time for the emission of Cr($^2E \rightarrow ^4A_2$) transition of [Cr(Me$_2$biim)(CF$_3$SO$_3$)$_3$ in MeOH at 293K in air-equilibrated solutions. λ_{exc} = 355 nm ; λ_{em} = 738 nm.

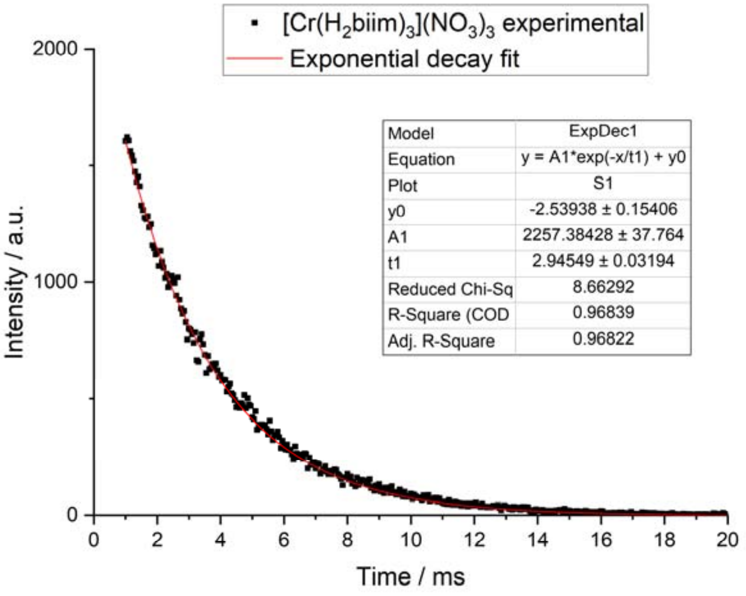

Figure S54 Emission decay curve as function of time for the emission of Cr($^2E \rightarrow ^4A_2$) transition of $[Cr(H_2biim)_3](NO_3)_3$ in MeOH/H_2O (1:1) at 77 K. λ_{exc} = 330 nm ; λ_{em} = 732 nm.

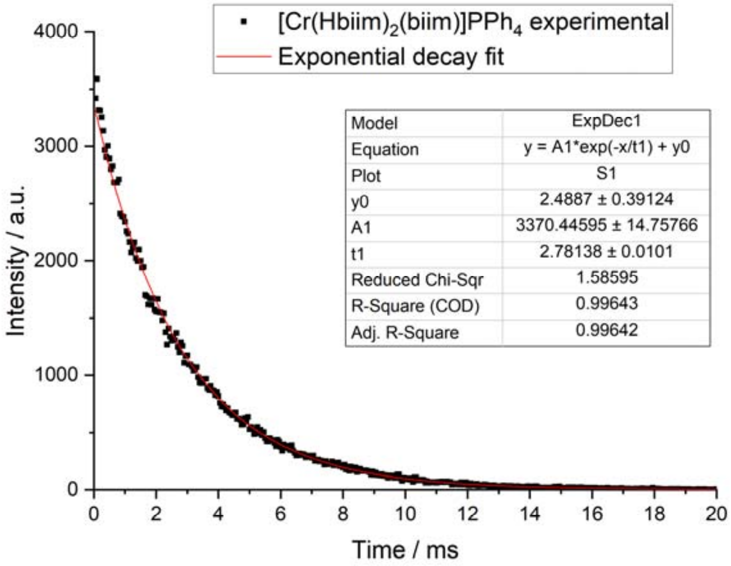

Figure S55 Emission decay curve as function of time for the emission of Cr($^2E \rightarrow ^4A_2$) transition of $[Cr(Hbiim)_2(biim)](PPh_4)$ in MeOH/H_2O (1:1) at 77 K. λ_{exc} = 330 nm ; λ_{em} = 733 nm.

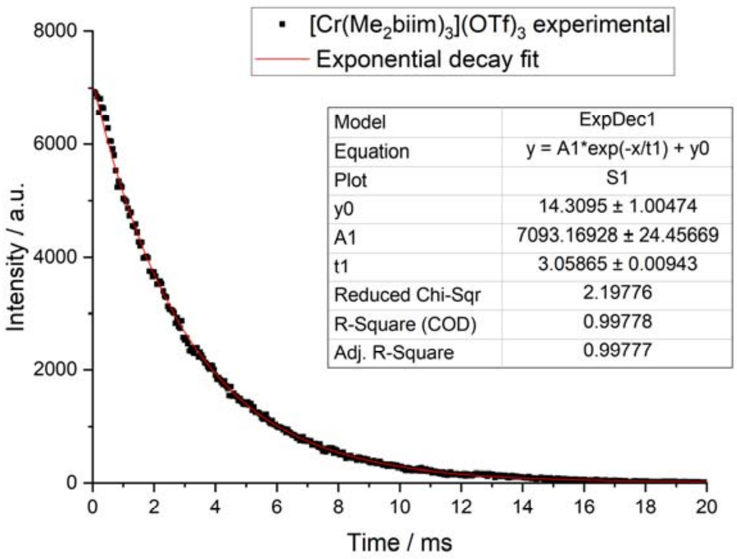

Figure S56 Emission decay curve as function of time for the emission of Cr(^{2}E→^{4}A$_2$) transition of [Cr(Me$_2$biim)(CF$_3$SO$_3$)$_3$ in MeOH/H$_2$O (1:1) at 77 K. λ_{exc} = 330 nm ; λ_{em} = 738 nm.

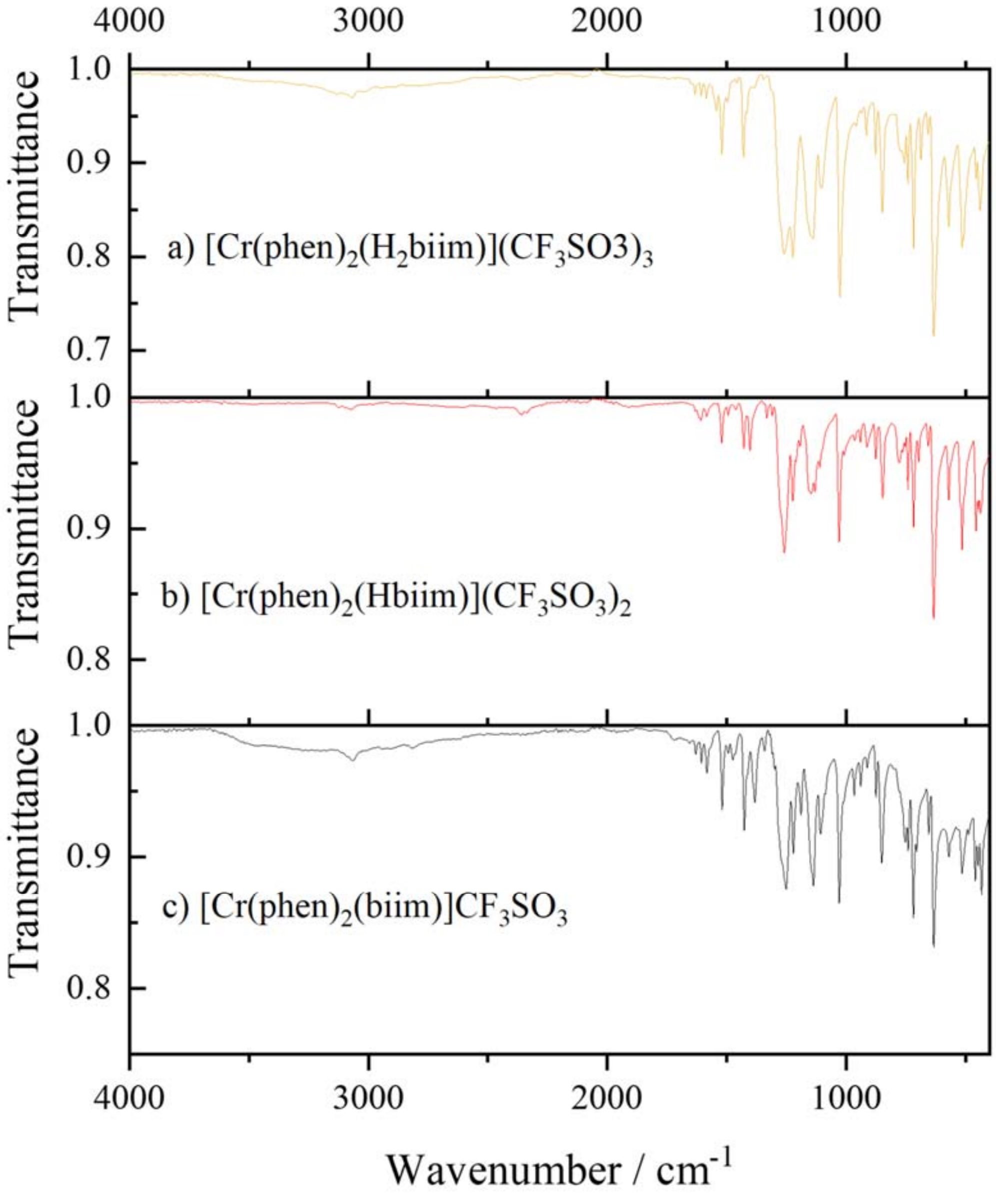

Figure S57 Stacked solid state FT-IR spectrum of a) $[Cr(phen)_2(H_2biim)](CF_3SO_3)_3(H_2O)_{0.25}$ (orange), b) $[Cr(phen)_2(Hbiim)](CF_3SO_3)_2(H_2O)_{0.5}$ (red) and $[Cr(phen)_2(biim)](CF_3SO_3)(CH_3OH)_{1.5}$ (black).

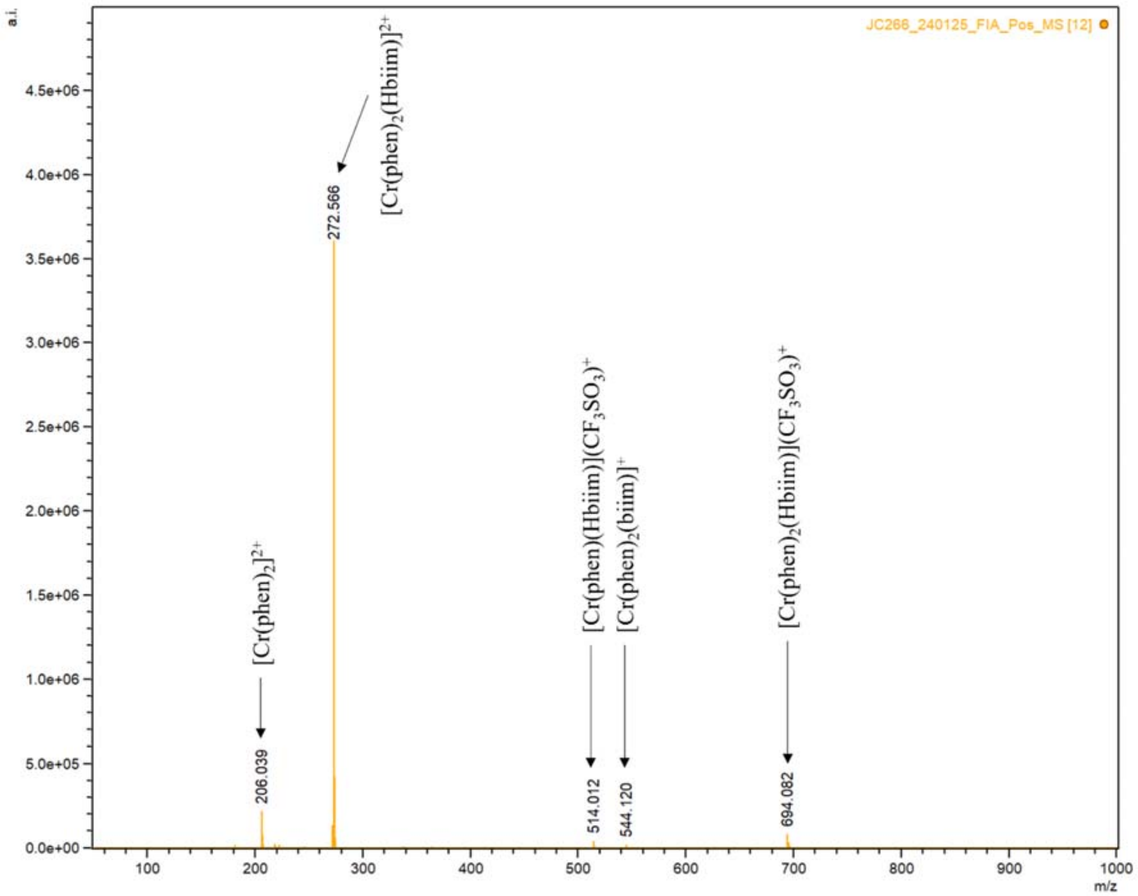

Figure S58 ESI-MS full spectrum of the complex $[Cr(phen)_2(H_2biim)](CF_3SO_3)_3$ in CH_3CN. The ESI-MS spectra of complexes $[Cr(phen)_2(Hbiim)](CF_3SO_3)_2$ and $[Cr(phen)_2(biim)](CF_3SO_3)$ are not depicted because they are identical to the spectrum of $[Cr(phen)_2(H_2biim)](CF_3SO_3)_3$.

Table S41 Observed *m*/*z*, intensity and attribution of each peak observed in the ESI-MS spectrum of $[Cr(phen)_2(H_2biim)](CF_3SO_3)_3$.

Peak n°	*m*/*z*	I / a.u.	Attribution (fragment)	Molecular formula	Calc. *m*/*z*
1	206.039	2.16E+05	$[Cr(phen)_2]^{2+}$	$C_{24}H_{16}CrN_4^{2+}$	206.0385
2	272.566	3.56E+06	$[Cr(phen)_2(Hbiim)]^{2+}$	$C_{30}H_{21}CrN_8^{2+}$	272.5641
3	514.012	3.94E+04	$[Cr(phen)(Hbiim)](CF_3SO_3)^+$	$C_{19}H_{13}CrF_3N_6O_3S^+$	514.0122
4	544.120	1.79E+04	$[Cr(phen)_2(biim)]^+$	$C_{30}H_{20}CrN_8^+$	544.1211
5	694.082	7.90E+04	$[Cr(phen)_2(Hbiim)](CF_3SO_3)^+$	$C_{31}H_{21}CrF_3N_8O_3S^+$	694.0810

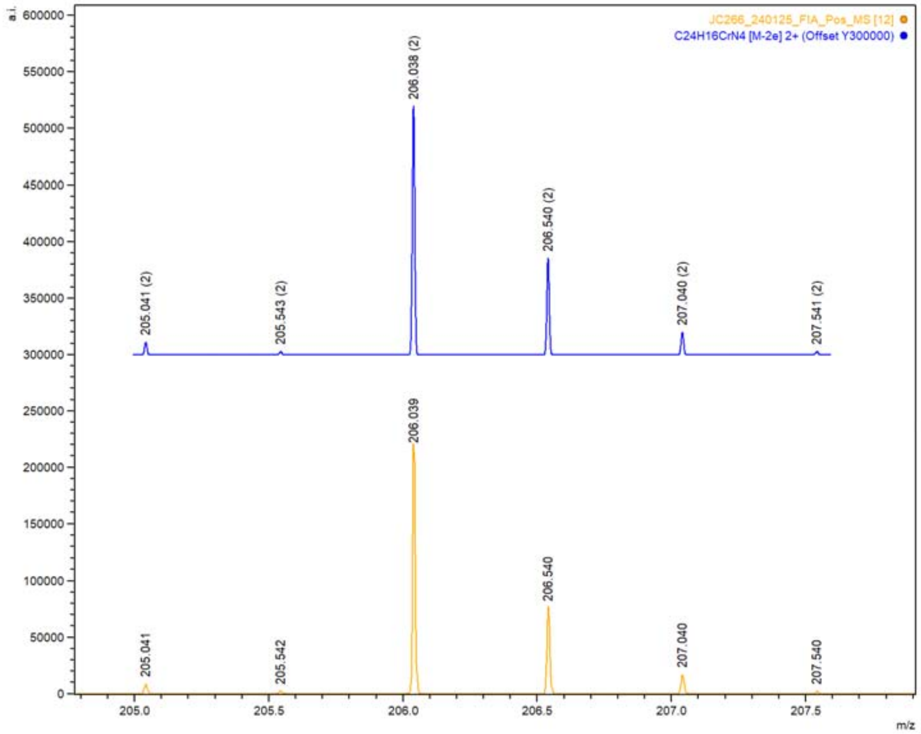

Figure S58: Experimental ESI-MS spectrum of complex [Cr(phen)$_2$(H$_2$biim)](CF$_3$SO$_3$)$_3$ in CH$_3$CN (orange trace). Calculated peaks for [Cr(phen)$_2$]$^{2+}$, $C_{24}H_{16}CrN_4{}^{2+}$ (blue trace).

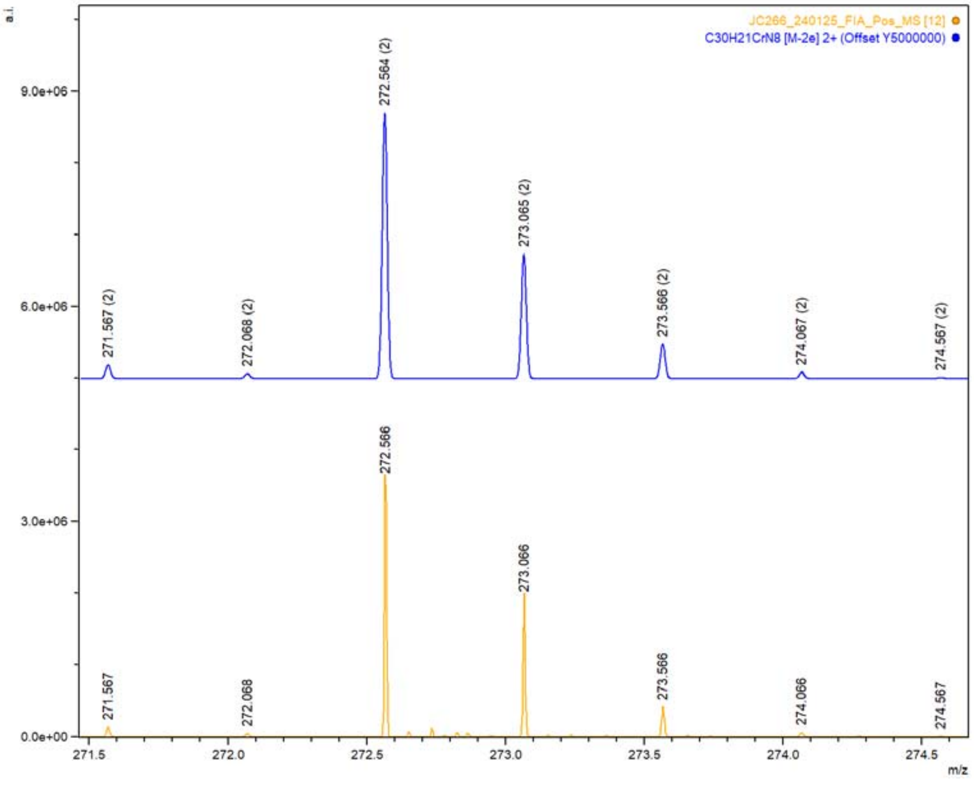

Figure S59 Experimental ESI-MS spectrum of [Cr(phen)$_2$(H$_2$biim)](CF$_3$SO$_3$)$_3$ in CH$_3$CN (orange trace)1 (orange). Calculated peaks for [Cr(phen)$_2$(Hbiim)]$^{2+}$, $C_{30}H_{21}CrN_8{}^{2+}$ (blue trace).

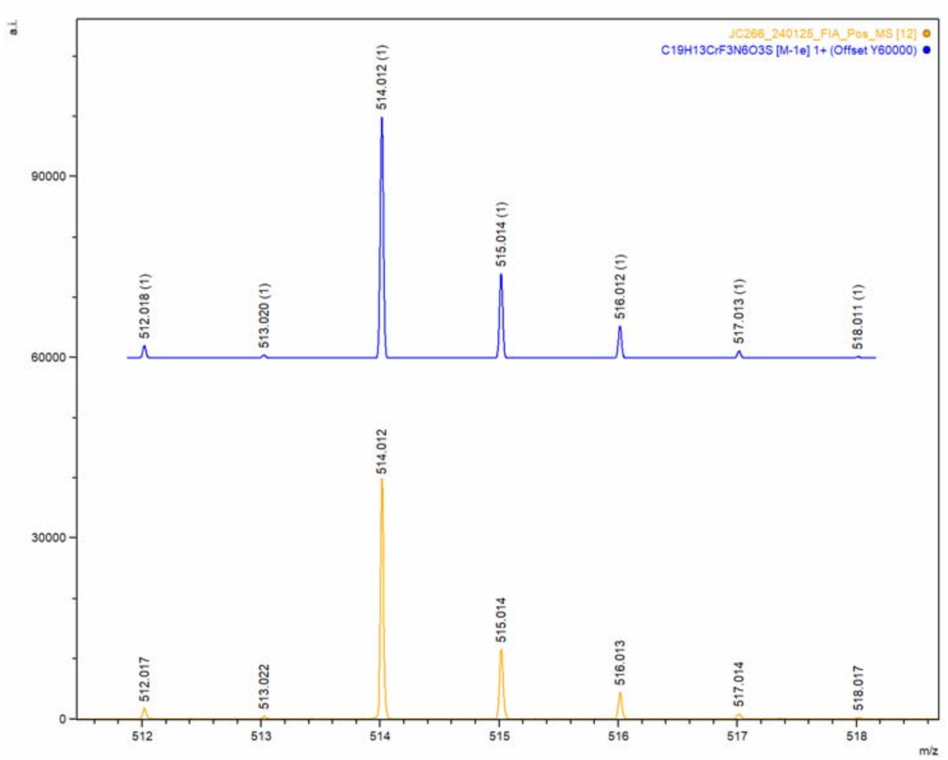

Figure S60 Experimental ESI-MS spectrum of $[Cr(phen)_2(H_2biim)](CF_3SO_3)_3$ in CH_3CN (orange trace). Calculated peaks for $[Cr(phen)(Hbiim)](CF_3SO_3)^+$, $C_{19}H_{13}CrF_3N_6O_3S^+$ (blue trace).

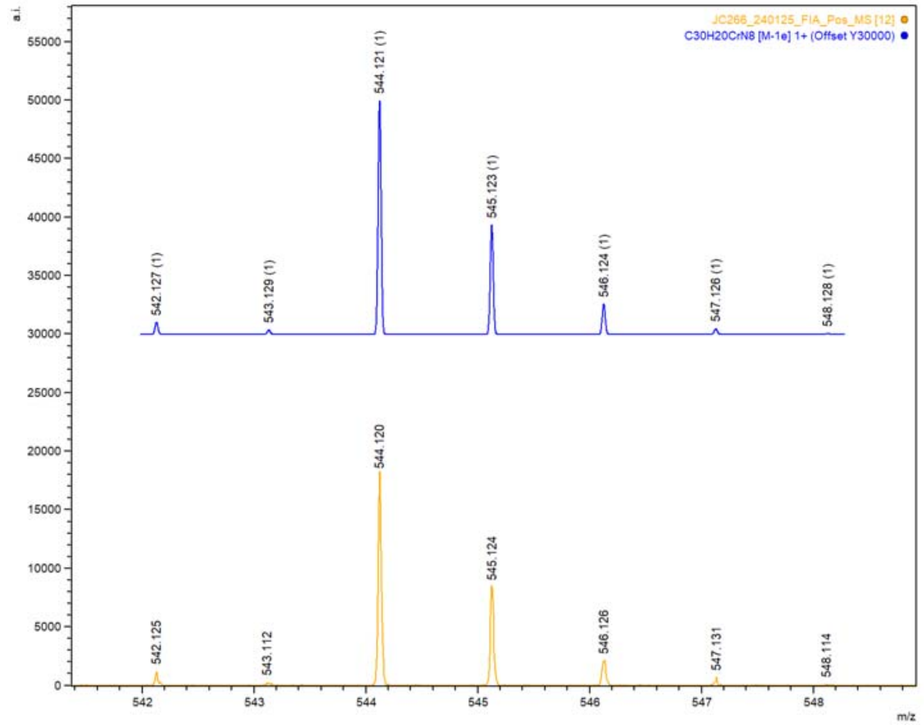

Figure S61 Experimental ESI-MS spectrum of $[Cr(phen)_2(H_2biim)](CF_3SO_3)_3$ in CH_3CN (orange trace). Calculated peaks for $[Cr(phen)_2(biim)]^+$, $C_{30}H_{20}CrN_8^+$ (blue trace).

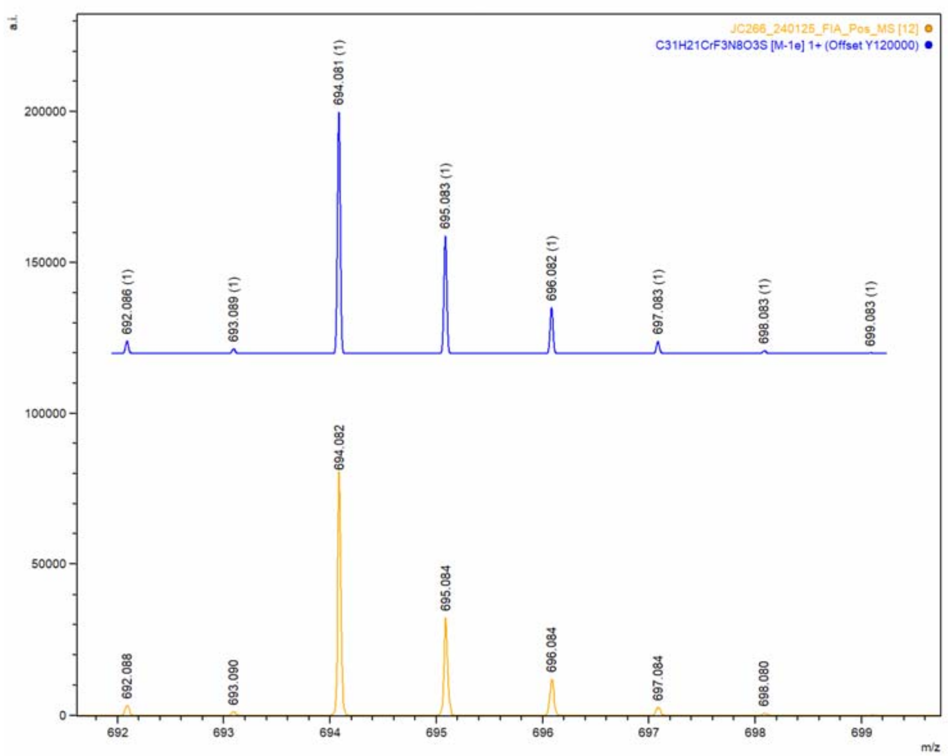

Figure S62 Experimental ESI-MS spectrum of $[Cr(phen)_2(H_2biim)](CF_3SO_3)_3$ in CH_3CN (orange trace). Calculated peaks for $[Cr(phen)_2(Hbiim)](CF_3SO_3)^+$, $C_{31}H_{21}CrF_3N_8O_3S^+$ (blue trace).

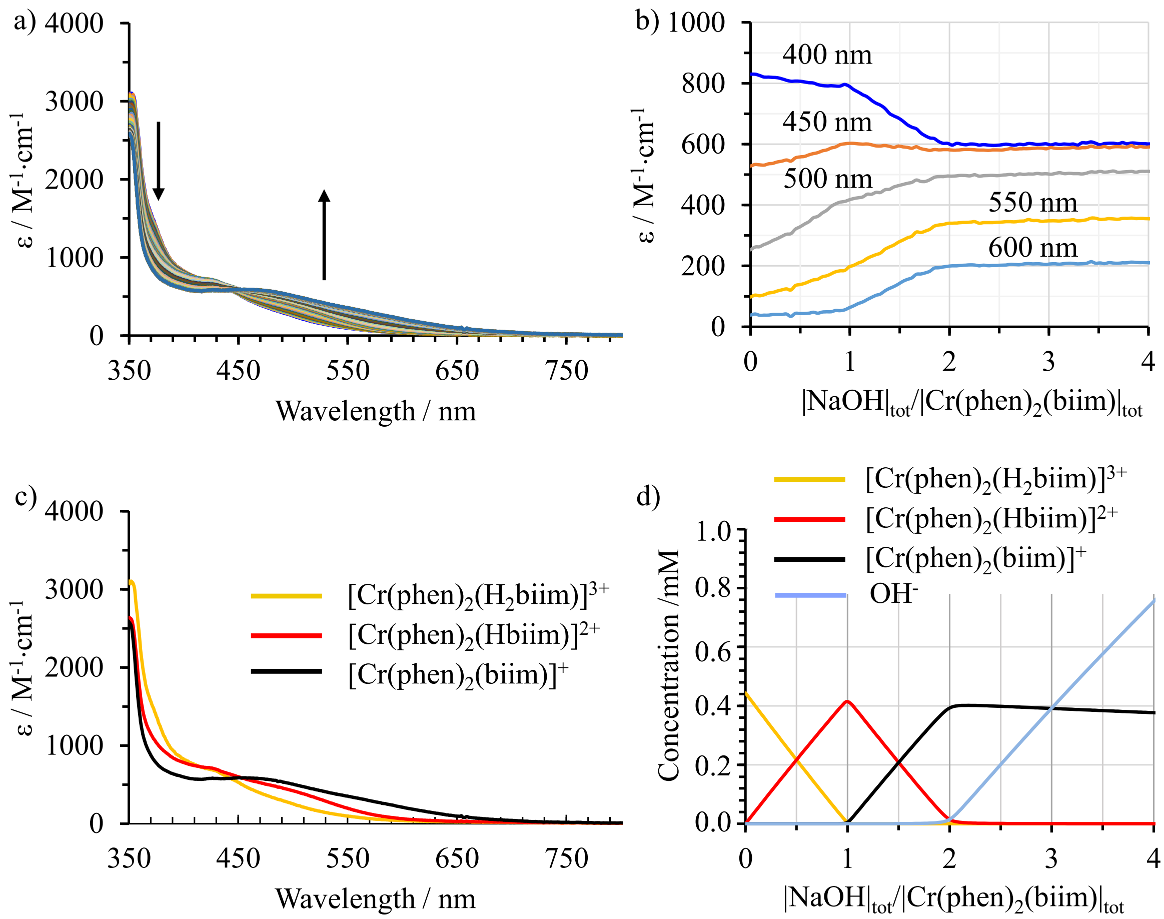

Figure S63 Variation of a) absorption spectra and b) molar extinction coefficients after each addition of NaOH; c) deconvoluted absorption spectra of $[Cr(phen)_2(H_2biim)]^{3+}$, $[Cr(phen)_2(Hbiim)]^{2+}$ and $[Cr(phen)_2(biim)]^{+}$ in H_2O and d) calculated concentration profiles of each species at each addition (bottom right).

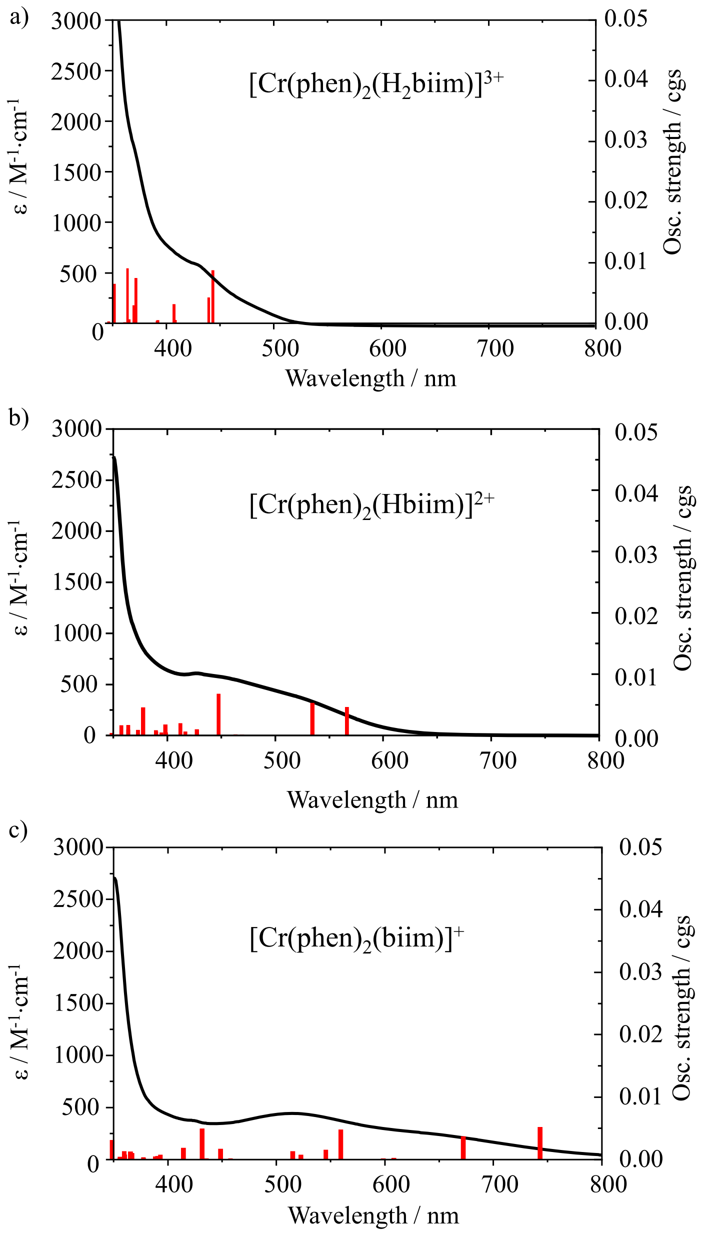

Figure S64 Experimental (black traces) visible absorption spectra of a) $[Cr(phen)_2(H_2biim)]^{3+}$, b) $[Cr(phen)_2(Hbiim)]^{2+}$ and c) $[Cr(phen)_2(biim)]^{+}$ in CH_3CN and calculated oscillatory strengths (red bars) of the computed electronic transitions.

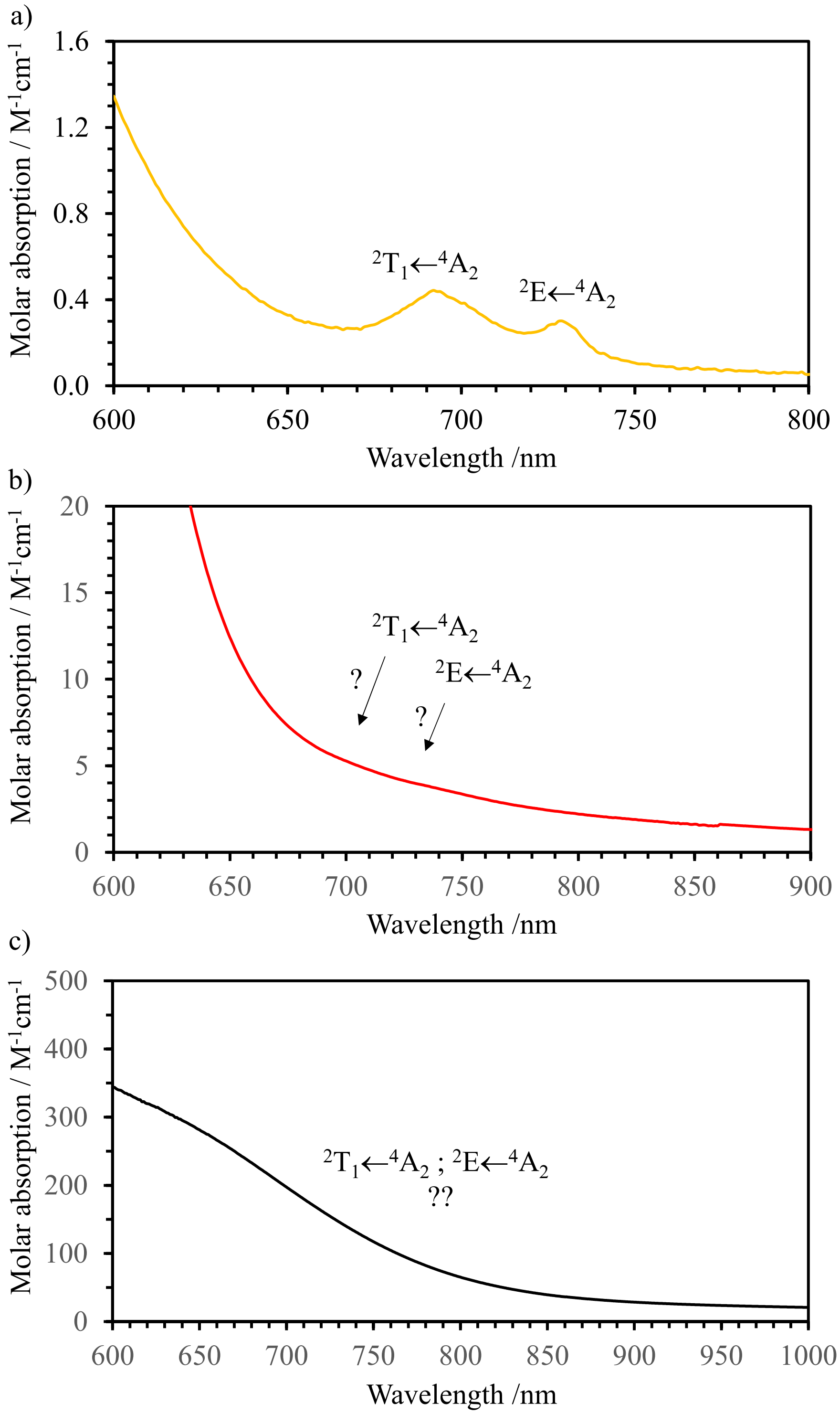

Figure S65 NIR absorption spectra of a) $[Cr(phen)_2(H_2biim)]^{3+}$, b) $[Cr(phen)_2(Hbiim)]^{2+}$ (red trace) and c) $[Cr(phen)_2(biim)]^{+}$ (black trace) in CH_3CN ($c \approx 10^{-2}$ mol·L^{-1}).

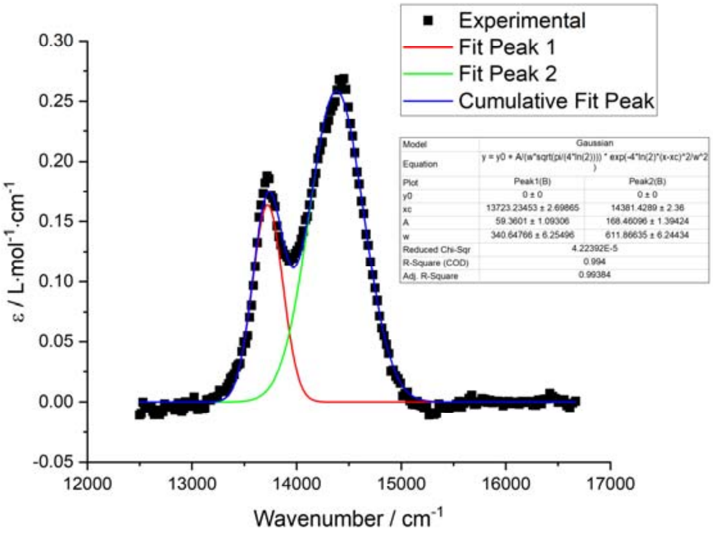

Figure S66 Gaussian deconvolution of the absorption spectrum of [Cr(phen)$_2$(H$_2$biim)](CF$_3$SO$_3$)$_3$ in CH$_3$CN, c = 1.03·10^{-2} M.

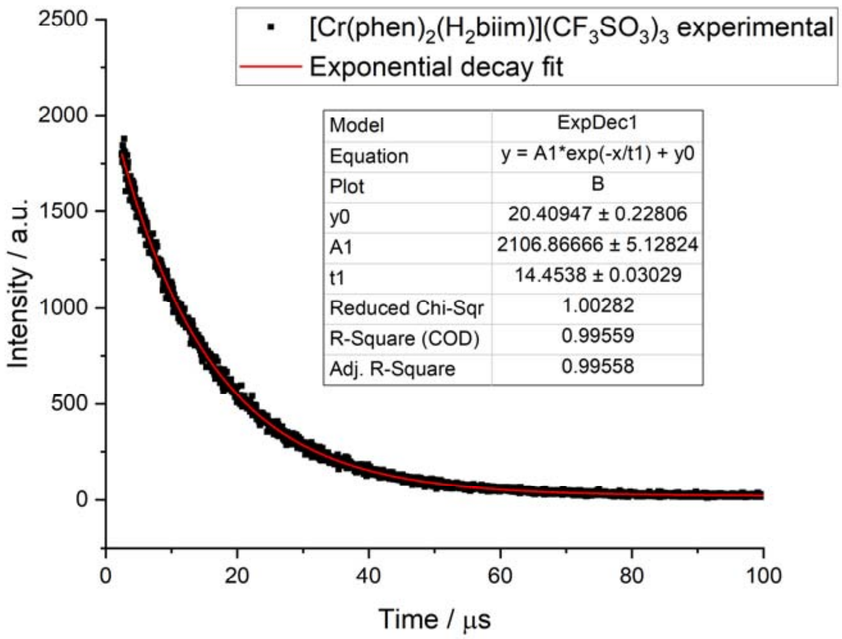

Figure S67 Emission decay curve as function of time for the emission of Cr($^2E \rightarrow ^4A_2$) transition of $[Cr(phen)_2(H_2biim)]^{3+}$ in CH_3CN at 293K in aerated solution. λ_{exc} = 355 nm; λ_{em} = 730 nm; $c = 1.0 \cdot 10^{-4}$ M.

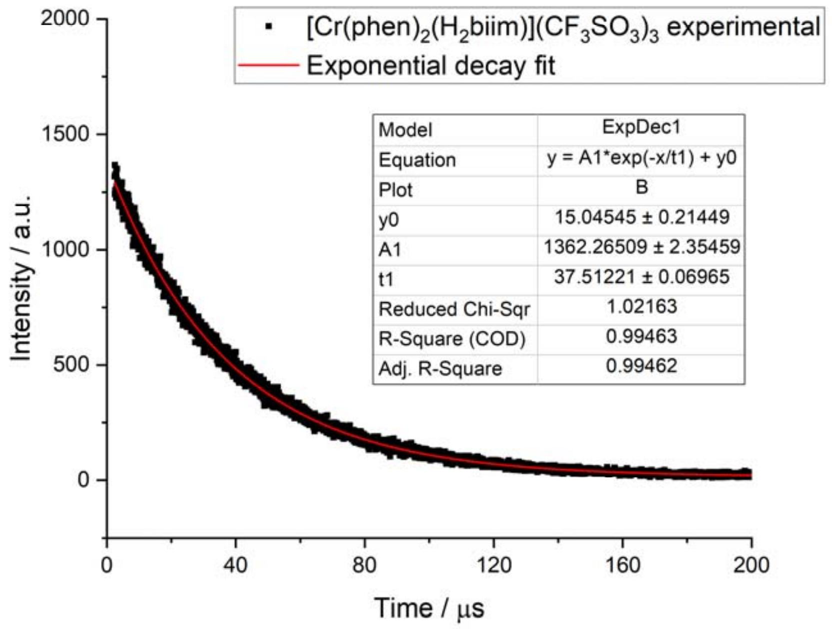

Figure S68 Emission decay curve as function of time for the emission of Cr($^2E \rightarrow ^4A_2$) transition of $[Cr(phen)_2(H_2biim)]^{3+}$ in CH_3CN at 293K in deaerated solution. λ_{exc} = 355 nm; λ_{em} = 730 nm; c = $1.0 \cdot 10^{-4}$ M.

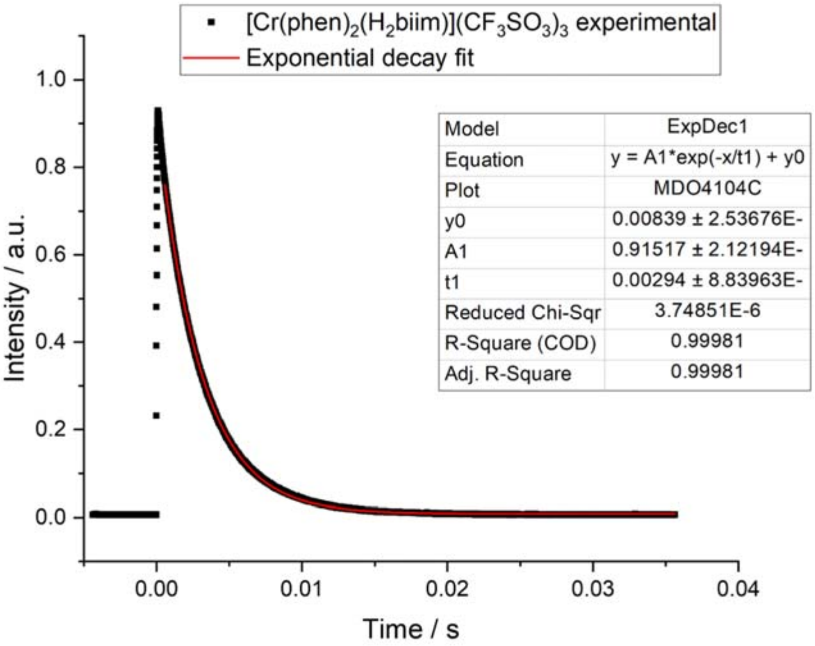

Figure S69 Emission decay curve as function of time for the emission of Cr($^2E \rightarrow ^4A_2$) transition of $[Cr(phen)_2(H_2biim)]^{3+}$ in CH_3CN/C_2H_5CN 6:4 at 77K in frozen solution. λ_{exc} = 355 nm; λ_{em} = 730 nm; $c = 1.0 \cdot 10^{-4}$ M.

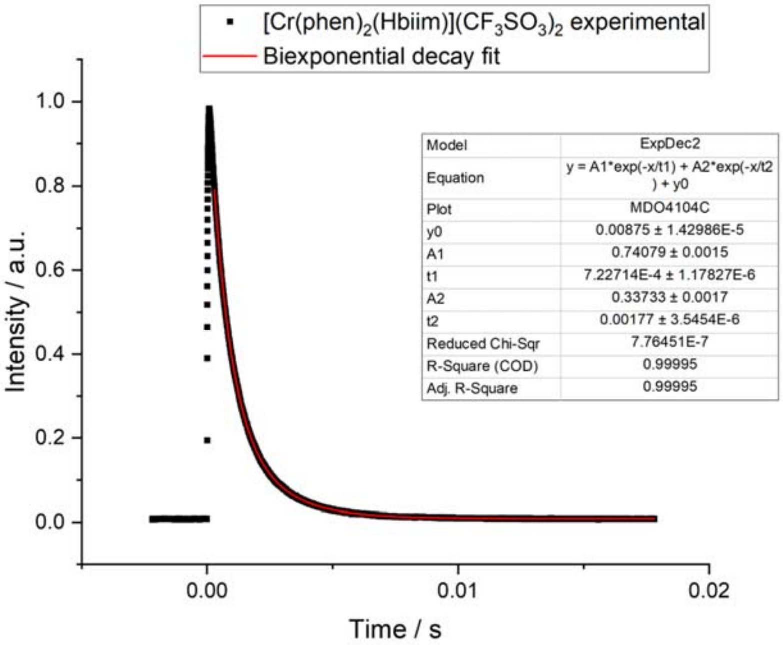

Figure S70 Emission decay curve as function of time for the emission of Cr($^2E \rightarrow ^4A_2$) transition of $[Cr(phen)_2(Hbiim)]^{2+}$ in CH_3CN/C_2H_5CN 6:4 at 77K in frozen solution. λ_{exc} = 355 nm; λ_{em} = 750 nm; $c = 1.0 \cdot 10^{-4}$ M.

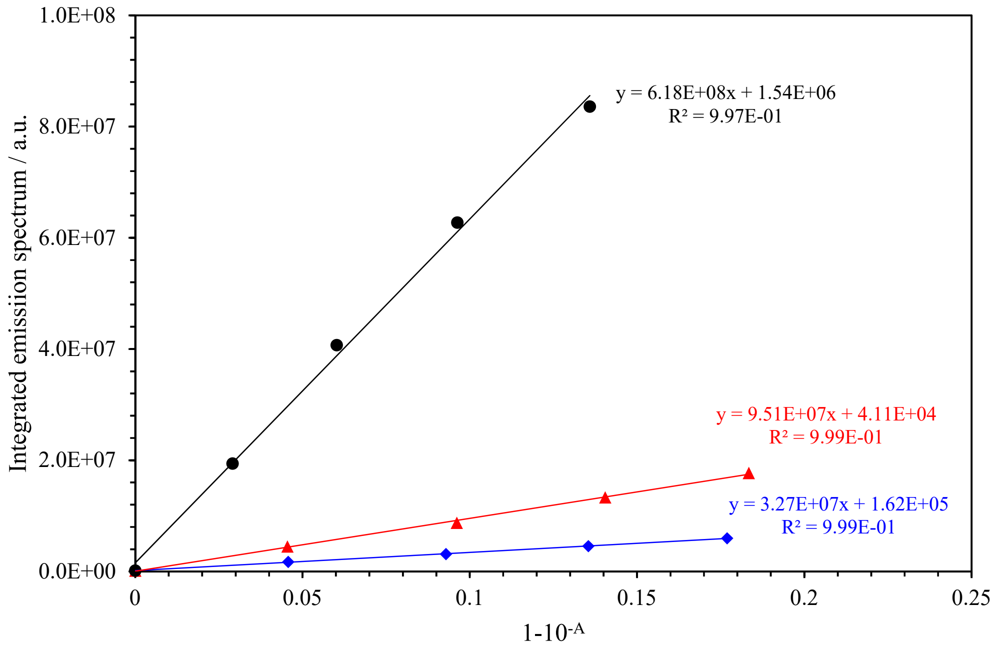

Figure S71 Plot of $\int \boldsymbol{I_i(\lambda)d\lambda}$ as a function of $(\mathbf{1-10^{-A_{(450\,nm)i}}})$ for complex $[Cr(phen)_2(H_2biim)]^{3+}$ in aerated (blue trace) and deaerated (red trace) acetonitrile, and for $[Ru(bpy)_3]^{2+}$ in aerated acetonitrile (black). The relative method has been used:

$$\Phi_{\mathbf{complex}} = \frac{\int \boldsymbol{I}_{\mathbf{complex}}(\boldsymbol{\lambda})\mathbf{d}\boldsymbol{\lambda}}{\int \boldsymbol{I}_{\mathbf{ref}}(\boldsymbol{\lambda})\mathbf{d}\boldsymbol{\lambda}}\left(\frac{\boldsymbol{n}_{\mathbf{complex}}}{\boldsymbol{n}_{\mathbf{ref}}}\right)^2 \frac{\mathbf{1-10}^{-\boldsymbol{A}_{\mathbf{ref}}}}{\mathbf{1-10}^{-\boldsymbol{A}_{\mathbf{complex}}}}\Phi_{\mathbf{ref}} = \frac{\boldsymbol{slope_{complex}}}{\boldsymbol{slope_{ref}}}\Phi_{\mathbf{ref}}$$

where $\int \boldsymbol{I_i(\lambda)}\mathbf{d}\boldsymbol{\lambda}$ is the integrated emission spectrum of the compound *i*, *n* is the refractive index of the solvent and A_i is the absorbance of the solution of compound *i* at the excitation wavelength (450 nm here). The reference compound was $[Ru(bpy)_3](PF_6)_2$ for which the quantum yield is known to be $1.8 \cdot 10^{-2}$ in aerated acetonitrile after excitation at 450 nm. (H. Ishida, S. Tobita, Y. Hasegawa, R. Katoh and K. Nozaki, *Coord. Chem. Rev.*, 2010, **254**, 2449-2458).
